# Ancient Rhamnaceae flowers impute an origin for flowering plants exceeding 250-million-years ago

**DOI:** 10.1016/j.isci.2022.104642

**Published:** 2022-06-18

**Authors:** Tianhua He, Byron B. Lamont

**Affiliations:** 1 College of Science, Health, Engineering and Education, Murdoch University, Murdoch, WA, Australia; 2 Ecology Section, School of Molecular and Life Sciences, Curtin University, Perth, WA 6003, Australia

**Keywords:** Paleontology, Earth history, Plants, Plant evolution

## Abstract

Setting the molecular clock to newly described 100-million-year-old flowering shoots of *Phylica* in Burmese amber enabled us to recalibrate the phylogenetic history of Rhamnaceae. We traced its origin to ∼260 million years ago (Ma) that can explain its migration within and beyond Gondwana since that time and implies an origin for flowering plants that stretches well beyond 290 Ma. Ancestral trait assignments also revealed that hard-seededness, fire-proneness, and to a lesser extent, heat-released seed dormancy, have a similarly long history in this clade.

## Highlights

• 100-million-year-old fossils of *Phylica* (Rhamnaceae) have recently been described
• Phylogenetic reconstruction using them reveals a crown age for Rhamnaceae at 259 Ma
• Ancestral trait reconstruction shows the associated vegetation was fire prone 259 Ma
• These are the oldest flowering-plant ages and fire-related traits obtained so far

## Graphical abstract

**Figure:**
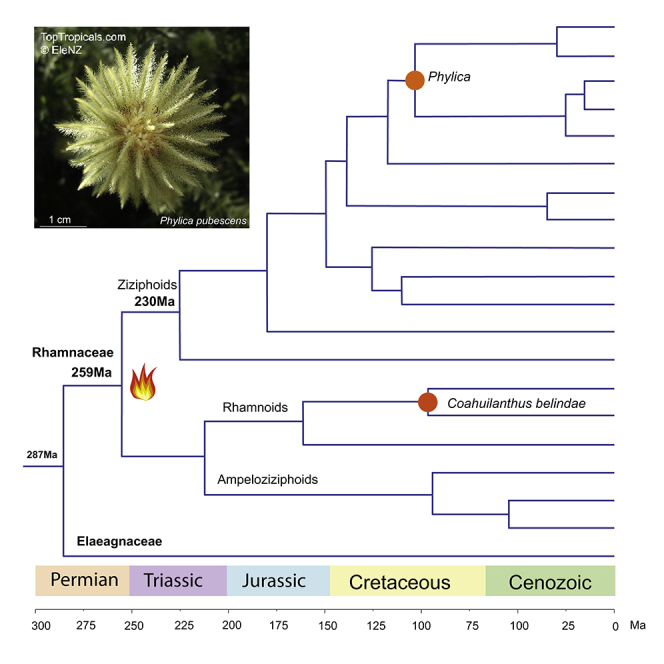

## Introduction

The more intensively the evolutionary histories of clades are studied, the further back in time their origin is pushed. A case in point are the flowering plants (Angiosperms) whose origin has gradually been taken from the mid-Cretaceous, as favored just a few years ago, to the Jurassic, then the Triassic and now possibly even into the Permian (Li et al., 2019; Silvestro et al., 2021). In this regard, the study of Shi et al. (2022) is a huge step forward as they provide convincing evidence for the presence of intact flowering shoots belonging to the *Phylica* clade (family Rhamnaceae, subfamily Ziziphoideae) in Burmese amber. Some specimens (*Phylica piloburmensis*) were dated to 99 million years ago (Ma) and others (*Eophylica priscastellata*) at 110−99 Ma. Extant species of the tribe Phylicieae are restricted to South Africa, and to a lesser extent Madagascar and a few remote islands nearby. The previous oldest fossils were pressed flowers in the subfamily Rhamnoideae from Mexico dated at 70.6 Ma (Calvillo-Canadell and Cevallos-Ferriz, 2007; Hauenschild et al., 2018).

But how did *Phylica* get to Myanmar if it is essentially a South African clade? Of the possible routes, Shi et al., 2022 prefer the attachment of the West Burma plate (corresponding to present-day Myanmar) to the northeast corner of Greater India. Once the Indian plate, together with Madagascar and Sri Lanka, severed its link with Africa at ∼115 Ma, it rapidly drifted north to collide with outheast Asia at 60−50 Ma (Reeves, 2018). The alternative view is that the West Burma plate was attached to the northwest edge of the Australian plate and separated at 145 Ma and eventually lodged between India and Asia 40 Ma (Young et al., 2019). Both scenarios require at least the stem of the *Phylica* clade to have existed before separation from the more southerly plate, on the assumption that transoceanic dispersal is unlikely (because of its tiny, poorly-dispersible, arillate seeds).

Clues to the preferred pathway should be retrievable from a dated phylogeny of the entire Rhamnaceae using these new fossil dates. In addition, because the molecular phylogeny of Rhamnaceae is well-known (Onstein and Linder, 2016; Hauenschild et al., 2018), it should be possible to trace this family to separation from its sister clade, providing information on the age of these two flowering plant clades. Because this approach is different from previous methods based essentially on palynomorphs and extrapolations from fossil diversity estimates (Li et al., 2019; Silvestro et al., 2021) it can provide an independent assessment of the age of at least two representative flowering-plant families. We undertook such an exercise here. In addition, because the *Phylica* specimens were associated with charcoal, and 30 of the 31 genera in Ziziphoideae currently have some or all of their species in fire-prone environments, this raises the possibility that this clade arose in a fire-prone environment (He et al., 2012; Lamont et al., 2019). Thus, we took the opportunity to perform ancestral trait reconstructions on the phylogeny using information available about species hard-seededness, fire-released seed dormancy, and fire-proneness to see how far back these could be traced.

## Materials and Methods

Available DNA sequences at seven loci for one or two species from each genus recognised in the Rhamnaceae (except for *Phylica* that includes five species in the analysis), and three species from Elaeagnaceae as the outgroup, were obtained from NCBI (https://www.ncbi.nlm.nih.gov). Phylogenetic reconstruction and dating were carried out with BEAST v1.10.4 (Suchard et al., 2018) using an uncorrelated relaxed molecular clock with two calibration points. The crown age of *Phylica* was set at a conservative mean of 104.5 Ma (midpoint of 99–110 Ma, Shi et al., 2022). The common crown of Rhamneae and Maesopsideae was set at a mean of 70.6 Ma using the compressed-flower fossils of *Coahuilanthus belindae* (Calvillo-Canadell and Cevallos-Ferriz, 2007 following Hauenschild et al., 2018). We used a normal function as priors at both calibration points, as priors with a normal distribution (with 10% of the mean as SD) allow the model to traverse a broader time range to better accommodate uncertainties in both the fossil age and divergence time between key taxon groups. GTR substitution and Yule process evolution models were used. Twenty million iterations over five runs were implemented. For ease of interpretation, species were collapsed to tribes for display. Ages are presented as the median ± 95% highest posterior density interval. The maximum clade credibility tree containing all taxa (Figure S1) and the aligned DNA matrix (Data S1) are given as Supplemental Information.

Fire-related traits (presence/absence) for each species in the molecular phylogeny were collated from data in Pausas and Lamont (2022) and numerous websites (identity of these additional sources available on request). Hard-seededness was gauged as impermeable to water or evidence of a sclerified seed coat (these sometimes referred to the endocarp and not the testa), heat-released dormancy is given as fire-stimulated germination in the results and required seeds treated with fire-type heat to have exceeded germination of the controls, and fire-proneness refers to occurrence of the species in vegetation likely to burn within its lifetime. Where there were conflicting reports or there was doubt [e.g., occurs on the edge of rainforests under a monsoon (i.e., fire-prone) climate] it was assigned to both trait states. If data were lacking for a given species but there was knowledge for other species in the genus, they were assigned these trait states on the assumption that they were diagnostic for the genus. Ancient trait reconstruction was conducted following a maximum likelihood probability model and implemented in Mesquite (Maddison and Maddison, 2021). Probability values at key steps in the phylogeny were inserted on the chronogram.

## Results and discussion

**Figure 1:**
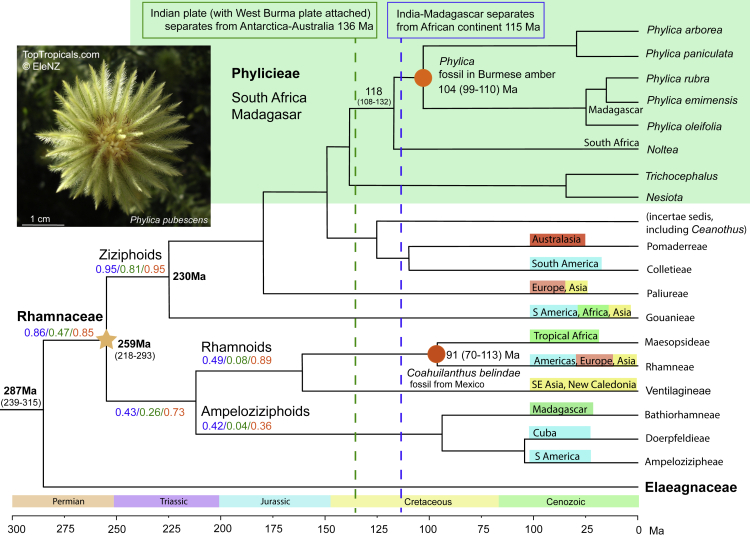
Dated Rhamnaceae phylogeny using the new *Phylica* fossils identified from Burmese amber to set the molecular clock for the Ziziphoid branch, plus a 70.6 My old fossil from Mexico for the Rhamnoid branch The crown age of *Phylica* was set at 104.5 Ma (midpoint of 99–110 My, Shi et al., 2022). Ages are given as the median ± 95% highest posterior density (HPD) interval. The light orange star is located at the crown of Rhamnaceae. For probability values at a given stem, those in blue refer to hard-seededness (impermeable to water), those in green to fire-stimulated germination (FSG, specifically heat-released dormancy), and those in red to fire-proneness (vegetation likely to burn within its lifetime). *Phylica pubescens* photo, © toptropicals.com (with permission).

Our analysis gives the monotypic Cape genus *Noltea* separating from *Phylica* at 118 Ma (108–132 Ma, 95% highest posterior density, HPD) with its common stem arising at 138 Ma (Figure 1). If this lineage arose in the Cape, South Africa (there are >150 extant species), this gives 30 million years (My) in which to reach the West Burma plate via Madagascar (two extant species) then Greater India, before India’s drift north began 88 Ma (Reeves, 2018), that appears quite feasible. Thus, the pathway via Madagascar-India as preferred by Shi et al., 2022, is more plausible than one via an isolated West Burma plate drifting north from 145 Ma (before the lineage arose according to our estimates). Such an outcome also serves to dismiss the hypothesis that this plate had an NW Australian origin and drifted independently from the Indian plate to reach Asia. The likelihood of *Phylica* arising 27 My earlier than our estimates, as required (Young et al., 2019), and traversing the 6,000 km that separated the Burmese plate from East Africa at that time, as required to ensure co-distribution (in either direction, depending on its origin) before West Burma broke away, seems quite remote.

Using a steady rate of DNA change based on the fossil Phylicas to set the molecular clock on the Ziziphoideae side and fossil *Coahuilanthus* on the Rhamnoideae-Ampeloziziphoideae side yielded the crown of Rhamnaceae in the late Permian, at 259 (218–293) Ma (Figure 1). It separated from Elaeagnaceae in the early Permian, 287 (239–315) Ma. Thus, the Rhamnaceae is clearly rooted in Gondwana, supporting Hauenschild et al. (2018), with spreading of its tribal ancestors to Africa, Australasia, the Americas, Europe and Asia before the splitting of Antarctica-Australia from the rest of Gondwana, 136 Ma (Figure 1). Thus, our chronogram can explain how Pomaderreae, an essentially Australasian tribe, can be sister to Colletieae, an essentially South American tribe, as their penultimate common ancestor arose 150 Ma. Similar support exists for Bathiorhamneae in Madagascar as sister to Ampeloziopheae in South America. It also makes sense of the distribution of *Colubrina* (Figure S1) that occurs on all continents except Europe and whose most-recent common ancestor can now be traced to 110 Ma. In addition, it explains how a 71-My-old fossil in Mexico placed at the crown of Rhamneae-Maesopsideae (Hauenschild et al., 2018) can have a central Gondwanan ancestor, possibly in the guise of Rhamneae with a current near-world distribution and whose stem originated 160 Ma, from which Maesopsideae may have split at 90 Ma. Further biogeographic analyses, using areograms, based on these new ages must await a more detailed molecular phylogeny than that we prepared here.

The difference in estimated dates between the most recently produced chronograms and that described here, using these new fossils, is profound. Ignoring the minor error terms in each, previous results are an order of magnitude lower for the origin of both *Phylica* and Phylicieae – the new chronogram adds almost 100 My to their ages, as *Phylica* was previously dated at 12–24 Ma (Onstein and Linder, 2016; Hauenschild et al., 2018). The effect on the estimated age of Rhamnaceae is even more remarkable: it is in the order of 150 My compared with that previously reported, 74–103 Ma (Onstein and Linder, 2016; Hauenschild et al., 2018). Any past statistical concerns about large levels of uncertainty surrounding the means pale into insignificance compared with the effect of the *ad hoc* presence/absence of fossils used to set the molecular clock, as shown here.

Are these new estimates on the evolutionary history of Rhamnaceae outlandish or might they have some credibility? First, having studied their morphology independently (BBL was an acknowledged reviewer of submitted versions of the manuscript), we have full confidence that the fossils indeed belong to *Phylica* [we are less convinced that a new basal genus, *Eophylica*, needed to be recognized, as both are much more similar to each other than they are to the extant sister to *Phylica*, *Noltea*, but this has no effect on interpreting the evolutionary pathways]. Second, modern dating methods of ambers are regarded as highly accurate (methods of Cruickshank and Ko, 2003; Shi et al., 2012; Yu et al., 2019; Xing and Qiu, 2020 used in their study) and we have no hesitation in accepting the age of the fossils.

How do these dates compare with other studies on the origin of flowering plants as a further approach to the level of credibility of our analysis? Cornet, 1989 described 11 ‘Angiosperm-like’ pollen morphotypes at 217–229 Ma in North America, and commented that “failure of palynologists to find distinctively angiospermous pollen types in (the) Triassic (and) Jurassic … has been more a problem of acceptance than a dearth of evidence”, and gave five further supporting references. Hochuli and Feist-Burkhardt, 2013 identified six similar morphotypes at 239–241 Ma in Switzerland. Silvestro et al., 2021 derived ten simulations each representing a family at the older margin of the 95% HPD error term of their best-fit curve to fossil richness at 256 Ma. There is even one record at 266 Ma. This was despite the fact that they omitted all fossil (pollen) records for the Jurassic-Triassic from their work on grounds of possible identification errors; had they been accepted as valid records then these dates may well have extended further into the Permian. The oldest fossils (pollen) that they used belonged to the Platanaceae at an age of 103–113 Ma, similar in age to the *Phylica* fossils but with much less identification certainty. Using similar methods, Li et al., 2019 obtained a mean of 209 Ma for the crown of flowering plants with the older margin of the 95% HPD error term at an age of 267 Ma. For five other papers, the oldest records ranged 244–279 Ma (Kumar et al., 2017). All these ages are comparable with a Rhamnaceae crown at 259 Ma but are somewhat younger than the stem (root) at 287 Ma. Thus, these new dates, based on the Shi et al., 2022 fossils, are quite plausible.

Note that the boundary between the Jurassic and Permian epochs is set at 251 Ma (Ogg et al., 2008) that almost corresponds to the age of the mean crown of the Rhamnaceae. This family separates from Elaeagnaceae at a mean of 287 Ma, corresponding to the early Permian. This great age would appear to have no precedent in past estimates of the origin of flowering plants. Even more surprising is that the Rhamnaceae is not even considered basal among flowering plants, with the Magnoliids, Monocots and early Eudicots regarded as more ancestral. This unexpected early origin of Rhamnaceae, and thus of the entire class of flowering plants, due to the excellent ’detective’ work of Shi et al., 2022, is based on the most reliable fossil evidence produced for any group of plants so far. These ancient *Phylica* fossils provide a compelling case for a radical re-assessment of the origin of flowering plants that must now be seen as not just well into the Triassic but also well into the Permian.

Fire-released seed dormancy, accompanied by arillate seeds that are ant-dispersed/buried, is well-established among the Ziziphoideae (Pausas and Lamont 2022) and our ancestral trait reconstructions show a strongly fire-prone history for this subfamily, with a probability (p) of 0.95 (Figure S1). This provides extra support that the Phylicieae occupied the fire-prone shrub stratum of open vegetation (fynbos), 110−100 Ma, as suggested by Shi et al., 2022 on the basis of charcoal concurrently in amber of similar ages to their fossils. The indications that the Rhamnoideae-Ampeloziziphoideae was also fire-prone is less convincing, at a p = 0.73, but overall our (indirect) evidence for Rhamnaceae arising in fire-prone vegetation 287 Ma is also strong (p = 0.85). The estimates of Belcher et al., 2010 that the probability of ignition of plant matter reached an all-time high of 0.90–1.00 in the 250–340 Ma period is consistent with our findings. A prerequisite for the evolution of fire-type, heat-released seed dormancy are hard (impermeable) seeds (Pausas and Lamont 2022). Such a likelihood is as strong as for fire- proneness in the Rhamnaceae, essentially on the Ziziphoid side (p = 0.95) (Figure 1). This is associated with heat-released seed dormancy for the Ziziphoid ancestor at 259 Ma (p = 0.81) but not for Rhamnoideae-Ampeloziziphoideae (p = 0.26). The fire-prone environment but rarity of seed dormancy in this latter clade implies a monsoon-dominated past (Pausas and Lamont 2022) consistent with the savanna setting of many extant species assessed here (Figure S1). These results now add an important fire-related trait to the growing list of ancient adaptations to fire (He et al., 2016; Lamont 2022).

### Limitations of the study

One possible limitation is that only one fossil (actually the mean of numerous fossils at that time) was used to date the Ziziphoid branch of the phylogeny, and one fossil to date the Rhamnoid branch. Other fossils are 30–60 million years younger so that they are not particularly informative, composed of palynomorphs rather than reproductive structures that are subject to misidentification problems, and the possibility that the age of the surrounding strata may not correspond to that of the embedded fossils. Using a large sample of younger fossils (Hauenschild et al., 2018) would reduce the estimated age of the entire clade and is biased because of the large extrapolation required. Thus, we assumed that the rate of DNA change was constant throughout evolutionary history for each main branch of the Rhamnaceae phylogeny based on the two fossils. The chances of finding older fossils than these in the future seem remote so that this possible limitation will continue.

Regarding trait assignments, not only are several speciose genera omitted from the molecular phylogeny, but, because each genus is only represented by one or two species except *Phylica*, these may not be representative of their genus. In addition, the relevant traits were sometimes poorly documented, especially experimental data on heat-released seed dormancy and imbibitional properties of the seed. It is understandable that succulent-fruited species are not so tested, as it would seem a redundant exercise in a non-fire prone environment, and explains why data were most deficient for the Rhamnoideae-Ampeloziziphoideae (Figure S1).

### Data and materials availability

All data used in this study have been presented in the paper either in the main text or as Supplemental information.

## STAR★Methods

### Key resources table

**Table:** 

REAGENT or RESOURCE	SOURCE	IDENTIFIER
**Software**		

BEAST v1.10.4	Suchard et al., 2018	https://beast.community
Mesquite	Maddison and Maddison, 2021	https://www.mesquiteproject.org

**Sequences for phylogenetic reconstruction**		

*Adolphia californica*	National Center for Biotechnology Information	AJ306539, HQ340160, AJ306540
*Alphitonia excelsa*	National Center for Biotechnology Information	JF270816, AJ390043, HQ325386, AJ390347
*Alphitonia oblata*	National Center for Biotechnology Information	KP299599, JX495724, AJ390039, DQ146607, KP299304, KP299394, HQ427084, JN900366
*Ampelozizyphus amazonicus*	National Center for Biotechnology Information	JQ588888.1, JQ593590.1, JN900296, JN900326, HG963533.1, JN900347
*Barbeya oleoides*	National Center for Biotechnology Information	KJ012651, AJ390028, JN900299, AJ390331, KJ426792.1, JN900348
*Bathiorhamnus cryptophorus*	National Center for Biotechnology Information	AJ390050, AF328833, AJ390353
*Berchemia discolor*	National Center for Biotechnology Information	KC627674.1, AJ390034, AJ390336
*Berchemiella yunnanensis*	National Center for Biotechnology Information	AJ225783, AF328823, KP299395
*Blackallia biloba*	National Center for Biotechnology Information	AJ390054, AF328822, KC633945
*Ceanothus americanus*	National Center for Biotechnology Information	KP299601, KP093710, KP094647, DQ146612, KP299305, DQ146568, KP095854, KT949413
*Ceanothus cordulatus*	National Center for Biotechnology Information	AY911559, EF528504
*Colletia spinosa*	National Center for Biotechnology Information	KP299606, GQ248177, GQ248666, AF328801, KP299311, AF327603, GQ248363
*Colubrina asiatica*	National Center for Biotechnology Information	KP299646, JX517337.1, JX572851, AF328812, KP299355, KP299441, KP299577
*Condalia microphylla*	National Center for Biotechnology Information	KP299647, JX517422, GQ248667, AF328808, KP299356, AF327606, GQ248364
*Crumenaria erecta*	National Center for Biotechnology Information	KP299658, KP110121.1, KP110412.1, KP299530, KP299369, KP299453, KP299581
*Dallachya vitiensis*	National Center for Biotechnology Information	AJ390045, KP299465
*Dirachma socotrana*	National Center for Biotechnology Information	AY911554, EF528501
*Discaria chacaye*	National Center for Biotechnology Information	KU564605, KU564851, AY911568, EF528518, KU564715, JN900385
*Doerpfeldia cubensis*	National Center for Biotechnology Information	AJ390041, DQ146614, AJ390345
*Emmenosperma alphitonioides*	National Center for Biotechnology Information	AJ390056, AY642154
*Frangula alnus*	National Center for Biotechnology Information	KJ012748.1, AJ390029, JN900289, AJ390339, KJ426911.1, JN900356
*Gouania lupuloides*	National Center for Biotechnology Information	KM894562, AJ390027, JN900306, JN900334, JN900354
*Granitites intangendus*	National Center for Biotechnology Information	AJ390030, AY626452, AJ390332, JN900351
*Helinus integrifolius*	National Center for Biotechnology Information	AF130225, AY257533, KP088824.1, AY626441, KP299384, AY626420, KM406247.1, JN900352
*Hovenia dulcis*	National Center for Biotechnology Information	KP093750, AJ225785, AY626453, AJ225792, KP095860, JN900360
*Karwinskia calderonii*	National Center for Biotechnology Information	AJ390046, AY911539, AJ390349
*Krugiodendron ferreum*	National Center for Biotechnology Information	KR734967.1, AJ390033, JN900293, JN900323, JN900358
*Lasiodiscus mildbraedii*	National Center for Biotechnology Information	AJ390064, AF328827, EF528507
*Maesopsis eminii*	National Center for Biotechnology Information	AF049849, AJ390058, AF328828, EF528529
*Nesiota elliptica*	National Center for Biotechnology Information	AJ390059, AY911599, EF528539
*Noltea africana*	National Center for Biotechnology Information	AM235105, KP299545, KP299386, KP299470
*Paliurus ramosissimus*	National Center for Biotechnology Information	AJ390062, AY911578, AJ390362
*Papistylus grandiflorus*	National Center for Biotechnology Information	KM895639, KR083095.1, KR083144.1
*Phylica arborea*	National Center for Biotechnology Information	KP093692, KP094629, KX302783.1, AJ390337, KX346915.2
*Phylica oleifolia*	National Center for Biotechnology Information	KU853085.1, HQ325597, DQ146580, DQ146536, EU075104, JN900371
*Phylica paniculata*	National Center for Biotechnology Information	JF317444.1, JF317425, U17038, KC821765.1, DQ838727
*Phylica rubra*	National Center for Biotechnology Information	JN966666.1, U17039, JN999629.1, GQ245525
*Pleuranthodes hillebrandii*	National Center for Biotechnology Information	JF954049, JF941944, KF620597.1, HM769680
*Polianthion wichurae*	National Center for Biotechnology Information	AJ306539, HQ340160, AJ306540
*Pomaderris angustifolia*	National Center for Biotechnology Information	JF270816, AJ390043, HQ325386, AJ390347
*Reissekia smilacina*	National Center for Biotechnology Information	KP299599, JX495724, AJ390039, DQ146607, KP299304, KP299394, HQ427084, JN900366
*Retanilla trinervia*	National Center for Biotechnology Information	JQ588888.1, JQ593590.1, JN900296, JN900326, HG963533.1, JN900347
*Reynosia uncinata*	National Center for Biotechnology Information	KJ012651, AJ390028, JN900299, AJ390331, KJ426792.1, JN900348
*Rhamnella franguloides*	National Center for Biotechnology Information	AJ390050, AF328833, AJ390353
*Rhamnidium elaeocarpum*	National Center for Biotechnology Information	KC627674.1, AJ390034, AJ390336
*Rhamnus davurica*	National Center for Biotechnology Information	AJ225783, AF328823, KP299395
*Sageretia thea*	National Center for Biotechnology Information	AJ390054, AF328822, KC633945
*Schistocarpaea johnsonii*	National Center for Biotechnology Information	KP299601, KP093710, KP094647, DQ146612, KP299305, DQ146568, KP095854, KT949413
*Scutia buxifolia*	National Center for Biotechnology Information	AY911559, EF528504
*Siegfriedia darwinioides*	National Center for Biotechnology Information	KP299606, GQ248177, GQ248666, AF328801, KP299311, AF327603, GQ248363
*Spyridium globulosum*	National Center for Biotechnology Information	KP299646, JX517337.1, JX572851, AF328812, KP299355, KP299441, KP299577
*Stenanthemum complicatum*	National Center for Biotechnology Information	KP299647, JX517422, GQ248667, AF328808, KP299356, AF327606, GQ248364
*Trichocephalus stipularis*	National Center for Biotechnology Information	KP299658, KP110121.1, KP110412.1, KP299530, KP299369, KP299453, KP299581
*Trymalium floribundum*	National Center for Biotechnology Information	AJ390045, KP299465
*Ventilago ecorollata*	National Center for Biotechnology Information	AY911554, EF528501
*Ventilago leiocarpa*	National Center for Biotechnology Information	KU564605, KU564851, AY911568, EF528518, KU564715, JN900385
*Ziziphus calophylla*	National Center for Biotechnology Information	AJ390041, DQ146614, AJ390345
*Elaeagnus angustifolia*	National Center for Biotechnology Information	AJ390056, AY642154
*Shepherdia canadensis*	National Center for Biotechnology Information	KJ012748.1, AJ390029, JN900289, AJ390339, KJ426911.1, JN900356
*Hippophae neurocarpa*	National Center for Biotechnology Information	KM894562, AJ390027, JN900306, JN900334, JN900354

**Other**		

Fossil for molecular clock calibration (*Phylica*)	Shi et al. 2022	https://doi.org/10.1038/s41477-021-01091-w
Fossil for molecular clock calibration (*Coahuilanthus belinda)*	Calvillo-Canadell and Cevallos-Ferriz., 2007	https://doi:10.3732/ajb.94.10.1658
Fire-related trait data	This paper	Figure S1

### Resource availability

#### Lead contact

Information and requests for resources should be directed to and will be fulfilled by Tianhua He (tianhua.he@.murdoch.edu.au).

#### Materials availability

This study did not generate new unique reagents and biological materials.

### Experimental model and subject details

No experimental model was involved as a part of this study.

### Method details

#### Dated phylogeny

Available DNA sequences at seven loci for one ore two species of each genus recognised in the Rhamnaceae (except for *Phylica* that includes five species in the analysis), and three species from Elaeagnaceae sl as the outgroup, were obtained from the NCBI (https://www.ncbi.nlm.nih.gov). Phylogenetic reconstruction and dating were carried out with BEAST v1.10.4 (Suchard et al., 2018) using an uncorrelated relaxed molecular clock with two calibration points. The crown age of *Phylica* was set at a conservative mean of 104.5 Ma (midpoint of 99–110 Ma, Shi et al., 2022). The common crown of Rhamneae and Maesopsideae was set at a mean of 70.6 Ma using the compressed-flower fossils of *Coahuilanthus belindae* (Calvillo-Canadell & Cevallos-Ferriz, 2007 following Hauenschild et al., 2018). We used a normal function as priors at both calibration points, as priors with a normal distribution (with 10% of the mean as standard deviation) allow the model to traverse a broader time range to better accommodate uncertainties in both the fossil age and divergence time between key taxon groups. GTR substitution and Yule process evolution models were used. Twenty million iterations over five runs were implemented. For ease of interpretation, species were collapsed to tribes for display. Ages are presented as the median 95% highest posterior density interval. The maximum clade credibility tree containing all taxa (Figure S1) and the aligned DNA matrix are given in the online Supplemental Information.

#### Ancient traits reconstruction

Fire-related traits (presence/absence) for each species in the molecular phylogeny were collated from data in Pausas and Lamont (2022) and numerous websites (identity of these additional sources available on request). Hard-seededness was gauged as impermeable to water or evidence of a sclerified seed coat (these were sometimes referred to the endocarp and not the testa), heat-released dormancy is given as fire-stimulated germination in the results and required seeds treated with fire-type heat to have exceeded germination of the controls, and fire-proneness refers to occurrence of the species in vegetation likely to burn within its lifetime. Where there were conflicting reports or there was doubt [e.g., occurs on the edge of rainforests under a monsoon (i.e., fire-prone) climate] it was assigned to both trait states. If data were lacking for a given species but there was knowledge for other species in the genus, they were assigned these trait states on the assumption that they were diagnostic for the genus. Ancient trait reconstruction was conducted following a maximum likelihood probability model and implemented in Mesquite (Maddison and Maddison, 2021). Probability values at key steps in the phylogeny were inserted into the chronogram.

### Quantification and statistical analysis

No specific quantification and statical analysis were involved as a part of this study.

## Data Availability

• Accession numbers of DNA sequences used for phylogenetic reconstruction are listed in the Key resources table • No original code was used to generate results in this study. • Any additional information required to reanalyze the data reported in this paper is available from the lead contact upon request.

## Supplemental information

Document S1. Figure S1and Data S1and Data S1
