# Supplementary material for: Ancient Rhamnaceae flowers impute an origin for flowering plants exceeding 250-million-years ago
Source: iScience. 2022 Jun 18;25(7):104642. doi: 10.1016/j.isci.2022.104642 (PMC9254029; doi:10.1016/j.isci.2022.104642)
Supplement: Document S1. Figure S1and Data S1and Data S1 [file mmc1.pdf]

iScience, Volume *25*

Supplemental information

Ancient Rhamnaceae flowers impute an origin for flowering plants exceeding 250-million-years ago

Tianhua He and Byron B. Lamont

Figure S1 (related to Fig. 1). Ancestral trait reconstruction for three fire-related traits added to the Rhamnaceae-Elaeagnaceae phylogeny. Probability values of trait existing at that time in blue refer to hard-seededness (impermeable to water), values in green to fire-stimulated germination (FSG, specifically heat-released dormancy), and values in red to fire-proneness (vegetation likely to burn within its lifetime). Filled squares: trait present; empty square: trait absent; half-filled squares: trait present or absent in different locations. Units of X-axis: million years ago. These probability values have been transferred to Fig. 1.

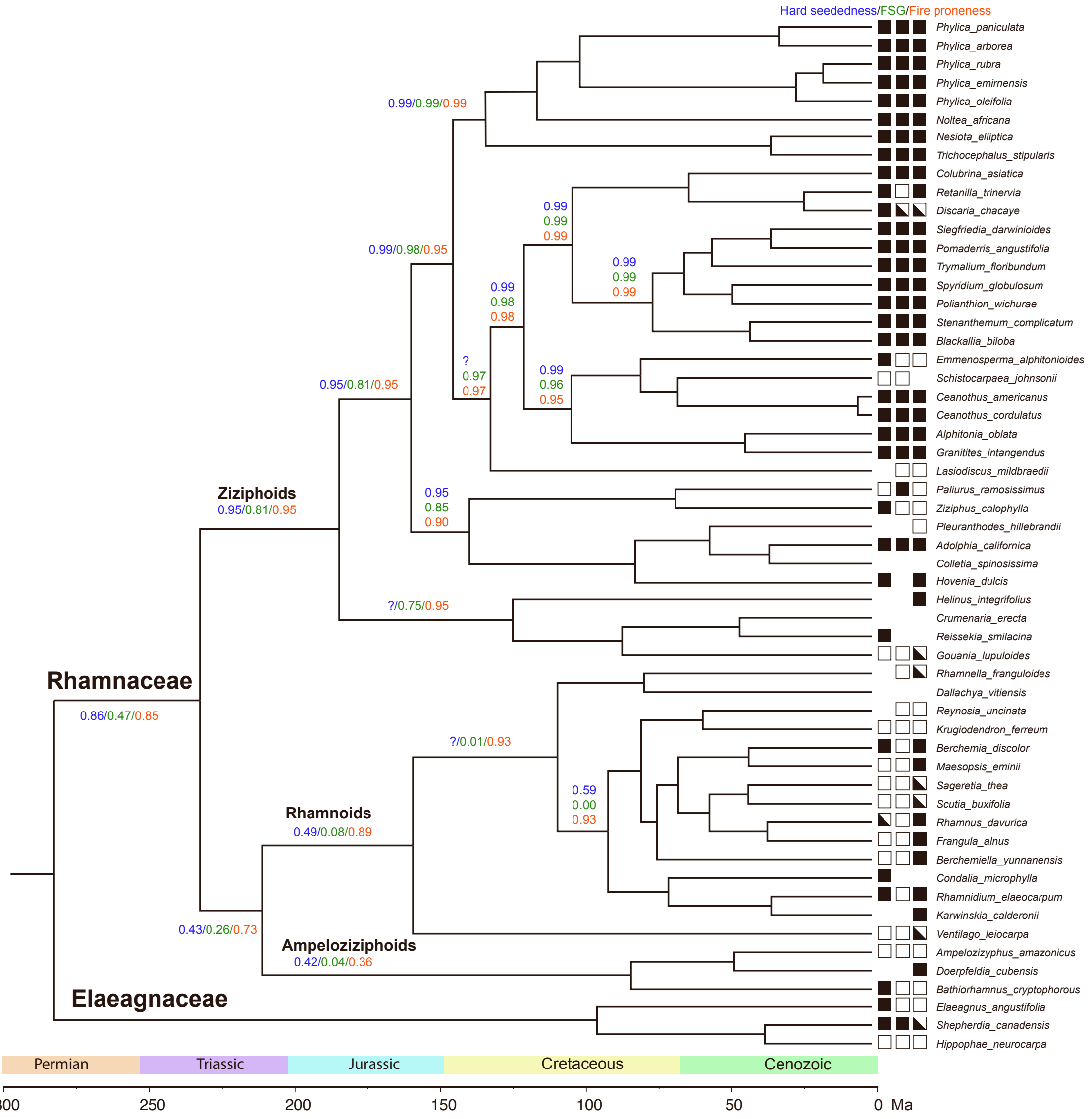

Supplemental data 1: DNA alignment matrix for dated phylogeny re-construction, related to STAR methods

>Ceanothus_americanus
CCCCTTTTCTCCGGTTCAAATCGAATGTCAAATGGAAGAGTTTCAAGGAT
ATTTCGAACTAAATAGATCTCGGCAACACGATCTCCTATACCCACTTATC
TTTCGGGAGTATATTTATGCATTTGCTCATGATCATGGTTTAAATAGATC
GAGTTTGCTGGAAAATGTAGGTTATGACAATAAATCTAGTTTACTAATTG
TAAAACGTTTAATTACTCGAATGTATCACCAGAATCATTTGATTATTTCC
GCTAATGATTCTGACCAAAATAAATGTTTTGGGTACAACAATAATTTGTA
TTCTCAAATGATATCAGAGGGCTTTGCAGTCATTGTGGAAATTCCATTTT
CCTTACGATTCCTACAATTAGTATCTTCGAGGCCAGAAATCGTAAAATAT
TCTAATTTACGATCAATTCATTCAATATTTCCATTTTTAGAGGACAAATT
CCCACATTTAAATTATGTATCAGATATACGAATACCCTACCCCATTCATC
TGGAAATCTTGATTCAAACCCTTCGCTATTGGGTGAAAGATGCCTCTTCT
TTGCATTTATTACGGCTTTTTCTTCACGAGTATTATAATTGGAATAGTTT
TAT--------------------------TACTCCAAAAAAATCTATTT
CTTTTTTTTTGAAAAGTAATTCAAGATTTTTCTTGTTCCTATATAATTCT
CATGTTTGTGAATACGAATCCATCTTACT----TTTTCTCCGTAACCAAT
CTTCTCATTTACGATTAACATCTTCTGGGGTATTTTTTGAGCGAATTTAT
TTCTATGGAAAAATAAAAGATCCTGTAGAAGAAGTCTTTTCTAATGATTT
TCCGGCGATCTTATGGTTCTTCACGGAGCCTTTCATGCATTATGTTAGAT
ATCAAGGAAAATCTATTTTGGTTTCAAAAGATACGCCTCTTCTAATGAAT
AAATGGAAATATTATCTTGTCCTTTTATGGCAAGGTCATTTTTATGTGTG
GGCTCAACCAGGAAGGATCTATATAAACCAATTAGGCAACCATTCCGTCG
GCTTTTTGGGCTATCTTTCAAGTGTGCGACTAAATCTTTCAGTGGTACGG
AGTCAAATGCTCGAAAATTCTTTTATAATGGATAATGCTATAAAGAAGCT
TGATACATTAGTTCCAATTAGTCCAATG----------------------

-------------------------------ATGGATCATACCTTTCC
TTCCCCTTCCAACTCCTATGTTAATAGGAGTAGGACTTCTCCTTTTTCCG
ACGGCAATAAAAAATCTTCGCCGTATGTGGGTTTTTCCTAGTGTTTTACT
GTTAAGTATAGTTATGGTTTTTTCAGCCCATATATTTATTCAACAAATAA
ATAACAGTTCTATCTATCTATCTGTTTGGTCTTGGACCATCAATAATGAT
TTTTCTTTAGAATTCGGCTACTTAATTGATCCACTTACTTCTCTTATGTT
AATATTAATCACTACTGTTGGAATTATGGTTCTTATTTATAGTGATAATT
ATATGTCTCATGATCAGGGATATTTGAGATTTTTTGCTTATATGAGTTTT
TCTAATACTTCAATGTTAGGATTAGTTACTAGTTCAAATTTGATACAAAT
TTATTTTTTTTGGGAATTAGTTGGAATGTGTTCTTATCTATTAATAGGTT
TTTGGTTCACCCGACCTATTGCGGCGAATGCTTGTCAAAAGGCGTTTGTA
ACTAATCGTGTAGGGGATTTTGGGTTATTATTAGGAATTTTAGGTTTTTA
TTGGATAACGGGTAGTTTAGAATTTAGGGATTTGTTTGAAATAGTAAATA
ACGTGGTTGATAATAATGAAGTTGATTTTTTATTTGTTACTTTGTGTGCC
TGTCTATTATTTGCCGGTGCAGTTGCTAAATCTGCCCAATTTCCCCTTCA
TGTATGGTTACCCGATGCTATGGAAGGACCTACCCCTATTTCGGCTCTTA
TACATGCTGCTACTATGGTAGCAGCCGGAATTTTTCTTGTAGCTCGGCTT
CTTCCGCTTTTTATAGTTATACCTTTCATAATGAATCTAATAGCTTTTAT
TGGGATAATAACATTATTTTTAGGAGCCACTTTAGCTCTTGCTCAAAAAG
ATATTAAGAAGGGTTTAGCTTATTCTACAATGTCTCAATTGGGTTATATG
ATGTTGGCTCTAGGTATGGGGTCTTATCGAGCTGCTTTATTTCATTTGAT
TACTCATGCTTATTCGAAAGCATTGTTGTTTTTAGGATCCGGATCCATTA
TTCATTCCATGGAAACAATTGTTGGATATTCTCCAGATAAAAGCCAGAAT
ATGGTTCTTATGGGCGGTTTAAGAAAACATGTACCAATTACAAAAACCGC
GTTTTTATTAGGTACACTTTCTCTGTGTGGCATTCCACCCCTTGCCTGTT

TTTGGTCCAAAGATGAAATTCTTAGTGATAGTTGGTTATATTCACCGATT
TTTGCAATAATAGCTTGTTCCACAGCTGGATTAACTGCATTTTATATGTT
TCGGATCTATTTACTTACTTTTGAAGGACATTTAAACGTTCAGTTTCAAA
TTTACAGTGGAAAAAAAAAATAGCTCGTTCTATTCAATATCTCTATGGGGT
AAAAAAGGGCAAAAGGTTATTAAAACAAATTTTCGTTTATTAACTTTATT
AAAAATGAATAATACTGAAAGGACTTCTTTTTTTTCTAAAAAGGCATATC
GAATTGATAGTAATCTAAGAAGCATG---------ATAAGCCCTTTTATT
ACTATTAGTCATTTTGGAACTAAAAAAACTTTCTCATATCCGCAGGAATC
GGACAATACTATGCTATTTCCTATGCTTGTATTAGTACTATTTACTTTGT
TCATTGGATTCATAGGAATTCCTTTCTTCAATCAATTCAATCAAGAAGCG
ATGGATTTGGATATATTATCAAAATTGTTAAACCCGGCTATAAACCTTTT
ACATCCAAATCCAAATACTTCTGTGGATTTGGATGAATTTATTTCAAACG
CAACTTTTTCAGTCAGTATAGCTTTTTTTGGAATCCTTATAGCGGCCTTT
TTATATAAGCCGGTTTATTCATCTTTACAAAATTTGAATTTCTTTAATTC
ATTTGTTA---------AAAGTATTCCTAAAAAAAGAAAAATTCTTTTGG
AAAAACTACTAAATGGGATATATGATTGGTCATATAATCGTGGTTATATA
GATGTTTTTTATGCAATATCCTTAACTAAAGGTATAAGAGGATTGGCTCA
ACTAACTCATTTTTTTGATAAACGAATAATTGATGGAATTACGAATGGTG
TCGGTATTGCGAGTTTTTTCGTG---------------------------

-------------------------TTACACTCCTGACTATGAAACAAA
AGATACTGATATCTTGGCAGCGTTTCGAGTAACTCCTCAACCTGGAGTTC
CGCCTGAGGAAGCAGGGGCCGCGGTAGCTGCTGAATCTTCTACTGGTACA
TGGACAACTGTATGGACTGACGGGCTTACCAGTCTTGATCGTTACAAAGG
TCGATGCTACCACATCGAGCCCGTTGCTGGAGAAGAAACTCAATTTATTG
CTTATGTAGCTTACCCCTTAGACCTTTTTGAAGAAGGTTCTGTTACTAAC
ATGTTTACTTCCATTGTGGGTAATGTTTTTGGGTTCAAGGCCCTGCGCGC

TCTACGTCTGGAGGATTTGCGAATCCCTCCTGCTTATTCTAAAACTTTCC
AAGGACCGCCTCACGGCATCCAAGTTGAAAGAGATAAATTGAACAAGTAT
GGCCGCCCCCTATTGGGATGTACTATTAAACCTAAACTGGGGTTATCCGC
TAAGAATTACGGTAGAGCAGTTTATGAATGTCTTCGCGGTGGACTTGATT
TTACCAAAGATGATGAGAACGTGAATTCCCAACCATTTATGCGTTGGAGA
GACCGTTTCTTATTTTGTGCCGAAGCCCTTTATAAAGCACAGACTGAAAC
AGGTGAAATCAAAGGGCATTACTTGAATGCTACTGCAGGTACATGCGAAG
AAATGATTAAAAGGGCTGTATTTGCCAGAGAATTGGGAGTTCCTATTGTA
ATGCATGATTACTTAACAGGGGGATTCACTGCAAATACTAGCTTGGCTCA
TTATTGCCGAGATAATGGTCTACTTCTTCACATCCACCGTGCAATGCATG
CAGTTATTGATAGACAGAAAAATCATGGTATACACTTTCGTGTACTAGCT
AAAGCGTTACGTATGTCTGGTGGAGATCATATTCACTCTGGTACTGTAGT
AGGTAAACTTGAAGGTGAAAGAGAAATAACTTTAGGCTTTGTTGATTTAC
TACGTGATGATTTTATTGACAAAGATCGAAGCCGTGGTATTTATTTCACT
CAAGATTGGGTCTCTCTACCAGGTGTTCTGCCTGTGGCTTCAGGGGGTAT
TCATGTTTGGCATATGCCTGCTCTGACCGAGATCTTTGGAGATGATTCCG
TACTACAGTTTGGCGGAGGAACTTTAGGACACCCTTGGGGAAATGCACCC
GGTGCCGTAGCTAATCGAGTAGCTCTAGAAGCATGTGTACAAGCTCGTAA
TGAGGGACGTGATCTTGCTCGTGAGGGTAATGAAATTATCCGTGAGGCTA
GTAAATGGAGTCCTGAACTAGCTGCTGCTTGTGAAGTATGG---------

--AAATTCAGAGAAACCCTGGAATTACAAATGGGCAATCCTGAGCCAAAT
CCTCTTTTCTGAAAACAAACAAGGATTCA-------GAA---A---GTGA
TAATAAAAAAGGATAGGTGCAGAGACTCAATGGAAGCTGTTCTAACAAAT
GG----AGTTGGCTGCGATGCGTT------AGTAAAGGAAT-CCTTCCAT
CGAAACTCCAGAAAGGATGAAGAATAAACCTATATATACGTATACGTACT
GAAATACTATCTCCAAACCAAATGATTAATTA-----------------
---------------------TGTTTATATGAAA-------------A
ATGAAAGAATTGTTGTGAA-----TCGATTCCAAGTAAAAA-AAAAAAAA
AAATGGAATATTCATTGATCAAATCATTTACTCCATCGTAATCTGAT---
-AGATCTTTTGAAAAATTGAT------TAATCGGACGAGAATAAAGATAG
AGTCCCATTCTACATGTCAA------------TATCGACAACAATGAAAT
TTATAGTAAGAGGAAAATCCGTCGACTTTA-GAAATCGTGAGGGTTCAAG
TCCCTCTATCCCCAAAAAGGC-CCATTTGATTCCCTAATTTTTTATC---
--CTATACT-C----TCATTTCGTTAGCGGTTCAAAATTCGTTATGTTTC
TCATTC------ATTAATT-TTTT-------------------------
------TCT--TTTCACAAGCCTTGT----GATATATATGATACACG---
----TAC--------AAATGAACATCA--TTGAGCAAGT--AACCCCGAT
TGTAAATTGG-------AATGATC-ATATT--------ATCGCTCGTACT
GTACTGAAA----CTTACAAAGTCTTCTTT----T-TGAA--GATCTAAG
AAATT----CCACCAAGGCCTGGTTAAGACTTTGTAAT----CCCCTTTT
CGTCTTTTTAATTGACATAGACCCAAGTCATCTATTAA------AATGAG
GATGATGCGTCGTG-----AATG--------------------------

--------TCGAAA-CCT-GCCTAGCAGAACGACCCGTGAACCTGTA--A
AAACATACCGG-GGGCTCGAG---GCCTTTAGGC--CTC-GGACTCTCT-
TGGTCGGTGGGCTGTA---TCCTGTGCCTTG--CTTGTCGATGCACGGGT
GCTG--CTTTTTCGGCCGC-ACAAACGAACCCCGGCGCAAAACCG-CGCC
-AAGGATCATCTAACGAATTGGCAC-TGCCCTGTCGCCCCAGAGATGGTG
TGCGGTT-GGGTGTGCGT--CGTATTC---TATATGTCAAAACGACTCTC
GGCAACGGATATCTCGGCTCTCGCATCGATGAAGAACGTAGCGAAATGCG
ATACTTGGTGTGAATTGCAGAATCCCGTGAACCATCGAGTCTTTGAACGC
AAGTTGCGCCCGAAGCCA--TTAGGCCGAGGGCACGTCTGCCTGGGCGTC
ACACAATGTTGCCCCCCCCAACCTCGAAGGTGA---------GA-------
-------------------GGGCGGATGCTGGCCTCCCGT-GTTTTTGT
TTTG--CGGTTGGCCGAAAT--GCGAGTCCTCGGCGACGAACGCCGCAAC
AATCGGTGGTTCTCCAACCCTCG-GTGCCCTGTTGTGTGCATGGATTGCC

GTTGTGGCACGATAGACC---CCAA-TGCGCT---GCATGCAGCGCTC-C
AA--

>Ceanothus_cordulatus
CCCCTTTTCTCCGGTTCAAATCGAATTTCAAATGGAAGAGTTTCAAGGAT
ATTTCGAACTAAATAGATCTCGGCAACACGATCTCCTATACCCACTTATC
TTTCGGGAGTATATTTATGCATTTGCTCATGATCATGGTTTAAATAGATC
GAGTTTGCTGGAAAATGTAGGTTATGACAATAAATCTAGTTTACTAATTG
TAAAACGTTTAATTACTCGAATGTATCACCAGAATCATTTGATTATTTCC
GCTAATGATTCTGACCAAAATAAATGTTTTGGGTACAACAAGAATTTGTA
TTCTCAAATGATATCAGAGGGCTTTGCAGTCATTGTGGAAATTCCATTTT
CCTTACGATTCCTACGATTAGTATCTTCGAGGCCAGAAATCGTAAAATAT
TCTAATTTACGATCAATTCATTCAATATTTCCATTTTTAGAGGACAAATT
CCCACATTTAAATTATGTATCAGATATACGAATACCCTACCCCATTCATC
TGGAAATCTTGATTCAAACCCTTCGCTATTGGGTGAAAGATGCCTCTTCT
TTGCATTTATTACGGCTTTTTCTTCACGAGTATTATAATTGGAATAGTTT
TAT---------------------------TACTCCAAAAAAATCTATTT
CTTTTTTTTTGAAAAGTAATTCAAGATTTTTCTTGTTCCTATATAATTCT
CATGTTTGTGAATACGAATCCATCTTACT----TTTTCTCCGTAACCAAT
CTTCTCATTTACGATTAACATCTTCTGGGGTATTTTTTGAGCGAATTTAT
TTCTATGGAAAAATAAAAGATCCTGTAGAAGAAGTCTTTTCTAATGATTT
TCCGGCGATCTTATGGTTCTTCACGGAGCCTTTCATGCATTATGTTAGAT
ATCAAGGAAAATCTATTTTGGTTTCAAAAGATACGCCTCTTCTAATGAAT
AAATGGAAATATTATCTTGTCCTTTTATGGCAAGGTCATTTTTATGTGTG
GGCTCAACCAGGAAGGATCTATATAAACCAATTAGGCAACCATTCCGTCG
GCTTTTTGGGCTATCTTTCAAGTGTGCGACTAAATCTTTCAGTGGTACGG
AGTCAAATGCTCGAAAATTCTTTTATAATGGATAATGCTATAAAGAAGCT
TGATACATTAGTTCCAATTAGTCCAATG----------------------

---------------------------------ATGGATCATACCTTTCA
TTCCCCTTCCAACCCCTATGTTAATAGGAGTAGGACTTCTCCTTTTTCCG
ACGGCAATAAAAAATCTTCGCCGTATGTGGGTTTTTCCTAGTGTTTTACT
GTTAAGTATAGTTATGGTTTTTTCAGCCCATATATTTATTCAACAAATAA
ATAACAGTTCTATCTATCTATCTGTATGGTCTTGGACCATCAATAATGAT
TTTTCTTTAGAATTCGGCTACTTAATTGATCCACTTACTTCTCTTATGTT
AATATTAATCACTACTGTTGGAATTATGGTTCTTATTTATAGTGATAATT
ATATGTCTCACGATCAGGGATATTTGAGATTTTTTGCTTATATGAGTTTT
TCTAATACTTCAATGTTAGGATTAGTTACTAGTTCAAATTTGATACAAAT
TTATTTTTTTTGGGAATTAGTTGGAATGTGTTCTTATCTATTAATAGGTT
TTTGGTTCACCCGACCTATTGCGGCGAATGCTTGTCAAAAGGCGTTTGTA
ACTAATCGTGTAGGGGATTTTGGGTTATTATTAGGAATTTTAGGTTTTTA
TTGGATAACGGGTAGTTTAGAATTTAGGGATTTGTTTGAAATAGTAAATA
ACGTGGTTGATAATAATGAAGTTGATTTTTTATTTGTTACTTTGTGTGCC
TGTCTATTATTTGCCGGTGCAGTTGCTAAATCTGCCCAATTTCCCCTTCA
TGTATGGTTACCCGATGCTATGGAAGGACCTACCCCTATTTCGGCTCTTA
TACATGCTGCTACTATGGTAGCAGCCGGAATTTTTCTTGTAGCTCGGCTT
CTTCCGCTTTTTATAGTTATACCTTTCATAATGAATCTAATAGCTTTTAT
TGGGATAATAACATTATTTTTAGGAGCCACTTTAGCTCTTGCTCAAAAAG
ATATTAAGAGGGGTTTAGCTTATTCTACAATGTCTCAATTGGGTTATATG
ATGTTGGCTCTAGGTATGGGGTCTTATCGAGCTGCTTTATTTCATTTGAT
TACTCACGCTTATTCGAAAGCATTGTTGTTTTTAGGATCCGGATCCATTA
TTCATTCCATGGAAACAATTGTTGGATATTCTCCAGCTAAAAGCCAGAAT

ATGGTTCTTATGGGCGGTTTAAGAAAACATGTACCAATTACAAAAACCGC
GTTTTTATTAGGTACACTTTCTCTGTGTGGCATTCCACCCCTTGCCTGTT
TTTGGTCCAAAGATGAAATTCTTAGTGATAGTTGGTTATATTCACCGATT
TTTGCAATAATAGCTTGTTCCACAGCTGGATTAACTGCATTTTATATGTT
TCGGATCTATTTACTTACTTTTGAAGGACATTTAAACGTTCAGTTTCAAA
TTTACAGTGGAAAAAAAAAATAGCTCGTTCTATTCAATATCTCTATGGGGT
AAAAAAGGGCAAAAGGTTATTAAAACAAATTTTCGTTTATTAACTTTATT
AAAAATGAATAATAATGAAAGGACTTCTTTTTTTTCTAAAAAGGCATATC
GAATTGATAGTAATCTAAGAAGCATG---------ATAAGCCCTTTTATT
ACTATTAGTCATTTTGGAACTAAAAACACTTTCTCATATCCGCAGGAATC
GGACAATACTATGCTATTTCCTATGCTTGTATTAGTACTATTTACTTTGT
TCATTGGATTCATAGGAATTCCTTTCTTCAATCAATTCAATCAAGAAACG
ATGGATTTGGATATATTATCAAAATTGTTAAACCCGGCTATAAACCTTTT
ACATCCAAATCCAAATACTTCTGTGGATTGGGATGAATTTATTTCAAACG
CAACTTTTTCAGTCAGTATAGCTTTTTTTGGAATCCTTATAGCGGCCTTT
TTATATAAGCCGGTTTATTCATCTTTACAAAATTTGAATTTCTTTAATTC
ATTTGTTA---------AAAGTATTCCTAAAAAAAGAAAAATTCTTTTGG
AAAAACTACTAAATGGGATATATGATTGGTCATATAATCGTGGTTATATA
GATGTTTTTTATGCAATATCCTTAACTAAAGGTATAAGAGGATTGGCTCA
ACTAACTCATTTTTTTGATAAACGAATAATTGATGGAATTACGAATGGTG
TCGGTATTGCGAGTTTTTTCGTG--------------------------------

--GTTGGATTCAAAGCC
GGTGTTAAAGATTATAAATTGACTTATTACACTCCTGACTATGAAACAAA
AGATACTGATATCTTGGCAGCGTTTCGAGTAACTCCTCAACCTGGAGTTC
CGCCTGAGGAAGCAGGGGCCGCGGTAGCTGCTGAATCTTCTACTGGTACA
TGGACAACTGTATGGACTGACGGGCTTACCAGTCTTGATCGTTACAAAGG
TCGATGCTACCACATCGAGCCCGTTGCTGGAGAAGAAACTCAATTTATTG

CTTATGTAGCTTACCCCTTAGACCTTTTTGAAGAAGGTTCTGTTACTAAC
ATGTTTACTTCCATTGTGGGTAATGTTTTTGGGTTCAAGGCCCTGCGCGC
TCTACGTCTGGAGGATTTGCGAATCCCTCCTGCTTATTCTAAAACTTTCC
AAGGACCGCCTCACGGCATCCAAGTTGAAAGAGATAAATTGAACAAGTAT
GGCCGCCCCCTATTGGGATGTACTATTAAACCTAAACTGGGGTTATCCGC
TAAGAATTACGGTAGAGCAGTTTATGAATGTCTTCGCGGTGGACTTGATT
TTACCAAAGATGATGAGAACGTGAATTCCCAACCATTTATGCGTTGGAGA
GACCGTTTCTTATTTTGTGCCGAAGCCCTTTATAAAGCACAGGCTGAAAC
AGGTGAAATCAAAGGGCATTACTTGAATGCTACTGCAGGTACATGCGAAG
AAATGATTAAAAGGGCTGTATTTGCCAGAGAATTGGGAGTTCCTATTGTA
ATGCATGATTACTTAACAGGGGGATTCACTGCAAATACTAGCTTGGCTCA
TTATTGCCGAGATAATGGTCTACTTCTTCACATCCACCGTGCAATGCATG
CAGTTATTGATAGACAGAAAAATCATGGTATACACTTTCGTGTACTAGCT
AAAGCGTTACGTATGTCTGGTGGAGATCATATTCACTCTGGTACTGTAGT
AGGTAAACTTGAAGGTGAAAGAGAAATA----------------------

-----AATTGGATTGAGCCTTG-GTATGGAAACCTACCAAGTGATAACTT
TCAAATTCAGAGAAACCCTGGAATTACAAATGGGCAATCCTGAGCCAAAT
CCTCTTTTCTGAAAACAAACAAGGATTCA-------GAA---A---GTGA
TAATAAAAAAGGATAGGTGCAGAGACTCAATGGAAGCTGTTCTAACAAAT
GG----AGTTGGCTGCGATGCGTT------AGTAAAGGAAT-CCTTCCAT
CGAAACTCCAGAAAGGATGAAGAATAAACCTATATATACGTATACGTACT
GAAATACTATCTCCAAACCAAATGATTAATTA------------------
----------------------TGTTTATATGAAA--------------A
ATGAAAGAATTGTTGTGAA-----TCGATTCCAAGTAAAAA-AAA-AAAA
AAATGGAATATTCATTGATCAAATCATTTACTCCATCGTAATCTGAT---
-AGATCTTTTGAAAAATTGAT------TAATCGGACGAGAATAAAGATAG
AGTCCCATTCTACATGTCAA------------TATCGACAACAATGAAAT
TTATAGTAAGAGGAAAATCCGTCGACTTTA-GAAATCGTGAGGGTTCAAG
TCCCTCTATCCCCAAAAAGGC-CCATTTGATTCCCTAATTTTTTATC---
--CTATACT-C----TCATTTCGTTAGCGGTTCAAAATTCGTTATGTTTC
TCATTC------ATTAATT-TTTT--------------------------
------TCT--TTTCACAAGCCTTGT----GATATATATGATACACG---
----TAC--------AAATGAACATCA--TTGAGCAAGT--AACCCCGAT
TGTAAATTGG-------AATGATC-ATATT--------ATCGCTCGTACT
GTACTGAAA----CTTACAAAGTCTTCTTT----T-TGAA--GATCTAAG
AAATT----CCACCAAGGCCTGGATAAGACTTTGTAAT----CCCCTTTT
CGTCTTTTTAATTGACATAGACCCAAGTCATCTATTAA------AATGAG
GATGATGCGTCGTG-----AATG---------------------------

--------TCGAAA-CCT-GCCTAGCAGAACGACCCGTGAACCTGTA--A
AAACATACCGGGGGGCTCGGG---GCCTTTAGGC--CTC-GGACTCTCT-
TGGTCGGTGGGCTGTA---TCCTGTGCCTTG--CTTGTCGATGCACGGGT
GCTG--CTTTTTCGGCCGC-ACAAACGAACCCCGGCGCAAAATCG-CGCC
-AAGGATCATCTAACGAATTGGCAC-TGCCCTGTCGCCCCAGAGATGGTG
TGCGGTT-GGGTGTGCGT--CGTATTC---TATATGTCAAAACGACTCTC
GGCAACGGATATCTCGGCTCTCGCATCGATGAAGAACGTAGCGAAATGCG
ATACTTGGTGTGAATTGCAGAATCCCGTGAACCATCGAGTCTTTGAACGC
AAGTTGCGCCCGAAGCCA--TTAGGCCGAGGGCACGTCTGCCTGGGCGTC
ACACAATGTTGCCCCCCCAACCTCGAAGGTGN---------GA-------
--------------------GGGCGGATGCTGGCCTCCCGT-GTTTTTGT

TTTG--CGGTTGGCCGAAAT--GCGAGTCCTCGGCGACGAATGCCGCAAC
AATCGGTGGTTCTCCAACCCTCG-GTGCCCTGTTGTGTGCATGAATTGCC
GTTGTGGCACGATAGACC---CCAA-TGCGCT---GCT------------

>Adolphia_californica
---------CCCCGTTCAAGTCGAATTTAAAATGGAGGAATTTCAAAGAT
ATTTCGAACTCGATAGATCTCAGCAGCATGACCTCCTATACCCACTTATC
TTTCGGGAGTATATTTATGCATTTGCTCATGATCATGGCTTAAATAGATC
AGATTTGTTGGAAAATGTAGGTTATGACAATAAATCTAGTTTACGAATTG
TAAAACGTTTCATTACTCGAATGTATCACCAGAATCATTTGATTATTTCC
GCTAACTATTCTACCCAAAATAAGTGTTTTGGGTACAACAAGAATTTGTA
TTCTCAAATGATATCAGAGGGATTTGCAGTCATTGTGGAAATTCCATTTT
CCTTACGATTACCACCNNNNNNNNNTTCGGGGACAGAAATTGTAAAATAT
TCTAATTTACGATCAATTCATTCAATATTTCCATTTTTAGAGGACAAATT
CCCACATTTAAATTATGGATCAGATATACGAATACCCTACCCCATTCATC
TGGAAATCTTGGTTCAAATTCTTCGCTATTGGGTGAAAGATGCCTCTTCT
TTGCATTTATTACGGCTTTTTCTTCACGAGTATTCTAATTGGAATAGTTT
TAT--------------------------TACTACAAAAAAAATCTATTT
CGATTTTTTCAAAAACAAATCCAAGATTATTCTTGTTCCTATATAATTCT
CATGCTTGTGAATACGAATCCATNNNNNN----NNNNNNNNNNAACCAAT
TTTCTCATTTACGATTAACATCCTCTGGGGGCTTTTTTGAGCGAATTTAT
TTCTATGGAAAAATAAAACATCCTGTAGAAGAAGTCTTTGCTAATGATTT
TCCGGCTATTCGACGGGTCTTCAAAGATCCTTTCATGCATTATGTTAGAT
ATCGGGGAAAATCCATTTTGGTTTCAAAAGATACGCCTCTTCTAATGAAT
AAATGGAAATATTACCTTGTTCGTTNATGGCAANGGCATTTTNATGTGTG
GGCTCAACCCGGAAGGATCTACATAAACCAATTATTCAAGCATTCCTTCG
GCTTTTTGGGCTATCTTTCAAGTGTGCGACAAAATATTTCATTGGTACGG
AGTCAAACGCTCGAAAATTCATTTATAACGGATAATGCTATTAAGAAGCT
TGATACATTAGCTCCAATGCATCCTCTG----------------------

--------------------------AAAGCAAGTGTTGGATTCAAAGCC
GGTGTTAAAGATTATAAATTGACTTATTACACTCCTGACTATGAAACCAA
AGATACTGATATCTTGGCAGCATTTCGAGTAACTCCTCAACCTGGCGTTC
CACCTGAGGAAGCAGGGGCCGCGGTAGCTGCTGAATCTTCTACTGGTACA

TGGACAACTGTATGGACTGACGGGCTGACCAGTCTTGATCGTTACAAAGG
TCGATGCTACCACCTCGAGCCCGTTGCTGGAGAAGAAAATCAATATATTG
CTTATGTAGCTTACCCCTTAGACCTTTTTGAAGAAGGTTCTGTTACTAAC
ATGTTTACTTCCATTGTGGGTAATGTTTTTGGGTTCAAGGCCCTGCGCGC
TCTACGTCTGGAAGATTTGCGAATCCCCCCTGCTTATTCTAAAACTTTCC
AAGGACCTCCTCACGGCATCCAAGTTGAAAGAGATAAATTGAACAAGTAT
GGCCGTCCCCTATTGGGATGTACTATTAAACCTAAATTGGGGTTATCCGC
TAAGAATTACGGGAGAGCCGTTTATGAATGTCTTCGCGGTGGACTTGATT
TTACCAAAGATGATGAGAACGTGAATTCCCAACCATTTATGCGTTGGAGA
GACCGTTTCTTATTTTGTGCCGAAGCCCTTTATAAAGCACAGGCTGAAAC
AGGTGAAATCAAAGGGCATTACTTGAATGCTACTGCAGGTACATGCGAAG
AAATGATTAAAAGGGCTGTATTTGCCAGAGAATTGGGAGTTCCTATTGTA
ATGCATGATTACTTAACAGGGGGATTCACTGCAAATACTAACTTGGCTCA
TTATTGCCGAGATAATGGTCTACTTCTTCACATCCACCGTGCAATGCATG
CAGTTATTGATAGACAGAAAAATCATGGTATACACTTTCGTGTACTAGCT
AAAGCGTTACGTATGTCTGGTGGAGATCATATTCACTCTGGTACTGTAGT
AGGTAAACTTGAAGGGGAAAGAGACATCACTTTAGGCTTTGTTGATTTAC
TACGTGATGATTTTATTGAAAAAGATCGAAGCCGTGGTATTTATTTCACT
CAAGATTGGGTCTCTCTACCAGGTGTTCTGCCTGTGGCTTCAGGGGGTAT
TCATGTTTGGCATATGCCTGCTCTGACCGAGATCTTTGGAGATGATTCCG
TACTACAGTTTGGCGGAGGAACTTTAGGACACCCTTGGGGAAATGCACCC
GGTGCCGTAGCTAATCGAGTAGCTCTAGAAGCATGTGTACAAGCTCGTAA
TGAGGGACGTGATCTTGCTCGTGAGGGTAATGAAATTATCCGTGAAGCTA
GTAAATGGAATGCTGAACTACCTGCTGCTTGTGAAATATGGAAGGAGACC
AAATTTGAATTCCAAACAATGGATACTTTGTAA----------------T
GAC--------------ACGAGGATTTTCAGTCCTCTGCTCTA-------
-CCAGCTGAG-CTATCCCGACCATTCACGACGCATCATCC----------
--TCAT-TTTAATAGATGGCTTGGGTCTATGTCAATTTAAAAAACGAAAA
GGGGATTACAAAGTCTTATCCAGGCCGTGGTGGAATTTCTTAGATCTTCA
A-----------------------------CA---AGAAGA-CTTTG---
TAAGTTTCAATACAGTACGAGCGATAATCTGATCA---------------

----------------------------------TTCA-ATTT-AC----
------AAT---------CGGGGTTACTTGCTCAATGATGTTCATTTGTA
CGTGTATCATATATATCGGCT---TGTGAAAAGAAAAAAATGAATGAATG
AGAAACCTAACGAATTTTGAACCGCTAAC---GAAATGAGAGTATAGGAT
AAAAAATTAGGGAATCAAATG--GGCCT---TTT----TGGGGATAGAGG
GACTTGAACCTTTCTAAAGTTAACGGATTTTCCTCTTACTATAAATTTCA
TTGTTGTCGATATTGACATGTAGAATGGGACTCTATCT------TTATTC
TCGTCCAATTAATCCATTTTTCAA----AAGATCTAT-CAGATTACGGTG
GAGTAAATGATTTGATCAATGAATAT----TCCAGATAGTATTTCAGTAC
GT---------ATATATAGGTTTATTCTTCATCCTTTCTGGAGTTTCGAT
GGAAGGATTC--------------------------------------CT
TTACTAACGCATCGCAGCCAACTCCA----TTTGTTAGAACAGCTTCCAT
TGAGT--------CTCTGCACCTATCCTTTTTTATTATCACTTTCTGAAT
CCTTGTTTGTTTTCAGA---------------AAACAGGATTTGGCTCAG
GATTGCCCATTTGT------AATTCCAGGGTTTCTCTGAATTTGAAAGTT
A-------------TCACTTGGTAGGTTTCC-------------------

--------TCGAAA-CCT-GCCCAGCAGAACTACCCGCGAACCCGTAAAA
ACACACTCGGAGGGGCCTTG-----GCCCTGTGC--CTC-GGGCTCTCT-
TGGCCGGG-GCGGCTG---CTCCCCGTGCACTGCATGTCCGTGCACGGGT
GCCG--CTTTCCTCGGCTG-CACAAACGAACCCCGGCGCAAACCG-CGC-
CAAGGAACATATAACGGATTGGCAC-CGCCTCGGCACCCCAGAGATGGTG
CGTGGACGGGGCGTGCGT--CGTATTC--TACAATGTCAAAACGACTCTC
GGCAACGGATATCTCGGCTCTCGCATCGATGAAGAACGTAGCGAAATGCG
ATACTTGGTGTGAATTGCAGAATCCCGTGAACCATCGAGTTTTTGAACGC
AAGTTGCGCCCGAAGCCA--TTAGGCCGAGGGCACGTCTGCCTGGGCGTC

ACACAATGTTGCCCCCTCCAGCCTCGGCATCGA-------GGG-------
-----------CGGGCGGGGGGGCGGACGATGGCCTCCCGTGTGCCGAGC
CATG--CGGCTGGCCAAAAT--GCGAGTCCTCGGCGACGAGTGCCGCGGC
AATCGGTGGTTTTCCAACCCTCG-GTGCCCTGCCGCGTGCACGGACCGCC
GCTGCGGCCCGACAGACC---CCAT-TGCGGT---GCG------------

>Spyridium_globulosum
CCCTTATGGCCCCGTTCAAGTCGAATTTAAAATGGAGGAATTTCAAAGAT
ATTTCGAACTCGATAGATCTCAGCAGCATGACCTCCTATACCCACTTATC
TTTCGGGAGTATATTTATGCATTTGCTCATGATCATGGCTTAAATAGATC
AGATTTGTTGGAAAATGTAGGTTATGACAATAAATCTAGTTTACGAATTG
TAAAACGTTTCATTACTCGAATGTATCACCAGAATCATTTGATTATTTCC
GCTAACTATTCTACCCAAAATAAGTGTTTTGGGTACAACAAGAATTTGTA
TTCTCAAATGATATCAGAGGGATTTGCAGTCATTGTGGAAATTCCATTTT
CCTTACGATTACCACCNNNNNNNNNTTCGGGGACAGAAATTGTAAAATAT
TCTAATTTACGATCAATTCATTCAATATTTCCATTTTTAGAGGACAAATT
CCCACATTTAAATTATGGATCAGATATACGAATACCCTACCCCATTCATC
TGGAAATCTTGGTTCAAATTCTTCGCTATTGGGTGAAAGATGCCTCTTCT
TTGCATTTATTACGGCTTTTTCTTCACGAGTATTCTAATTGGAATAGTTT
TAT---------------------------TACTACAAAAAAATCTATTT
CGATTTTTTCAAAAACAAATCCAAGATTATTCTTGTTCCTATATAATTCT
CATGCTTGTGAATACGAATCCATNNNNNNN----NNNNNNNNNNAACCAAT
TTTCTCATTTACGATTAACATCCTCTGGGGGCTTTTTTGAGCGAATTTAT
TTCTATGGAAAAATAAAACATCCTGTAGAAGAAGTCTTTGCTAATGATTT
TCCGGCTATTCGACGGGTCTTCAAAGATCCTTTCATGCATTATGTTAGAT
ATCGGGGAAAATCCATTTTGGTTTCAAAAGATACGCCTCTTCTAATGAAT
AAATGGAAATATTACCTTGTTCGTTNATGGCAANGGCATTTTNATGTGTG
GGCTCAACCCGGAAGGATCTACATAAACCAATTATTCAAGCATTCCTTCG
GCTTTTTGGGCTATCTTTCAAGTGTGCGACAAAATATTTCATTGGTACGG
AGTCAAACGCTCGAAAATTCATTTATAACGGATAATGCTATTAAGAAGCT
TGATACATTAGCTCCAATGCATCCTCTG----------------------

---------------------------AAGCAAGTGTTGGATTCAAAGCC
GGTGTTAAAGATTATAAATTGACTTATTACACTCCTGAATATGAAACCAA

AGATACTGATATCTTGGCAGCGTTTCGAGTAACTCCTCAACCTGGAGTTC
CGCCTGAGGAAGCGGGGGCCGCGGTAGCTGCTGAATCTTCTACTGGTACA
TGGACAACTGTATGGACTGACGGGCTTACCAGTCTTGATCGTTACAAAGG
TCGATGCTACCACATCGAGCCCGTTGCTGGAGAAGAAAGTCAATTTATTG
CTTATGTAGCTTACCCCTTAGACCTTTTTGAAGAAGGTTCTGTTACTAAC
ATGTTTACTTCCATTGTGGGTAATGTTTTTGGGTTCAAGGCCCTGCGCGC
TCTACGTCTGGAGGATTTGCGAATCCCTACTGCTTATACTAAAACTTTCC
AAGGACCGCCTCACGGCATCCAAGTTGAAAGAGATAAATTGAACAAGTAT
GGCCGCCCCCTATTGGGATGTACTATTAAACCTAAATTGGGGTTATCCGC
TAAGAATTACGGTAGAGCAGTTTATGAATGTCTTCGCGGTGGACTTGATT
TTACCAAAGATGATGAGAACGTGAATTCCCAACCATTTATGCGTTGGAGA
GACCGTTTCTTGTTTTGTGCCGAAGCCCTTTATAAAGCACAGGCTGAAAC
AGGTGAAATCAAAGGGCATTACTTGAATGCTACTGCAGGTACATGCGAAG
AAATGATGAAAAGGGCTGTATTTGCCAGAGAATTGGGAGTTCCTATTGTA
ATGCACGATTACTTAACAGGGGGATTCACTGCCAATACTAGCTTGGCTCA
TTATTGCCGAGATAATGGCCTACTTCTTCACATCCACCGTGCAATGCATG
CAGTTATTGATAGACAGAAAAATCATGGTATACACTTTCGTGTACTAGCT
AAAGCGTTACGTATGTCTGGTGGAGATCATATTCACTCTGGTACTGTAGT
AGGTAAACTTGAAGGGGAAAGAGAAATCACTTTAGGCTTTGTTGATTTAC
TACGTGATGATTTTGTTGAAAAAGATCGAAGCCGTGGTATTTATTTCACT
CAAGATTGGGTCTCTCTACCAGGTGTTCTACCTGTGGCTTCAGGGGGTAT
TCATGTTTGGCATATGCCTGCTCTGACCGAGATCTTTGGAGATGATTCCG
TACTACAGTTTGGCGGAGGAACTTTAGGACACCCTTGGGGAAATGCACCC
GGTGCCGTAGCTAATCGAGTAGCTCTAGAAGCATGTGTACAAGCTCGTAA
TGAAGGACGTGATCTTGCTCGTGAGGGTAATGAAATTATCCGTGAGGCTA
GTAAATGGAGTCCTGAACTAGCTGCTGCTTGTGAAGTATGGAAGGAGATC
GAATTTGAATTCGAAGCAATGGATACCTTGTAA-----GTAGACGCTACG
GACTTAATTGGATTGAGCCTTG-GTATGGAAACCTACCAAGTGATAACTT
TCAAATTCAGAGAAACCCTGGAATTACAAATGGGCAATCCTGAGCCAAAT
CCTGTTTTNTGAAAACAAACAAGTATTCA-------GAA---A---GTGA
TAATAAAAAAGGATAGGTGCAGAGACTCAATGGAAGCTGTTCTAACAAAT
GG----AGTTGGCTGCGATGCGTT------AGTAAAGGAAT-CCTTCCAT
CGAAACTCCAGAAAGGATGAAGAATAAACCTATATATACGTATACGTACT
GAAATACTATCTCCAAACCAAATGATTAATGACGACCCGAATATTTTTTT
T----------------TTATATGTTTATATGAAA--------------A
ATGAAAGAATTGTTGTGAA-----TCGATTCCAAGTAAAAA------AAA
AAATGGAATATTCATTGATCAAATCATTTACTCCATCGTAATCTGAT---
-AGATCTTTTGAAAAATGGAT------TAATCGGACGAGAATAAAGATAG
AGTCCCATTCTACATGTCAA------------TATCGACAACAATGAAAT
TTATAGTAAGAGGAAAATCCGTCGACTTTA-GAAATCGTGAGGGTTCAAG
TCCCTCTATCCCCAAAAAGGC-CCAGTTGATTCCCTAATTTTTTATC---
--CTATACT-C----TCATTTCGTTATCGGTTCAAAGTTCATTATGTTTC
TCATTC------ATTCATT-TTTT--------------------------
------TCT--TTTCACAAGCCTTGT----GGTATATATGATACACA---
----TAC--------AAATGAACATCA--TTGAGCAAGT--AACCCCGAT
TGTAAATTGG-------AATGATC-ATATT--------ATCGCCCGTACT
GTACTGAAA----CTTACAAAGTCTTTTTT----T-TTAA--GATCTAAG
AAATT----CCACCAAGGCCTGGATAACACTTTGTAAT----CCCCTTTT
CGTCTTTTTAATTGACATAGACCCAAGTCATCTATTAA------AATGAG
GATGATGCGTCGTG-----AATGGTCGGGATAGCTCAGCTGGTAGAGCAG
AGGACTGAAAATCCTCGTGTCACAACTTTCAAATA---------------
GGCGGTTCGCTGCCTGCGACGTCGCGAGAAGTCCACTGAACCTTATCATT
TAGAGGAAGGAGAAGTCGTAACAAGGTTTCCGTAGGTGAACCTGCGGAAG
GATCATTGTAGAAG-CCT-GCCTAGTCGAACGACCCGCGAACTCGTAAAA
AAAATTTCTGGGGGGCTTCGG---GGCCTTAGGC--CTC-GGGCTTTCC-
TGGTCGGG-GGCTGCA---TGCTCTG--TGTCGCTTGACGATGCATGGGT
GCTGT-TTCCCCCGGCTGT--AAAAACGAACCCCGGCGCAAACCGCGCCA
AGGAA--CTTCCAAAGAATTAGCAC-CGCCCTGTCACCCCAGAAATGGTG
TGTGGCCGTGGTGTGCGT--CGTATTC--AACTAAGTCAAAACGACTCTC
GGCAACGGATATCTCGGCTCTCGCATCGATGAAGAACGTAGCGAAATGCG

ATACTTGGTGTGAATTGCAGAATCCCGTGAACCATCAAGTCTTTGAACGC
AAGTTGCGCCCGAAGCCA--TTAGGCCGAGGGCACGTCTGCCTGGGCGTC
ACACAATGTTGCCCCCCCAACTTCGATCTCGAA-----------------
-----------GGTGGTGAGGGGCGGATGCTGGCCTCCCGTGCCTCACGG
TTTG--CGGCTGGTCAAAAT--GCGAGTCGCCGGCGACGAGTCCCGCAGC
AATCGGTGGTTCTCCAACCCTCG-GTGCCCTGCTGCGTGCACGAATCGCT
GCTGTGGACGGATAGACC---CCAA-TGCGC----TGCAGAAGCGGCGTT
TACAATGCGACCCCAGTCAGGCGGACCACCCAAGA---------------

>Karwinskia_calderonii

--TCTTCT
TTGCATTTATTACGGCTTTTTCTTCACGAGTATTCTAATTGGAATAGTTT
TAT---------------------------TACTACAAAAAAATCT----
--ATTTTTTCAAAAACTAATCCACGATTATTCTTGTTCCTATATAATTCT
CATGTTTGTGAATACGAATCCATCTTACT----TTTTCTCCGCAACCAAT
TTTCTCGTTTACGATTAACATCTTCTGGGGGCTTTTTTGAGCGAATATAT
TTCTATGGAAAAATAAAACATCCTGTCGAAGAAGTCTTTGCTAATGATTT
TACGGCTATTCGACGGGTCTTCAAGGATCCTTTCATGCATTATGTTAGAT
ATCGGGGAAAATCCATTTTGGTTTCAAAAGATACGCCTCTTCTAATGAAT
AAATGGAAATATTACCTTGTCCATTTATGGCAATGGCGTTTTTATGTGTG
GGCTCAACCCGGAAGGATCTACATAAACCAATTATTCAAGCATTCTTTCG
GCTTTTTGGGCTATCTTTCAAGTGGGCGACTAAATCGTTCATTGGTACGG
AGTCAAACGCTCGAAAATTCATTTATAACGGATAATGCTATTAAGAAGCT
TGATACATTAGCTCCAATTAGTCCTCTGATTGGATCGTTGTCTAAAATGA
AATTTTGTAGCGCACTAGGACATCCTATTAGTAAGTCGACCTGGACCGAT
TCGTCGGATTTTTCTATTATCGACCGCTTTGCGCGTATATGCAGAAATCT
TTCTCATTATTACAGCGGGTCCTCAAAAAAAAAAAAGTTTGTATCGAATAA

-ATAACTTCCCTCTAGACCTAGCTGCTGTCGACGTTCCATCTACAAATGG
ATAAGACTTCGCTTT---GGTATTAGTGTATACGAGTTCTT-GAAAATAA
AGGAGCAATAATCAATTTCTTGTTCTATCAAGAAGTTTGTTATTGCTCCT
TTA-TTATTTAGGAGTCGTTTTTCTTACGTAAGTTTTTTTC-------TT
TACTTCAACAAATTCTTCGTT----ATGTAGAAACTTTTTTTTTATATAT
GTTCTTCTCAATCTTTTTTTA-----CTTT---TTTATTTATT---TTTT
TACATTTTTTTAAATAAAAGAAGGGAAA-CCATTATCTTTATCTTGTATT
TCAT-----CTTATAAGAAAGAAAAAAGGTGA---------ATGGTTTGA
CATATTTGG------------------TTTGGAATTTGTAAATAGTCTAG
GGGCGGATGTAGCCAAGTGGATCAAGGCAGT-------------------

-----------CCCAGAGCATACCCAACCCGCTATAATATTCATATATAC
AATGAT---AATATATATCAGAAAAAGATTGCCCTTTTATTTTAAAATAA
AAATGAAATA------------AAATGAAACATTCTTAAGAGTATAATAT
TGGAAAAA------------------TTGGATTATTATTCTTAATACTAT
AATTCTTTTCTGAATCATATCAATTTCACAAATGGAATCGTATTCAAATA
ACACACAGAGGGAATTGAATCTATTTGTCATCCCTAAATGTTAAATATTT
AACTTTAACATAGAATATCTAGAAT------TAGTAACAATTGTTTAGTC
TATCTGATAAATAT----GAGG-------GTCCCATATTATA-TTATTGT
TTTGGGGA--TTCTGGTTTTATCAAACAGTATGAAAAAATCACAACCCTC
TCTTACATCAGTCTTTATCCACTATTTCACTAAAAAAAGAAATAATTTTT
TTTTATT-TCTAATT-TT-------TTTTA----------TA--TTATAT
GTTTTTTATTTCTATAACATTAAAAAATAAAA------------------
--------------------------AAAAAA----TTAGAATATTAAC-
--------------------TTAATAAATATATAAAAAAAATGGAATTGA
AATATTCC--------ATTTTAAGATTAATA-------------------

------------------------------A---------------ATAT
ATGTATATGGTATTAAATGGAATTCTTTCTGAGACTTCGTCAGAAACCAG
CCATTCATATTTCATATAGAAATAAAGG-------------------GTA
TAGATTATTATGTACTGGCCGAACCAATGACTATTCGTGATTCCATAATT
GAATCAATTACATACTGGTTCCAATCTAAAGGAATGTTATGGTAAAACTT

CGTTT---------------------------AGTGTTGGATTCAAGGCT
GGTGTTAAAGATTATAAATTGACTTATTACACTCCTGAATATGAAACCAA
AGATACCGATATCTTGGCAGCATTTCGAGTAACTCCTCAACCCGGAGTTC
CACCTGAAGAAGCAGGGGCCGCAGTAGCTGCTGAATCTTCTACTGGTACA
TGGACAACTGTATGGACTGACGGGCTTACCAGTCTTGATCGTTACAAAGG
TCGATGCTACCACATCGAGCCCGTTGCTGGAGAAGAAACTCAATTTATTG
CTTATGTAGCTTACCCCTTAGACCTTTTTGAAGAAGGTTCTGTTACTAAC
ATGTTTACTTCCATTGTGGGTAATGTATTTGGGTTCAAGGCCCTGCGCGC
TCTACGTCTGGAGGATTTGCGAATCCCCACTGCTTATACTAAAACTTTCC
AAGGCCCGCCTCATGGCATCCAAGTTGAGAGAGATAAGTTGAACAAGTAT
GGCCGCCCCCTATTGGGATGTACTATTAAACCGAAATTGGGGTTATCCGC
TAAGAATTACGGTAGAGCCGTTTATGAATGTCTT----------------

------------CAAAAAGTC-CCATTGGATTCCCTAATTATTTATC---
-------CTATGCTCTCATTTCGTTAACGGTTCAAAATTCGTTATGTTTC
TCATTCATTCTACTCTTTTACTTTATAAATGGTCTGAGCGGAAATTTTTT
TCT--------TTTC-------------------ACAAGCCTTGTGATAT
ATAGGATACACGTACAAATGAACATCGTTGGGCACGT----AACCCCGAT
TGTAAATTGT-------AATGATT-AACAATACATATTATTACTTGTACT
GTACTGAAACTTACA----AAGTC------TTCTTTTTG-AAGATCCAAG
AAATT----CCATCAAGACCCGGATAAGGTTTTGTAATC----CCCTTTT
CGTCTTTTTCATTGACATAGAACCAAGTCCTCTATTAATATTAAAATGAG
GATGGTGCGTCGTG-----AATGGTCGGGATAGCTCAGCTGGTAGAGCAG
AGGACTGAAAATC-------------------------------------

-------GTAG--AAACCTGCACAGCAGAACGACCCGTGAACCCGTAAAG
CACAGCTTGGGGGCGAGGGGC---CAACCCGGAC--CCC-C----TTTGG
TGGGTGG--GCGGCGA---C-------CCGGGCATCGATGATGCCCGCCG
TCATCGCCTTCCC-CGCCG-CACAAACGAAACCGGGCGCAAAACGCGCCA
--AGGAACCAAAACGAATCAAG--CACGCCCCGGCGCCCCAGAGATGGTT

CGCGCTCGGGGTGCTGCG--TCGTAT--TCTATATGTCAAAACGACTCTC
GGCAACGGATATCTCGGCTCTCGCATCGATGAAGAACGTAGCGAAATGCG
ATACTTGGTGTGAATTGCAGAATCCCGTGAACCATCGAGTCTTTGAACGC
AAGTTGCGCCCGAAGCCA--CTAGGCCGAGGGCACGTCTGCCTGGGCGTC
ACACAACGTTGCCCCCCCCCAACTCCACCCCGTCTTCGAGGGG-------
-----------GACTGGGGGGGGCGGATGCTGGCCTCCCGTGCGCCATTG
CTCG--CGGCTGGCCCAAAT--GCGAGCACTCGGCGATGAGCGCCGCGGC
AATCGGTGGTTGTCCAACCCTCG-GTGCAACGCTGCGCGCGCGAGCCGCC
CATCCGTGCCCGTCGGCC---CCAA--CGCGCC---GCAAATGCGGCGTC
CACAACGCGACCCCAGT---------------------------------

>Berchemia_discolor

--TCTTCT
TTGCATTTATTACGGCTTTTTCTTCACGAGTATTCTAATTGGAATAGTTT
TAT---------------------------TACTCCAAAAAAATCT----
--ATTTTTTCAAAAACTAATCCACGATTATTCTTGTTCCTATATAATTCT
CATGCTTGTGAATACGAATCCATCTTACT----TTTTCTCCGCAACCAAT
TTTCTCGTTTACGATTAACATCTTCTGGGGGCTTTTTTGAGCGAATATAT
TTCTATGGAAAAATAAAACATCCTGTAGAAGAAGTCTTTGCTAATGATTT
TCCAGCTATTCGACGGGTCTTCAAGGATCCTTTCATGCATTATGTTAGAT
ATCAAGGAAAATCCATTTTGGTTTCAAAAGATACGCCTCTTCTAATGAAT
AAATGGAAATATTACCTTGTCCGTTTATGGCAATGGCATTTTTATGTGTG
GGCTCAACCCGGAAGGATCTACATAAACCAATTATTCAAGCATTCCTTCG
GCTTTTTGGGCTATCTTTCAAGTGTGCGACTAAATCTTTCATTGGTACGG
AGTCAAATGCTCGAAAATTCATTTATAATGGATAATGCTATTAAGAAGCT
TGATACATTAGCTCCAATTAGTCCTCTGATTGGATCGTTGGCTAAAATGA
AATTTTGTAGCGCACTAGGACATCCTATTAGTAAGTCGACCTGGACCGAT
TCGTCGGATTTTTCTATTATCGACCGCTTTGCGCGTATATGCAGAAATCT
TTCTCATTATTACAGCGGGTCCTCAAAAAAAAAGAGTTTGTAT-------

--------------------------------ATATTTATAGTGATAATT
ATATGGCTCATGATCGGGGATATTTGAGGTTTTTTGCTTATATGAGTTTT
TCTAATACTTCAATGTTAGGATTGGTTACTAGTTCAAATTTGATACAAAT
TTATTTTTTTTGGGAATTGGTTGGAATGTGTTCTTATCTATTAATAGGCT
TTTGGTTCACACGACCTATTGCGGCGAATGCCTGTCAAAAAGCGTTTGTA
ACTAACCGCGTAGGGGATTTTGGTTTATTATTAGGAATTTTAGGTTTTTA
TTGGATAACGGGTAGTTTAGAATTTCGGGATTTGTTTGAAATATTCAATA
ACGTGGTTTATAATAATGAAGTTAATTTTTTATTTGTTACTTTGTGTGCC
TTTCTATTATTTGCCGGTGCAGTTGCTAAATCTGCCCAATTTCCCCTTCA

TGTATGGTTACCCGATGCTATGGAAGGGCCTACCCCTATTTCGGCTCTTA
TACACGCTGCTACTATGGTAGCAGCGGGAATTTTTCTTGTAGCTCGGCTT
CTTCCGCTTTTCATAGTTATACCTTTCATAATGAATCTAATAGCCTTGAT
TGGTATAATAACATTACTTTTAGGAGCCACTTTTGCTCTTGTTCAAAAAG
ATATTAAGAGAGGTCTAGCTTATTCTACAATGTCTCAATTGGGTTATATG
ATGTTGGCTCTAGGTATGGGTTCTTATCGAGCTGCTTTATTTCATTTGAT
TACTCATGCTTATT------------------------------------

-----------------------------CGACGTTCCGTCTATAAATGG
ATAAGACTTCGCTTT---GGTATTAGGGTATACGAGTTCTTTGAAAATAA
AGGAGCAATAATAAATTTCTTGTTCTATCAAGAAGTTTGTTATTGCTCCT
TTA-TTATTTAGGAGTCTTTTTTCTTACGTGAATTTTTTTC-------TT
TACTTCAACAAATTCTTCGTT----ATGTAGAAACTTTTATTTTCTATAT
GTTCTTCTCAATCTTTTTTTA-----ATTT---TTTATTTTACA------
----TTTTTTTAAATAAAAGAAAGGAAACCATTATATTTTATCTTGTATT
TCATCTTAGAAGAGAAGAAAGAAAAAAGGTGA---------ATGGTTTGA
CATATTTGG------------------TTTGGAATTTGTAAATAGTCTAG
GGGCGGATGTAGCCAAGTGGATCAAGGCAGTGGATTGTGAATCA------
----------------GCAACGGGTTTTGGTCCCGCTATTCGGAGGTTCG
AATCCTTCCGTCCCAGAGCATACCCAACCCGCTATAATATTCATATATAC
AATGAT---AATATATATCAGAAAAAGATTGCCCTTTTAAAATGAAAC--
-------------------------------ATTCTTAAGAGTATAATAT
TGGAAAAATTG------------------GATTATTATTCGTAATACTAT
AATTCTTTTCTGAATCATATCAATTTCACAAATTGAATCGTATTCAAATA
ACACACAGAGGGAATTGAATCTATTTGTCATTCCTAAATGTTAAA-----
-TGTTTAAGATAGAATATCTATAATTCATTTCAGTAACAATTGTTTAGTC
TATCTGATAGATAT----GGGG-------GTTCCATATTATTGTTTTGGG
GATTCTGGTTTTATC--------AAACAGTATGAAAAAATAACAACCCTC
CCTTACATCAGTCTTTATCCCCTATTTCACTAAATAAAATAAATTATTTA
TTTTATTCGTGTTATTTATCATTTTTTTTA----------TA--TTATAT
GTTTTTTATTTCTATAATATAAAAAAA--------------------AAA
TTCAATTAAAATATTAACTTAATAAATACTTA----ATAAATATAAATA-
--------------------AACTTAATAAATATAAATAAATGGAATTGA
AATATTCC--------ATTTTAAGATTAATA-------------------

------------------------------AATATATGTATATGGTATTA
AAGTATATGGTATTAAATTGAATTCTTTCTGAGACTTAGTCAGAAACTAG
CCATTCATATTTCATATAGAAATAGAAAAGTATAGTAGAAAAGTATAGAT

TATATTATTATGTACCGGCCGAACCAATGACTATTCGTGATTCCATAATT
GAATCAATTACATACTGGTTCCAATCTAAAGGAATGTTATGGTAAAACTT
CGTTT--------------------------AAGTGTTGGATTCAAAGCT
GGTGTTAAAGATTATAAATTGACTTATTACACTCCTGAATATGAAACCAA
AGATACCGATATCTTGGCAGCGTTTCGAGTAACTCCTCAACCCGGAGTTC
CACCTGAGGAAGCAGGGGCCGCGGTAGCTGCTGAATCTTCTACTGGTACA
TGGACAACTGTATGGACTGACGGGCTTACCAGTCTTGATCGTTACAAAGG
TCGATGCTACCACATCGAGCCCGTTGCTGGAGAAGAAAGTCAATTTATTG
CTTATGTAGCTTACCCCTTAGACCTTTTTGAAGAAGGTTCTGTTACTAAC
ATGTTTACTTCCATTGTGGGTAATGTATTTGGGTTCAAGGCCCTGCGCGC
TCTACGTCTGGAGGATTTGCGAATCCCCCCTGCTTATTCTAAAACTTTCC
AAGGCCCGCCTCATGGCATCCAAGTTGAAAGAGATAAGTTGAACAAGTAT
GGCCGCCCCCTATTGGGATGTACTATTAAACCGAAATTGGGGTTATCCGC
TAAGAATTACGGTAGAGCCGTTTATGAATGTCTTCGCGGTGGACTTGATT
TTACCAAAGATGATGAGAACGTGAATTCCCAACCGTTTATGCGTTGGAGA
GACCGTTTCTTATTTTGTGCCGAAGCAATTTATAAAGCACAGGCCGAAAC
TGGTGAAATCAAAGGGCATTACTTGAATGCTACGGCAGGTACATGCGAAG
AAATGATCAAAAGGGCTGTATTTGCCAGAGAATTGGGAGTTCCTATCGTA
ATGCACGATTACTTAACGGGGGGATTCACTGCAAATACTAGCTTGGCTCA
TTATTGCCGAGATAATGGTCTGCTTCTTCACATCCACCGTGCAATGCATG
CCGTTATTGATAGACAGAAAAATCATGGTATGCACTTTCGTGTACTAGCT
AAAGCGTTACGTCTGTCTGGTGGAGATCATATTCACGCTGGTACTGTAGT
GGGTAAACTTGAAGGGGAAAGAGACATCACTTTAGGCTTTGTTGATTTAC
TACGTGATGATTTTGTTGAGAAAGATCGAAGCCGTGGTATTTATTTCACT
CAAGATTGGGTCTCTCTACCAGGTGTTATACCCGTGGCTTCAGGAGGTAT
TCATGTTTGGCATATGCCTGCTCTGACCGAGATCTTTGGAGACGATTCCG
TACTACAATTCGGCGGAGGAACTTTAGGGCACCCTTGGGGAAATGCACCC
GGTGCCGTAGCTAATCGAGTAGCTCTAGAAGCATGTGTACAAGCTCGTAA
TGAGGGACGTGATCTTGCTCGTGAAGGTAATGAAATTATCCGTGAGGCTA
GTAAATGGAGTCCTGAACTAGCTGCTGCTTGTGAAGTATGGAAGGAGATT
AAATTTGAATTCCCAGCAATGGATACTTTGTAA-----------------
---------------------TGGTATGGAAACCTACCAAGTGATAACTT
TCAAATTCAGAGAAACCCTGGAATTAAAAACGGGCAATCCTGAGCCAAAT
CCTGTTTTCTGAAAACAAACAAAGGTTCAGAAAGCGATAATAAA-AA--A
GGATAGATAGG----GATGCAGAGACTCAATGGAAGTTGTTCTAACAAAT
GG----AGTTGGCCACGATGCGTT------AGTAAAGGACT-CCTTCCAT
CGAAACTCCAGAAAGTATGAAGAATAAACGTATATATACGTACTGAAATA
CTATCTCCAACTCCAAACCAAATGATTAATGACGACCCGAATCTTTTTT-
--TTTTATATATATAAAA-------------------------------A
ATGAAAGAATTGTTGT----------GAATCGATTCCAAGTTG-AAAACA
GAATCGAATATTCATTGATCAAATCATTTACTCCATCGTAATCTGAT---
-AGATCTTTTGAAGAATTGAT-----T-AATCGGACGAGAATAAAGATAG
AGTCCCGTTCTACATGTCAA------------TATCGACAACAATGCAAT
TTATAGTAAAAGGAAAATCCGTCGACTTTA-GAAATCGTGAGGGTTCAAG
TCCCTCTATCCCCAAAAAGTC-CCATTGGATTCCCTAATTATTTATC---
-------CTATGCTCTCATTTCGTTAACGGTTCAAAATTCGTTATGTTTC
TCATTCATTCTACTCCTTTACTTTACAAATGGTC------TGAGCGGAAA
TTG--------TTTTCTTTTCACAA-----GCC--------TTGTGGTAT
ATAGGATACACGTACAAATGAACATCGTTGGGCGCGT----AACCCCGAT
TGTAAATTGT-------AATGATT-AACAATACATATTATTAGTTGTACT
GTACTGAAACTTACA----AAGTC------TTCTTTTTG-AAGATCCAAG
AAATT----CCACCAAGACCTGGATAAGGCTTTGTAATC----CCCTTTT
CGTCTTTTTCATTGACATAGAACCAAGTCCTCTATTA------AAATGAG
GATGATGCGTCGTG-----AATGGTCGGGATAGCTCAGCTGGT-------

---G
GATCATTGTTGAAACCCT-GCACAGCAGAACGACCCGTGAACCTGTAAAA
CACATCTCGGGGGCGAGGGGC---CAACCCGGAC--CCC-C----T----
-GGGCGG--GGGGTGC---CAGCACGGGCGTCGGTGGCCGGCGCCCGCGG

CTGTTGCCTTCCCC-GCCG-CACAAACGAACCCCGGCGCAAACCGCGCCA
--AGGAACTCGAACAGATCGG---CATGCCCCGTCGGCCCAGAGATGGTG
CGCGCTCGGGGTGTTGCA--TCGTATTATGTGTATGTCAAAACGACTCTC
GGCAACGGATATCTCGGCTCTCGCATCGATGAAGAACGTAGCGAAATGCG
ATACTTGGTGTGAATTGCAGAATCCCGTGAACCATCGAGTCTTTGAACGC
AAGTTGCGCCCGAAGCCA--TTAGGCCGAGGGCACGTCTGCCTGGGCGTC
ACACAACGTTGCTCCCCCAACCCA-AGCCTCGGCTCCGAG--G-------
------------AGGGCGGGGGGCGGATGCTGGCCTCCCGTGCGCCATGG
CTCG--CGGTTGGTCCAAAT--GCGAGTGCTCGGCGACGGGCGCCGCGGC
AATCGGTGGTTGTTCAACCCTCG-GTGCCACGCCGCGCGCGCGAGTCGCC
CACCGGTGCTCGGCGACC---CCCT-AATGCGC---C-------------

>Reynosia_uncinata

----------TACGGCTTTTTCTTCACGAGTATTCTAATTGGAATAGTTT
TAT---------------------------TACGACAAAAAAATCT----
--ATTTTTTCAAAAACTAATCCACGATTATTCTTGTTCCTATATAATTCT
CATGTTTGTGAATACGAATCCATCTTACT----TTTTCTCCGCAACCAAT
TTTCTCGTTTACGATTAACATCTTCTGGGGGCTTTTTTGAGCGAATATAT
TTCTATGGAAAAATAAAACATCCTGTAGAAGAAGTCTTTGCTAATGATTT
TCCGGCTATTCGAAGGGTCTTCAAGGATCCTTTCATGCATTATGTTAGAT
ATCAGGGAAAATCCATTTTGGTTTCAAAAGATACACCTCTTCTAATGAAT
AAATGGAAATATTACCTTGTCCGTTTATGGCAATGGCATTTTTATGTGTG
GGCTCAACCCGGAAGGATCTACATAAACCAATTATTCAAGCATTCCTTCG
GCTTTTTGGGCTATCTTTCAAGTGTGCGACTAAATCTTTCATTGGTACGG
AGTCAAACGCTCGAAAATTCATTTATAATGGATAATGTTATTAAGAAGCT
TGATACATTAGCTCCAATTAGTCCTCTGATTGGATCGTTGGCTAAAATGA
AATTTTGTAGCGCACTAGGACATCCTATTAGCAAGTCGACCTGGACCGAT
TCGTCGGATTTTTCTATTATCGACCGCTTTGCGCGTATATGCAGAAATCT
TTCTCATTATTACAGCGG--------------------------------

-------------------------CTGTCGACGTTCCGTCTACAAACGG
ATAAGACTTCGCTTT---GGTATTAGTGTATACGAGTTTTT-GAAAATAA
AGGAGCAATAATAAATTTCTTGTTCTATCAAGAAGTTTGTTATTGCTCCT
TTA-TTATTTAGGAGTCGTTTTTCTTACGTAAGTTTTTTTC-------TT
TACTTCAAAAAATTCTTCGTT----ATGTAGAAACTTTTTTTATATATGT
TCTTCTCATTTGAATTTTTTA-----TTTT---TTTT-----------TT
TTTACATAAATAAAAGAAGGAAGGGAAACCATTATCTTTTATCTTGTATT
TCATCTTAGAAGAAAGAAAAAAGG--------------------------

---------------------GGGTTTTGGTCCCGCTATTCGGAGGTTCG
AATCCTTCCGTCCCAGAGCATACCCAACCCCCTATAATATTCATATATAC
AATGAT---AATATATATCAGAAAAAGATTGCCCTTATTAAAATGAAATA
AAATTAAACATTCTTAAGAGTATAATATTACATTCTTAAGAGTATAATAT
TGGAAAAATTGGATTATTATTCTTAATACTA----------TAATTATTT
TATTATTTTCTGAATCATATCAATTTCACAAATTGAATCGTATTCAAATA
ACACACAGAGAGAATGGAATCTATTTGTCATCCTTAAATGTTAAA-TTAA
ATATTTAACATAGAATATATAGAATTCATTTCAGTAACAATTGTTTAGTC
TATCTGATAAATGA----TAAATATAAAGATCCCATATTATTGTTTTGGG
GATT--------CTGGTTTTATCAAACAGTATGAAAAAATAACAACCCTC
CCTTACATCAGTCTTTATCCACTATTTCACTAAAAAAATAAAAAATTTAT
TTTCTTTTTTTATTCATAATTTTTTGTATAT-------TATAT--GTATAT
GTTTTTTATTTCTATAACATTAAAAA------------------------
--------------------------AAAAAA----TGAAAATATTAAC-
--------------------TTAATAAATATATAAATAAAATGGAATTGA
AATATTCC--------ATTTTAAGATTAATA-------------------

------------------------------A---------------ATAT

ATGTATATGGTATTAAATTGAATTTTTTCTGAGACTTCGTTAGAAACCAG
CCATTCATATTTCATATAGAAATAAAAGGTATAGAT--------------

-----------------------------GCAAGTGTTGGATTCAATGCT
GGTGTTAAAGATTATAAATTGACTTATTACACTCCTGAATATGAAACCAA
AGATACCGATATCTTGGCAGCATTTCGAGTAACTCCTCAACCCGGAGTTC
CACCTGAAGAAGCAGGGGCCGCGGTAGCTGCTGAATCTTCTACTGGTACA
TGGACAACTGTATGGACTGACGGGCTTACCAGTCTTGATCGTTACAAAGG
TCGATGCTACCACATCGAGCCCGTTGCTGGAGAAGAAAATCAATTTATTG
CTTATGTAGCTTACCCCTTAGACCTTTTTGAAGAAGGTTCTGTTACTAAC
ATGTTTACTTCCATTGTGGGTAATGTATTTGGGTTCAAGGCCCTGCGCGC
TCTACGTCTGGAGGATTTGCGAGTCCCCCCTGCTTATTCTAAAACTTTCC
AAGGCCCGCCTCATGGCATCCAAGTTGAGAGAGATAAGTTGAACAAGTAT
GGCCGCCCTCTATTGGGATGTACTATTAAACCGAAATTGGGGTTATCCGC
TAAGAATTACGGTAGAGCCGTTTATGAATGTCTTCGCGGTGGACTTGATT
TTACCAAAGATGATGAGAACGTGAATTCCCAACCGTTTATGCGTTGGAGA
GACCGTTTCGTATTTTGTGCCGAAGCACTTTATAAAGCACAGGCCGAAAC
TGGTGAAATCAAAGGGCATTACTTGAATGCTACGGCAGGTACATGCGAAG
AAATGATGAAAAGGGCTGCATGTGCCAGAGAATTGGGAGTTCCTATCGTA
ATGCACGATTACTTAACGGGGGGATTCACTGCAAACACTACCTTGGCTCA
TTATTGCCGAGATAATGGTCTGCTTCTTCACATCCACCGTGCAATGCATG
CCGTTATTGATAGACAGAAAAATCATGGTATGCACTTTCGTGTACTAGCT
AAAGCGTTACGTCTGTCTGGTGGAGATCATATTCACGCTGGTACTGTAGT
GGGTAAACTTGAAGGGGAAAGAGACATCACTTTAGGCTTTGTTGATTTAC
TACGTGATGATTTTGTTGAGAAAGATCGAAGCCGTGGTATTTATTTCACT
CAAGATTGGGTCTCTCTACCAGGTGTTCTGCCCGTGGCTTCAGGGGGTAT
TCATGTTTGGCATATGCCTGCTCTGACCGAGATCTTTGGAGACGATTCCG
TACTACAATTCGGCGGAGGAACTTTAGGGCACCCTTGGGGAAATGCACCC
GGTGCCGTAGCTAATAGAGTAGCTCTAGAAGCATGTGTACAAGCCCGTAA
TGAGGGACGTGATCTTGCTCGTGAAGGTAATGAAATTATCCGTGAAGCTA
GTAAATGGAGTGCTGAACTAGCTGCTGCTTGTGAAGTATGGAAGGAGATT
AAATTTGAATTCCCAGCAATGGATACTTTGTAA-----------------
---TTAA-TGAGTG--AGCTTTGGTATGGAAACCTACCAAGTGATAACTT
TCAAATTCAGAGAAACCCTGGAATTAAAAACGGGCAATCCTGAGCCAAAT
CCTGTTTTCTGAAAACAAACAAAGGTTCAGAAAGCGATAATAAA-AA--A
GGATAGATAGGGATAGGTGCAGAGACTCAATGGAAGTTGTTCTAACAAAT
GG----AGTTGGCCACGATGT------------AAAGGACT-CCTTCCAT
CGAAATTCCAGAAAGTATGAAGAATAAACGTATATAT------ACGTACT
GAAATACTATCTCCAAACCAAATGATTAATGACGACCCGAATCTTTTTTT
----TTATTTATATAAAA--------------------------------A
ATGAAAGAATTGTTGT----------GAATCGATTCCAAGTTG-AAAACA
GAATCGAATATTCATTGATCAAATCATTTACTCCATCGTAATCTGAT---
-CGATCTTTTGAAGAATTGAT-----T-AATCGGACGAGAATAAAGATAG
AGTCCCGTTCTACATGTCAATATCGATGTCAATATCGACAACAATGCAAT
TTATAGTAAAAGGAAAATCCGTCGACTTTA-GAAATCGTGAGGGTTCAAG
TCCCTCTATCCCCAAAAAGTC-CCGTTGGATTCCCGAATTATTTATC---
-------CTATGCTCTCATTTCGTTAACGGTTCAAAATTCGTTATGTTTC
TCATTCATTCTACTCTTTTACTTTACAAATGGTC------TGAGCGGAAA
TTT--------TTTTCTTTTCACAA-----GCC--------TTGTGATAT
ATAGGATACACGTACAAATGAACATCGTTGGGCACGT----AACCCCGAT
TGTAAATTGT-------AAAGATT-AACAATACATATTATTACTTGTACT
GTACTGAAACGTACA----AAGTC------TTCTTTTTG-AAGATCCAAG
AAATT----CCACCAAGACCTGGATAAGGCTTTGTAATC----CCCCTTT
CGTCTTTTTCATTGACATAGAAGCAAGTCCTCTATTA------AAATGAG
GATGGTGCGTCGTG-----AATGGTCGGGATAGGTCAGCTGGTTGAGCAG
ACACTGA--

--AAG
GATCATTGTTGAAACCCT-GCACAGCAGAACGACCCGCGAACCTGTAAAA

TACAACTCGGGGGGCGAGGGG---CAACCCGGAC--CCC-C----CT---
-GGCTGG--GGAGCAC---CAACTCGGTCCTCGCTCGTCGATGCCCGCTG
TTTGTTGCCTTC--CCCGC-CGCAAACGAACCCCGGCGCAAACCGCGCCA
--AGGAACYCGAACGAATCAAA-GCGCGCCCCGACGCCCCAGAGATGGTG
TGCG-TTTGGGTGCCGCG--TCGTAT--TCTATATGTCAAAACGACTCTC
GGCAACGGATATCTCGGCTCTCGCATCGATGAAGAACGTAGCGAAATGCG
ATACTTGGTGTGAATTGCAGAATCCCGTGAACCATCGAGTCTTTGAACGC
AAGTTGCGCCCGAAGCCA--ATAGGCCGAGGGCACGTCTGCCTGGGCGTC
ACACAACGTTGCCCCCCAA-CCACAAACCCCGACCCCGGG--G-------
-----------AGGGCWGGGGGGCGGATGCTGGCCTCCCGTGAACCATGG
CTCG--CGGCTGGCCCAAAT--GCGAGCACCCGGAGACTAGCGCCGCGGC
GATCGGTGGTTGTCCRACCCTCG-GTGCCACGCTGCGCGCGCGAGTCTCC
CATCCCCGCTCCTATGGC---C-CA-ACGCGTC---GCACACGCGGCGTT
CACAACGCGACCCCAGGTCAGGCGGGGCTACCCGCTGAGTTTAA------

>Krugiodendron_ferreum

-----------ACGGCTTTTTCTTCACGAGTATTCTAATTGGAATAGTTT
TAT---------------------------TACTACAAAAAAATCG----
--ATTTTTTCCAAAACTAATCCACGATTATTCTTGTTCCTATATAATTCT
CATGTTTGTGAATACGAATCCATCTTATT----TTTTCTCCGCAACCAAT
TTTCTCGTTTACGATTAACATCTTCTGGGGTCTTTTTTGAGCGAATATAT
TTCTATGGAAAAATAAAACATCCTGTAGAAAAAGTCTTTGCTAATGATTT
TCCGGCTATTCGACGGGTCTTCAAGGATCCTTTCATGCATTATGTTAGAT
ATCAGGGAAAATCCATTTTGGTTTCAAAAGATACGCCTCTTCTAATGAAT
AAATGGAAATATTACCTTGTCCATTTATGGCAATGGCATTTTTATGTGTG
GGCTCAACCCGGAAGGATCTACATAAACCAATTATTCAAGCATTCCTTCG
GCTTTTTAGGCTATCTTTCAAGTGTGCGACTAAATCTTTCATTGGTACGG
AGTCAAACGCTCGAAAATTCATTTATAATGGATAATGTTATTAAGAAGCT
TGATACATTAGCTCCAATTAGTCCTCTGATTGGATCGTTGGCTAAAATGA
AATTTTGTAGCGCACTAGGACATCCTATTAGTAAGTCGACCTGGACCGAT
TCGTCGGATTTTTCTATTATCGACCGCTTTGCGCGTATATGCAGAAATCT
TTCTCATTATTACAGCGG--------------------------------

-----------TCTAGACCTAGCTGCTGTCGACGTTCCATCTACAAATGG
ATAAGACTTCGCTTT---GGTATTAGTGTATACGAGTTCTT-GAAAATAA
AGGAGCAATAATCAATTTCTTGTTCTATCAAGAAGTTTGTTATTGCTCCT
TTA-TTATTTAGGAGTCGTTTTTCTTACGTAAGTTTTTTTC-------TT
TACTTCAACAAATTCTTCGTT----ATGTAGAAACTTTTATTTTATATAT
GTTCTTCTCAATCTTTTTTGA-----ATTT---TTTTTTTTTTTTTATTT
TACATTTTTTTTAATAAAAGAAGGGAAACCATTATCTTTTATCTTGTATT
TCAT-----CTTAGAAGAAAGAAAAAAGGTGA---------ATGGTTTGA
CATATTTGG------------------TTTGGAATTTTTAAATAGTCTAG
GGGCGG--
----------------GCAACGGGTTTTGGTCCCGCTATTCGGAGGTTCG
AATCCTTCCGTCCCAGAGCATACCCAACTCGCTATAATATTCATATATAC
AATGAT---AATATATATCAGAAAAGGATTGCCTTTTTAAAATTAAATAA
AATTAA----------------ACAT-------TCTTAAGAGTATAATAT
TGGAAAAATTGGATTATTATTCTTAATACTATTATTATTCTTAATACTAT
AATTCTTTTCTGAATCATATCGATTTCACAAATTGAATCGTATTCAAATA
ACACACAGAGGGAATTGAATCTATTTGTCATCCCTAAATGTTAAA-----
-TATTTAACATAGAATATCTAGAATTCATTTCAGTAACAATTGTTTAGTC
TATCTGATAAATAC----GAGG-------GTCCCATATTATTGTTTTTGG
GGTTCTGGTTTTATCAATTTATCAAATAGTATGAAAAAATAACAACCCTC
CCTTACATCAGTCTTTATCCACTATTTCACTAAAAAAATAAATAATTTTT
TTTATATTA-------TATGTTTTTTAT--------------------T-
-T---------CTATAACATTAAAAAA-----------------------
----------------------------AAAT----TTTTAATATTAAC-
--------------------TTAATAAATATAGAAATAAAATGGAATTGA
AATATTCC--------ATTTTAAGATTAATA-------------------

------------------------------A---------------ATAT
ATGTATATGGTATTAAATTGAATTCTTTCTGAGACTTCGTCAGAAACCAG
CCATTCATATTTCATATAGAAATAAAAG-------------------GTA
TAGATTATTATGTACCAGCCGAACCAATGACTATTCGTGATTCCATAATT
GAATCAATTACATACTGGTTCCAATCTAAAGGAATGTTATGGTAAAACTT
CGTTT--------------------TAAAGCAAGTGTTGGATCCAATGCT
GGTGTTAAAGATTATAAATTGACTTATTACACTCCTGAATATGAAACAAA
GGATACCGATATCTTGGCAGCATTTCAAGTAACTCCTCAACCCGGAGTTC
CACCTGAAGAAGCAGGGGCCGCGGTAGCTGCTGAATCTTCTACTGGTACA
TGGACAACTGTATGGACTGACGGGCTTACCAGTCTTGATCGTTACAAAGG
TCGATGCTACCACATCGAGCCCGTTGCTGGAGAAGAAAGTCAATTTATGG
CTTATGTAGCTTACCCCTTAGACCTTTTTAAAGAAGGTTCTGTTACTAAC
ATATTTACTTCCATTGTGGGTAATGTATTTGGGTTCAAGGCCCTGCGCGC
TCTACGTCTGGAGGATTTGCGAATCCCCCCTGCTTATTCTAAAACTTTCC
AAGGCCCGCCTCATGGCATCCAGGTTGAGAGAGATAAATTTAACAAGTAT
GGCCGCCCCCTATTGGGATGTACTATTAAACCGAAATTGGGGTTATCCGC
TAAGAATTACGGTAGAGCCGTTTATGAATGTCTTCGCGGTGGACTTGATT
TTACCAAAGATGATGAGAACTTGAATTCCCAACCGTTTATACGTTGGAGA
GACCGTTTCTTATTTTGTGCCGAAGCACTTTATAAAGCACAGGCTGAAAC
TGGTGAAATCAAAGGGCATTACTTGAATGCTACGGCAGGTACATGCGAAG
AAATGATGAAAAGGGCTATATTTGCCAGAGAATTGGGAGTTCCTATCGTA
ATGCACGATTACTTAACGGGGGGATTCACTGCAAATACTAGCTTGGCTCA
TTATTGCCGAGATAATGGTCTGCTTCTTCACATCCACCGTGCAATGCATG
CCGTTATTGATAGACAGAAAAATCATGGTATGCACTTTCGTGTACTAGCT
AAAGCGTTACGTCTGTCTGGTGGAGATCATATTCACGCTGGTACTGTAGT
GGGTAAACTTGAAGGGGAAAGAGACATCACTTTAGGCTTTGTTGATTTAC
TACGTGATGATTTTGTTGAGAAAGATCGAAGCCGTGGTATTTATTTCACT
CAAGATTGGGTCTCTCTACCAGGTGTTATACCCGTGGCTTCAGGGGGTAT
TCATGTTTGGCATATGCCTGCTCTGACCGAGATCTTTGGAGACGATGCCG
TACTACAATTCGGCGGAGGAACTTTAGGGCACCCTTGGGGAAATGCACCC
GGTGCCGTAGCTAATCGAGTAGCTCTAGAAGCATGTGTACAAGCTCGTAA
TGAGGGACGTGATCTTGCTCGTGAAGGTAATGAAATTATCCGTGAGGCTA
GTAAATGGAGTGCTGAACTAACTGCTGCTTGTGAAGTATGGAAGGAGATT
AAATTTGAATTCCCAGCAATGGATACTTTGTAA-----------------
---TTAA-TTGGAT--GAGCCTTGGTATGGAACCTACCAAGTGATAACTT
TCAAATTCAGAGAAACCCTGGAATTAAAAACGGGCAATCCTGAGCCAAAT
CCTGTTTTCTGAAAACAAACAAAGGTTCAGAAAGCGATAATAAA-AA--A
GGATAGATAGGGATAGGTGCAGAGACTCAATGGAAGTTGTTCTAACAAAT
GG----AGTTGGCCACGATGCGTT------AGTAAAGGACT-CCTTCCAT
CGAAACTCCAGAAAGTATGAAGAATAAACGTATATATA------CGTACT
GAAATACTATCTCCAAACCAAATGATTAATGACGACCCGAATCTTTTTTT
TTTTTTATTTATATAAAA-------------------------------A
ATGAAAGAATTGTTGT----------GAATCGATTCCAA-GTG-AAAACA
GAATCGAATATTCATTGATCAAATCATTTACTCCATCGTAATCTGAT---
-AGATCTTTTGAAGAATTGAT-----T-AATCGGACGAGAATAAAGATAG
AGTCCCGTTCTACATGTCAA------------TATCGACAACAATGCAAT
TTATAGTAAAAGGAAAATCCGTCGACTTTA-GAAATCGTGAGGGTTCAAG
TCCCTCTATCCCCAAAAAGTC-CCATTGGATTCCCTAATTATTTATC---
-------CTATGCTCTCATTTCGTTAACGGTTCAAAATTCGTTATGTTTC
TCATTCATTCTACTCTTTTACTTTACAAATGGTC------TGAGCGGAAA
TTT--------TTTTCTTTTCACAA-----GCC--------TTGTGATAT
ATAGGATACACGTACAAATGAACATCGTTGGGCACGT----AACCCCGAT
TGTAAATTGT-------AATGATT-AACAATACATATTATTACTTGGACT
GTACTGAAACTTACA----AAGTC------TTCTTTTTG-AAGATCCAAG
AAATT----CCACCAAGACCTGGATAAGGCTTTGTAATC----CCCTTTT
CGTCTTTTTCATTGACATAGAACCAAGTACTCTATTA------AAATGAG
GATGGTGCGTCGTG-----AATGGTCGGGATAGCTCAGCTGGTAGAGCAG
AGACTGAATA--

---AAG
GATCATTGTTGAAACCCTGCAAAAGCAGAATGACCCGCGAACTCGTAAAA
TACAAACCCGGGGGCGAGGGG---CAACCCGGAC--CCC-----------
TTTGGCG--GGGGGTG---CAGCTCGGGCGTTGCTGGTCGATGCCCGCTG
CYGCCGCCTTCCCT-GCCG-CACAAAYGAACCCCGGCGCAAACCGCGCCA
--AGGAACTCAAACGAATGAAG-CGTATGCCCCGGCGCCCCAGAGATGGT
GCGCGTCGGGGTGTTGCG--TCGTAT--TCTATATGTCAAAACGACTCTC
GGCAACGGATATCTCGGCTCTCGCATCGATGAAGAACGTAGCGAAATGCG
ATACTTGGTGTGAATTGCAGAATCCCGTGAACCATCGAGTCTTTGAACGC
AAGTTGCGCCCGAAGCCA--TTAGGCCGAGGGCACGTCTGCCTGGGCGTC
ACACAACGTTGCCCCCCAA-CCCCAAACCTCGACCTCGAG--G-------
-----------AGGGACGGGGAGCGGATGCTGGCCTCCCGTGTGCCATGG
CTCG--CGGTTGGCCTAAAT--ACGAGCACTCGGCGACGAGCGCCGCGGC
AATCGGTGGTTGTCCAACCCTCG-GTGCCACGCTGCGCGCGCAAGTCGCT
CATCTGTGCTCATCGGCC---CCTA-ATGCGCC---GCACACGCGGCGTC
CACAACGCGACCCCAGGTCAGGCGGGGCTACCCGCTGAGTTTAA------

>Rhamnella_franguloides

------------------------CACGAGTATTCTAATTGGAATAGTTT
TAT---------------------------TACTACAAAAAAATCG----
--ATTTTTTCAAAAACTAATCCACGATTATTCTTGTTCCTATATAATTCT
CATGTTTGTGAATACGAATCCATCTTACT----TTTTCTCCGCAACCAAT
TTTCTCGTTTACGATTAACATCTTCTGGGGGCTTTTTTGAGCGAATATAT
TTCTATGGAAAAATAAAACATCCTGTAGAAGAAGTCTTTGCTAATGATTT
TCCGGCTATTCGACGGGTCTTCAAGGATCCTTTCATGCATTATATTAGAT
ATCAGGGAAAATCCATTTTGGTTTCAAAAGATACGCCTCTTCTAATGAAT
AAATGGAAATATTACCTTGTCCATTTATGGCAATGGCATTTTTATGTGTG
GGCTCAACCTGGAAGGATCTACATAAACCAATTATTCAAGCATTCCTTCG
GCTTTTTGGGCTATCTTTCAAGTGTGCGACTAAATCTTTCATTGGTACGG
AGTCAAACGCTCGAAAATTCATTTATAATGGATAATGTTATTAAGAAGCT
TGATACATTAGCTCCAATTAGTCCTCTGATTGGATCGTTGGCTAAAATGA
AATTTTGTAGCGCACTAGGACATCCTATTAGTAAGTCGACCTGGACCGAT
TCGTCGGATTTTTCTATTATCGACCGCTTTGCGCGTATATGCAGAA----

-----------------------------TGCTATAATATTCATATATAC
AATGAT---AATATATATCAGAAAAAGATTGCCCTTTTAAAATTTAATAA
AATGAA----------------ACATTCAACATTCTTAAGAGTATAATAT
TGGAAAAATTG------------------GATTATTATTCATAATACTAG
AATTCTTTTCTGAATCATATCAATTTCATAAATTGAATCGTATTCAAATA
ACACATAGAGGGAATTGAATCTATTTGTCATCCCTAAATGTTAAA-----
-TATTTAACATAGAATATCTAGAATTCATTTCAGTAACAATTGTTTAGTC
TATCTGATAAATAT----GAGG-------GTCCCATATTATTGTTTTTGG
GATTCTGGTTTTATC--------AAATAGTATGAAAAAATAACAACCCTC
CCTTACATCAGTCTTTATCCATTTTTTCACTAAAAAAAGAAAATTTCAAT
TTAACTTAATAAAAATAAAGAAA---------------------------
-----------------------TAAT-----------------------
------AAATATATAAATAAAA------AAG-------------AAAAT-
--------------------GGAATTGAAATATTCCATTTTAAGATTAAT

AAATATAT--------GTTTTAAGATTAATA-------------------

------------------------------T---------------ATAT
ATGTATATGGTATTAAATTGAATTCTTTCTGAGACTTCGCCAGAAACCAG
CCATTCATATTTCATATAGAAATAAAAG-------------------GTA
TAGATTAGTATGTACCAGCCGAACCAATGACTATTCGTGATTCCATAATT
GAATCAATTACATACTGGTTCCAATCTAAAGGAATGTTATGGTAAAACTT
CGTTT---
GGTGTTAAAGANTATAAATTGACTTATTACACTCCTGACTATGAAACCAA
AGATACCGATATCTTGGCAGCATTTCGAGTAACTCCTCAACCCGGAGTTC
CACCTGAAGAAGCAGGGGCCGCGGTAGCTGCTGAATCTTCTACTGGTACA
TGGACAACTGTATGGACTGACGGGCTTACCAGTCTTGATCGTTACAAAGG
TCGATGCTACCACATCGAGCCCGTTGCTGGAGAAGAAACTCAATTTATTG
CTTATGTAGCTTACCCCTTAGACCTTTTTGAAGAAGGTTCTGTTACTAAC
ATGTTTACTTCCATTGTGGGTAATGTATTTGGGTTCAAGGCCCTGCGCGC
TCTACGTCTGGAGGATTTGCGAATCCCCACTGCTTATGTTAAAACTTTCC
AAGGCCCGCCTCATGGCATCCAAGTTGAGAGAGATAAGTTGAACAAGTAC
GGCCGCCCCCTATTGGGATGTACTATTAAACCGAAATTGGGGTTATCCGC
TAAGAATTACGGTAGAGCCGTTTATGAATGTCTTCGCGGTGGACTTGATT
TTACCAAAGATGATGAGAACGTGAATTCCCAACCGTTTATGCGTTGGAGA
GACCGTTTCTTATTTTGTGCCGAAGCACTTTATAAAGCACAGTCTGAAAC
TGGTGAAATCAAAGGGCATTACTTGAATGCTACGGCAGGTACATGCGAAG
AAATGATGAAAAGGGCTATATTTGCCAGAGAATTGGGAGTTCCTATCGTA
ATGCACGATTACTTAACGGGGGGATTCACTGCAAATACTAGCTTGGCTCA
TTATTGCCGAGATAATGGTCTGCTTCTTCACATCCACCGTGCAATGCATG
CCGTTATTGATAGACAGAAAAATCATGGTATGCACTTTCGTGTACTAGCT
AAAGCGTTACGTATGTCTGGTGGAGATCATATTCACGCCGGTACTGTAGT
GGGTAAACTTGAAGGGGAAAGAGACATCACTTTAGGCTTTGTTGATTTAC
TACGTGATGATTTTGTTGAGAAAGATCGAAGCCGTGGTATTTATTTCACT
CAAGATTGGGTCTCTCTACCAGGTGTTTTGCCCGTGGCTTCAGGGGGTAT
TCATGTTTGGCATATGCCTGCTCTGACCGAGATCTTTGGAGACGATTCCG
TCCTACAATTCGGCGGAGGAACTTTAGGTCACCCTTGGGGAAATGCACCC
GGTGCCGTAGCTAATCGAGTAGCTCTAGAAGCATGTGTACAAGCTCGTAA
TGAGGGACGTGATCTTGCTCGTGAAGGTAATGAAATTATCCGTGAGGCTA
GTAAATGGAGTGCTGAACTAGCTGCTGCTTGTGAAGTATGGAAGGAGATT
AAATTTGAATTCCCAGCAATGGATACTTTGTAA-----------------

------------CAAAAAGTC-CCATTGGATTCCCTAATTATTTATC---
-------CTATGCTCTCATTTCGTTAACGGTTCAAAATTCGTTATGTTTC
TCATTCATTCTACTCTTTTACTTTACAAATGGTCTGAGCGGAAATTTTTT
TCT--------TTTC-------------------ACAAGCCTTGTGGTAT
ATAGGATACACGTACAAATGAACATCGTTGGGCACGT----AACCCCGAT
TGTAAATTGT-------AATGATT-AACAATACATATTATTACTTGTACT
GTACTGAAACTTACA----AAGTC------TTCTTTTTG-AAGATCCAAG
AAATT----CCACCAAGACCTGGATAAGGCTTTGTAATC----CCCTTTT
CGTCTTTTTCATTGACATAGAACCAAGTCCTCTATTA------AAATGAG
GATGGTGCGTCGTG-----AATGGTCGGGATAGCTCAGCTGGTAGAGCAG

AGGACTGAAAATC-------------------------------------

---AAG
GATCATTG-TTGAAACCT-GCACAGCAGAACGACCCGTGAACCCGTAAAA
TACAACCGGGG-GGCGAGGAG---CCAATCCGGA--CC----------CC
TCGGCGG--GGAGGCG---TGGCTCGGGCTTCGCTGGCCGATGCCCGCCG
CCGCCGCCTTCCCG-CTGC-ACAAACGAAACCCCGGCGCAAACCGCGCCA
--AGGAACTCGAACGAATCAGG-CACCGCCCCGGCGCCCCAGAGATGGTG
CGCGCTCGGGGTGCTGCG--CCGTATTCTG--TATGTCAAAACGACTCTC
GGCAACGGATATCTCGGCTCTCGCATCGATGAAGAACGTAGCGAAATGCG
ATACTTGGTGTGAATTGCAGAATCCCGTGAACCATCGAGTCTTTGAACGC
AAGTTGCGCCCGAAGCCA--CTAGGCCGAGGGCACGTCTGCCTGGGCGTC
ACACAACGTTGCCCCCCCCACCCAAAACCTCGTACTCGAG--G-------
-----------GGGACGGGGGGGCGGATGCTGGCCTCCCGTGCGCCCCGG
CTCG--CGGCTGGCCCAAAT--GCGAGCACCCGGCGGCGAGCGCCGCGGC
AATCGGTGGTTTTCCAAACCTCG-GTGCCAAGCCGCGCGCGCGAGCCGCC
CATGCGTGCCCGTCGACC---CCCA-GCGCGCC---GCACACGCGGCGTT
CACAACGCGACCCCAGGTCAGGCGGGGCTACCCGCTGAGTTTAA------

>Ventilago_leiocarpa

--------------------------TATTGGGTGAAAGATGCCTCTTCT
TTGCATTTATTACGGCTTTTTCTTCACGAGTATTATAATTGGAATAGTTT
TAT---------------------------TACTCCAAAAAAATCTATTT
CTATTTTTTCAAAAACTAATCCAAGATTATTATTGTTTCTATATAATTCT
CATGTTTTTGAATACGAATCCATCTTACT----TTTTCTCCGTAACCAAT
TTTCTCGTTTACGATTAACATCTTCTGGGGGCTTTTTTGAGCGAATATTT
TTCTATGGAAAAATAAAACATCCTGTAGAAGAAGTCTTTGCTAATGATTT
TCCGGCTATTCGACGGTTCTTCAAGGATTCTTTCATGCATTATGTTCGAT
ATCAAGGAAAATCCATTTTGGTTTCAAAAGATACGCCTCTTCTAATGAAT
AAATGGAAATATTACCTTATCCGTTTATGGCAATGTCATTTTTATGTGTG
GGCTCAACCAGGAAGGATCTCCATAAACCAATTATTCAAGCATTCCTTCG
GCTTTTTGGGCTATCTTTCAAGCGTGCGACTAAATCTTTCATTGGTACGG
AGTCAAATGCTAGAAAATTCATTTATAATGAATAATGCTATGAAGAAGCT
TGATACATTAGTTCCAATTAGTCCTCTGATTGGATCGTTGGCTAAAATGA
AGTTTTGTAGCGCACTAGGACATCCTGTTAGTAAGTCGACCTGGACCGAT
TCGTCGGATTTTTATATTATCGACCGCTTTGCGCGTATATGC--------

---------------------------------GTTCCATCTACAAATGG
ATAAGACTTCTATGATTAGTGTATACGAGTTTTTGAAATGAAGAAAATAA
AGGAGCAATAATAAACCTCTTGGTATATCAAGAAGTTTGGTATTGCTCCT
TTA-TTTTCATAATAAATAATTATTTATTAGTTTTTTGCT-----TAGCA
TAAGCCTTTTTTTCTTTACTTCAACATATTGATACTTTCATTATTTTTGT
TATGTAGTAATTTATATATA-----TATTTTAGTTTATTTTTTT------
---TTTCTT---CGACA------ACTAAAAAATTTTATT--TAAATTATT
TAAAATAGAATAATAAAAAAAAATAAAGATGAATGTT----GTAAATTGA
ATTTGTTTTTTTTTATTATTAATTAATAGATAGAAATACTAATAGTATCG
GGGCGGATGTACCCAAGGGGATTAAGGCAGGGAATTGGGAATCCCC----

-----------------------ACTAAAGCAAGTGTTGGATTCAAGGCT
GGTGTTAAAGATTATAAATTGAATTATTACACTCCTGACTACGAAACCAA
AGATACTGATATCTTGGCAGCATTTCGAGTAACTCCTCAACCCGGAGTTC
CGCCTGAGGAAGCAGGGGCCGCGGTAGCTGCTGAATCTTCTACTGGTACA
TGGACAACTGTATGGACTGACGGGCTTACCAGTCTTGATCGTTACAAAGG
TCGATGCTACCACATCGAGCCCGTTGCTGGAGAAGAAAATCAATATATTG
CTTATGTAGCTTACCCCTTAGACCTTTTTGAAGAAGGTTCTGTTACTAAC
ATGTTTACTTCCATTGTAGGTAATGTATTTGGGTTCAAGGCCCTGCGCGC
GCTACGTTTGGAGGATTTGCGAGTCCCTCCGGCTTATTCTAAAACTTTCC
AAGGCCCGCCTCATGGCATCCAAGTTGAAAGAGATAAATTGAACAAGTAT
GGCCGCCCCCTATTGGGATGTACTATTAAACCTAAATTGGGGTTATCCGC
TAAGAATTACGGTAGAGCCGTTTATGAATGTCTTC---------------

------TTAATTAGTGGAGCTTGGTATGGAAACCTACCAAGTGATAACTT
TCAAATTCAGAGAAACCCTGGAATTAAAAACGGGCAATCCTGAGCCAAAT
CCTGTTTTCTGAAAACAAACAAAGGTTCAGATAGCGATGAAAAAAAAAAA
GGATGAATGGGGATGGGTGCAAAAACTCCTTGGAAGTTGTTCTAACAAAT
GG----AGTTGGCGGCGATCCATT------ATTAAAGGACT-CCTTCCAT
CGAAACTCCCAAAAGGATGAAAAATAAACCTTTATTTCCTTTCT------
GAAATAATATCTCCAAACCAAATGATTAATGACAACCCGAATTTTTTTTT
TT---TTTTTTTTTAAAA-------------------------------A
AAGAAAGAATTGTTGT----------GAATCGATTCCAAGTTG-AAAAAA
GAATCGAATATTCATTGATCAAATCATTTACTCCATCGTAATCTGAT---
-NGATCTTTTGAAGAATTGAT-----T-AATCGGACGAGAATAAAGATAG
AGTCCCATTTTACATGTTAA------------TATCGACAACAATGATAT
TTATAGTAAGAGGAAAATCCGTCGACTTTA-TAAATCGTGAGGGTTCAAG
TCCCTCTATCCCCAAAAAGTC-CTATAAGATTCCCTAATTATTTATC---
-------CTATGCTCTCATTTCGTTAGCGGTTCAAAATTCGTTATGTTTC
TCATTCATTTTACTTTTTTACTTTACAAATGGTTTGAGCGGAAATTTTTT
TCT--------TTTC-------------------ACAAGCCTTGTGATAT
ATAGGATACACGTACAAATAAACATCGTTGAGCACGT----AACAACGAT
TGAAAATTGT-------AATGATT-AACAATACATATTATTTTATTACTC
GTACTGTACTAAAAC----TTACAAA-GTCTTCTTTTTGAAGATTCCAAG
AAATT----CCACCAAGGCCCGGATAAGACTTTGTAATCGCCCCCCTTTT

CGTCTTTTTAATTGACATAGAACCATGTCCTCTATTA------AAATGAG
TCTGGTGCGTCGTG-----AATGGTCGGGATAGCTCAGTGTAGAGCAGAC
TGAA--

-CACACTGTTGCCCCCCTAATCCCTTGTCACTCGTTCT-----------G
TAGTGCATGTGGAGTAAAAAGGGCGTATATTGGCCTCCCGTGCGATATGG
ACTTCATGGTTGGCCCAAAC--TCAAGTCCCTTGTCACGTGCATCGTGAC
AAAAGGTGGTTGTCGATCAGTCGGTGCCCCGTCACTTGATGCCGGACACG
TAAAGGGACTCCCAGCGA--CCCCAATGCATCG---ATAGTGTAGGTGCT
TTGAAAG---

>Maesopsis_eminii

--TCTTCT
TTGCATTTATTACGGCTTTTTCTTCACGAGTATTCTAATTGGAATAGTTT
TCT---------------------------TACTACAAAGAAATCTATTT
CTATTTTTTCAAAAACTAATCCAAGATTATTCTTGTTCCTATATAATTCT
CATGCTTGTGAATACGAATCTATCTTACT----TTTTCTCCGCAACCAAG
TTTCTCGTTTACGATTAACATCTTCTGGGGGCTTTTTTGAGCGAATATAT
TTCTATGGAAAAATAAAACATCCTGTAGAAGAAGTCTTTGCTAATGATTT
TCCGGCTATTCGCCGGCTCTTCAAGGATCCTTTCATGCATTATGTTAGAT
ATCAAGGAAAATCCCTTTTGGTTTCAAAAGATACGCCTCTTCTAATGAAT
AAATGGAAATATTACCTTGTCCATTTATGGCAATGTCATTTTTATGTGTG
GGCTCAACCTGGAAAGATCTACATAAACCAATTATTCAAGCATTCCTTCG
GCTTTTTGGGCTATCTTTCAAGCGTGCGACTAAATCTTTCATTGGTACGG
AGTCAAACGCTCGAAAATTCATTTATAATGGATAATGCTATTAAGAAGCT
TGATACATTAGCTCCAATTAGTCCTCTGATTGGATCGTTGGCTAAAATGA
AATTTTGTAGCGCACTAGGACATCCTGTTAGTAAGTCGACCTGGACCGAT
TTGTCGGATTTTTCTATTATCGACCGCTTTGCGCGTATATGCAGAAATCT
TTCTCATTATTACAGCGGATCCTCAAAAAAAAGGAGTTTGTATCGAATAA
AAT---

----------------------------AGCAAGTGTGGGATTCAAAGCT
GGTGTTAAAGATTATAAATTGACTTATTACACTCCTGACTATGAAACCAA
AGATACTGATATCTTGGCAGCATTTCGAGTAACTCCTCAACCTGGAGTTC
CACCCGAGGAAGCAGGGGCCGCGGTAGCTGCTGAATCTTCTACTGGTACA
TGGACAACTGTATGGACTGACGGGCTTACCAGTCTTGATCGTTACAAAGG
TCGATGCTACTACATCGAGCCCGTTGCTGGAGAAGAAAATCAATTTATTG
CTTATGTAGCTTACCCCTTAGACCTTTTTGAAGAAGGTTCTGTTACTAAC
ATGTTTACTTCCATTGTGGGTAATGTATTTGGGTTCAAGGCCCTGCGCGC
TCTACGTCTGGAGGATTTGCGAATCCCTACTTCTTATTCTAAAACTTTCC
AAGGCCCGCCTCATGGCATCCAAGTTGAGAGAGATAAGTTGAACAAGTAT
GGCCGCCCCTTATTGGGATGTACTATTAAACCTAAATTGGGGTTATCCGC
TAAGAATTACGGTAGAGCCGTTTATGAATGTCTTCGCGGTGGACTTGATT
TTACCAAAGATGATGAGAACGTGAATTCCCAACCATTTATGCGTTGGAGA
GACCGTTNCTTATTTTGTGCCGAAGCAATTTATAAAGCACAGNCCGAACT
NNGTGAAATCAAANGGCATTACTTGAATGCTACGGCAGGTACATGGGAAG
AAATGATCAAAAGGGCTGTATGTGCCAGAGAATTGGGAGTTCCTATCGTA
ATGCACGATTACTTAACGGGGGGATTCACTGCAAATACTAGCTTGGCTCA
TTATTGCCGAGATAATGGTCTGCTTCTTCACATCCACCGTGCAATGCATG
CAGTTATTGATAGACAGAAAAATCATGGTATGCACTTTCGTGTACTAGCT
AAAGCGTTACGTCTGTCTGGTGGAGATCATATTCACGCTGGTACTGTAGT
GGGTAAACTTGAAGGGGAAAGAGACATCACTTTAGGCTTTGTTGATTTAC
TACGTGATGATTTTATTGAGAAAGATCGAAGCCGCGGTATTTATTTCACT
CAAGATTGGGTCTCTCTACCAGGTGTTCTGCCCGTGGCTTCAGGGGGTAT
TCACGTTTGGCATATGCCTGCTCTGACCGAGATCTTTGGAGACGATTCCG
TACTCCAATTTGGCGGAGGAACTTTAGGGCACCCTTGGGGAAATGCACCC
GGTGCCGTAGCTAATCGAGTAGCTCTAGAAGCATGTGTACAAGCTCGTAA
TGAGGGACGTGATCTTGCGCGTGAAGGTAATGAAATTATCCGTGAGGCTA
GTAAATGGAGTCCTGAACTAGCTGCTGCTTGTGAAGTATGGAAGGAGATT
AAATTTGAATTCCCGGCAATGGATACTTTGTAA-----------------
---TTAATTGGATG--AGCCTTGGTATGGAAACCTACCAAGTGATAACTT
TCAAATTCAGAGAAACCCTGGAATTAAAAACGGGCAATCCTGAGCCAAAT
CCTGTTTTCTGAAAACAAACAAAGGTTCAGAAAGCGATAATAAAAAA--A
GGATAGATATGGATAGGTGCAGAGACTCAATGGAAGTTGTTCTAACAAAT
GG----AGTTGGCTACGATGCGTT------AGTAAAGGACT-CCTTCCAT
CGAAACTCCAGAAAGGATGAAGAATAAACCTATATATACGTACTGAAA--
----TACTATCTCCAAACCGAATGATTAATGACGACCCGAATCTTTTTTT
TTATTTACAACAAAAATG-------------------------------A
AAAAAAGAATTGTTGT----------GAATCAATTCCAAGTTG-AAAAAA
GAATCGAATATTA---------ATCATTTACTCTATCGAAATCTGAT---
-AGATCTTTTGAAGAATCGAT-----T-AATCGGACGAGAATAAAGATAG
AGTCCCGTTCTACATGTCAA------------TATCGACAATAATGCAAT
TTATAGTAAAAGGAAAATCCGTCGACTTTA-GAAATCGTGAGGGTTCAAG
TCCCTCTATCCCCAAAAAGTC-CCAGTGGATTCCCTAATTATTTATC---
-------CTATGCTCTCATTTCGTTAACGGTTCAAAATTCGTTATGTTTC
TCATTCATTCTACTCTTTTACTTTACAAAGGGTC------TGAGCGGAAA
TCT--------TTTTCGTATCACAA-----GCC--------TTGTGATAT
ATAGGCTACACGTACAAATGAACATCGTTGGGCACGT----AACCCCGAT
TGTAAATTGT-------AATGATT-AACAATACATATTATTACTCGTAGT

GTACTGAAACTTACA----AAGTCTT-GTCTTCTTTTTG-AAGATCCAAG
AAATT----CCACCAAGACCAGGATAAGGCTTTGTAATC----CCCCTTT
CGTCTTTTTCATTGACATAGAACCAAGTCTTCTATTA------AAATGAG
GATGGTGCGTCGTG-----AATGGTCGGGATAGCTCAGCTGGTAGAGCAG
AGACTGAA--

>Scutia_buxifolia

------------CGGCTTTTTCTTCACGAGTATTCTAATTGGAATAGTTT
TAT---------------------------TACTACAAAAAAATCGATTT
CTATTTTTTCAAAAACTAATCCAAGATTATTCTTGTTCTTATATAATTCT
CATGCTTGTGAATACGAATCCATCTTACT----TTTTCTCCGCAACCAAT
TTTCTCGTTTACGATTAACATCTTCTGGGGGCTTTTTCGAGCGAATATAT
TTCTATGGAAAAATAAAACATCCTGTAGAAAAAGTCTTTGCTAATGATTT
TCCGGCTATTCGACCGTTCTTCAAGGATCCTTTCATGCATTATGTTAGAT
ATCAGGGAAAATCCATTTTGGTTTCAAAAGATACGCCTCTTCTAATGAAT
AAATGGAAATATTACCTTGTCCGTTTATGGCAATGGCATTTTTATGTGTG
GGCTCAACCCGGAAGGATCTACATAAACCAATTATTCAAGCATTCCTTCG
GCTTTTTGGGCTATCTTTCAAGTGTGCGACTAAATCTTTCATTGGTACGG
AGTCAAACGCTCGAAAATTCATTTATAATGGATAATGCTATTAAGAAGCT
TGATACATTAGCTCCAATTAGTCCTCTGATTGGATCGTTGGCTAAAATGA
AATTTTGTAGCGCACTAGGACATCCTGTTAGTAAGTCGACCTGGACCGAT
TCGTCGGATTTTTCTATTATCGACCGTTTTGCGCGTATATGCAGAAATCT

-----------------------------------TTAAGAGTATAATAT
TGGAAAAA------------------TTGGATTATTATTCTTAATACTAT
AATTCTTTTCTGAATCATATCAATTTTACAAATTGAATCGTATTCAAATA
ACACACAGAGGGAATTGAATCTATTTGTCATCTCTAAATGTTAATGTTAA
ATATTTAACATAGAATATCTAGAATTCATTTCAGTAACAATTGTTTAGTC
TATCTGATAGATAT----GAGG-------GTCCCATATTATTGTTATTGT

TTTTGGGA--TTTTGTTTTTATCAAACAGTATGAAAAAATAACAACCCTC
CCTTACATCAGTCTTTATCCACTATTTCACTAAAAAAATAAATTATTTAT
TTTATTCGTGTTATT-TATCATTTTTTTT----------TA--TTCTAT
GTTTTTTATTTCTATAATATTAATATTA--AA------------------
--------------------------AAAAAA----ATTCAATTAAAAT-
--------------------ATTAACTTAATATAAATAAAATAGAATTGA
AATATTCC--------ATTTTAAGATTAATA-------------------

------------------------------A---------------ATAT
ATGTATATGGTATTAAATTGAATTCTTTCTGAGACTTCGTCAGAAACTAG
TCATTCATATTTCATATAGAAATAAAAG-------------------GTA
TAGATTATTAAGTACCGGCCGAACCAATGACTATTCGTGATTCCATAATT
GAATCAATTACAT-------------------------------------

--------------------------------TCCTACATATGATACCAA
AGATACCGATATCTTGGCAGCATTCCGAGTATCTCCTCAACCCGGAGTTC
CACCTGAGGAAGCAGGAGCCGTGGTAGATGCTGAATCTTCTACTGGTACA
TGGACAACTGTATGGACTGACGGGCTTACCAGTCTTGATCGTTACAAAGG
TCGATGCTACCACATCGAGCCCGTTGCTGGAGAAGAAACTCAATTTATTG
CTTATGTAGCTTACCCCTTAGACCTTTTTGAAGAAGGTTCTGTTACTAAC
ATGTTTACTTCCATTGTGGGTAATGTATTTGGGTTCAAGGCCCTGCGCGC
TCTACGTCTGGAGGATTTGCGAATCCCCACTGCTTATGTTAAAACTTTCC
AAGGCCCGCCTCATGGCATCCAAGTTGAGAGAGATAAGTTGAACAAGTAT
GGCCCCCCCCTATTGGGATGCACTATTAAACCTAAATTGGGGTTATCCGC
TAAGAATTACGGTAGAGCCGTTTATGAATGTCTTCGCGGTGGACTTGATT
TTACCAAAGATGATGAGAACGTGAATTCCCAACCGTTTATGCGTTGGAGA
GACCGTTTCGTATTTTGTGCCGAAGCAATTTATAAAGCACAGGCCGAAAC
TGGTGAAATCAAAGGGCATTACTTGAATGCTACGGCAGGTACATGCGAAG
AAATGATGAAAAGGGCTGTATTTGCCAGAGAATTGGGAGTTCCTATCGTA
ATGCACGATTACTTAACGGGGGGATTCACTGCAAATACTACCTTGGCTCA
TTATTGCCGAGATAATGGTCTGCTTCTTCACATCCACCGTGCAATGCATG
CAGTTATTGATAGACAGAAAAATCATGGTATGCACTTTCGTGTACTAGCT
AAAGCTTTACGTATGTCTGGTGGAGATCATATTCACGCTGGTACTGTAGT
GGGTAAACTTGAAGGGGAAAGAGACATCACTTTAGGCTTTGTTGATTTAC
TACGTGATGATTTTATTGAGAAAGATCGAAGCCGTGGTATTTATTTCACT
CAAGATTGGGTCTCTCTACCAGGTGTTCTGCCCGTGGCTTCAGGGGGTAT
TCACGTTTGGCATATGCCTGCTCTGACCGAGATCTTTGGAGACGATTCCG
TACTACAATTCGGCGGAGGAACTTTAGGGCACCCTTGGGGAAATGCACCC
GGTGCCGTAGCTAATCGAGTAGCTCTAGAAGCATGTGTACAAGCTCGTAA
TGAGGGACGTGATCTTGCTCGTGAAGGTAATGAAATTATCCGTGAGGCTA
GTAAATGGAGTCCTGAATTAGCTGCTGCTTGTGAAGTATGGAAGGAGATT
AAATTTGAATTCGAAGCAATGGATACTTTGTAA-----------------

---------------------------------------TATTTATC---
-------CTATGCTCTCATTTCGTTAACAGTTCAAAATTCGTTATGTTTC
TCAGTCATTCTACTCTTTTACTTTACAAATGGTCTGAGCGGAAATTTTTT
TCT--------TTTCACAATC-------------ACAAGCCTTGTGATAT

ATAGGATACACGTACAAATGAGCATCGTTGGGCACGT----AACCCCGAT
TGTAAATTGT-------AATGATT-AACAATACATATTATTACTTGTACT
GTACTGAAACTTACA----AAGTC------TTCTTTTTG-AAGATCCAAT
AAATT----CCACCAAGACCTGGATAAGGCTTTGTAATC----CCCTTTT
CGTCTTTTTCATTGACATAGAACCAAGTCCTCTATTA------AAATGAG
GATGGTGCGTCGTG-----AATGGTCGGGATAGCTCAGCTGGTAGAGCAG
AGGACTGAAAATC-------------------------------------

---G
GATCATTGTCGAAACC-CTGCACAGCAGAACGACCCGCGAACTTGTAAAA
TACAACTTGGGGGCGAGGGGC---CC-AGCGGCC--CCG-GACCTCCTTG
GTTTGGG--GGGGTGC---CAGCTCGGGCATYGCTGGTCGATGCTCGTTG
CTGTTGCCTTCCT-TGCCG-CACTAACGAACCCCGGCGCAAACCGCGCCA
--AGGAAC-TTGAACGA--ATCAGCACGCCCCGGTGCCCCAGAAATGGTG
CGCGTTCGGGGCGTTGCG--TCGTAT--TCTTTATGTCTAAACGACTCTC
GGCAACGGATATCTCGGCTCTCGCATCGATGAAGAACGTAGCGAAATGCG
ATACTTGGTGTGAATTGCAGAATCCCGTGAACCATCGAGTCTTTGAACGC
AAGTTGCGCCCGAAGCCA--TTAGGCCGAGGGCACGTCTGCCTGGGCGTC
ACACAACGTTGCCCCCCCAACCCCAAACCTCGACTTCGAGGAG-------
-----------GAG----GGGGGCGGATGCTGGCCTCCCGTGTGCCACGG
CTCG--CGGTTGGCCCAAAT--GCGAGTACTCGGCGACGAGCGCCGCGGC
TATCGGTGGTTGTCCAACCCTCG-GTGCCATGCTGCGCGCGCGACTCGCT
CATTGCTACTCGATGACC---CCCA-ACGCGTCG----------------

>Sageretia_thea

--------------------------TATTGGGTGAAAGATGCCTCTTCT
TTGCATTTATTACGGCTTTTTCTTCACGAGTATTCTAATTGGAATAGTTT
TAT---------------------------TACTACAAAAAAATCTATTT
CTATTTTTTCAAAAAGTAATCCAAGATTATTCTTGTTCCTATATAATTCT
CATGCTTGTGAATACGAATCCATCTTACT----TTTTCTCCGCAACCAAT
TTTCTCGTTTACGATTAACATCTTCTGGGGGCTTTTTTGAGCGAATATAT
TTCTATGGAAAAATAAAACATCCTGTAGAAGAAGTCTTTGCTAATGAGTT
TCCGGCTATTCGACGGGTCTTCAAGGATCCTTTCATGCATTATGTTAGAT
ATCAGGGAAAATCCATTTTGGTTTCAAAAGATACGCCTCTTCTAATGAAT
AAATGGAAATATTACCTTGTCCGTTTATGGCAATGGCATTTTTATGTGTG
GGCTCAACCCGGAAGAATCTACATAAACCAATTATTCAAGCATTCCTTCG
GCTTTTTGGGCTATCTTTCAAGTGTGCGACTAAATCTTTCATTGGTACGG
AGTCAAACGCTCGAAAATTCATTTATAATGGATAATGCTATTAAGAAGCT
TGATACATTAGCTCCAATTAGTCCTCTGATTGGATCGTTGGCTAAAATGA
AATTTTGTAGCGCACTAGGACATCCTGTTAGTAAGTCGACCTGGACCGAT
TCGTCGGATTTTTCTATTATCGACCGCTTTGCGCGTATATGCAGAAATCT
TTCTCATTATTACAGCGGGTCC----------------------------

---TACAAATGG
ATAAGACTTCGCTTT---GGTATTAGTGTATACGAGTTCTT-GAAAATAA
AGGAGCAATAATCAATTTCTTGTTCTATCAAGAAGTTTTTATTGCTCCT
TTA-TTATTTAGAAGTATTTTTTCTTACGTAAGTTTTTTTC-------TT
TACTTCAACAAATTCTTCGTT----ATGTAGAAACTTTTTTTT-ATATAT
GTTCTTCTCAATCTTTTTTGA-----ATTT---T----------TGATTT
TCCATTAAAAAAAAAAAAAGAAAGGAAACCATTATCTTTTATCTTGTATT
TCAT-----CTTAGAAGAAAGAAAAAAGGTGA---------ATGGTTTGA
CATATTTTG------------------TTTGGAATTTGTAAATAGTCT--

--------------------------GATTGCCCTTTTAAAAT-------
--GAAA----------------CATTTAAACATTCTTAAGAGTATAATAT
TGGAAAAATT------------------GGATTCTTATTCTTAATACTAT
AATTATTTTCTGAATCATATCAATTTCACAAATTGAATCGTATTCAAATA
ACACACAGAGGGAATTGAATCTATTTGTCATCCCTAAATGTTAAA-----

-TATTTAACATAGAATATCTAGAATTCATTTCAGTAACAATTGTTTAGTC
TATCTGATAGATAT----GAGG-------GTCCCATATTATTGTTTTTGG
GATTCTGGTTTTATC--------AAACAGTATGAAAAAATAACAAACCTC
CCTTACATCGGTCTTTATCCACTATTTCACTAAATATTTCACTAAAAAAA
GAAAGAATTTCTTTTTTTTATTCGTGTTAT----------GT--ATATG-
-TTTTTTATTTCTATAATATTAAAAATATTAACTTAATAAATAATAAATA
TTAAATATATATAAATATAGAAATAAAA---------------TATATA-
------------------AATATAGAAATAAAAAAAAAAAATGGAATTGA
AATAGTCC--------ATTTTAAGATTAATA-------------------

------------------------------AATATATGTATATGGTATTA
AAGTATATGGTATTGAATTGAATTCTTTCTGAGACTTCGTCAGAAACTAG
CCATTCATATTTCATATAGAAATAAAAG-------------------ATA
TAGATT--

-------------------------------AAGTGTTGGATTCAAGGCT
GGTGTTAAAGATTATAAATTGACTTATTACACTCCTGAATATGAAACCAA
AGATACCGATATCTTGGCAGCATTTCGAGTAACTCCTCAACCCGGAGTTC
CACCTGAGGAAGCAGGGGCCGCGGTAGCTGCTGAATCTTCTACTGGTACA
TGGACAACTGTATGGACTGACGGGCTTACCAGTCTTGATCGTTACAAAGG
TCGATGCTACCACATCGAGCCAGTTGCTGGAGAAGAAAGTCAATTTATTG
CTTATGTAGCTTACCCCTTAGACCTTTTTGAAGAAGGTTCTGTTACTAAC
ATGTTTACTTCCATTGTGGGTAATGTATTTGGGTTCAAGGCCCTGCGTGC
TCTACGTCTGGAGGATTTGCGAATCCCACTTGCTTATACTAAAACTTTCC
AAGGCCCGCCTCATGGCATCCAAGTTGAGAGAGATAAGTTGAACAAGTAT
GGCCGCCCCCTATTGGGATGTACTATTAAACCTAAATTGGGGTTATCCGC
TAAGAATTACGGTAGAGCCGTTTATGAATGTCTTCGCGGTGGACTTGATT
TTACCAAAGATGATGAGAACGTGAATTCCCAACCGTTTATGCGTTGGAGA
GACCGTTTCTTATTTTGTGCCGAAGCACTTTATAAAGCACAGGCCGAAAC
TGGTGAAATCAAAGGGCATTACTTGAATGCTACGGCAGGTACATGCGAAG
AAATGATGAAAAGGGCTGTATTTGCCAGAGAATTGGGAGTTCCTATCGTA
ATGCACGATTACTTAACGGGGGGATTCACTGCAAATACCAGTTTGGCTCA
TTATTGCCGAGATAATGGTCTGCTTCTTCACATCCACCGTGCAATGCATG
CCGTTATTGATAGACAGAAAAATCATGGTATGCACTTTCGTGTACTAGCT
AAAGCGTTACGTGTGTCTGGTGGAGATCATATGCACGCTGGTACTGTAGT
GGGTAAACGTGAAGGGNNNNNNNNNNNNNNNNNNNNNNNNNNNNNNNNNN
NNNNNNNNNNNNNNNNNNNNNNNNNNNNNNNNNNNNNNNNNNNNNNNNNN
NNNNNNNNNNNNNNNNNNNNNNNNNNNNNNNNNNNNNNNNNAGGGGGTAT
TCACGTTTGGCATATGCCCGCTCTGACCGAGATCTTTGGAGACGATTCCG
TACTACAATTCGGCGGAGGAACTTTAGGACACCCTTGGGGAAATGCACCT
GGTGCCGTAGCTAATCGAGTAGCTCTAGAAGCATGTGTACAAGCTCGTAA
TGAGGGGCGTGATCTTGCTCGTGAAGGTAATGAAATTATCCGTGAGGCTA
GTAAATGGAGTCCTGAACTAGCTGCTGC----------------------

----------------------TGGTATGGAAACTACCAAGTGATAACTT
TCAAATTCAGAGAAACCCTGGAATTAAAAATGGGCAATCCTGAGCCAAAT
CCTGTTTTCTGAAAACAAACAAAGGTTCAGAAAGCGATAATAAA-AA--G
GGATAGATAGGGATAGGTGCAGAGACTCAATGGAAGTTGTTCTAACAAAT
GG----AGTTGGCCACGATGCGTT------AGTAAAAGACT-CCTTCCAT
CGAAACTCCAGAAAGTATGAAGAATAAACGTATATATACGTATACGTACT
GAAATACTATCTCCAAACCAAATGATTAATGACGACCCAAATCTTTTTT-
----TTATTTATATAAAA-------------------------------A
ATGAAAGAATTGTTGT----------GAATCGATTCCAAGTTG-AAAACA
GAATCGAATATTCATTGATCAAATCATTTACTCCATCGTAATCTGAT---
-AGATCTTTTGAAGAATTGAT-----T-AATCGGACGAGAATAAAGATAG
AGTCCCGTTCTACATGTCAA------------TATCGACAACAATGCAAT
TTATAGTAAAAGGAAAATCCGTCGACTTTA-GAAATCGTGAGGGTTCAAG
TCCCTCTATCCCCAAAAAGTC-CCATTGGGTTCCCTAATTATTTATC---
-------CTATGCTCTCATTTCGTTAACGGTTCAAAATTCGTTATGTTTC

TCATTCATTCTACTCTTTTACTTTACAAATGGTC------TGAGCGGAAA
TTT--------TTTTCTTTTCACAA-----GCC--------TTGTGATAT
ATAGGATACACGTACAAATGAACATCGTTGGGCACGT----AACCCCGAT
TGTAAATTGT-------AATGATT-AACAATACATATTATTACTTG----
-TACTGAAACTTACA----AAGTC------TTCTTTTTG-AAGATCCAAG
AAATT----CCACCAAAACCTGGATAAGGCTTTGTAATC----CCCTTTT
CGTCTTTTTCATTGACATAGAACCAAGTCCTCTATTA------CAATGAG
GATGGTGCGTCGTG-----AATGGTCGGGATAGCTCAGCTGGT-------

---G
GATCATTGTTGAAACC-CTGCACAGCAGAACGACCCGCGAACTTGTAAAA
TATAACTTGGGGGCGAGGGGC---CCTAGTGGCC--TCG-GACCTCCTTT
GGCTTGG--GGGGTGC---CAACTCGGGCATCGCCGGTCGGTGCCCGTTG
CTGTTGCCTTCCC-TGCCG-CACAAACGAACCCCGGCGCAATCCGCGCCA
--AGGAATTTTCAACGA--ATCAGCAAGCCTCGGCGCCCCAGAGATGGTG
CGCGTTTGGGGCTTTGCG--TGGTAT--TCTATATGTCAAAACGACTCTC
GGCAACGGATATCTCGGCTCTCGCATCGATGAAGAACGTAGCGAAATGCG
ATACTTGGTGTGAATTGCAGAATCCCGTGAACCATCGAGTCTTTGAACGC
AAGTTGCGCCCAAAGCCA--TTAGGCCGAGGGCACGTCTGCCTGGGCGTC
ACACAACGTTGCCCCCCCAACCCCAATCCTCAACTTTGAGGAG-------
-----------GACGGCGGGGGGCGGATGCTGGCCTCCCGTGCGCCACGG
TTTG--CGGTTGGCCCAAAT--ACGAGTACTCGGCGACGAGCGCCGCGGC
AATCGGTGGTTGTCCAACCCTCG-GTGCCATGCTGCGCGCGCGAGTCGCT
CTTCATTACTCGACGACC---CCAA-TGCGTCG-----------------

>Berchemiella_yunnanensis

-------TTGGAAAATGTAGGTTATGACAATAAATCTATTTTACGAATTG
TAAAACGTTTCATTACTCGAATGTATCACCAGAATCATTTGATTATTTCC
GCTAACGATTCTACCCAAAATAAATGTTTTGGGTACAACAAGAATTTTTA
TTCTCAAATGATATCAGAGGGATTTGCAGTTATTGTGGAAATTCCATTTT
CCTTACGATTACCACCT---------TCGGGGACAGAAATTGTAAAATAT
TATAATTTACAATCAATTCATTCAATATTTCCTTTTTTAGAGGACAAATT
CCCACGTTTAAATTATGTATCAGATGTACTAATACCCTACCCCATTCATC
TGGAAATCTTGGTTGAAATCCTTCGCTATTGGGTGAAAGATGCCTCTTCT
TTGCATTTATTACGGCTTTTTCTTCACGAGTATTCTAATTGGAATAGTTT
TAT---------------------------TACTACAAAAAAATCTATTT
CTATTTTTTCAAAAACTAATCCAAGATTATTCTTGTTCCTATATAATTCT
CATGCTTGTGAATACGAATCCATCTTACT----TTTTCTCCGCAACCAAT
TTTCTCGTTTACGATTAACATCTTCTGGGGGCTTTTTTGAGCGAATATAT
TTCTATGGAAAAATAAAACATACTGTAGAAGAAGTCTTTGCTACTGATTT
TCCGGCTATTCGACGGGTATTCAAGGATCCTTTCATGCATTATGTTAGAT
ATCAGGGAAAATCTATTTTGGTTTCAAAAGATACGCCTCTTCTAATGAAT
AAATGGAAATATTACCTTGTCCGTTTATGGCAATGGCATTTTTATGTGTG
GGCTCAACCCGGAAGGATCTACATAAACCAATTATTCAAGCATTCCTTCG
GCTTTTTGGGCTATTTTTCAAGTGTGCGACTAAATCTTTCATTGGTACGG
AGTCAAACGCTCGAAAATTCATTTATAATGGATAATGCTATTAAGAAGCT
TGATACATTAGCTCCAATTAGTCCTCTGATTGGATCGTTGGCTAAAATGA
AATTTTGTAGCGCACTAGGACATCCTATCAGTAAGTCGACCTGGACCGAT
TCGTCGGATTTTTCTATTATCGACCGCTTTGCGCGTATATGCAGAAATCT
TTCTCATTATTACAGCGGGTCCTCAAAAAAA-------------------

--------------------------GGAGTAGGACTTCTACTTTTTCCG
ACGGCAATAAAAAATCTTCGCCGTATGTGGGTTTTTCCTAGTGTTTTACT
GTTAAGTATAGTTATGGTTTTTTCAGCTCATATATTTATTCAACAAATAA
ATAATAGTTCTATCTATCTATATGGATGTTCTTGGACCATCAATAATGAT
TTTTCTTTAGAATTTGGCTATTTAATTGATCCCCTTACTTCTCTTATGTT
AATATTAATAACTACTGTTGGAATTATGGTTCTTATTTATAGTGATAATT
ATATGGCTCATGATCGGGGATATTTGAGGTTTTTTGCTTATATGAGTTTT
TCTAATACTTCAATGTTAGGATTGGTTACTAGTTCAAATTTGATACAAAT
TTATTTTTTTTGGGAATTGGTTGGAATGTGTTCTTATCTATTAATAGGCT
TTTGGTTCACACGACCTATTGCGGCGAATGCTTGTCAAAAAGCGTTTGTA
ACTAACCGCGTAGGGGATTTTGGTTTATTATTAGGAATTTTAGGTTTTTA
TTGGATAACGGGTAGTTTAGAATTTCGGGATTTGTTTGAAATATTCAATA
ACGTGGTTTATAATAATGAAGTTAATTTTTTATTTGTTACTTTGTGTGCC
TTTCTATTATTTGCCGGTGCAGTTGCTAAATCTGCCCAATTTCCCCTTCA
TGTATGGTTACCCGATGCTATGGAAGGGCCTACCCCTATTTCAGCTCTTA
TACACGCTGCTACTATGGTAGCAGCGGGAATTTTTCTTGTAGCTCGGCTT
CTTCCGCTTTTCATAGTTATACCTTTCATAATGAATCTAATAGCTTTGAT
TGGTATAATAACATTACTTTTAGGAGCCACTTTTGCTCTTGTTCAAAAAG
ATATTAAGAGAGGTCTAGCTTATTCTACAATGTCTCAATTGGGTTATATG
ATGTTGGCTCTAGGTATGGGGTCTTATCGAGCTGCTTTATTTCATTTGAT
TACTCATGCTTATTCGAAAGCATTGTTATTTTTAGGGTCCGGATCAATTA
TTCATTCAATGGAAACTATTGTTGGCTATTCTCCAGATAAAAGTCAGAAT
ATGGTTCTTATGGGCGGTTTAAGAAAACATGTACCAATTACAAAAACAGC
GTTTTTATTAGGTACACTTTCTCTTTGTGGTATTCCACCCCTTGCCTGTT
TTTGGTCCAAAGATGAAATTCTTAATGATAGTTGGTTATAT---------

-------CCGTCCCAGAGCATACCCAACTCGCTATAATATTCATATATAC
AATGAT---AATATATATCAGAAAAAGATTGCCCTTTTAAAATTAAATTC
AACATT----------------------CTTAAGCTTAAGAGTATAATAT
TGGAAAAATTG------------------GATTATTATTCTTAATACTAT

AATTCGTTTCTGAATCATATCAATTTCACAAATTGAATTGTATTCAAATA
ACACATAGAGGGAATTGAATCTATTTGTCATGCCTAAATGTTAAA-----
-TATTTAACATCGAATATCTAAAATTCATTTCAGTAACAATTGTTTAGTC
TATCTGATAAATAT----GAGG-------GTTCCATATTATTGTTTTTGG
GATTCTGGTTTTATC--------AAACAGTATGAAAAAATAACAACCCTC
CCTTACATCAGTCTTTATCCACTATTTCACTAAAAAAAGAATTTTTTTTT
TTTTTATATTATATTTTTTTTTATTT------------------------
-----------CTATAACATTAAAAAAGAAA-------------------
--------------------AAAAGAAAAATA----TTAACTTAAATAA-
--------------------ATAAGCAATATATAAAAAAAATGGAATTGA
AATATTCC--------ATTTTAAGATTAATA-------------------

------------------------------A---------------ATAT
ATGTATATGGTATTAAATGGAATTCTTTCT------GCGTCAGAAACCAG
CCATTCATATTTCANATAGAAATAAAAG-------------------GTA
TAGATTATTATGTACCAGCCGAATCTA-TACTATTCGTGATTCCATAATT
GAATCAATTACATACTGGTTCCAATCTAAA--------------------

----------------------TTATTACACTCCTGAATATGAAACCAA
AGATACCGATATCTTGGCAGCATTTCGAGTAACTCCTCAACCCGGAGTTC
CACCTGAGGAAGCGGGGGCCGCGGTAGCTGCTGAATCTTCTACTGGTACA
TGGACAACTGTATGGACTGACGGGCTTACCAATCTTGATCGTTACAAAGG
TCGATGCTACCACATCGAGCCCGTTGCTGGAGAAGAAAGTCAATTTATTG
CTTATGTAGCTTACCCCTTAGACCTTTTTGAAGAAGGTTCTGTTACTAAC
ATGTTTACTTCCATTGTGGGTAATGTATTTGGGTTCAAGGCCCTGCGCGC
TCTACGTCTGGAGGATTTGCGAATCCCTCCTGCTTATTCTAAAACTTTCC
AAGGCCCACCTCATGGCATCCAAGTTGAGAGAGATAAGTTGAACAAGTAT
GGCCGCCCCCTATTGGGATGTACTATTAAACCGAAGTTGGGGTTATCCGC
TAAGAATTACGGTAGAGCCGTTTATGAATGTCTTCGCGGTGGACTTGATT
TTACCAAAGATGATGAGAACGTGAATTCCCAACCGTTTATGCGTTGGAGA
GACCGTTTCTTATTTTGTGCCGAAGCGCTTTATAAAGCACAGGCCGAAAC
TGGTGAAATCAAAGGGCATTACTTGAATGCTACGGCAGGTACATGTGAAG
AAATGATCAAAAGGGCTGTATTTGCCAGAGAATTGGGGGTTCCTATCGTA
ATGCACGATTACTTAACGGGGGGATTCACTGCAAATACTAGCTTGGCTCA
TTATTGCCGAGATAATGGTCTGCTTCTTCACATCCACCGTGCAATGCATG
CAGTTATTGATAGACAGAAAAATCATGGTATACACTTTCGTGTACTAGCT
AAAGCGTTACGTATGTCTGGTGGAGATCATATTCACTCTGGTACTGTAGT
GGGTAAACTTGAAGGGGAAAGAGACATCACTTTAGGCTTTGTTGATTTAC
TACGTGATGATTTTATTGATAAAGATCGAAGCCGTGGTATTTATTTCACT
CAAGATTGGGTCTCTCTACCAGGTGTTCTGCCCGTGGCTTCAGGGGGTAT
TCATGTTTGGCATATGCCTGCTCTGACCGAGATCTTTGGAGACGATTCCG
TACTACAATTTGGCGGAGGAACTTTAGGGCACCCTTGGGGAAATGCACCC
GGTGCCGTAGCTAATCGAGTAGCTCTAGAAGCATGTGTACAAGCTCGTAA
TGAGGGACGTGATCTTGCTCGTGAAGGTAATGAAATTATCCGTGAGGCTA
GTAAA---

--GGTTCAAG

TCCCTCTATCCCCAAAAAGTC-CCATTGGATTCCCTAAATTTTTATC---
-------CTATGCTCTCATTTTGTTAACTGTTCAAAATTCGTTATGTTTC
TCATTCATTCTACTCTTTTACTTTACAAATGGTC------TGAGCGGAAA
TTT--------TTTTCTTTTCACAA-----GCC--------TTGTGATAT
ATAGGATACACGTACAAATGAACATCGTTGGGTACGT----AACCCCGAT
TGTAAATTGT-------AATGATT-AACAATACATATTATTACTTGTACT
GTACTGAAACTTACA----AAGTC------TTCTTTTTG-AAGATCCAAG
AAATT----CCACCAAGACCTGGATAAGGTTTTGTAATC----CCCTTTT
CGTCTTTTTCATTGACATAGAAACAAGTCCTCTATTA------AAATGAG
GATGGTGCGTCGTG-----AATGGTCGGGATAGCTCAGCTGGTAGAGCAG
AGG---
---------------------------------ACTGAACCTTATCATT
TAGAGGAAGGAGAAGTCGTAACAAGGTTTCCGTAGGTGAACCTGCGGAAG
GATCATTGTTGAAACCCT-GCACAGCAGAACGACCCGCGAACTAGTAAAA
TTACAACCGGGGGGCGAGGGG---CAAACCCAGA--CCC-C----CCCTT
GGCTGGG--GCGGTGC---CGGCTCGGGCTTCGCTTGCCGATGCCCGCCG
CCGTCGCCTCCCCC-GCCG-CACAAACGAACCCCGGCGCAAACCGCGCCA
--AGGAACTCGAACGAATCAAG-CATTGCCCCGGCGCCCCAGAGATGGTG
CGCGTTCGGGGTGCTGCG--TCGTATTCTG--TATGTCATAACGACTCTC
GGCAACGGATATCTCGGCTCTCGCATCGATGAAGAACGTAGCGAAATGCG
ATACTTGGTGTGAATTGCAGAATCCCGTGAACCATCGAGTCTTTGAACGC
AAGTTGCGCCCGAAGCCA--CTAGGCCGAGGGCACGTCTGCCTGGGCGTC
ACACAACGTTGCCCCCCCAACCCCAAACCTCGTCTTCGAG--G-------
-----------AGGAAGGGGGGGCGGATGCTGGCCTCCCGTGCGCCCCGG
CTCG--CGGTTGGCCCAAAT--GCGAGCACTCGGCGACGAGCGCCGCGGC
AATCGGTGGTTGTCCAACCCTCG-GTGCCATGCTGCGCGCGCGAGCCGCT
CATCCGTGCTCGTCGACC---CCCA-ACGCGCC---GTACACGCGGCGTT
TACAACGCGACCCCAGGTCAGGCGGGGCTACCCGCTGAGTTTAAGC----

>Frangula_alnus
-------------------------------ATGGAGGAATTTCAAAGAT
ATTTCGAACTCGATAGATCTCAGCAGCATGACCTCCTATACCCACTTATC
TTTCGGGAGTATATTTATGCATTTGCTCATGATCATGGCTTAAATAGATC
AGATTTGTTGGAAAATGTAGGTTATGACAATAAATCTAGTTTACGAATTG
TAAAACGTTTCATTACTCGAATGTATCACCAGAATCATTTGATTATTTCC
GCTAACGATTCTACCCAAAATAAGTGTTTTGGGTACAACAAGAATTTGTA
TTCTCAAATGATATCAGAGGGATTTGCAGTCATTGTGGAAATTCCATTTT
CCTTACGATTACCACCT---------TCGGGGACAGAAATTAGAAAATAT
TATAATTTACAATCAATTCATTCAATATTTCCTTTTTTAGAGGACAAATT
CCCACGTTTAAATTATGTATCAGATGTACTAATACCCTACCCCATTCATC
TGGAAATCTTGGTTCAAATTCTTCGCTATTGGGTGAAAGATGCCTCTTCT
TTGCATTTATTACGGCTTTTTCTTCACGAGTATTCTAATTGGAATAGTTT
TAT---------------------------TACTACAAAAAAATCTATTT
CGATTTTTTCAAAAACAAATCCAAGATTATTCTTGTTCCTATATAATTCT
CATGCTTGTGAATACGAATCCATCTTACT----TTTTCTCCGCAACCAAT
TTTCTCATTTACGATTAACATCCTCTGGGGGCTTTTTTGAGCGAATATAT
TTCTATGGAAAAATAAAACATCCTGTAGAAGAAGTCTTTGCTAATGATTT
TCCGGCTATTCGACGGGTCTTCAAAGATCCTTTCATGCATTATGTTAGAT
ATCGGGGAAAATCCATTTTGGTTTCAAAAGATACTCCTCTTCTAATGAAT
AAATGGAAATATTACCTTGTTCGTTTATGGCAATGGCATTTTTATGTGTG
GGCTCAACCCGGAAGGATCTACATAAACCAATTATTCAAGCATTCCTTCG
GCTTTTTGGGCTATCTTTCAAGTGTGCGACAAAATATTTCATTGGTACGG
AGTCAAACGCTCGAAAATTCATTTATAACGGATAATGCTATTAAGAAGCT
TGATACATTAGCTCCAATCAGTCCTCTGATTGGATCATTGGCTAAAATGA
AATTTTGTAGCGTACTAGGACATCCTGTTAGTAAGTCGACCTGGACCGAT
TCGTCGGATTTTTCTATTATCGACCGCTTTGCACGTATATGCAGAAATCT
TTCTCATTATTACAGCGGGTCCTCAAAAAAAAAGAGTTTGTATCGAATAA
AATATATACTTCGACTTTCTTGTGTTAAAACTTTAGCTCGTAAACACAAA

AGTACTGTACGGGTTTTTTTGAAAAGATTCGGTTCGGAGTTATTGGAAGA
ATTCTTTACGGAGGAAGAAGATGTTCTTTCTTTTATTTTTCCAAGAACTT
ATTCTACTTTTCGGAGTTTATATAGAGGGCGAGTTTGGTATTTGGATATT
GTTTGCATCAACGATCTGGTCAATCAGGAATGA------------------

--TGATAATT
ATATGGCTCATGATCGGGGATATTTGAGGTTTTTTGCTTATATGAGTTTT
TCTAATACTTCAATGTTAGGATTAGTTACTAGTTCAAATTTGATACAAAT
TTATTTTTTTTGGGAATTGGTTGGAATGTGTTCTTATCTATTAATAGGCT
TTTGGTTCACACGACCTATTGCGGCGAATGCTTGTCAAAAAGCGTTTGTA
ACTAACCGCGTAGGGGATTTTGGTTTATTATTAGGAATTTTAGGTTTTTA
TTGGATAACGGGTAGTTTAGAATTTCGGGATTTGTTTGAAATATTCAATA
ATGTGGTTTATAATAATGAAGTTAATTTTTTATTTGTTACTTTGTGTGCC
TTTCTATTATTTGCCGGTGCAATTGCTAAATCTGCCCAATTTCCCCTTCA
TGTATGGTTACCCGATGCTATGGAAGGACCTACCCCTATTTCGGCTCTTA
TACACGCTGCTACTATGGTAGCAGCGGGAATTTTTCTTGTAGCTCGGCTT
CTTCCGCTTTTCATAGTTATACCTTTCATAATGAATATAATAGCTTTGAT
TGGTATAATAACATTATTTTTAGGAGCCACTTTTGCTCTTGGTCAAAAAG
ATATTAAGAGAGGTCTAGCTTATTCTACAATGTCTCAATTGGGTTATATG
ATGTTGGCTCTAGGTATGGGGTCTTATCGAGCTGCTTTATTTCATTTGAT
TACTCATGCTT---

--------------------------------------ACTGCCTTGATCCA
CTTGGCTACATCCGCCCTCAGACTATTTACAAATTCCAAACCAAAAATGT
CAAACCATTCACCTTTTTTTCTTTCTTCTAAGATGAA--ATACAAGATAA
AAGAT--------------------------------AATGGTTTTCTTTCT
TTTATTTTTTATTTAAAAAAATGTAAA------------------AAAAA
AAATTCAAAAAAGATTGAGAAGAACATATATAA-----------ATATAA
AAGTAAAAGAAAGGTTTCTACATAACGAAG-AATTAGTTGAAGT-AAAGA
AAAAAACTT--ACGTAAG-----AAAAAAGAC-----------TCCTAAA
TAATAAAGGAGCAATAACAAACTTCTTGATAGAACAAGAAATTAATTATT
GCTCCTTTATTTTTATTTTCAAGAACTCGT---ATACACTAATACCAAA-
GCGAAGTCTTATCCGTTTGT-----AGATGGAACGTCGACAGCCGCTAGG
TCTAGAGGGAAGTTAT----------------------------------
-------------AGAGCATACCCGACCCGCTATAATATTCATATATAC
AATGAT---AATATATATCAGAAAAAGATTGCCCCTTTTAAATTAAAAAG

AATGAA----------------ACAT-------TCTTAAGAGTATAATAT
TGGAAAAATTGGATTAGTATTCTT------------------AATACTAT
AATTTTTTTCTGAATCATATCAATTTCACAATTTGAATCGTATTCAAATA
ACACACAGAGGGAATTGAATCTATTTGTCATCCCTAAATGGTAAA-----
-TATTTACCATAGAATATCTAGAATTTATTTCAGTAACAATTGTTTAGTC
TATCTGATAGATAT----GAGG-------GTCCCATATTATTGTTTTTGG
GATTCTGGTTTTATC--------AAACAGTATGAAAAAATAACAACCCTC
CCTTACATCAGTCTTTATCCACTATTTCACTAAAAAAAGAAATTCTTTAT
TTTCTATTTTATTCGTGTTATTTATATT--------------------C-
-TATTTTTTTTATATTATAGAAATAAAAAAAATAGAATATAAAATTAAAT
TAAAATAAATATCTATATAAAATAAAAATATA----TAAATATAAAAAA-
--------------------AATAGAAATCTATAAATAAAATGGAATATT
TCAATTCC--------ATTTTAAGATTAATA-------------------

------------------------------A---------------ATAT
ATGTATATGGTATTAAATTGAATTCTTTCTGAGACTTCGTCAGAAACTAG
CCATTCATATTTCATATAGAAATAAAAG-------------------GTA
TAGATTATTATGTACCGGCCGAACCAATGACTATTCGTGATTCCATAATT
GAATCAATTACATACTGATTCCAATCTAAAGGAATGTTATGGTAAAACTT
CGTTT---

-----------CATGGCATCCAAGTTGAGAGAGATAAGTTGAATAAGTAT
GGCCGCCCCCTATTGGGATGTACTATTAAACCTAAATTGGGGTTATCCGC
TAAGAATTACGGTAGAGCCGTTTATGAATGTCTTCGCGGTGGACTTGATT
TTACCAAAGATGATGAGAACGTGAATTCCCAACCGTTTATGCGTTGGAGA
GACCGTTTCTTATTTTGTGCCGAAGCTATTTATAAAGCACAGGCCGAAAC
TGGTGAAATCAAAGGACATTATTTGAATGCTACGGCAGGTACATGCGAAG
ATATGATGAAAAGGGCTGTATTTGCCAGAGAATTGGGAGTTCCTATCGTA
ATGCACGATTACTTAACGGGGGGATTCACTGCAAATACTACCTTGGCTCA
TTATTGCCGAGATAATGGTCTGCTTCTTCACATCCACCGTGCAATGCATG
CAGTTATTGATAGACAGAAAAATCATGGTATGCACTTTCGTGTACTAGCT
AAAGCGTTACGTCTGTCTGGTGGAGATCATATTCACGCTGGTACTGTAGT
GGGTAAACTTGAAGGGGAAAGAGAGATCACTTTAGGCTTTGTTGATTTAC
TACGTGATGATTTTGTTGATAAAGATCGAAGCCGTGGTATTTATTTCACT
CAAGATTGGGTCTCTCTACCAGGTGTTCTGCCCGTGGCTTCAGGGGGTAT
TCACGTTTGGCATATGCCTGCTCTGACCGAGATCTTTGGAGACGATTCCG
TACTACAATTCGGCGGAGGAACTTTAGGGCACCCTTGGGGAAATGCACCC
GGTGCCGTAGCTAATCGAGTAGCTCTAGAAGCATGTGTACAAGCTCGTAA
TGAGGGACGTGATCTTGCTCGTGAAGGTAATGAAATTATTCGTGAGGCTA
GTAAATGGAGTCCTGAACTAGCTGCTGCTTGTGAAGTATGGAAGGAGATT
AAATTTGAATTCCCAGCAATGGATACTTTGTAA-----------------

---CAAAAAGTCCCAAAAGTA-CCATTGGATTCCCTAATTATTTATC---
-------CTATGCTCTCATTTCGTTAACGGTTCAAAATTCCTTATGTTTC
TCATTCAGTCTACTCTTTTCCTTTACAAATGGTAATGGTCTGAGCGGAAA
TTC--------TTTTCTTTTC-------------ACAAGCCTTGTGATAT
ATAGGATACACGTACAAATGAACATCGTTGGGCACGT----AACCCCGAT
CGTAAATTGT-------AATAATT-AACAATACATATTATTACTTGTACT
GTACTGAAACGTACA----AAGTC------TTCTTTTTG-AAGATCCAAG
AAATT----CCACCAAGACCTAGATAAGGCTTTGTAATC----TCCTTTT
CGTCTTTTTCATTGACATAGAACCAAGTCCTCTATTA------AAATGAG
GATGGTGCGTCGTG-----AATGGTCGGGATAGCTCAGCTGGTAGAGCAG
AGGACTGAAAATC---

--AAG
GATCATTGTCGAAACC-CTGCACAGCAGAACGACCAGCGAACTTGTAAAA
CCAACTCGG-GGGCGAGGGG----CCCTAGGGCC--CCG-GACCTCCTTG
GCTTGGG--AGGCACC---ATCACCGGGCGTCGCTGGTCGACGCGCGTTG
GTGTTGCCTTCCC-AGCCG-CACAAACGAACCCCGGCGCAAAACGCGCCA
--AGGAACTTGAACGAATCAGCAATATGCCCCGGCGCCCCAGAGATGGTG
TGTGTTCGGGGTGTTGCG--TCGTAT--TCTTTATGTCAAAACGACTCTC
GGCAACGGATATCTCGGCTCTCGCATCGATGAAGAACGTAGCGAAATGCG
ATACTTGGTGTGAATTGCAGAATCCCGTGAACCATCGAGTCTTTGAACGC
AAGTTGCGCCCGAAGCCA--TTAGGCCGAGGGCACGTCTGCCTGGGCGTC
ACACAACGTTGCCCCCCCCCAAAC----CTCAAC---CTCGAG-------
-----------GAGGACGGGGGGCGGATGCTGGCCTCCTGTGCGCCACGG
CTCG--CGGTTGGCCCAAAT--GCGAGTGCTCGGCGACGAGCGCCGCGGC
AATCGGTGGTTGTCCAACCCTCG-GTGCCATGCTGCGCGCGCGAGTCGCT
CGCTGTGTTTACTCCACG---ACCC-CCAATGCGTCGCATCCGCGACGCT
CACAACGCGACCCC--

>Rhamnus_davurica
-------------------------------------ATGGAGGAATTTCAAAGAT
ATTTCGAACTAGATAGATCTCAGCAGCATGACCTCCTATACCCACTTATC
TTTCGGGAGTATATTTATGCATTTGCTCATGATCATGGCTTAAATAGATC
AGATTTGTTGGAAAATGTAGGTTATGACAATAAATCTAGTTTACGAATTG
TAAAACGTTTCATTACTCGAATGTATCGCCAGAATCATTTGATTATTTCC
GCTAACGATTCTACCCAAAATAAGTGTTTTGGGTACAACAAGAATTTGTA
TTCTCAAATGATATCAGAGGGATTTGCAGTCATTGTGGAAATTCCATTTT
CCTTACGATTACCACCT---------TCGGGGACAGAAATTGTAAAATAT
TATAATTTACAATCAATTCATTCAATATTTCCTTTTTTAGAGGACAAATT
CCCACGTTTAAATTATGTATTAGATGTACTAATACCCTACCCCATTCATC
TGGAAATATTGGTTCAAATCCTTCGCTATTGGGTGAAAGATGCCTCTTCT
TTGCATTTATTACGGCTTTTTCTTCACGAGTATTCTAATTGGAATAGTTT
TAT---------------------------------TACTACAAAAAAATCTATTT
CGATTTTTTCAAAAACAAATCCAAGATTATTCTTGTTCCTATATAATTCT
TATGCTTGTGAATACGAATCCATCTTACT----TTTTCTCCGCAACCAAT
TTTCTCATTTACGATTAACATCTTCTGGGGTCTTTTTTGAGCGAATATAT
TTCTATGGAAAAATAAAACATCCTGTAGAAGAAGTCTTTGCTAATGATTT
TCCGGCTATTCGACGGGTCTTCAAAGATCCTTTCATGCATTATGTTAGAT
ATCAGGGAAAATCCTTTTTGGTTTCAAAAGATACGCCTCTTCTAATGAAT
AAATGGAAATATTACCTTGTCCATTTATGGCAATGGCATTTTTATGTGTG
GGCTCAACCCGGAAGGATCTACATAAACCTATTATTCAAGCATTCCTTCG
GCTTTTTGGGCTATCTTTCAAGTGTGCGACAAAATCTTTCATTGGTACGG
AGTCAAACGCTCGAAAATTCATTTATAATGGATAATGCTATTAAGAAGCT
TGATACATTAGCTCCAATCAGTCCTCTGATTGGATCGTTGGCTAAAATGC
AATTTTGTAGCGCACTAGGACATCCTGTTAGTAAGTCGACCTGGACCGAT
TCGTCGGATTTTTCTATTATCAACCGCTTTGCGCGTATATGCAGAAATCT

TTCTCATTATTACAGCGGGTCCTCAAAAAAAAAAGAATTTGTATCGAATAA
AATATATACTTCGACTTTCTTGTGTTAAAACTTTAGCTTGTAAACACAAA
AGTACTGTACGGGTTTTTTTGAAAAGATTCGGTTCGGAGTTATTGGAAGA
ATTCTTTACGGAGGAAGAAGATGTTCTTTCTTTTATTTTTCCAAGAACTT
ATTCTACTTTTCGGAGTTTATATAGAGGGCGAGTTTGGTATTTGGATATT
GTTTGCATCAATGATCTGGTCAATCAGGAATGA---GATCATACCTTTCA
TTCCCCTTCCAACCCCTTTGTTAGTAGGAGTAGGACTTCTACTTTTTCCC
ACGGCAATAAAAAACCTTCGTCGTATGTGGGTTTTTCCTAGTGTTTTACT
GTTAAGTATAGTTATGCTTTTTTCAGCTCATATATTTATTCAACAAATAA
ATAATAGTTCTATCTATCTATATGTATGGTCTTGGATTATCAATAATGAT
TTTTCTTTAGAATTTGGCTATTTGATCGATCCACTTACTTCTATTATGGT
ACTATTAATCACTACTGTTGGAATTATGGTTCTTATTTATAGTGATAATT
ATATGGCTCATGATCAAGGATATTTGAGATTTTTTGCTTATATGAGTTTT
TTCAGTACTTCCATGTTGGGATTAGTTACTAGTTCAAATTTGATACAAAT
TTATATCTTTTGGGAATTGGTTGGAATGTGTTCTTATCTATTAATAGGCT
TTTGGTTCACACGACCTATTGCGGCGAATGCTTGTCAAAAAGCGTTTGTA
ACTAACCGCGTAGGGGATTTTGGTTTATTATTAGGAATTTTAGGTTTTTA
TTGGATAACAGGGAGTTTAGAATTTAGGGATTTGTTCGAAATATTCAATA
ACGTGGTTTATAATAATGAAGTTAATTTTTTATTTGTTACTTTGTGTGCC
TTTCTATTATTTGCCGGTGCAATTGCTAAATCTGCACAATTTCCTCTTCA
TGTATGGTTACCCGATGCTATGGAAGGACCTACCCCTATTTCGGCTCTTA
TACACGCTGCTACTATGGTAGCGGCGGGAATTTTTCTTGTAGCTCGGCTT
CTTCCGCTTTTCATAGTTATACCTTACATAATGAATATAATAGCTTTGAT
TGGTATAATAACATTATTTTTAGGAGCCACTTTTGCTCTTGTTCAAAAAG
ATATTAAGAGAGGTCTAGCTTATTCTACAATGTCTCAATTGGGTTATATG
ATGTTGGCTCTAGGTATGGGGTCTTATCGAGCTGCTTTATTTCATTTGAT
TACTCATGCTTATTCGAAAGCATTGTTATTTTTAGGGTCCGGATCAATTA
TTCATTCAATGGAAACTATTGTTGGATATTCTCCAAATAAAAGTCAGAAT
ATGGTTCTTATGGGCGGTTTAAGAAAACATGTACCAATTACAAAAACTGC
GTTTTTATTAGGTACACTTTCTCTTTGTGGTATTCCACCTCTTGCCTGTT
TTTGGTCAAAAGATGAAATTCTTAATGATAGTTGGTTATATTCACCTATT
TTTGCAATAATCGCTTGTTACACAGCAGGATTAACTGCATTTTATATGTT
TCGGATCTATTTACTTACTTTTGAAGGACACTTAAATGTTCATTTTCAAA
CTTACAGCGGCAAAAAAAAAAGCCCGTTCTATTCAATATCTCTATGGGGT
AAAGAAGGACCAAAAGTTATTAAAAAAAAAAATAGCTTATTGGCTTTATT
AACAATCAATAATAATCAAAGGACTTCTTTTTTTTGTAAAAAGACATATC
GAATTGATAGTAATGATAGTAATGTAAGAAGCATGGCGCAGCCTTTTAGT
ACTATTATTCGTTTTGGTACTAAGAACACTTATTTCTATCCTCACGAGTC
GGACAATACTATGCTATTTCCTATGCTTGTATTAGTTCTATTTACTTTGT
TAATTGGAGTAATAGGAATTCCGCTCAA---T---------CAAGAAGGG
GGGGATTCGGATATATTATCAAAATGGTTAACCCCGGCTATAAACCTTTT
ACATCAAAATGAAAAAAATTCTGTGGATTGGTATGAATTTATGACAAACG
CAACCTTTTCAGTCAGTATCGCTTTTTTCGGAATCCTTATAGCGTCTTTT
TTATATAAGCCGGCTTATTCATCTTTACAAAATTTGAATTTCTTTAATTC
AGTTGTTA---------AAAGGATTCCGAAAAAAACAAAGATTTTGGGGG
ATAAACTACTAAATTTTATATATGATTGGTCATATAATCGCGGTTACATA
GATTCTTTTTATTCAATATCCTTAACTCGGGGTATAAGAGGTTTGGCTCA
ACTAACTCATTTTTTTGATAAACGAATAATTGATGGAATTACGAATGGGG
TCGGTATTGTGAGTTTTTTTCTAGG------------------------
-----CTTCCCTCTAGACCTAGCTGCTGTCGACGTTCCATCTACAAACGG
ATAAGACTCCGCTTT---GGTATTAGTGTATACGAGTTCTT-GAAAATAA
AGGAGCAATAATGAATTTCTTGTTCTATCAAGAAGTTTGTTATTGCTCCT
TTA-TTATTTAGGATTCTTTTTTCTTACGTAAGTTTTTTTC-------TT
TACTTCAACAAATTCTTCGTT----ATGTAGAAACTTTTATTTTATATAT
GTTCCTCCCAATCTTTTTTGA-----ATTT---TTATTTT---------T
TTAAATGTAAAAAATAAAAGAAAGGAAACCATTATCTTTTATCTTGTATT
TCAT-----CTTAGAAGAAAGAAAAAAGGTGA---------ATGGTTTGA
CATTTTTGG-----------------TTTGGAATTTGTAAAGAGTCTGG
GGG---

------------------------AAGATTGCCCCTTTAAAATTAAATAA
AATGAA----------------ACAT-----TCTTTATAATATATAATAT
TGG------------------AAAAATTGGATTATTATTCTTAATACTAT
AATGATTTTCTGAATCATATCAATTTCACAAATTGAATCGTATTCAAATA
ACACACAGAGGGAATTGAATCTATTTGTCATCCCTAAATGTTAT------
------ACCATAGAATATCTATAATTTAGTTCAGTAACAATTGTTTAGTC
TATGTGATAGATAT----GAGG-------GTCCCATATTATTGTTTTTGG
GATTCTGGTTTTAGC--------AAACAGTATGAAAAAATAAC-AACCCC
CCTTACATCAGTCTTTATCCACTATTTCACTAAAAAAAGAAATTCTTTTT
TTTTTATTCGTGTTATTTATAATTTTTTTC----------TA--TTCTAT
GTTTTTTATTTCTATAATATAAATAAAAA----------------AAAAA
TTCAATTGAAAT-------------------------------ATTAAC-
--------------------TTAAAAAATCTATAAATAAAATGGAATTGA
AAT---

----------------------------------ATTCATTAATAAATCT
ATGTATATGATATTAAATTGAATTCTTTCTGAGACTTCGTCAGAAACTAG
CCATTCATATTTCATATAGAAATAAAAG-------------------GTA
TAGATTATTATGTACCGGCTGAACCAATGACTATTCGTGATTCCATAATT
GAATCAATTACATACTGGTCC-----------------------------

----------------------ATGTATTTGGGTTCAAGGCCCTGCGCGC
TCTACGTCTGGAGGATTTGCGAATCCCCCCTGCTTATACTAAAACTTTCC
AAGGCCCGCCTCATGGCATCCAAGTTGAGAGAGATAAGTTGAACAAGTAT
GGCCGCCCCCTATTGGGATGTACTATTAAACCTAAATTGGGGTTATCCGC
TAAGAATTACGGTAGAGCCGTTTATGAATGTCTTCGCGGTGGACTTGATT
TTACCAAAGATGATGAGAACGTGAATTCCCAACCGTTTATGCGTTGGAGA
GACCGTTTCTTATTTTGTGCCGAAGCAATTTATAAAGCACAGGCCGAAAC
TGGTGAAATCAAAGGGCATTACTTGAATGCTACGGCAGGTACATGCGAAG
AAATGATGAAAAGGGCTGCATGTGCCAGAGAATTGGGAGTTCCTATCGTA
ATGCACGATTACTTAACGGGGGGATTCACTGCAAATACTACCTTGGCTCA
TTATTGCCGAGATAATGGTCTGCTTCTTCACATCCACCGTGCAATGCATG
CGGTTATTGATAGACAGAAAAATCATGGTATGCACTTTCGTGTACTAGCT
AAAGCGTTACGTATGTCTGGTGGAGATCATATTCACGCTGGTACTGTAGT
GGGTAAACTTGAAGGGGAAAGAGAGATCACTTTAGGCTTTGTTGATTTAC
TACGTGATGATTTTGTTGATAAAGATCGAAGCCGTGGTATTTATTTCACT
CAAGATTGGGTCTCTTTACCGGGTGTTCTGCCCGTGGCTTCAGGGGGTAT
TCACGTT---

--------------------------TGGAAACCTACCAAGTGATAACTT
TCAAATTCAGAGAAACCCTGGAATTAAAAACGGGCAATCCTGAGCCAAAT
CCCGTTTTCTGAAAACAAACAAAGGTTCAGAAAGCGATAATAAA------
-------AAAGGATAGGTGCAGAGACTCAATGGAAGTTGTTCTAACAAAT
GG----AGTTGGCCACGATGCGTT------AGTAAAGGACT-CCTTCCAT
CGAAACTCCAGAAAGTATGAAGAATAAACGTATATATACGTACT------
GAAATACTATCTCCAAACCAAATGATTAATGACGACCCGAATCTTTTTTT
TTTA-------TATAAAA-------------------------------A
ATGAAAGAATTGGTGT----------GAATCGATTCCAAGTTG-AAAACA

GAATGGAATATTCATTGATCAAATCATTTACTCCATCGTAATTTGATAGA
TAGATCTTTTGAAGAATTGAT-----T-AATCGGACGAGAATAAAGATAG
AGTCCCGTTCTACATGTCAA------------TATCGACAACAATGCAAT
TTATAGTAAAAGGAAAATCCGTCGACTTTA-GAAATCGTGAGGGTTCAAG
TCCCTCTATCCCCAAAAAGTC-CCATTGGATTCCCTAATTATTTATC---
-------CTATGCTCTCATTTCGTTAACGGTTCAAAATTCGTTATGTTTC
TCATTCATTCTACTCTTTTAGTTTACAAATGGTAATGGTCTGAGCGGAAA
TAT--------TTTTATTTTCACAA-----GCCTTGTGATATTGTGATAT
ATAGGATACACGTACAAATGAACATCGTTGGGCACGT----AACCCTGAT
TGTAAATTGT-------AATGATT-AACAATACATATTCTTACTTGTATT
GTACTGAAACGTACA----AAGTC------TTCTTTTTG-AAGATCCAAG
AAATT----CCACCAAGACCTGGATAAGGCTTTGTAATC----CCCTTTT
CGTCTTTTTCATTGACATAGAACCAAGTCCTCTATTA------AAATGAG
GATGGTGCGTCGTG-----AATGGTCGGGATAGCTCAGCTGGTAGAGC--

---G
GATCATTGTCGATACC-CTGCACAGCAGAACGACCCGCGAACTAGTAAAT
CCAACTCGG-GGGCGAGGGG----CCCTAGAGCC--CCG-RACCTCCTTG
GCCTGGG--GGACGCC---ATCGTCGAGCGGCGCTGGTCGACGCTCGTTG
GTGCCGCCCTCCA-AGCCG-CACAAACGAACCCCGGCGCAAAACGCGCCA
--AGGAACTTCAACGAATCAGC---ATGCCCCGGCGCCCCAGAAATGGTG
CGTGTTCGGGGCGTTGCG--TCGTAT--TCTTTATGTCAAAACGACTCTC
GGCAACGGATATCTCGGCTCTCGCATCGATGAAGAACGTAGCGAAATGCG
ATACTTGGTGTGAATTGCAGAATCCCGTGAACCATCGAGTCTTTGAACGC
AAGTTGCGCCCGAAGCCA--TTAGGCCGAGGGCACGTCTGCCTGGGCGTC
ACACAACGTTGCCCCC--CCAAAC----CTCGAC---TTCCAA-------
-----------GAGGACGGGGGGCGGATGCTGGCCTCCCGTGCGCCACGG
CTCG--CGGTTGGCCTAAAT--GCGAGTACTCGGCGACGAGCGCCGCGGC
AATCGGTGGTTGTCCAACCCTCG-GTGCCATGCTGCGCGCGCGATCCGCT
CACTGTTACTCGACGAC-------C-CCAATGCGTCGTAGCCACGACGCT
CACAACGCGAC---------------------------------------

>Elaeagnus_angustifolia

TTGCATTTACTACGACTCTATCTTCACAAGTATTTTAATTGGAATAGTAT
TAT---------------------------TACCCCAAAGAATTCTGTTT
TTCTTTTTTCAAAAATTAATCCAAGATTTTTATTGTTCCTATATAATTCT
CATGTTTGTGAATACGAATCTATCTTACT----TTTTCTTCGTAACAAAC
CTTCTCATTTACGATTAACGTCTTCTGGATCTTTTTTTGAACGAATTTTT
TTCTATGCAAAAATCAAACATTCTGTAGAAGAAGTATTTGCTAATGATTT
TTCAATTACCCCGTGGTTCTTCAAGGCCCCTTTCATGCATTATGTTCGAT
ATCGAGGAAAATCTATTTTGGCTTCAAAAGATACACCTCTTCTGATGAAT
AAATGGAAATATTACCTTATCCATTTATGGCAATCTTATTTTTATGTGTG
GTCTCAACCAGCAAGGATCTATATAAACCAATTATCCAAGCATTCCCTCA
TTTTTTTGGGTTATTTTTCAAGTATACGACTAAAGCTTTCGGTGGTACGG
AGTCAAATGCTAAAAAATGCATTTCTAATAGATAATGCAATGAAGAGGCT
TGATACATTAGTTCCAATTAGTCCTCTAATTGGATCGTTAGCTAAAATGA

AATTTTGTAATGGATTAGGACATCCGGTTAGTAAGTCAATCTGGGCCGAT
TCGTCGGATTTGGATATTATCGATCGATTTGCACATATATGTAGAAATCT
TTCTCATTATTACAGCGGATCCTCAAAAAAAAAAAAGTTTGTATCGAATAA
AATATATACTTCGACTTTCTTGTGTTAAAACTTTGTCTCGTAAACACAAA
AGTACTGTACGCGCTTTTTTGAAAAGACTAGGTTTGGGATTATTAGAAGA
ATTCTTTACAGAA---GAAGAGATTCTCTCTTTGATTTTTCCAAAAACTT
ATTCTACTTTTCGTAAGTTATATAGAGGACGAATTTGGTATTTGGATATT
TTTTGTATCAATGATCTGGTCAATCATGAATGA-----------------
-----------------------ATAGGAGTAGGACTTTTACTTTTTCCG
ACAGCAACAAAAAATTCTCGCCGTATATGGGTTTTTCCTAGTGTTTTATT
GTTAAGTATAGTTATGTTTTTTTCAGCCCATCTATTTATTCAACAACTAA
ATAACCATTCTACCTATCTATATGTATGGTCTTGGACCCTCAATAATAAT
TTCTCTTTAGAATTTGGTTACTTGATTGATCCACTTACTTCTATTATGTT
AATATTAGTCACTACTGTTGGAATTATGGTTCTTATTTATAGTGACAATT
ATATGTCACACGATCAAGGATATTTAAGATTTTTTGCTTATTTGAGCCTT
TTTAATACTTCAATGTTGGGATTAGTTACTAGTTCGAATTTGATACAAAT
TTATTTTTTTTGGGAATTGGTTGGAATGTGTTCTTATCTATTAATAGGTT
TTTGGTTCACACGACCAGTTGCGGCGAATGCTTGTCAAGAGGCGTTTGTA
ACTAATCGCGTAGGGGATTGGGGTTTATTATTAGGAATATTAGGTTTTTA
TTGGATAACGGGTAGTTTAGAATTTCGAGAGTTGTTTGAAATATTGAATA
ACCTGGTTTCTAATAATCAAGTTAATTTTTTATTTGCTACTTTGTGTTCC
TTTTTATTATTTGCCGGTGCTGTTGCTAAATCTGCCCAATTCCCCCTTCA
TGTATGGTTACCTGATGCTATGGAAGGACCGACTCCTATTTCAGCTCTTA
TACATGCGGCTACTATGGTAGCAGCAGGAATTTTTCTCGTAGCCCGGCTT
CTTCCTCTTTTCATAATTATACCTTACATAATGAATCTAATAGCTTTGAT
AGGTATAATAACATTACTTTTGGGAGCCACCTTAGCTCTTGCTCNNNNNN
NNNNNNNNNNNNNNNNNNNNNNNNNNNNNNNNNNNNNNNNNNNNNNNNNN
NNNNNNNNNNNNNNNNNNNNGGTCTTATCGAGCTGCCTTATTTCATTTGAT
TACTCATGCTTATTCAAAAGCATTGTTATTTTTAGGATCCGGATCCATTA
TTCACTCAATGGAAACTATTGTTGGATATTCTCCGGATAAAAGTCAGAAT
ATTATTCTTATGGGCGGGTTAAGAAAACATATACCAATTACAAAAACCGC
TTTTTTGTTAGGGACTCTTTCTCTTTCTGGTATTCCCCCCCTTGCCTGTT
TTTGGTCCAAAGATGAAATTCTTAACGATAGTTGGTTGTATTCACCGATT
TTTGCAATAATAGCTTGTTCCACAGCAGGATTAACTGCATTTTATATGTT
TCGAATCTATTTACTTACTTTTGAGGGTCATTTAAGTGTTCATTTTCAAA
CTTACAGCGGCAAAAAAAATAACTCATTCTATTCAATATCTTTATGGGGA
AAGGGGGGAAGAATTAAA------AACAATTTTCATTTATTACCTTTAGT
AAATCGGAAAAAAGCATATCGAG---------TT---------------G
ATAAGACTGTAAAAACAAAAACTATG---------ACACAGTCCTTTATT
ACTACTTATCATTTTGGTACTACAAACACATTTTCGTATCCGCATGAGTC
GGACAATACCATGCAATTTCCTATACTTGTATTACTTCTATTTACTTTGT
TGATTGGAGTTATAGGAATGACTTTTTGGAATCAAGAAGGTG--------
-------TGGATATATTATCCAAATTATTAACTCCTTCTATAAATCTTTT
ACA------CCCAAACAATTCTGTGGATTCTTTTGAATTTTTTTCAAATA
CTATTTTTTCAGTCAGTATAGCTTATTTTGGAATCCTTATAGCGTCTTTT
TTATATAAACCTGTTTATTCATCTTTACAAAATTTGAATTTATTTAATTC
ATTTGTTA---------AAAGTATTTCTAAAAAAATGAAAATTGCGAGAG
ATAAAATAATAAATGTGATATATAATTGGTCATATCATCGTGGTTATATA
GACTCTTTTTATGAAATATTCCTAATTAAAGGTATAAGAGTATGGCTGAA
CTA---

-----------------------------------GTTGGATTCAAAGCG
GGTGTTAAAGATTATAAATTGACTTATTACACTCCTGACTATCAAACCAA
AGATACTGATATCTTAGCAGCATTTCGAGTAACTCCTCAACCAGGAGTTC
CACCCGAAGAAGCAGGGGCAGCGGTAGCTGCTGAATCTTCTACTGGTACA
TGGACAACTGTATGGACTGACGGGCTTACCAGTCTTGATCGTTACAAGGG
TCGATGCTACGGCCTTGAGCCCGTTGCTGGAGAAGAAAATCAATATATTG
CTTATGTAGCTTACCCCTTAGACCTTTTTGAAGAAGGCTCTGTTACTAAC
ATGTTTACTTCCATTGTGGGTAATGTGTTTGGATTCAAGGCCCTGCGCGC
TCTACGTCTGGAAGATTTGCGAATCCCTACTGCTTATACTAAAACTTTCC
AAGGCCCGCCTCATGGTATCCAAGTTGAGAGAGATAAATTGAACAAGTAT
GGCCGTCCCCTATTAGGATGTACTATTAAACCTAAATTGGGGTTATCCGC
TAAGAATTATGGTAGAGCTGTTTATGAATGTCTTCGCGGTGGGCTTGATT
TTACCAAAGATGATGAGAACGTGAATTCCCAACCATTTATGCGTTGGAGA
GACCGTTTCTTATTTTGTGCCGAAGCCCTTTTTAAAGCACAGGCTGAAAC
AGGTGAAATCAAAGGGCATTATTTGAATGCTACGGCAGGTACATGCGAAG
AAATGATGAAAAGGGCTATATTTGCCAGAGAATTGGGAGCTCCTATCGTA
ATGCACGATTACTTAACTGGGGGATTCACTGCAAACACTAGCTTGGCTCA
TTATTGCCGAGATAATGGTTTACTTCTTCACATCCACCGTGCAATGCATG
CAGTTATTGATAGACAGAAAAATCATGGTATGCACTTCCGTGTACTGGCT
AAAGGGTTACGTATGTCTGGTGGAGATCATATTCACGCTCGTACTGTAGT
AGGTAAGCTTGAAGGGGAAAGAGAAATTACTTTAGGCTTTGTTGATTTAC
TACGTGATGATTATATTGAAAAAGATCGAAGCCGTGGTATTTATTTCACC
CAAGATTGGGTCTCTCTACCGGGTGTTCTTCCTGTGGCTTCAGGGGGTAT
TCACGTTTGGCATATGCCTGCTCTGACCGAGATCTTTGGAGATGATGCCG
TACTACAATTCGGCGGAGGAACTTTAGGGCACCCTTGGGGAAATGCACCT
GGTGCTGTAGCTAACCGAGTAGCTCTAGAAGCATGTGTACAAGCTCGTAA
TGAGGGGCGTGATCTTGCTCGTGAGGGTAATGRAATTATTCGTGAGGCTT
GTAAATGGAGTCCTGAACTAGCTGCTGCTTGTGAAGTATGGAAGGAGATC
AAATTTGAATTTGAAGCTATGGATACAATTTGA-----------------

-------------------GGAATTAATAATGGGCGATCCTGAGCCAAAT
CCAGTTTTCTGACTCTGTGAAAACAAACAAGG----GTT---C---AGAA
AGCAATAAAAGGATAGGTGCAGAGACTCAATGGAAGCTGTTCTAACAAAT
GG----AGTTGGCTACGTTGTGTT------AGTAAAGGAATCCTTCCTTT
CGAAACTTCCGAAAGGATGAAGAAGACCCTATATATACGATATACGTACC
GGAATACTATCTGAAAAT-----GATTAATGATGGCCTGAATCTTTTTTT

TTTATTGATAT------GAAAAA------TG-------------------
-------AAAAAATTGTTG-----TGAATCGATTCACATCTGAAAAAAAA
AAATCGAATATTGATGGATCAAATCATTTACTCCATCATAATCTGAT---
-AGATCTTTTGAAGAATGGAT------TAATCGGACGAGAATAAAGATAG
AGTCCCATTCTACATGTCAA------------TATCGACAACAATGAAAT
TTATAGTAAGAGGAAAATCCGTCGACTTTA-GAAATCGTGAGGGTTCAAG
TCCCTCTATCCCCAAAAGGGC-CTATTTGATTTCCCTCAT----------
---------------TTCTTTCGTTAGTGGTTCAAAATTCGTTATGTTTT
TCATTCATTCTAACTATAATCTTTCGCATTCACCAACGGATTTGGGGAGA
ATTTTTTCT--TATCACAAGCCTTGC----GATACATATGATATTAT---
----GATATGTGGACAAATGAATATCT--TTGAGCAAGT--AATTCCAAT
TTCAAATTGG-------AATTATT-TACAATCCATACCATTACTGTTAAT
GAAGTTAATGAAACTTACAAAGTTTC-------ATTTTGGAAGATCCAAG
AAATT----GC--------------GTAACTTTGTAA-----TCCCTTTT
TATCTTTTTAATTGACATAGACCC--------------------------

---------------TGGAGAATCCTGGG-CCCCAGAGTGTCTTTGACAC
AAGTTGCGCCCGAAGCCA--TCCGGTTGAGGGCACGTCTGCCTGGGCGTC
ACACACCGTTGCCCTCCCGACACCACGTGCCTTCAAGGCTGCG-------
----------TCGGTCGTGGAGGCGTACATTGGCTTCCCGCGAGCCTCGT
CTCG--TGGCTGGCCCAAA-T-TCTAGTCATTGGCGACCGGTGCCACGAC
AATG-GTGGTCGTCGAACCTTCG-GTCCCCCGTCGTGGGTTCCGGTCGTG
CAT-------GATGCAGGGACCCCG----AAGCATCCGGTGAAGGATGCT
CTCGATGCGACCCCAGGTCAGGCGGGGCTACCCGCTGAGTTTAAGCATAT
CAATAAGCGGAGGAAAAGAAACTTACAAGGATTCCCCTAGTAACGGCGAG
CGAACCGGGAACACCCCACGTGGAAAATCTCATACGCTTGTGGTTCTAAT
TGTGTTCTGGGAAAACGTCACA
>Hippophae_neurocarpa

---GCCTCTTCT
TTGCATTTACTACGACTATTTCTTCACGAGTATTCTAATTGGAATAGTAT
TAT---------------------------TACCCCAAAGAATTCTGTTT
TGATTTTTTCAAAAAGTAATCCAAGATTATTCTTGTTCCTATATAATTCT
TATGTTTGTGAATACGAATCTATCTTACT----TTTTCTTCGTAACCAAT
CTTCTCATTTACGATTAATGTCTTCTGGATCTTTTTTTGAACGAATTTTT
TTCTATACAAAAATAAAACATCCTGTAGAAGAAGTCTTTGTTAATGATTT
TTCAGCTACCCCGTGGTTCTTTAAGGCTCCTTTCATGCATTATGTTCGAT
ATCGAGGAAAGTATATTTTGGCTTCAAAAGATACGCCTCTTCTGATGAAT
AAATGGAAATATTACCTTATCTATTTATGGCAATGTTATTTTTATGTGTG
GTCTCAACCAACAAGGATCTCTATAAATCCATTATCCAAGCATTCCCTCG
CTTTTTTGGGCTATTTTTTAAGTATACGACTAAACCTTTCGGTGGTACGG

AGTCAAATGCTAAAAAATGCATTTATAATGGATAATGCTATGAAGAGACT
TGATACATTAGTTCCAATTAGTTCTTTAATTGGATCGTTAGCTAAAATGA
AATTTTGTAATGGATTAGGACATCCGGTTAGTAAGTCGATCTGGGCCGAT
TGGTCGGATTTGGATATTATCGATCGATTTGCGCATATATGTAGAAATCT
TTCTCATTATTACAGTGGATCCTCAAAAAAAAAAGGTTTGTATCGAATAA
AATATATACTTCGACTT---

---------GATTATAAATTGACTTATTACACTCCTGACTATCAAACCAA
AGATACTGATATCTTAGCAGCATTTCGAGTAACTCCTCAACCTGGAGTTC
CACCTGAAGAAGCAGGGGCAGCGGTAGCTGCTGAATCTTCTACTGGTACA
TGGACAACTGTATGGACTGACGGGCTTACCAGTCTTGATCGTTACAAAGG
TCGATGCTACCATCTTGAGCCCGTTGCTGGAGAAGAAAATCAATATATTG
CTTATGTAGCTTACCCCTTAGACCTTTTTGAAGAAGGCTCTGTTACTAAC
ATGTTTACTTCCATTGTGGGTAATGTGTTTGGATTCAAGGCCCTTCGCGC
TCTACGTCTGGAGGATTTGCGAATCCCTACTGCTTATACTAAAACTTTCC
AAGGCCCGCCTCATGGTATCCAAGTTGAGAGAGATAAATTGAACAAGTAT
GGTCGTCCCCTATTAGGATGTACTATTAAACCTAAATTGGGGTTATCCGC
TAAAAATTACGGTAGAGCTGTTTATGAATGTCTT----------------

-----TTCAGAGAAACCCTGGAATTAAAAATGGGCAATCCTGAGCCAAAT
CCAGTTTTCTGATTCTTAAAACAAACAGGG-----GTTC---A---GAAA
GCAATATAAAGGATAGGTGCAGAGACTCAATGGAAGCTGTTCTAACAAAT
GG----AGTTGGCTGCGTTGCGTT------AGTAAAGGAAT-CCTTCCAT

CGAAACTTACGAAAGGATGAAGAATACCCTATATATACGATATACGTACT
GAAATACTATCTGAAAAT-----GATTAATGACGACCTGAATCTTTTTT-

----------------------------TTTTAGAATTGATATGAAAAAA
GAATCGAATATTCATTGATCAAATCATTTACTCCATCATAATCTGAT---
-AGATCTTTTGAAGAATTGAT------TAATCGGACGAGAATAAAGATAG
AGTCCCATTCTACATGTCAA------------TATTGACAACAATGAAAT
TTATAGTAAGAGGAAAATCCGTCGACTTTA-GAAATCGTGAGGGTTCAAG
TCCCTCTATCCCCAAAAAGGC-CTATTTGATTCCCTAATTATTTATC---
--CCCTCAT------TTCTTTCGTTAGCGGTTCAAAATTCGTTATGTTTC
TCATTCATTCTAACTATAATCTTTCGCATTCACAAATTGATTTGATCAGA
AATTTTTTTCTTATCACAAGCCCTGT----GATACATATGATATGCG---
----GCC--------AAATCAATATCT--TTGAGCAAAAGTAATTCCAAT
TTGAAATTGG-------AGTGATT-TCCAATACATACCATTACTCATACG
TTAATGAAA----CTTTGGAAG-------TTTTATTTTGGAAGAGCCAA-
GAAAT----TCCATGAGGCTTGGATAAAACTTTGGAAT-----CCCTTTT
TATCTTTTGAATTGACATAGACCCAAGCTATCTATTAG------AATAAG
GATGGTGCGTTGTG-----AATGGTCG-----------------------

--------TCGAAA-CCT-GCCATGCAGAACGACCTGCGAACTGGTTTAA
AAATAGGGAATGATCTAGCCTCTTGGCCATGATTATTACCCATTGCTAGT
TGGAATGTTTTTTGTGCTTGCACCTTTTCTCTGTTAAAGGGTGTAAGAAT
GATAATGTTCCCC-TGGCAAAAAAAACTAACCCCGGCGCAGATCGCGTCA
--AGGAACTCGAA-CGAATGAGCAT-CCTCTCCTTGGCCCGGACACGGTG
TCCT--TGTGAGGGTTAT--GTTGTCTTCGATATGTAAAAAACGACTCTC
GGCAACGGATATCTCGGCTCTCGCATCGATGAAGAACGTAGCGAAATGCG
ATACTTGGTGTGAATTGCAGAATCCCGTGAACCATCGAGTCTTTGAACGC
AAGTTGCGCCCGAAGCCA--TCAGGCTGAGGGCACGTCTGCCTGGGCGTC
ACACACCGTTGCCCTCCCAACATCACGTTCCTTCAAGGCAACG-------
----------TTGGTTGTGAAGGCGCATATTGGCTTCCCGTGAGCTTTGA
CTTG--TGGTTGGCCCAAAAT-TTTAGTCATTGGCAACCTGTGCTGCGAC
AATG-GTGGTTGTCGAACCTTCG-GTGCCCCGTCGTGTGTGCGGGTTGTT
CGT-------GATGTAAGGAC-CCA----ATGCATTCAATGT-GATGCCT
CTTGATGCGACCCCAGGTCAGGCGGG------------------------

>Shepherdia_canadensis

---------------------CTTCACGAGTATTCTAATTGTAATAGTAT
TAT---------------------------TACCCCAAAGAATTCTGTTT
TGATTTTTTCAAAAAGTAATCCAAGATTATTCTTGTTCCTATATAATTCT
CATGTTTGTGAATACGAATCTATCTTACT----TTTTCTTCGTAACCAAC
CTTCTCATTTACGATTAACGTCTTCTGGATCTTTTTTTGAACGAATTTTT
TTCTATGCAAAAATAAAACATCCTGTAGAAGAAGTCTTTGCTAATGATTT
TTCAGCTACCCCGTGGTTCTTCAAGTCTCCTTTCATGCATTATGTTCGAT
ATCGAGGAAAATCCATTTTGGCTTCAAAAGATACGCCTCTTCTGATGAAT
AAATGGAAATATTACCTTATCTATTTATGGCAATGTTATTTTTATGTGTG

GTCTCAACCAACAAGGATCTATATAAACCCATTATCCAAGCATTCCCTCG
CTTTTTTGGGCTATTTTTCAAGTATACGACTAAACCTTTCGGTGGTACGG
AGTCAAATGCTAAAAAATGCATTTATAATGGATAATGCTATGAAGAGACT
TGATACATTAGTTCCAATTAGTCCTTTAATTGGATCGTTAGCTAAAATGA
AATTTTGTAATGGATTAGGACATCCGGTTAGTAAGTCGATCTGGGCCGAT
TCGTCGGATTTGGATATTATCGATCGATTTGCGCGTATATGTAGAAATCT
TTCTCATTATTACAGCGGATCCTCAAAAAAAAAAAGTTTGTATCGAATAA
AATATATA--

-----------------------------------GTTGGATTCAAAGNG
GGTGTTAAAGATTATAAATTGAATTATTACACTCCTGACTATCAAACCAA
AGATACTGATATCTTAGCAGCATTTCGAGTAACTCCTCAACCTGGAGTTC
CACCTGAAGAAGCAGGGGCAGCGGTAGCTGCTGAATCTTCTACTGGTACA
TGGACAACTGTATGGACTGACGGGCTTACCAGTCTTGATCGTTACAAAGG
TCGATGCTACCACATTGAGCCCGTTGCTGGAGATGAAAATCAATATATTG
CTTATGTAGCTTACCCCTTAGACCTTTTTGAAGAAGGCTCTGTTACTAAC
ATGTTTACTTCCATTGTGGGTAATGTGTTTGGATTCAAGGCCCTGCGCGC
TCTACGTCTGGAGGATTTGCGAATCCCTACTGCTTATACTAAAACTTTCC
AAGGCCCGCCTCATGGTATCCAAGTTGAGAGAGATAAATTGAACAAGTAC
GGCCGTCCCCTATTAGGATGTACTATTAAACCTAAATTGGGGTTATCCGC
TAAAAATTACGGTAGAGCTGTTTATGAATGTCTTCGCGGTGGACTTGATT
TTACCAAGGATGATGAGAACGTGAATTCCCAACCATTTATGCGTTGGAGA
GACCGTTTCTTATTTTGTGCCGAAGCCCTTTTTAAAGCACAGGCTGAAAC
AGGTGAAATCAAAGGGCATTATTTGAATGCTACTGCAGGTACATGCGAAG
AAATGATGAAAAGGGCAGNATTTGCCAGAGAATTGGGAGTTCCTATCGTA
ATGCACGATTACTTAACTGGGGGATTCACTGCAAACACTACCTTGGCTCA
TTATTGCCGAGATAATGGTCTACTTCTTCACATCCACCGTGCAATGCATG
CAGTTATTGATAGACAGAAAAATCATGGTATGCACTTCCGTGTACTGGCT
AAAGCGTTACGTATGTCTGGTGGAGATCATATTCACGCTGGTACTGTAGT
AGGTAAGCTTGAAGGGGAAAGAGAAATTACTTTAGGCTTTGTTGATTTAC
TACGTGATGATTATATTGAAAAAGATCGAAGCCGTGGTATTTATTTCACC
CAAGATTGGGTCTCTCTACCGGGTGTTCTTCCTGTGGCTTCAGGGGGTAT
TCACGTTTGGCATATGCCTGCTCTGACCGAGATCTTTGGAGATGATGCCG
TACTACAATTCGGCGGAGGAACTTTAGGGCACCCTTGGGGAAATGCACCT
GGTGCTGTAGCTAACCGAGTAGCTCTAGAAGCATGTGTACAAGCTCGTAA
TGAGGGGCGTGATCTTGCTCGTGAGGGTAATGAAATTATTCGTGAGGCTT
GTAAATGGAGTCCTGAACTAGCTGCTGCTTGTGAAGTATGGAAGGAGATC
AAATTTGAATTTGAAGCTATGGATACAATTTGA-----------------
-GACTTAATTGGATTGAGCCTTGGTATGGAAACCTACCAAGTGAGAACTT
TCAAATTCAGAGAAACCCTGGAATTAAAAATGGGCAATCCTGAGCCAAAT
CCAGTTTTCTGATTCTGAAAACAAACAAGG-----GTTC---A---GAAA

GCAATACAAAGGATAGGTGCAGAGACTCAATGGAAGCTGTTCTAACAAAT
GG----AGTTGGCTGCGTTGCGTTAGTAAAG--GAATCCTT-CCTTCCAT
CGAAACTTCCGAAAGGATGAAGAATACCCTATATATACGATATACGTACT
GAAATACTATCTGAAAAT-----GATTAATGAGGACCTGAATCTTTTTT-

--------------------------------TTTTTATAATTGATATGAAAAAA
GAATCTAATATTCATTGATCAAATCATTTACTCCATCATAATCTGAT---
-AGATCTTTTGAAGAATTGAT------TAATCGGACGAGAATAAAGATAG
AGTCCCATTCTACATGTCAA--------------TATCGACAACAATGAAAT
TTATAGTAAGAGGAAAATCCGTCGACTTTA-GAAATCGTGAGG-------

------------------TGCAGAATCCCGTGAACCATCGAGTCTTTGAACGC
AAGTTGCGCCCAAAGCCA--TCAGGTTGAGGGCACGTCTGCCTGGGCGTC
ACACACCGTTGCCCTCCCAACATCATGTTCCTTGAAGGATACT-------
------------TTGGTTGTGAAGGCGCATATTGGCTTCCCGTGAGCTTTGT
CTTG--TGGTTGGCCCAAA-A-TCTAGTCATTGGCAACTTGTGCCACGAC
AATG-GTGGTTGTCGAACCTTCG-GTGCCCCGTCGTGTGTTCTGGTTGTT
TGT-------GATGCAAGGATCCCA----ATGCATTCAATGT-GATGCCT
TTCGATGCGACCCCAGGTCAGGCGGGGCTACCCGCTGAGTTTAA------

>Dirachma_socotrana

TTGCATTTATTACGGCTTTTTCTTCACGAGTATTATAATTGGAGTAGTCT
TATT---------------------------------------ACTCCAAACAAAT
CTATTTTTTTAAAAAGAAATACAAGATTATTCTTGTTCTTATATAATTCT
CATGTTTGTGAATACGAATCCATCTTACT----TTTTCTCCGTAACCAAT
CTTATCATTTACCATTAACAGATTCTGGGGACTTTTTTGAGCGAATATAT
TTCTATGAAAAAATAAAACATCCTGTAAAAGAAATCTTTGCTAATGATTT
TTCGGCTATGCTATGGTACTTCAAGGATCCTTTCATACATTATGTTAGAT

ATCAAGGAAAATCCATTTTTGCTTCAAAAAATACGCCTCTTCTGATGAAT
AAATGGAAATATTTCTTTGTCCATTTATGGCAATGTCATTTTTATGTGTG
GTCTCAAACAGAAAAGATCTATATAAACCAATTATCCAAGTATTCCCTCG
GCTTTTTAGGCTATCTTTCAAGTATGCAACTCCATTTTTCAGTAGTACGG
AGTCAAATGCTAGAAAATTCATTTATAATGGATAATGCTATAAAGAAGCT
TGATATATCGGTTCTAATTGATCCAATGATCCAATCATTGGCTAAAATGA
AATTTTGTAATGCATTAGGACATCCCATTAGTAAGTCGACTTGGTCCGAT
TCATCGGATTTTGCTATTATCGACCGATTTTTGCGTATATGCAGAAATTT
TTCTCATTATTACAGTGGATCCTCAAAAAAAAAGATTTTATATCGAGTAA
AATATATACTTCGACTTTCTTGTGTTAAAACTTTAGCTCGTAAACACAAA
AGTACTGTACGCGCTTTTTTGAAAAGATTAGGTTCGGAATTTTTGGAAGA
ATTCTTTACGGAAGAAGAAGAAGTTCTTTCTTTGATTTTTCCACGAACTT
GTTCTACTTTGCGGAGATTATATAGAGGGCGAATTTGGTATTTGGATATT
TTTTGCATGAATGATCTGGTCAATCATGAATGA-----------------

-------------------------TGGGTTTTTCCTAGTGTTTTATT
ATTAAGTATAGTTATGGTTTTTTCAGCTTATCTATCTATTCAGCAAATAA
ATAATGGTTCTGTCTATCTATCTGTGTGGTCTTGGGCCATTAATAATGAT
TTTTCTTTAGAATTTGGCTACTTGGTTGATCCACTTACTTCTATTATGTT
AATATTAATCACTACTGTTGGAATTATGGTTCTTATTTATAGTGATAATT
ATATGTCTCATGATCAGGGATATTTGAGATTTTTTGCTTATATGAGTTTT
TTTAATACTTCAATGTTAGGATTAGTTACTAGTTCTAATTTGATACAAAT
TTATTTTTTTTGGGAATTGGTTGGAATGTGTTCTTATCTATTAATAGGTT
TTTGGTTCACACGACCTATTGCTGCGAATGCTTGTCAAAAAGCGTTTGTA
ACTAATCGCGTGGGGGACTTTGGCTTATTATTAGGAATTTTAGGTTTTTA
TTGGGTAACGGGTAGTTTAGAATTTCGGGATTTGTTTCAAATATTCAATA
ACTTGGTTTACAATAATGAAGTTAATTTTTTATTTGTTACTTTGTGTGCC
TTTCTATTATTCGCCGGCGCAGTTGCTAAATCTGCTCAATTTCCCCTTCA
TGTATGGTTACCCGATGCTATGGAAGGCCCTACTCCTATTTCGGCTCTTA
TACATGCTGCTACTATGGTAGCAGCGGGCATTTTTCTTGTTGCTCGACTT
CTTCCACTTTTCAGTATTATACCTTACATAATGAATCTAATAGCTTTGAT
AGGTATAATAACATTACTTTTGGGGGCCACTTTAGCTCTTGCTCAAAAAG
ATATTAAGAGAGGGTTAGCCTATTCTACAATGTCTCAATTGGGTTATATG
ATGTTGGCTCTGGGTATGGGTTCTTATCGAGCCGCTTTATTTCATTTGAT
TACTCATGCTTATTCGAAAGCATTGCTTTTTTTAGGATCCGGATCAATTA
TTCATTCAATGGAAACTATTGTTGGATATTCTCCAGATAAAAGCCAAAAT
ATGGTTCTTATGGGCGGTTTAAGGAAACATGTACCAATTACAAAAACTGC
TTTTTTATTAGGTACACTTTCTCTTTGTGGTATTCCACCCCTTGCTTGTT
TTTGGTCCAAAGACGAAATTCTTAATGATAGTTGGTTATATTCACCGATT
TTTGCAATAATAGCTTGTTCCACAGCAGGATTAACTGCATTTTATATGTT
TCGGATCTATTTACTTACTTTTGAAGGTCATTTAAACGTTAATTTTCAAA
CTTATAGCGGCAAAAAAAAATAGCTCGTTCTATTCAATATCTCTATGGGGT
AAAGAAGGGCAAAAAGAAAAAAAAAAAAAAAAATTCTTTATTAGTTTTATT
AACGATGAATA---------AGACCCCTTTTTTTTGTAAAAAGACATATC
GAATTGATAGTAATGTAAGAAAAATGACGCGACCTTTTCTT---ACTATT
ACTCCTGGGGCTGGGAGTACTAATAACATTTTATCATATCCTTATGAGTC
AGATAATACTATGTTATTTCCTATGCTTATATTAGTCCTATTTACTTTGT
TTATTGGAATCATAGGAATTCCTTTCTTCTTG---------ATTGAAGAA
AGGAATTTGGATATATTATCAAAATGGTTAACCCCATCTATAAACCTTTT
ACATCAAAATCAAGAAAATTATTTTGATTGGTATGAATTTATAATAAATG
CGACTTTTTCAGTCAGTATAGCTTCTTTAGGAATACTTATAGCAGCTTTT
TTATATAAGCCTGTTTATTCATCTTTAAAAAGTTTTGATTTTATTAATTC
ATTTGTCAAATTTTTAAAAAGTATTCCTAATAACATTAAAATCATTGGGG
ATAAAATAAAAAATATTATATATAATTGGTCAGCTAATCGGGGGTATATA
GATGTTTTTTATTCAATATCCTTAACTCAAGGTATAAGAGGGTTAGCTAA
ACTGA---

-------------------------------AAGTGTTGGATTCAAAGCT
GGTGTTAAAGATTATAAATTGACTTATTNNNCCCCTGAATATGAAACCAA
AGATACTGATATCTTGGCAGCCTTTCGAGTAACTCCTCAACCTGGAGTTC
CGCCTGAAGAAGCAGGAGCCGCGGTAGCTGCTGAATCTTCTACTGGTACA
TGGACAACTGTATGGACCGATGGGCTTACCAGTCTTGATCGTTACAAAGG
TAGATGCTACCACATTGAGCCCGTTCCTGGAGAAGAAAGTCAATTTATTG
CCTATGTAGCTTACCCCTTAGACCTTTTTGAAGAGGGTTCTGTTACTAAC
ATGTTTACTTCCATTGTGGGTAATGTATTTGGGTTCAAGGCTCTGCGCGC
TCTACGTCTGGAGGATTTGCGAATCCCTACTGCTTATATTAAAACTTTTA
TAGGACCGCCTCATGGTATCCAAGTTGAGAGAGATAAATTGAACAAGTAT
GGACGNCCCCTATTGGGATGTACTATTAAACCTAAATTGGGGTTATCCGC
TAAGAATTACGGTAGAGCAGTTTATGAATGTCTTCGCGGTGGACTTGATT
TTNCCAAAGATGATGAGAACGTGAATTCCCAACCATTTATGCGTTGGAGA
GACCGTTTCTTATTTTGCGCCGAAGCAATTTATAAATCGCAGGCAGAAAC
GGGTGAAATCAAAGGGCATTACTTGAATGCTACTGCAGGTACATGCGAAG
AAATGATCAAAAGGGCTGTATTTGCAAGAGAATTGGGAGTTCCTATCGTA
ATGCACGATTACTTAACAGGGGGATTCACTGCAAATACTAGCTTGGCTTA
TTATTGCCGAGATAATGGTCTACTTCTTCACATCCACCGTGCAATGCATG
CAGTTATTGATAGACAGAAAAATCATGGTATGCACTTTCGTGTACTAGCT
AAAGCGTTACGTCTGTCTGGTGGAGACCATATTCACGCCGGTAGTGTAGT
AGGTAAACTTGAAGGGGAAAGAGACATCACTTTAGGTTTTGTTGATTTAC
TACGTGATGATTATATTGAAAAAGACCGAAGCCGTGGTATTTATTTCACT
CAAGATTGGGTCTCTACACCAGGTGTTTTNCCCGTNNNTTCAGGGGGTAT
TCACGTTTGGCATATGCCTGCTCTGACCGAGATCTTTGGAGATGATGCCG
TACTACAATTCGGCGGAGGAACTTTAGGGCACCCTTGGGGAAATGCACCC
GGTNCCGTAGCTAATCGAGTAGCCCTAGAAGCATGTGTAAAGGCTCGTAA
TGAGGGACGCGATCTTGCTCGTGAGGGTAATGAAATTATCCGTGAGNCTA
GTAAATGGAGTCCTGAACTAGCTGCTGCTTGTGAAGTATGGAAGGGAATC
AAATTTGAATTTGAAGCAGTGGATACATTTGAA-----------------
---------------------TGGTATGGAAACCTACCAAGTGATAACTT

TCAAATTCAGAGAAACCCTGGAATTAAAAATGGGCAATCCTGAGCCAAAT
CCCGTTTTCTGAAAACAAAGAAAGGTTAAGAATTCAGAAAACG---ATAT
AAGAAAAAAAGGGATAGGTGCAGAGACTCAATGGAAGCTGTTCTAACAAAT
GG----AGTTGGTTGCGTTGAGTT------AGTAACGGAAT-CCTTCCTT
CAAAAGTCCATAAAGTATGAAGAATAAACCTATATATAC------GTACT
GAAATATTATCTCCAAAT-----GATTAATGACGACCAAAATAAAATCTA
TATTTTTTTATATTTATTTATAGGTATAGGACAAATCAAATATAGGACAA
ATGAAAGAATTGTTGTGAAGCGATTTCTCAATCAAGTCAAGTTGAAAAAA
GAATCGAATATTCATTAATCAAATCATTTACTCCATCATAATCTGAT---
-AGATCTTTTGAAGAATTGAT------TAATCGGACGAGAATAAAGATAG
AGTCCCACTCTACATGTCAA------------TATCGACAACAATGAAAT
TTATAGTAAAAGGAAAATCCGTCGACTTTTAGAAATCGTGAGGGTTCAAG
TCCCTCTATCCCCAAAAGAGG-ATTCCCTAATTATTTA------------
-----TCTTATCCTCTCATTTCTTTAGCGATTAAAAATTTGCTATGTTTC
TCACTCATTCTAATTCTACTTTTTCACAAACTCACAAACGGAGGGAAAAT
---TTTTTTCTTATCACAT-----AT----CATATATATGATACACGTAC
AAATGATACACGTACAAATGAACATCTTTGAGCAAACAAGTAATCCCGAT
TTTAAAATGG-------AATGATT-AACAATACATATCATTACCCGTACT
GTACTGAAA----GTTACAAAGTCT-------TCGTTTTGAAGATCCAAG
AAATT----CCATCAGGGACTGGATAATACTTTGTAAT----TCG-----
--TCTTTTTAATTGACATAGACCCCAGTCTTTCATATA--TTAAAATGAA
GATGATTTGTCGTG-----AATGGTCGGGTAG-CTCAGCTGGT-------

>Barbeya_oleoides

-------TATTACGTCTTTTTCTTCATGAGTATTATCATTTGAATAGTTT
TAT---------------------------TACTCCAAAAAAATTGATTT
CTATTTTTTCAAAAAGTAATTCAAGATTATTCTTGTTCCTATATAATTCT
CATGTTTGTGAATACGAATCCATCTTACT----TTTTTTTCGTAACCAAT
CTTCTCATTTACGATTAACATCTTCTAGGGGCTTTTTTGAGCAAATCCAT

TTCTATGGGAAAATAAAACATCCTGTCAAAAAAGTCTTTGTTAATGATTT
TCCGTCTATCCCATGGCTCTTTAAGGATTCTTTCATGCATTATGTTAGAT
ATCAAGGAAAATCCATTTTGGCTTCAAACGATATGCCTCTTCTGATGAAT
AAATGGAAATATTACCTTGTCCATTTATGGCAATGTCATTTTTATGTGTG
GTCTCAATCAGGAAGGATCCGTATAAACCAATTATCCAAGCATTCCCTCG
GCTTTTTGGGCTATCTTTCAAGTATGGGACTCAATTTTTCAGTGGTACGG
AGTCAAATGCTAGAAAATTCATTTTTAATGGATAATGCTATGAAGAAACT
TGATACATTAGTTTCAATTAGTCCTCTGATTGGATCATTGGCTAAAATGA
AATTTTGTAACGCATTAGGATATCCTGTTAGTAAGTCGACCTGGGCGGAT
TCGTCGGATTTTTATATTATCGACAGATTTACGCGTATATGCAGAAATCT
TTCTCATTATTATAGTGGATCCTCAAAAAAAAAAGAGTTTGTATCAAATAA
AATATATACTTCGACTTTCTTGTGTTAAAACTTTGGCTCGTAAACACAAA
AGTACTGTACGTGCTTTTTTGAAAAGATTTGGTTCGGAATTATTTGAAGA
ATTCTTTATGGAGGAAGAAGAGGTTCTTTCTTGGATTTTTCCAAGAACTT
ATTCTATTTT------TTTATATAGAGGGCGAATTTGGTATTTGGATATT
TTTTGCATCAATGATCTAGTCAATCATGAATGA----------------

------------------------------------CTAGTGTTTTATT
GTTAAGTATAGTTATGCTTTTTTCAGCCTATCTATGTATTCAGCAAATAA
ATAACAGTTCTATCTATCTATATGTATGGTCTTGGACCATCAATAATGAT
TTTTCTTTAGAATTTGGTTACTTGATTGATCCACTTACTTCTATTATGTT
AATATTAATCACTACTGTTGGAATTATGGTTCTTATTTATAGTGATAACT
ATATGTCTCATGATCAGGGGTATTTGAGGTTTTTTGCTTATATTAGTTTT
TTTAATACTTCAATGTTAGGATTAGTTACTAGTTCTAATTTGATACAAAT
TTATTTTTTTTGGGAATTAGTTGGAATGTGTTCTTATCTATTAATAGGTT
TTTGGTTCACACGACCTATTGCAGCGAATGCTTGTCAAAAAGCATTTGTA
ACTAATCGCGTAGGGGATTTTGGTTTATTATTAGGAATTTTAGGTTTTTA
TTGGATAACGGGTAGTTTAGAATTTCGGGATTTGTTTGAAATATTCAAAA
ATTGGATTTATAATAATGAAGTTAATGTTTTATTTGTTATTTTGTGTGCC
CTTCTATTATTTACCGGTGCAGTTGCTAAATCTGCCCAATTTCCCCTTCA
TGTATGGTTACCCGATGCGATGGAAGGACCTATCCCTATTTCGGCTCTTA
TACATGCAGCTACTATGGTAGCGGCTGGAATTTTTCTTGTAGCTCGTCTT
CTTCCGCTTTTCGTAGTTATACCTTACATAATGAATATAATAACTTTGAT
AGGTACAATAACATTACTTTTAGGAGCCACTTTAGCTCTTGCTCAAAAAG
ATATTAAGAGAGGTTTAGCTTATTCTACAATGTCTCAATTGGNNNNNNNN
NNNNNNNNNNNNNNNNNNNNNNNNNNNNNNNNNGCTTTATTTCATTTGAT
TACTCATGCTTATTCAAAAGCATTGTTGTTTTTAGGATCCGGATCAATTA
TTCATTCAATGGAAACTATTGTTGGATATTCTCCAGATAAAAGCCAGAAT
ATGGTTCTTATGGGTGGTTTAAGAAAACATGTACCAATTACAAAAATTGC
TTTTTTATTAGGTACACTTTCTCTTTGTGGTATTCCACCCCTTTCCTGTT
TTTGGTCCAAAGATGAAATTATTAATGATAGTTGGTTATATTCACCGATT
TTTGCAATAATAGCTTATTCCACAGCAGGATTAACTGCATTTTATATGTT
TCGGATCTATTTACTTACTTTTGAGGGTCATTTAAACGTTAATTTTCAAA
TGTACAGTGGAAAAAAAAAATAGCTCATTCTATTCAATACCTCTATGGGGT
AAAGAAGGATCAAAAGTAATTAAAAACAAAAATTGCTTATTCACTTTATT
AACAATGAATAAAAATGAAAAGACTTCTTTTTTTTTTAAAAAGATATATA
GAATTGATAGTTATGTAAGACACATA---------GCACGTCCTTTTCTT
ACTATTACTCATTTTGATATTAAGAATATTTTTGCGTATCCCCGGGAGTC
GGACAATACTATGCTATTTCCTATGCTTGTATTAGCCCTATTTACTTTGT
TCATTGGAGTTATAGGAATTCCTTTCTTCAATCAATTCAATCAAGAAGTA
ATGTATTTAGATATATTATCAAACTTGTTAACTCCGTCTATAAACCTTTT
ACATCAAAATAAAAAAGATTCTGTGGATTGGTATGAATTTATGACAAAGG
CAACTTTTTCAGTTAGTATAGCTTATTTGGGAATCCTTATTGCGTCTTTT
TTATATAAACCTGTTTATTCATCTTTACAAAATTTGAATTTCTTTAATTA
TTTTGTTA---------AAAGTATTTCTAATAAAATTCAAATTGTTTTGA
ATAAAAAAATAAATGTGATATATGATTGGTCATATAATCGCGGTTACATA
GATGTTTTTTATACGATATCCTTAACTGAAGGGATAAG------------

-------------------------------AAGTGTTGGATTCAAAGCT
GGTGTTAAAGATTATAAATTGACTTATTACACTCCTGACTATGAAACTAA
AGATACTGATATCTTGGCAGCATTTCGAGTAACTCCTCAACCTGGAGTTC
CACCTGAGGAAGCAGGGGCCGCGGTAGCGGCTGAATCTTCTACTGGTACA
TGGACAACCGTATGGACTGACGGGCTTACCAGTCTTGATCGTTACAAAGG
TCGATGCTACCACATCGAGCCCGTTGCTGGAGAAGAAAGTCAATTTATTG
CTTATGTAGCTTACCCTTTAGACCTTTTTGAAGAAGGTTCTGTTACTAAC
ATGTTTACTTCCATTGTCGGTAATGTATTTGGGTTTAAGGCCCTGCGCGC
TCTACGTCTGGAAGATTTGCGAATCCCTCCTGCTTATGTTAAAACTTTCC
AAGGCCCGCCTCATGGCATCCAAGTTGAGAGAGATAAATTGAACAAGTAT
GGCCGCCCCCTATTGGGATGTACTATTAAACCTAAATTGGGGTTATCTGC
TAAGAATTACGGTAGAGCTGTTTATGAATGTCTTCGCGGTGGACTTGATT
TTACCAAAGATGATGAAAACGTGAATTCCCAACCATTTATGCGTTGGAGA
GACCGTTTCTTATTTTGTGCTGAAGCCATTTATAAAGCACAGGCTGAAAC
AGGTGAAATTAAAGGGCATTACTTGAATGCTACTGCGGGTACATGCGAAG
AAATGATCAAAAGGGCTGTATTTGCAAGAGAATTGGGCGTTCCTATCGTA
ATGCATGATTACTTAACTGGGGGATTCACTGCAAATACTAGCTTGGCTCA
TTATTGCCGAGATAATGGTNNNNNNNNNNNNNNNNNNNNNNNNNNNNNNN
NNNNNNNNNNNNNNNNNNNNNNNNNNNNNNNNNNNNNNNNNNNNNNNNNN
NNNNNNNNNNNNNNNNNTGGTGGAGATCATATTCACGCTGGTACTGTAGT
AGGTAAACTTGAAGGAGAAAGAGACATCACTTTAGGCTTTGTTGATTTAC
TACGTGATGATTTTATCGAAAAAGATCGAAGCCGTGGTATTTATTTCACT
CAAGATTGGGTCTCTATACCAGGTGTTTTGCCCGTGGCTTCAGGGGGTAT
TCACGTTTGGCATATGCCTGCTCTGACCGAAATCTTTGGAGATGATTCCG
TACTACAATTCGGCGGAGGAACTTTAGGGCACCCTTGGGGAAATGCACCC
GGTGCCGTAGCTAATCGAGTAGCTCTAGAAGCATGTGTACAAGCTCGTAA
TGAGGGACGTGATCTTGCTCGTGAGGGTAATGAAATTATCCGTGAAGCTA
GTAAATGGAGTCCTGAACTAGCTGCTGCTTGTGAAGTATGGAAGGAGATC

AAATTTGAATTTGCAGCAGTGGATACTTTGTAA-----------------
---------------------TGGTATGGAAACCTACCAAGTGAGAACTT
TCAAATTCAGAGAAACCCTGGAATTAAAAATGGGCAATCCTGAGCCAAAT
CCTGTTTTATGAAAAAAACAAAG--GTTCAGAAAGCGA------------
TAATAAAAAAGGATAGGTGCAGAGACTCAATGGAAGCTGTTCTAACAAAA
GG----AGTTGGCTGCGTTGCGTT------AGTAAAGGAAT-CCTTCCAG
TGAAACTTCAGAAAGGATGAAGAATAAACCTATATATACGTACTG-----
-AAATACTATCTCC-----AAATAATTACTGACGACCCGAATCTGTTTT-
-TATATATTTAGATAAAA-------------------------------A
ATGAAAGAATTGTTGT----------GAATCGATTCCTACTTGAAAAAAA
GAATCGAATATTCATTGATCAAATCATTTATTCCATCATAATCTGAT---
-AGATCTTTTGAAGAATTGAT-----AAATCCGGACGAGAATAAAGATAG
AGTCCCATTCTACATGTCAA------------TATCGACAACAATGAAAA
TTATAGTAAGAGGAAAATCCGTCGACTTTA-GAAATCGTGAGGGTTCAAG
TCCCTCTATCCCCAAAAAGCC-CTATTTGATTCCCTAATTATTTATC---
-------CTATCTTCTCATTTCGTTAGCGATTCAAAATTCGCTATGTTTC
TCATTCATTCGAATTTTACTCTTTCACAAACGAGTCTGAACGGGTCTGGG
CGGAAAT----TTGTTTATTATCAC-----AAATCTTGGGATATATATGA
TTATGATATACGTACAAATGAACATCTTTGAGCAAGC----AATCCCGAT
TTCAAATTTA-------AATGATT-AACAATAGATATCATTAGTCGTACT
GTACTGAAAGTTAGA----AAGTC------TTTTTTTTTGAAGATACAAG
AAATT----CCTCCAGGGCCCGAATAAGACTTTGTAATCCACT-----TT
TCATCTTTTAATTGACATAGACCCAAGTCATCTATTAAA------ATGAG
GATGATGCGTCGTGAATTGAATGGTCGGGATAGCTCAGCTGGT-------

>Phylica_rubra

-------------------------------CTTGTTCCTATATAATTCT

CATGTTTGTGAATACGAATCCATCTGACTTTTTTTTTCTCCGTAACCAAT
CCTCTCACTTACGATTAACATCTTCCGGGGTCTTTTTTGAGCGAATATGT
TTCTATGGAAAAAGAAAACATCCTGTAGAAGAAGCCTTTTCTAATTCTTT
TCCGGCGATCTTATGGTTCTTCACGGATCCTTTCATGCATTATGTTAGAT
ATCAAGGAAAATCCACTTTGGCTTCAAAAGATACGCCTCTTCTAGTGAAT
AAACGGAAATGTTACCTTGTCCTCTTATGGAAATGTCATTTCTATGTGTG
GGCTCANCCAGGAAGGATCCATATAAACCAATCAGCCAATCGCTCCTTCG
GCTTTTTGGGCCATCTTTCAAGGGTGCGACTCAATCTTTCAGTGGTA---
------CGGCTAGAAAATTCATTTATAATGGATAATGCTATGAAGAAGCT
TGATACATTAGTTCCAATTAGTCCAATGATTGGATCATTGGCTAAAATGA
AATTTTGTAACGCATTAGGACATCCTGTTAGTAAGTCGACCTGGGCCGAT
TCGTCAGATTTTGATATTATCGACCGATTTTCCCGTATATGCAGAAATCT
TGATCATTATTCCAGC----------------------------------

-----------------CTACTTAATTGATCCACTTACTTCTATTATGTT
AATATTAATCACTACTGTTGGAATTATGGTTCTTATTTATAGTGATAATT
ATATGTCTCATGATCAGGGATATTTGAGATTTTTTGCTTATATGAGTTTT
TCTAATACTTCAATGTTAGGATTAGTTACTAGTTCAAATTTGATACAAAT
TTATTTTTTTTGGGAATTGGTTGGAATGTGTTCTTATCTATTAATAGGTT
TTTGGTTCATCCGGCCTATTGCGGCGAATGCTTGTCAAAAGGCGTTTGTA
ACTAATCGTGTAGGGGATTTTGGTTTATTATTAGGAATTTTAGGTTTTTA
TTGGATAACGGGTAGTTTAGAATTTAGGGATTTGTTTGAAATATTCAATA
ACGTGGTTGATAATAATGAAGTTGATTTTTTATTTGTTACTTTGTGTGCC
TGTCTATTATTTACCGGTGCAGTTGCTAAATCTGCCCAATTTCCCCTTCA
TGTATGGTTACCCGATGCTATGGAAGGACCTACCCCTATTTCGGCTCTTA
TACACGCCGCTACTATGGTAGCAGCCGGAATTTTTCTTGTAGCTCGGCTT
CTTCCGCTTTTCATAGTTATACCTTTCATAATGAATCTAATAGCTTTTAT
TGGGATAATAACATTACTTTTAGGAGCC----------------------

---------------------------GTCGAAGTTCCGTCTACAAACGG
ATAAGACTTCG--------TTATTAGTGTATACTGAGTTATTGAACATAA
AGGAGCAATAATCAATTTCTTGTTCTAGCAAGAAGTTTGGTATTGCTCCT
TTT-TTATTTATTTATTTAGGAGTTTTT-TTACTTAA-ATCAGTTTTTTT
TTCTTTACTTCATCTTTACTTCAACATACCCATATTTTCGTTATATAGAA
ATTTTTATTTTCGATTAAATGTTTCGATTCCATTTTT-------------

----TTTCTTGAAGAATGAACAAAAAAGATAAATGGT----GTAAATTGA
CCTATTTGTTTGTA----------ATTTTT----------AATTTAATAG
GGGCGGATGTAGCCAAGTGGATCAAGGCAGTGG-----------------

--------------------------------AGTGTTGGATTCAAAGCC
GGCGTTAAAGATTATAAATTGACTTATTACACTCCTGACTATGAAACCAA
AGATACTGATATCTTGGCAGCGTTTCGAGTAACTCCTCAACCTGGAGTTC
CGCCTGAGGAAGCAGGGGCCGCGGTAGCTGCTGAATCTTCTACTGGTACA
TGGACAACTGTATGGACTGACGGGCTTACCAGTCTTGATCGTTACAAAGG
TCGATGCTACCACCTAGAGCCCGTTGCTGGAGAAGAAAATCAATATATTG
CTTATGTAGCTTACCCCTTAGACCTTTTTGAAGAAGGTTCTGTTACTAAC
ATGTTTACTTCCATTGTGGGTAATGTTTTTGGGTTCAAAGCCCTGCGCGC
TCTACGTCTGGAGGATTTGCGAATCCCTCCTGCTTATTCTAAAACTTTCC
AAGGACCGCCTCACGGCATCCAAGTTGAAAGAGATAAATTGAACAAGTAT
GGCCGCCCCCTATTGGGATGTACTATTAAACCTAAATTGGGGTTATCCGC
TAAGAATTACGGTAGAGCAGTTTATGAATGTCTT----------------

--GT
GAC--------------ACGAGGATTTTCAGTCCTCTGCTCTA-------
-CCAGCTGAG-CTATCCCGACCATTCACGACGCATCATCC----------
--TCAT-TTTAATAGATGACTTGGGTCTATGTCAATTAAAAAGACGAAAA
GGGGATTACAAAGTCTTATCCATGCCTTGGTGGAATTTCTTAGATCTTCA
A-----------------------------AA---AGAAGA-CTTTG---
TAAGTTTCAATACAGTACGGGCAATAATATGATCA---------------

----------------------------------TTCCAATTT-AC----
------AAT---------CGGGGTTACTTGCTAAATGATGTTCATTTGTA
CGTGTATCATATATATCGGCT---TGTGAAAATCAAAAAATTAATGAATG
AGAAATATAACGAATTTTGAACCGCTAAC---GAAATGAGAGTATAGGAT
AAAAAATTAGGGAATCAAATG--G-GTG---GGG--ATAGAGGGACTTGA
ACC---

--CATT
TAGAGGAAGGAGAAGTCGTAACAAGGTTTCCGTAGGTGAACCTGCGGAAG
GATCATTGTCGAAA-CCT-GCTCAGCAGAACGACCCGCGAACCTGTGAA-
AACATACTGGGA---GCGAGG---GACCTTTGGT--CTC-GAACTCTCT-
TGGTCGG-GGGGGCTG---CATCCCGTGCATTGCTTGACGATGCATTGGT
GTTG--CTTTCCAGGCCGC-ACAAACGAACCCCGGCGCCAAATCG--CGC
CAAGGAAAATCTAACGAATTGGCAC-TGCCCCTCCGCCCCAGAGATGGTG
TGCGGTTGGGGTGTGCGT--CGTATTC---TATATGTCAAAACGACTCTC
GGCAACGGATATCTCGGCTCTCGCATCGATGAAGAACGTAGCGAAATGCG
ATACTTGGTGTGAATTGCAGAATCCCGTGAACCATCGAGTCTTTGAACGC
AAGTTGCGCCCGAAGCCA--TACGGCCGAGGGCACGTCTGCCTGGGCGTC
ACACAACGTTGCCCC-CCAACCTC----AAAG------------------
-------------GTGAGGGGGGCGGATGCTGGCCTCCCGTGTGCTACGG
CATG--CGGCTGGTTTGAAT--ACGAGTCCTCGGCGACGAGTGCCGCAAC
AATCGGTGGTTCTCCAACCCTCG-TGGCCCTGTTGCGTGCATAGACCGCC
GATGTGGCCCGATAGACC---CCAC-TG-CGT---TGCTAATGCAGCGTA
TCCAACGCGACCCC------------------------------------

>Phylica_arborea

-----------TTTTCTTCACGAGTNTTATAATTG-------GAATCGTN
TTATTACTCCAAANAAATCTATTTCTTTT--TTTCTTTTAAGTAGTTCAA
GATTTTTCTTGTTCCTATATAATTCTCATGTTTGTGAATACGAATCCNNN
NNNNNNNGGCTAGAAAATTCATTTATAATGGATAATGCTATGAAGAAGCT
TGATACATTAGTTCCAATTAGTCCAATGATTGGATCATTGGCTAAAATGA
AATTTTGTAACGCANTAGGACATCCTGTTAGTAAGTCGACCTGGGCCGAT
TCGTCAGATTTTGATATTATCGACCGATTTTCCCGTATATGCAGAAATCT
TTCTCATTATTCCAGCGGATCCTCAAAAAAA-------------------

-------------ATCTTCGCCGTATGTGGCTTTTTCCTAGTGTTTTACT
GTTAAGTATAGTCATGATTTTTTCAGCCCATATATTTATTCAACAAATCA
ATAACAGTTCTGTTTATCTATCTGTATGGTCTTGGACCATCAATAATGAT
TTTTCTTTAGAATTTGGCTACTTAATTGATCCACTTACTTCTATTATGTT
AATATTAATTACTACTGTTGGAATTATGGTTCTTATTTATAGTGATAATT
ATATGTCTCATGATCAGGGATATTTGAGATTTTTTGCTTATATGAGTTTT
TTTAATACTTCAATGTTAGGATTAGTTACTAGTTCAAATTTGATACAAAT
TTATTTTTTTTGGGAATTAGTTGGAATGTGTTCTTATCTATTAATAGGTT
TTTGGTTCATCCGGCCTATTGCGGCGAATGCTTGTCAAAAGGCGTTTGTA
ACTAATCGTGTAGGGGATTTTGGTTTATTATTAGGAATTTTAGGTTTTTA
TTGGATAACGGGTAGTTTAGAATTTAGGGATTTGTTTGAAATATTCAATA
ACGTGGTTGATAATAATGAAGTTGATTTTTTATTTGTTACTTTGTGTGCC
TGTCTATTATTTGCCGGTGCAGTTGCTAAATCTGCCCAATTTCCCCTTCA
TGTATGGTTACCCGATGCTATGGAAGGACCTACCCCTATTTCGGCTCTTA
TACACGCCGCTACTATGGTAGCAGCCGGAATTTTTCTTGTAGCTCGGCTT
CTTCCGCTTTTCATAGTTATACCTTTCATAATGAATCTAATAGCTTTTAT
TGGGATAATAACATTACTTTTAGGGGCCACTTTAGCTCTTGCTCAAAAAG
ATATTAAGAGGGG-------------------------------------

-ATAACTTCCCTCTAGATCTAGCTGCTGTCGAAGTTCCGTCTACAAACGG
ATAAGACTTCG--------TTATTAGTGTATACTGAGTTATTGAACATAA
AGGAGCAATAATCAATTTCTTGTTCTAGCAAGAAGTTTGGTATTGCTCCT
TTT-TTATTTAG--------GAGTTTTT-TTACTTA--AATAAGTTTTTT
TTCTTTACTTCATCTTTACTTCAACATACCCATATTTTCGTTATATAGAA
ATTTTTATTTTCTATTAAATGTTTCAATTCCATTTTTT------------

----TTTCTTGAAGAATGAACAAAA----AAAATGGT----GTAAATTGA
CCTATTTGTTTGTA----------ATTTTT----------AATAG-----
GGGCGGATGTAGCCAAGTGGATCAAGGCAGT-------------------

-------------------------------AAGTGTTGGATTCAAAGCC
GGCGTTAAAGATTATAAATTGACTTATTACACTCCTGACTATGAAACCAA
AGATACTGATATCTTGGCAGCGTTTCGAGTAACTCCTCAACCTGGAGTTC
CGCCTGAGGAAGCAGGGGCCGCGGTAGCTGCTGAATCTTCTACTGGTACA
TGGACAACTGTATGGACTGACGGGCTTACCAGTCTTGATCGTTACAAAGG
TCGATGCTACCACCTAGAGCCCGTTGCTGGAGAAGAAAATCAATATATTG
CTTATGTAGCTTACCCCTTAGACCTTTTTGAAGAAGGTTCTGTTACTAAC
ATGTTTACTTCCATTGTGGGTAATGTTTTTGGGTTCAAAGCCCTGCGCGC
TCTACGTCTGGAGGATTTGCGAATCCCTCCTGCTTATTCTAAAACTTTCC
AAGGACCGCCTCACGGCATCCAAGTTGAAAGAGATAAATTGAACAAGTAT
GGCCGCCCCCTATTGGGATGTACTATTAAACCTAAATTGGGGTTATCCGC
TAAGAATTACGGTAGAGCAGTTTATGAATGTCTT----------------

-------------ATTGAGCCTTGGTATGGAAACTACCAAGTGATAGCTT
TCAAATTCAGAGAAACCCTGGAATTACAAATGGGCAATCCTGAGCCAAAT
CCTGTTTTCTGAAAACAAACAAGGATTCA-------GAA---A---GTGA
TAATAAAAAAGGATAGGTGCAGAGACTCAATGGAAGCTGTTCTAACAAAT
GG----AGTTGGCTGCGATGCGTT------AGTAAAGGAAT-CCTTCCAT
CGAAATTCCAGAAAGGAGGAAGGATAAACGTATATATACGTATACGTACG
GAAATACTGTCTCCAAACAAAATGATTAATGACGACCCGAATCTTTTTTT
TTTTT--TTAT------TTATATGTTTATATGAAA--------------A
ATGAAAAAATTGTTGTGAA-----TCGATTCCAAGTAAA--------AAA
AAATGGAATATTCGTTGATCAAATCATTTACTCCTTCGTAATCTGAT---
-AGATCTTTTGAAAAATTGAT------TAATCGGACGAGAATAAAGATAG
AGTCCCATTCTACATGTCAA------------TATCGACAACAATGAAAT
TTATAGTAAGAGGAAAATCCGTCGACTTTA-GAAATCGTGAGGGTTCAAG
TCCCTCTATCCCCA------C-CCATTTGATTCCCTAATTTTTTATC---
--CTATACT-C----TCATTTCGTTAGCGGTTCAAAATTCGTTATGTTTC
TCATTC------ATTAATT-TTTT--------------------------
------GAT--TTTCACAAGCCTTGT----GATATATATGATACACG---
----TAC--------AAATGAACATCA--TTTAGCAAGT--AACCCCGAT
TGTAAATTGG-------AATGATC-ATATT--------ACCGCTCGTACT
GTATTGAAA----CTTACAAAGTCTTCTTT----TTG---AAGATCTAAG
AAATT----CCACCAAGGCCTGGATAAGACTTTGTAAT----CCCCTTTT
CGTCTTTTTAATTGACATAGACCCAAGTCATCTATTAA------AATGAG
GATGATGCGTCGTG-----AATGGTCGGGATAGCTCAGCTGGTAGAGCAG
AGGACTGA--------------------------------CGGCGACGTG
GGCGGTTCGCTGCCTGCGACGTCGCGAGAAGTCCACTGAACCTTATCATT
TAGAGGAAGGAGAAGTCGTAACAAGGTTTCCGTAGGTGAACCTGCGGAAG
GATCATTGTCGAAA-CCT-GCTCAGCAGAACGACCCGCGAACCTGTCAAA
-ACATACTGGGA---GCAAGA---TGCCTTAGGT--CTC-GAACTCTCT-
TGGTCGG-GGGGGCTG---CATCCCGTGCATCGCTTGCCGATGCATTGGT
GTTG--CTTTCCAGGCCGC-ACAAACGAACCCCGGCGCCAAATCG--CGC
CAAGGAAAATCTAACGAATTGGCAT-TGCCTCTCCGCCCCAGAGATGGTG
TGCGGTTGGGGTGTGCGT--CGTATTC---TATATGTCAAAACGACTCTC
GGCAACGGATATCTCGGCTCTCGCATCGATGAAGAACGTAGCGAAATGCG
ATACTTGGTGTGAATTGCAGAATCCCGTGAACCATCGAGTCTTTGAACGC
AAGTTGCGCCCGAAGCCT--TCTGGCCGAGGGCACGTCTGCCTGGGCGTC
ACACAATGTTGCCCCCCCAACCTC----AAAG------------------
-------------GTGAG-GGGGCGGATGCTGGCCTCCCGTGTGCCACGT
CATG--CGGCTGGTTTGAAT--ATGAGTCCTCGGCGACGAGTGCCGCAAC
AATCGGTGGTTCTCCAACCCTCG-TGGCCCTGTTGCGTGCATAGACTGCC
GTTGTGGCTCGATAGACC---CCAC-TG-CGT---TGCTAATGCAGCGTA
TCCAACGCGACCCCAGGTCAGGCGG-------------------------

>Helinus_integrifolius

--TCTTCT
TTGCATTTATTACGACTTTTTCTTCACGAGTATTATAATTGGAATAGTTT
TATTAATTGGAATAATTGGAATAGTTTTATTACTCCAAAAAAATCTATTT
CTTTTTTTTTGAAAAGTAATTCAAGATTTTTTTTGTTCCTATATAATTCT
CATGTTTGTGAATACGAATCCATCTTACT----TTTTCTCCGTAACCAAT
CTTCTCATTTACGATTAACATCTTCTGGGGTATTGTTTGAGCGACTTTTT
TTCTATGGCAAAATAAAACATCTTGTCGAAGAAGTATTTTCTAACGCTTT
TCCGGCGATCTTATGGTTCTTCACAGAGCCTTTCATGCATTATGTTCGAT
ATCAAGGAAAATCTATTTTGATTTCAAAAGATACGCCTCTTCTAATGAAT
AAATGGAAATATTATCTTGTCCTTTTCTGGCAATGTTTTTTTTATGTATG
GGCTCAACCAGGAAGGATCTATATAAACCAACTAGCCAAGCATCCCTTCA
GCTTTTTGGGCTATCTTTCAAGGGTGCGACTCAATTTTTCAGTGGTACGG
AGTCAAATGCTAGAAAATTCATTTATAACGGATAATGCTATAAAGAAACT
TGATACATTAGTTCCAATTAGTCCAATGATTGGATCGTTGGCTAAAATGA
AATTTTGTAACGCATTAGGACATCCTGTTAGTAAGTCGTCCTGGGCCGAT
TCGGCAGATTTTGATATTATCGACCGATTTGCCCGTATAGGACGAAATCT
TTCACATTATTCCAGCGGATCCTCAAAAAAAAAGAATTTGTAT-------

----------------------------AGCAAGTGTGGGATTCATAGCC
GGTGTTAAAGATTATAAATTGACTTATTACACTCCTGAATATGAAACCAA
AGATACTGATATCTTGGCAGCGTTTCGAGTAACTCCTCAACCTGGAGTTC
CGCCTGAGGAAGCAGGGGCAGCGGTAGCTGCTGAATCTTCTACTGGTACA
TGGACAACTGTATGGACTGACGGGCTTACCAGTCTTGATCGGTACAAAGG
TCGATGCTACGGGCTTGAGCCCGTTGCTGGAGAAGAAAATCAATATATTG
CTTATGTAGCTTACCCCTTAGACCTTTTTGAAGAGGGTTCTGTTACTAAC
ATGTTTACTTCCATTGTGGGTAACGTTTTTGGGTTCAAGGCCCTGCGCGC
TCTACGTCTGGAGGATTTACGAATCCCTCCTGCGTATACTAAAACTTTCC
AAGGACCGCCTCATGGCATCCAAGTTGAAAGAGATAAATTGAACAAGTAC
GGCCGCCCACTATTGGGATGTACTATTAAACCTAAATTGGGGTTATCCGC
TAAGAATTACGGTAGAGCAGTTTATGAATGTCTTCGCGGTGGACTTGATT
TTACCAAAGATGATGAGAACGTGAATTCCCAACCATTTATGCGTTGGAGA
GACCGTTTCTTATTTTGTGCCGAACCACTTTATAAAGCACAGGCTGAAAC
AGGTGAAATCAAAGGGCATTACTTGAATGCTACTGCAGGTACATGCGAAG
AAATGATTAAAAGGGCTGTATTTGCCAGAGAATTGGGAGCTCCTATTGTA
ATGCACGATTACTTAACCGGGGGATTCACTGCAAATACTAGCTTGGCTCA
TTATTGCCGAGATAATGGTCTACTTCTTCACATCCACCGTGCAATGCATG
CGGTTATTGATAGACAGAAAAATCATGGTATGCACTTTCGTGTACTAGCT
AAAGCATTACGTATGTCTGGTGGAGATCATATTCACGCTGGTACTGTAGT
AGGTAAACTTGAAGGGGAAAGAGACATCACTTTAGGCTTTGTTGATTTAC
TACGTGATGATTTTGTTGAAAAAGATCGAAGCCGTGGTATTTATTTCACT

CAAGATTGGGTCTCTCTACCAGGCGTTCTGCCTGTGGCTTCAGGGGGTAT
TCATGTTTGGCATATGCCTGCTCTGACCGAGATCTTTGGAGATGATTCCG
TACTACAGTTCGGCGGAGGAACTTTAGGACACCCTTGGGGGAATGCACCC
GGTGCCGTAGCTAATCGAGTAGCTCTAGAAGCATGTGTACAAGCTCGTAA
TGAGGGACGTGATCTTGCTCGTGAAGGTAATGAAATTATCCGTGAGGCTA
GTAAATGGAATCCTGAACTAGCTGCTGCTTGTGAAGTATGGAAGGAGATC
AAATTTGAATTCCAACCAATGGATACTTTGTAA-----------------
------TTAATTGGATGCGCTTGGTATGGAAACCTACCGAGTGATAACTT
TCAAATTCAGAGAAACCCGGGAATTACAAACGGGCAATCCTGAGCCAAAT
CCTGTTTTCTGAAACCAAACAAGGATTCA-------GAA---A---GTGA
TAATAAAACGGGATAGGTGCAGAGACTCAACGGAAGCTGTTCTAACAAAT
GG----AGTTGGCTGCGGTGCGTT------AGTAAAGGAAT-CCTTCCAT
CGAAACTCCAGAAAGGATGAAGAATAAACCTATATCTACGTATACGTACT
GAAATACTATCTCCAAACCAAATGATTAATGACGACCCGAATCTTTTTTT
TATTTATATGT------TTATATGAAACAGGAAAG---------------
-----------AATTGTTG-----TGAATCGATTCCA----AGTAAAAGA
AAATGGAATATTCATTGATCAAATCATTTACTCCATCGTAATCTGAT---
-AGATCTTTTGAACAATTGAC------TAATCGGACGAGAATAAAGATAG
AGTCCCATTCTACATGTCAA------------TATTGGCAACAATGAAAT
TTATAGTAAGAGGAAAATCCGTCGACTTTA-GAAATCGTGAGGGTTCAAG
TCCCTCTATCCCCAACCCAAA-GGGCCCGTTTGATTCCCTAATTTTT---
--TATCCTA-TACTCTCATTTCGTTAGCGGTTCAAAATCCGTTATGTTTA
TCATTC------ATTAAGTTTTTT--------------------------
------TCG--TTTCACAAGCCTTGT----GATATATATGATACCCG---
----TAC--------AAATGGAACTCA--TTGAGCAAGT--AACCCCGAT
TGTAAATTGA-------AATGATC-ATATT--------ATCCCTCGTACT
GTACTGAAA----CTCACAAAGTTTTTTTTTTTTTTTTTGCAGATCTACG
AAATT----CCACCAAGGCCTGGATAAGACTTTGTAAT----CCCTTTTT
CGTCTTTTTAATTGACATAGACCCAAGTCATCTATTAA------AATGAG
GATGATGCGTCGTG-----AATGGTCGGGATAGCTCAGCTGGTAGAGCAG
AGATGA--

--------TAGAAACC-T-GCACAGCAGAACGACTTGTGAACATGTGAAA
TTAAATCGGGGATAGCTTCGGGGTCGACTTCGACCTTTA-AGCATCTCTT
GGTCCGA--GGGGTTG---CAATCGGTGCATTGCTCATC-------GGCT
GCG-TGCTTTTTCGGCCAAA-CAAACGAAACTCCAGCGCAAAACGCGCC-
-AAGGAACTTATAACGAATTCGCAT-CGCCTGTCATACCAAAG---ATGG
TATG--------CGTTGGGCTTTGCATCGTGAATGTCTAAAATGACTCTC
GACAACGGATATCTCGGCTCTCGCATCGATGAAGAACGTAGCGAAATGCG
ATACTTGGTGTGAATTGCAGAATCCCGTGAACCATCGAGTCTTTGAACGC
AAGTTGCGCCCGAAGCCT---TAGGCCGAGGGCACGTCTGCTTGGGTGTC
ACACAATGTTGCCCCTCACACCTTGAAGGT---GAAGGGGCGA-------
-------------------------AAGATGGCCTCCCGTGTGGTA---
---G--CGGTTGGCTTAAGTACGAGTCCTCGATGGTTTGCGTTGCAAAAA
TCGGTGGCGATCTAAAATCCCCAGCGTGCTATTTTGCTGCATGAATTGCC
GACGTGGCTTGCTAAACC----------CCTATGCCGCACGTGCGGCCTT
TCCAA---

>Gouania_lupuloides

--TCTTCT
TTGCATTTATTACGACTTTTTCTTCACGAGTATTATAATTGGAATAGTTT
TATTACT---------------------CC------CCCAAAATCTATTT
CTTTTTTTTTGAAAAGTAATTCAAGATTTTTCTTGTTCCTATATAATTCT
CATGTTTGTGAATATGAATCCATCTTACT----TTTTCTCCGTAACCAAT
CTTCTCATTTACGATTAACATCTTCTGGGGTTTTTTTTGAGCGAATTTTT
TTCTATGGAAAAATAAAACATCCTGTAGAAGAAGTCTTTTCTAACGATTT
TCCGGTGGTCTTATGGTTCTTCACGGAGCCTTTCATGCATTATGTTCGAT
ATCAAGGAAAATCTCTTTTGGTTTCAAAAGATACGCCTGTTCTAATGAAT
AAATGGAAATATTATCTTGTCCTTTTATGGCAATGTTTTTTTTATGTGTG
GGCTCAACCCGGAAGGATCTATATAAACCAACTAGCCAACCATACCTTCG
GCTTTTTGGGCTATCTTTCAAGTGTGCGACTCAATCTTTCAGTGGTACGG
AGTCAAATGCCCGAAAATTCATTTATAATGGATAATGCTATAAAGAAGCT
TGATACATTAGTTCCAATTAGTCCAATGATTGGGTCGTTGGCTAAAATGA
AATTTTGTAACGCATTAGGACATCCTGTTAGTAAGTTGGCCTGGGCCGAT
TCGGCAGATTTTGATATTATCGGACGATTTGCCCATATATGCAGAAATCT
TTCTCATTATTCCAGCGGATCCTCAAAAAAAAAGAGTTTGTATCGAATAA

--TACT
GTTAAGTATAGTTATGATTTTGTCAGCCCATATATTTATTCAACAAATAA
ATAACAGTTCTATCTATTTATCTGTATGGTCTTGGACCATCAATAATGAT
TTTTCTTTAGAGTTTGGTTACTTAATTGATCCGCTTACTTCTCTTATGTT
AATATTAATCACTACTGTTGGAATTCTGGTTCTTATTTATAGCGATAATT
ATATGTCTCATGATCGGGGATATTTGAGATTTTTTGCTTATATGAGTTTT
TCTAATACTTCAATGTTAGGATTAGTTACTAGTTCAAATTTGATACAAAT
TTATTTTTTTTGGGAATTGGTTGGAATGTGCTCTTATCTATTAATAGGTT
TTTGGTTCACCCGACCCTTTGCGGCGAATGCTTGTCAAAAGGCGTTTGTA
ACTAATCGGGTAGGGGATTTGGGGTTATTATTAGGAATTTTAGGTTTTTA
TTGGATAACGGGGAGTTTAGAATTTAGGGATTTGTTTGAAATATTCAATA
ATGTGGTTCATAATAATGAAGTTGATTTTTTATTTGTTACTTTATGTGCC
TGTCTATTATTTGCCGGTGCCGTTGCGAAATCTGCCCAATTTCCCCTTCA
TGTATGGTTACCCGATGCTATGGAAGGACCTACCCCAATTTCGGCTCTTA
TACACGCCGCTACTATGGTAGCAGCCGGAATTTTTCTTGTAGCTCGGCTT
CTTCCGCTTTTCATAGTTATACCTTTCATAATGAATCTAATAGCTTTTAT
TGGGATAATAACATTACTTTTAGGAGCCACTTTAGCTCTTGCTCAAAAAG
ATATTAAGAGGGGTTTAGCTTATTCTACAATGTCTCANNNNNNNNNNNNN
NNNNNNNNNNNNNNNNNNNNGTCTTATCGAGCTGCTTTATTTCATTTGAT
TACTCATGCCTATTCGAAAGCATTGTTGTTTTTAGGATCCGGATCAATTA
TCCATTCCATGGAAAGAATTGTTGGATATTCTCCAGATAAAAGCCAGAAT
ATGGTTCTTATGGGCGGTTTAAGAAAACATGTACCAATTACAAAAACTGC
GTTTTTATTAGGTACACTTTCTCTGTGTGGTATTCCGCCGCTTGCCTGTT
TTTGGTCCAAAGATGAAATTCTTAGTGATAGTTGGTTATATTCACCCATT
TTTGCAATAATCGCTTGTTCCACAGCCGGATTAACTGCATTTTATATGTT
TCGGATCTATTTACTTACTTTTGAAGGACATTTAAATGTTCAGTTTCAAA
TTTACAGTGGAAAAAAAAAATAGCTCGTTCTATTCAATATCTCTATGGGGT
AAAGAAGGGCAAAAAGTTTTTAAACCAAAATTTCATTTATTAACGTTATT
AACAATGAATAATAATGAAAGAACTTCTTTTTTTTCTAAAAAGGACTATC
GAATTAATAGTAATCTAAGAAGCACG---------ATGCGCCCTTTTATT
ACTATTAGTCGTTTTGAAACTAAGAACACTTTCTCATATCCGCAGGAATC
GGACAATACTATGCTATTTCCTATGCTTGTATTAGTGCTATTTACTTTAT
TCATTGGATTCATAGGAATTCCTTTCGTCAATCAATTCAATCAAGAAGCT
GTAGATTTGGATATATTATCCATATTGTTAAACCCAGCTATAAACCTTTT
ACACCCAAATCCAAATACTTCTGTGGATTTGTATGAATTTTTTTCAAACG

CAACTTTTTCAGTTAGTATAGCTTTTTTTGGAATCCTTATAGCGTCTTTT
TTATATAAGCCAGTTTATTCCTCTTTACAAAATTTGAATTTCTTTAATTC
ATTTCTTA---------AAAGTATTCCTAAAAAACGAAAAATTCTTTTGG
AAAAACTACTAAATGGGGTATATGATTGGTCATATAATCGTGGTTACATA
GATGGTTTTTATGCAATATCCTTAACTAAAGGTATAAGAGGTTTGGCTCA
ACTC--

-ATAACTTCCCTCTAGATCTAGCTGCTGTNGAAGTTCCGTCTACAAACGG
ATAAGACTTCA--------GTATTAGTGTATACTGAGTTATTGAACATAA
AGGAGCAATAATCCATTTCTTGTTCTAGCAAGAAGTTTGGTATTGCTCCT
TTT-TTATTTATTTAGGAGTTTTTTTAC-----TTAA------ATTTTTT
TTCTTTACTTCATCTTTACTTCAACATACCCATACTTTTTTGTTTTGGTA
TGGAGAAATTTTTTTTTGATATCCTCTTCTCAATTTTTTTATTTTACATT
TAAGGGCTT--------------CAATTGCATTTTTTTTCTTGTAGTTAC
TCTTAGAGAAGAATGAAAAAACAAAATGATAAATGGT----GTAAATTAA
CCTATTTGTTT--------------------------------GTAATAG
GGGCGGATGTAGCCAAGTGGATCAAGGCAGT-------------------

--------------------------------AGTGTTGGATTCAAAGCC
GGTGTTAAAGATTATAAATTGACTTATTACACTCCTCAATATGAAACCAA
AGATACTGATATCTTGGCAGCGTTTCGAGTAACTCCTCAACCTGGAGTTC
CGCCTGAGGAAGCAGGGGCGGCGGTAGCTGCTGAATCTTCTACTGGTACA
TGGACAACTGTATGGACTGACGGGCTTACCAGTCTTGATCGTTACAAAGG
TCGATGCTACGGGCTTGAGCCCGTTGCTGGAGAAGAAAATCAATATATTG
CTTATGTAGCTTACCCCCTAGACCTTTTTGAAGAAGGTTCTGTTACTAAC
ATGTTTACTTCCATTGTGGGTAACGTTTTTGGGTTCAAGGCCCTGCGCGC
TCTACGTCTGGAGGATTTGCGAGTCCCTACTTCGTATATTAAAACTTTCC
AAGGACCACCTCATGGCATCCAAGTTGAAAGAGATAAATTGAACAAGTAT
GGCCGCCCACTATTGGGATGTACTATTAAACCTAAATTGGGGTTATCTGC
TAAGAATTACGGTAGAGCTGTTTATGAATGTCTT----------------

---GGTTCAAAG
TCCCTCTATCCCCAACCAAAA-AGGTCCGTTTGATTCCCTAATTTT----
--TTATCCTATACTCTCATTTCGTTAGCGGTTCAAAATCCGTT-----AT
TCATTCATTAATTTTTCACAAGCC--------------------------
----------------------TTG-----TATATATATGATACACGTAC
---------------AAATGAACATCATTGAGCAAGT----AACCCCGAT
TGGAAATTGAAATTATTGTAAATTGGAATAATCGTATTATCACTCGTACT
GTACCGAAACTCACAAAGTCTTCTTGTTCTTTTTTTTTTGACGATCTAAG
AAATT----CCACCAAGGCCTGGATAAGACTTTGTAAT----CCCTTTTT
CTTCTTTTTAATTGACATAGACCCACGTCCTCTATTAA------AATGAG
GATGGTGCGTCGTG-----AATGGTCGGGATAGCTCAGCTGGTAGAGCAG
AGG---
----------------------------------ACTGAACCTTATCATT
TAGAGGAAGGAGAAGTCGTAACAAGGTTTCCGTAGGTGAACCTGCGGAAG
GATCATTGTCGAAACC-T-GCACAGCAGAGTCACCCGTGAACCAGTGAAA
GCACATGGGAGAGCCTGGGTGGCTTG--------CCCTT-GG-TCTCTCC
TGGTCTA--GGGGCTG---CATTCGGCGCATTTCCCGTT-------GGAT
GTT-TGCTTCCTCGGACGCACAAACTAAAACTCCGGCGCAGGATGCGCC-
-AAGGACCTCATAATGGAATTGCAT-CTCCCAATCGCTCTAGGGATGGAG
CGTG--------AGTGGGGGTTGCATTGTTAGATGTCTAAAACGACTCTC
GGCAACGGATATCTTGGCTCTCGCATCGATGAAGAACGTAGCGAAATGCG
ATACTTGGTGTGAATTGCAGAATCCCGTGAACCATCGAGTYTTTGAACGC
AAGTTGCGCCCGAAGCCA--TTAGGCCGAGGGCACGTCTGCCTGGGCGTC
ACACAATGTTTGCTCCCCCACGACCTCGGTCTCGGAGGGGCGG-------
--------------------------ATGATGGTCTCCCGTGGGGTA---
---G--CGGTTGGCCCAAATGAGAGAGTCCATGGCTGTTCGTGCCGACAC
AATCGGTGGTGTTGAAAAACCCAGCGTCCCATGTTGTTTCACGAACTGTC
CGTGTGGTCCGACAGACC----------C-GATGCTGCAATTGCGGCCTT
TCCACTGCGACCCCAGGTCAGGCGGGGCCACCCGCTGAGTTTAAGC----

>Colubrina_asiatica

-----ATTTAAACTATGTAGCAGATGTACGAATACCCTACCCCATTCATC
TGGAAATCTTGATTCAAACCCTTCGCTATTGGGTGAAAGATGCCTTTTCT
TTGCATTTATTACGTCTTTTTCTTCACGAGTATTATAATTGGGATAGTTT
TATTACT---------------------CC------AAAAAAATCTATTT
CTTTTTTTTTGAAAAGTAATTCAAGATTTTTCTTGTTCCTATATAATTTT
CATGTTTGTGAATACGAATCCATTTTACT----TTTTTTTTGTAACCAAT
TTTTTCGTTTACGATTAACATTTTTTGGTTTTTTTTTTGAGCGAATTTTT
TTCTATGGAAAAATAAACCATCCTGTAGAAGAAGTCTTTTCTAATGATTT
TCCGGCGATCTTATGGTTCTTCACAGAGCCTTTCATGCATTATGTTCGAT
ATCAAGGAAAATCTATTTGGGTTTCAAAAGATACGCCTATTCTAATGAAT
AAATGGAAATATTATCTTGTCCTTTTATGGCAACGTCATTTTTATGTGTG
GGCTCAACCAGGAAGGATCTATATAAATCAATTAGCCAACCATTCCTTCG
GTTTTTTGGGCTATCTTTCAAGTGTGCGACTAAATCTTTCAGTGGTACGG
AGTCAAATGCTAGAAAATTCGTTTATAATGGATAATGCTATAAAGAAGCT
TGATACATTAGTTCCAATTAGTCCAATGATTGGATCATTGGCTAAAATGA
AATTTTGTACCGCATTAGGATATCCTGTTAGTAAGTCGACCTGGACCGAT
TCGTCAGATTTTGATATTATCGACCGATTTGCCCGTATATGCAGAAATCT
TTCTCATTAT--

-------------------------GGAAGTTATGCATGAACGTAATGCT
CATAACTTCCCTCTAGATCTAGCTGCGGTGGAAGTTCCGTCTACAAACGG
ATAATACTTCG--------GTATTAGTGTATACTGAGTTATTGAACATAA
AGGAGCAATAATCAATTTCTTGTTCTAGCAAGAAGTTTGGTATTGCTCCT
TTT-TTATTTAGGAGTTTTTTTACTTAA-----ATAT-AAACAAGTTTTG
TTCTTTACTTCATCTTTACTTCAACATACCCGCACTTTCGTTATGTAGAA
ATT-----TTTCTTTTCTATATCCTCTTCTCACTTTTTTTTATTTCACAT
TTAATTTAA--------------CTGTTTCAATTTTATTTATTATTTTTT
TCTATGAGAAACTACAAGAAAAAAAATGATAAATGGT----GTAAATTGA
CCTATTTGTTTGGA----------TTTT---------------GTAATAG
GGGCGGATGTAG--------------------------------------

-------------------------TAAANCAAGTGTTGGATNCAANGCC
GGTGTTAAAGATTATNAATTGACTTATTACACTCCTGACTATGNAACCAA
AGATACTGATATCTTGGCAGCGTTTCGAGTAACTCCTCAACCTGGAGTTC
CGCCTGAGGAAGCAGGGGCCGCGGTAGCTGCTGAATCTTCTACTGGTACA
TGGACAACTGTATGGACTGACGGGCTTACCAGTCTTGATCGTTACAAAGG
TCGATGCTACCACCTTGAGCCCGTTGCTGGAGAAGAAAATCAATATATTG
CTTATGTAGCTTACCCCTTAGACCTTTTTGAAGAAGGTTCTGTTACTAAC
ATGTTTACTTCCATTGTGGGTAATGTTTTTGGGTTCAAAGCCCTGCGCGC
TCTACGTCTGGAGGATTTGCGAATCCCTCCTGCTTATACTAAAACTTTCC
AAGGACCGCCTCACGGCATCCAAGTTGAAAGAGATAAATTGAACAAGTAT
GGCCGCCCCCTATTGGGATGTACTATTAAACCTAAATTGGGGTTATCCGC
TAAGAATTACGGTAGAGCTGTTTATGAATGTCTTCGCGGTGGACTTGATT
TTACCAAAGATGATGAGAACGTGAATTCCCAACCATTTATGCGTTGGAGA
GACCGTTTCTTATTTTGTGCCGAAGCCCTTTATAAAGCACAGACTGAAAC
AGGTGAAATCAAAGGTCATTACTTGAATGCTACTGCAGGTACATGTGAAG
AAATGATTAAAAGGGCTGTATTTGCCAGAGAATTGGGAGTTCCTATTGTA
ATGCATGATTACTTAACAGGGGGATTCACTGCAAATACTAGCTTGGCTCA
TTATTGCCGAGATAATGGTCTACTTCTTCACATCCACCGTGCAATGCATG

CAGTTATTGATAGACAGAAAAATCATGGTATGCACTTTCGTGTACTAGCT
AAAGCGTTACGTCTGTCTGGCGGAGATCATATTCACGCTGGTACTGTAGT
AGGTAAACTTGAAGGGGAAAGAGACATCACATTAGGCTTTGTTGATTTAC
TACGTGATGATTTTATTGAAAAAGATCGAAGCCGTGGTATTTATTTCACT
CAAGATTGGGTCTCTATACCAGGTGTTTTGCCTGTGGCTTCAGGGGGCAT
TCATGTTTGGCATATGCCTGCTCTGACCGAGATCTTTGGAGATGATGCCG
TACTACAGTTTGGCGGAGGAACTTTAGGACACCCTTGGGGAAATGCACCC
GGTGCCGTAGCTAATCGAGTAGCTCTAGAAGCGTGTGTACAAGCTCGTAA
TGAGGGACGTGATCTTGCTCGTGAGGGTAATGAAATTATCCGTGAGGCTA
GTAAATGGAGTCCTGAACTAGCTGCTGCTTGTGAAGTATGGAAGGAGATC
AAATTTGAATTCCAAGCAATGGATACTTTGTAA-----------------
-----------ATGGAGGAGCTTGGTATGGAAACTACTGAGTGATAACTT
TCAAATTCAGAGAAACCCTGGAATTACAAATGGGCAATCCTGAGCCAAAT
CCTGTTTTCTGAAAACAAACAAGGATTCA-------GAA---A---GTGA
TAATAAAAAGGGATAGGTGCAGAGACTCAATGGAAGCTGTTCTAACAAAT
GG----AGTTGGCTGCGATGTGTT------AGTAACGGAAT-TCTTCCGT
CGAAACTACAGAAAGGATGAAGAATAAACGTATATATAC------GTACT
GAAATCCTATCTCCAAACCAAATGATTAATGACGACTCGAATCTTTTTTT
TATTTCTATGT------TTATATGAAAAATGAAAG---------------
-------AATTGTTGTGAA-----TCGATTC-CAAGTCAAAAAAAAAAAA
AAATGGAATATTCATTGGTCAAATCATTTACTCCATCGTAATCTGAT---
-AGATCTTTTGAAAAATTGAT------TACTCGGACGAGAATAAAGATAG
AGTCCCATTCTACATGTCAA------------TATCGACAACAATGAAAT
TTATAGTAAGAGGAAAATCCGTCGACTTTA-GAAATCGTGAGGGTTCAAG
TCCCTCTATCCCCAAAAAGGC-CCATTTGATTCCCTAATTTTTTATG---
--CTATACTCTCATTTCATTTCGTTAGCGGGTCAAAATTCGTTATGTTTC
TCGTTTCTCATTCATTAATTTTTT-------------------------
------TCT--TTTCACAAGCCTTGT----GATATATATGATACACG---
----TAC--------AAATGAACATCA--TTGAGCAAGT--AACCCCGAT
TGTATATTGG-------AATGATC-ATATT--------ATCGCTCGTACT
GTACTGAAA----CTTGCAAAATCTTCT-------TTTTGAAGATCTAAG
AAATT----TTAACAAGGTCTGGCTAAGACTTTGTAAT----CCCCTTTT
CGCCTTTTTAATTGACATAGACCCAAGTCATCTATTAA------AATGAG
GATGATGCAGCGTG-----AATGGTCGGGATAGCTCAGCTGGTAGAGGAG
AGATGAA---
GGCCGTTCGCTGCCCGCGACGTCGCGAGAAGTCCACTGAACCTTATCATT
TAGAGGAAGGAGAAGTCGTAACAAGGTTTCCGTAGGTGAACCTGCGGAAG
GATCATTGTCGAAA-CCT-GCCCACAGGAACGACCCGCGAACAAGTGGAA
ACATCACATACGGGGGCTGCGG--GGCCTTTTTG--GCT-CCGGGCTCTC
TCGGCCGAGGGCTGCA---CCCTGCG------------------------
------G----TCCTCAGG-CCTAACAAAACCCCGGCGCGAAATGCGCCA
AGGAACATCGTAAACGAACCGGCAG--CACCCCCGGCCCCAGAAATGGAG
CGC----CGTTCGTGGTGCGCGTCGTATTCTATATGTCAAAACGACTCTC
GGCAACGGATATCTCGGCTCTCGCATCGATGAAGAACGTAGCGAAATGCG
ATACTTGGTGTGAATTGCAGAATCCCGTGAACCATCGAGTCTTTGAACGC
AAGTTGCGCCCGAAGCCA--TATGGCCGAGGGCACGTCTGCCTGGGTGTC
ACACAACGTTGCCCCCCCCAAACTTCTACCACGAAGG---CA--------
-----------AGGGGGGGGGGGCGGAGGATGGCCTCCTGTGCGCGGTGG
CGCG--CGGCTGGCCGAAAATGCAAAGCCTCCGGCGAAGAGCGCCGCAAC
AATCGGTGGTTGTCCAACCCTCGGTGCCCGTTGCG--CACGCTGATAGCC
GCTGTGGCCCGATAGACC---CCAACGCGCTGCTGCAAAAAATGCGGGTC
TCCAACGCGACCCCAGTCAGGCGGGTACCCG-------------------

>Pomaderris_angustifolia

------------------------CACGAGTATTATAATTGGAATAGTTT
TATTACT---------------------CCAAAAAAAAAAAAATCTATTT
CTTTTTTTTTGAAAAGTAATTCAAGATTTTTCTTGTTCCTATATAATTCT
CATGTTTGTGAATACGAATACATCCTACT----TTTTCTCCGTAACCAAT
CTTCTCATTTACGATTAACATCTTCTGGGGTTTTTTTTGAGCGAATTTTT
TTCTATGGAAAAATAAAACATCCCGTAGAAGAAGTCTTTTCTAATGATTT
TCCGGCAATCTTATGGTTCTTCACGGAGCCTTTCATGCATTATGTTAGAT
ATCAAGGAAAATCTTTTTTGGTTTCAAAAGATACGCCTCTTCTAATGAAT
AAGTGGAAATATTATCTTGTCCTTTTATGGCAATGTCATTTTTATGTGTG
GGCTCAACCAGGAAGGATCTATATAAACCAATTAGCCAACCATTCCTTCG
GCTTTTTGGGCTATCTTTCAAGTGTGCGACTAAATCTTTCAGTTGTACGG
AGTCAAATGCTAGAAAATTCGTTTATAATGGATAATGCTATAAAGAAGCT
TGATACATTAGTTCCAATCAGTCCAATGATTGGGTCATTGGCTAAAATGA
AATTTTGTAACGCATTAGGACATCCTGTTAGTAAGTCGACCTGGGCCGAT
TCGGCAGATTTTGATATTATCGACCGATTTACCCGTATATGCAGAAATCT
TTCTCATTATCCAGCGGATCCT-CAAAAAAAAAAGAGTG-----------

----GACTTCG--------GTATTGGTGTATACTGAGTTATTGAACATAA
AGGAGCAATAATAAATTTCTTGTTCTACCAAGAAGTTTGGTATTGCTCCT
TTT-TTATTTAGGATTTTTTTTGACTTAAATAAGTCTGTTTCTTTACTTC
ATCGTTACTTCATCTTTACTTCAACATACCCTT-----CGTTATGTAGAA
ATTTTTATTTTCGATATCCTCTTCTCAATTTTTTTTCTTATAGTTTTTCT
TATAAGA--------------------------------------ATTAA
AATAAGAATTAAAATAAAAAATTTAAATATAAATGGT----GTAAATTGA
CCTATT--

---C
GATCCTTCCGTCCCAGAGCACCCCCAACCCATTATAGCATTATAATATAT
ATAATGAATGCTATATATCAGAAAAAAGATTGCC-------TTTTAAAAC
A------CCCTTCTGTTTAAGAGTATAATATTAAGAGTATAATATTGGAT
TGGAAAAGTTTTATTAGGATTCTTAATAATTCTTCTCTGAATCATATCTT
TTTCACCATCTGAATCATATCTTTTTCACCAATTGAATCGTGTTCAAATA
ACACGCAGATGTAATTGAATCTATTTGTGATCCCTAAATATTAAA-----
-TATTTAA-ATAGAATATCTATAATTCCTTTCAGTAACAATTTTTTTCAG
TAAAAGTTTTTTAGTCTATCTGTATGGGGGTCCCATATTATTGTTATTTC
GATTC--------CTATTTTAGTAGCCAGTATGAAAAAACAACAACCTTA
CCTTACACCAGTCTTTATCCACTATTTCATATTTCACTAAAAAAATAAAT
TGGATTTTTTAGCTATCTAGTTTTTTAATCATTGATGTTTTAATACAAAT
ATGGATTTATTCTAGGATGCTAGAATGTGGTCCTTACTTTAGAATGTTAT
TCAAATAATGATCTCGAAGCCGGAAATTTTTA----AATCACTTAAATCT
TTTTGATGATTTCTTATGAGTTCGTAGTACTCGAAGAAGTGTGAAATTGG
TAAATTTGTCCTATCACTATCAAGTTACTTGAAGATGCAGATTCAAAAAG
AAATTTATTTATTTATTTAATATTATATTAAATAAATAAAT---------

-----------------------AAAAAAATGAAATAAAATATATGTACT
GGGTATTGGGGATTCAATTTAATTCGTTCTGAGACTTAGTCAGAACCTAG
CCATTCATATTTCATATAGAAAGA-------------------AAAAGTA
TAGATTACTATGTACCGACCGAACCAATGACTATTCATGATTCCATAATT
GAATCAATTACATACTGGTCCAAA--------------------------
-------------------------------TTCTGTTGGACTCAAAGCC
GGTGTTAAAGATTATAAATTGACTTATTACACTCCTGAATATGAAACCAA
AGATACTGATATCTTGGCAGCGTTTCGAGTAACTCCTCAACCTGGAGTTC
CGCCTGAGGAAGCGGGGGCCGCGGTAGCTGCTGAATCTTCTACTGGTACA
TGGACAACTGTATGGACTGACGGGCTTACCAGTCTTGATCGTTACAAAGG
TCGATGCTACCACATCGAGCCCGTTGCTGGAGAAGAAAGTCAATTTATTG
CTTATGTAGCTTACCCCTTAGACCTTTTTGAAGAAGGTTCTGTTACTAAC
ATGTTTACTTCCATTGTAGGTAATGTTTTTGGGTTCAAGGCCCTGCGCGC
TCTACGTCTGGAGGATTTGCGAATCCCTCCTGCTTATACTAAAACTTTCC
AAGGACCGCCTCACGGCATCCAAGTTGAAAGAGATAAATTGAACAAGTAT
GGCCGCCCCCTATTGGGATGTACTATTAAACCTAAATTGGGGTTATCCGC
TAAGAATTACGGTAGAGCTGTTTATGAATGTCTT----------------

-------TTGGATTCGAGCCTTGGTATGGAAACCTACCAAGTGATAACTT
TCAAATTCAGAGAAACCCTGGAATTACAAATGGGCAATCCTGAGCCAAAT
CCTGTTTTCTGAAAACAAACAAGTATTCA-------GAA---A---GTGA
TAATAAAAAAGGATAGGTGCAGAGACTCAATGGAAGCTGTTCTAACAAAT
GG----AGTTGGCTGCGATGCGTT------AGTAAAGGAAT-CCTTCCAT
CGAAACTCCAGAAAGGATGAAGAATAAACCTATATATACGTATACATACT
GAAATACTATCTCCAAACCAAATGATTAATGACGACCCGAATATTTTTTT
TTTT----TTT------TTATATGTTTATATGAAA--------------A
ATAAAAGAATTGTTGTGAA-----TCGATTCCAAGTAAAAA------AAA
AAATAGAATATTCATTGATCAAATCATTTACTCCATCGTAATCTGAT---
-AAATCTTTTGAAAAATGGAT------TAATCGGACGAGAATAAAGATAG
AGTCCCATTCTACGTGTCAA------------TATCGACAACAATGAAAT
TTATAGTAAGAGGAAAATCCGTCGACTTTA-GAAATCGTGAGGGTTCAAG
TCCCTCTATCCCCAAAAAGGC-CCAGTTGATTCCCTAATTTTTTATC---
--GTATACT-C----TCATTTCGTTATCGGTTCAAAATTCATTATCTTTC
TCATTC------ATTAATT-TTTT--------------------------
------TCT--TTTCACAAGCCTTGT----GGTATATATGATACACA---
----TAC--------AAATGAACATCA--TTGAGCAAGT--AACCCCGAT
TGTAAATTGG-------AATGATC-ATATT--------ATCGCCCGTACT
GTACTGAAA----CTTACAAAGTCTTTTTT----TTTTAA--GATCTAAG
AAATT----CCACCAAGGCCTGGATAACACTTTGTAAT-----CCCTTTT
CGTCTTTTTAATTGACATAGACCCAAGTCATCTATTAA------AATGAG
GATGATGCGTCGTG-----AATGGTCGGGATAGCTCAGCTGGTAGAGCAG
AGGACTGAAAATCCTCGTGTCACCAGTTCAAATAA---------------

---AAG
GATCATTGT-CGAACCCT-GCCTAGCAGAACGACCCGTGAACCCGTAAA-
AAC--ATCTCTGGGGGCTTGG---GGCCTTAGGC--CTC-GGGCTCTCT-
TGGTCGAG-GGCTGCA---TGCTGTG--CTTCGCTTGCCGATGCATAT--
GTACTGCTTCCCCGGCTGC--ACAAACGAACCCCGGCGCAAACCGCGCCA
AGGAT--CATCGAACGAATTAGCAC-TGCCCCGTCACCCCAGAGATGGTG
TGTGGTCGGGGTGTGCGT--CGTATTC--AATTAAGTCAAAACGACTCTC
GGCAACGGATATCTCGGCTCTCGCATCGATGAAGAACGTAGCGAAATGCG
ATACTTGGTGTGAATTGCAGAATCCCGTGAACCATCAAGTCTTTGAACGC
AAGTTGCGCCCGAAGCCA--TTAGGCCGAGGGCACGTCTGCCTGGGCGTC
ACACAATGTTGCCCCCCCAACCTCAATCTTGAA-----------------
-----------GG---CGGGGGGCGGATGCTGGCTTTCCGTGCCTCACGG
TTTG--CGGTTGGCCGAAAT--ACGAGTCCCCGGCGACGAGTGCCGCAGC
AATCGGTGGTTCTCCAACCCTCG-GTGCCCCGCTGCGTGCACAGATCGCT
GCTGTGGACAGATAGACC---CCAA-TACGT----CGCAGCTGCGGGGTC
TACAATGCGACCTCAGGTCAA-----------------------------

>Paliurus_ramosissimus

---CTTCT
TTGCATTTATTACGGCTTTTTCTTCACGAGTATTATAATTGGAATAGTTT
TATTACT---------------------CC------AAAAAAATCTATTT
CTTTTTTTTTGAAAAGTAATTCAAGATTTTTCTTGTTCCTATATAATTCT
CATGTTTATGAATACGAATCCATCTTACT----TTTTCTCCGTAACCGAT
CTTCTCATTTACGATTAACATCTTCTGGGGTATTTTTTGAGCGAATTTAT
TTCTATGGAAAAATAAAACATCCTGTACAAGAAGTCTTTTCTAATGATTT
TCCGGCGGTCTTATGGTTCTTCACGGAGCCTTTCATGCATTATGTTAGAT
ATCAAGGAAAATCAATTTTGGTTTCAAAAGATACGCCTCTTCTAATGAAT
AAATGGAAATATTTTCTTTTCCTTTTATGGCAATGTCGTTTTTATGTGTG
GGCTCAACCAGGAAGGATCTATATAAACCAATTAGCCAACCATTCTTTCG
GCTTTTTAGGCTATCTTTTAAGTGTGCGACTAAATCTTTCAGTGGTACGG
AGTCAAATGCTAGAAAATTCATTTATAATGGATAATGCTATAAAGAAGCT
TGATACATTAGTTCCAATTAGCCCAATGATTGGATCATTGGCTAAAATGA
AATTTTGTAACGCATTAGGACATCCTGTTAGTAAGTCGACCTGGGCCGAT
TCGTCAGATTTTGAGATTATCGACCGATTTGCCCGTATATGCAGAAATCT
TTCTCATTATTTCAGCGGATCC----------------------------

-TTTCTTTAGAATTTGGATACTTAATTGATCCACTTACTTCTCTTATGTT
AATATTAATCACTACTGTTGGAATTATGGTTCTTATTTATAGTGATAATT
ATATGTCTCATGATCAGGGATATTTGAGATTTTTTGCTTATATGAGTTTT
TCTAATACTTCAATGTTAGGATTAGTTACTAGTTCAAATTTGATACAAAT
TTATTTTTTTTGGGAATTAGTTGGAATGTGTTCTTATCTATTAATAGGTT
TTTGGTTCACCCGACCTATTGCGGCAACTGCTTGTCAAAAGGCGTTTGTA
ACTAATCGTGTAGGGGATTTTGGGTTATTATTAGGAATTTTATGTTTTTA
TTGGATAACGGGTAGTTTAGAATTTAGGGATTTGTTTGAAATCTTCAATA
ACGTGGTTGATAATAATGAAGTTGATTTTTTATTTGTTACTTTGTGTGCC
TGTCTATTATTTGCCGGTGCAGTTGCTAAATCTGCCCAATTTCCCCTTCA
TGTATGGTTACCCGATGCTATGGAAGGACCTACCCCTATTTCCGCTCTTA
TACACGCAGCTACTATGGTAGCAGCCGGAATTTTTCTTGTAGCTCGGCTT
CTTCCGCTTTTCATAGTTATACCTTTCATAATGAATCTAATAGCTTTTAT
TGGGATAATAACATTACTTTTAGGAGCCACTTTAGCTCTTGCTCAAAAAG
ATATTAAGAGGGGTTTAGCTTATTCTACAATGTCTCAATTGGGTTATATG
ATGTTGGCTCTAGGTATGGGGTCTTATCGAGCGGCTTTATTTCATTTGAT
TACTCATGCTTATTCGAAAGCATTGTTGTTTTTAGGATCCGGATCAATTA
TTCATTCCATGGAAACAATTGTTGGATATTCTCCAGATAAAAGCCAGAAT
AT--

-----------------------------CGAAGTTCCGTCTACAAACGG
ATAAGACTTTG--------GTATTAGTGTATACTGAGTTATTGAACATAA
AGGAGCAATAATCAATTTCTTGTTTTAGCAAGAAGTTTGGTATTGCTCCT
TTA-GGAGTTTTTTTACTTAAATTTTTT-TCTTTACT-TCATCTTTACTT
CAATTTACTTCATCTTTACTTCAACATACCCATACTTTCGTTATGTAGAA
ATTTTTATTTTCGATATCCGCTTCTCAATTTTTTTTA------------
-------TTTTACATTTAA----AGGTTTCAATTCCATT--TTTTCTTCT
TTTAGTTTGAAGAATGAAAAAAAAAAATGATAAATGGT----GTAAATTGA
CCTATTTGTTTGTA----------ATTTGTAAATTTGTAATTTGTAATAG
GGGCGGATGTAGCCAAGTGGATCAAGGCAG--------------------
---C
GATCCTTCCGTCCCAGAGCATACCCAACCCATTATAGCATTATAATATAT
ATAATGCTATAT----ATCAGAAAAAAGATTGCCTTAA----AATAAAAC
A------CCTTTCTGTTTRAGAGTATAATAATAAGAGTATAATATTGGAT
TAGAAAAATTTTATTAGTATTCTTAATAATTCTTCTCTGAATCATA----
-------------------TCTTTTTCACAAATTGAATCGTGTTCAAATA
ACACGCAGATATAATTGGATCCATTTGTGATCCCTAAATGTTAAA-----
-TATTTAA-ATAAAATATCTATAATTCCTTTCAGTAA-------------
---CACTTTTTTAGTCTATCTGTATAGGGGTCCCATATTATTGTTATTTC
GATTCCAAT--------TTTAGAAATCAGTATGAAAAAACAACAACCTTA
CCTTACACCAGTCTTTATCCACT-------AGTTCACTAAAAAAATAAAT
TGGATTTTTTAGCTATCTAGTTTTTTAATCATTGATGGTTTAATACAAAT
ATGGATTTATCTTAGGATGCTAAAATGTGGTCCTTACTTTAAAATGTTAT
TCAAATAATGATCTCGAGTCCGGAAATTTTTA----AATCACTTAAATCT
TTTTGATGATTTCTTATGAGTTCGTAGTACTCGAAGAAGTGTGAAATTGG
TAAATTTATCCTATCACTATCAAGTTACTTGAAGGTGCAGATTCAAAAAG
AAATTTTTTATTTTATTCGTGGTATAAATATATGTGTTGGGGATTA----

---------------AATTGAATTCGTTCTGAGACTTCGTCAGAACCTAG
CCATTCATATTTCATATAGAAAGA-------------------AAAGGTA
TAGATTACTATGTACCGACTGAACCAATGACTATTCATGATTCCATAATT
GAATCAATTACATACTGGTCC-----------------------------
-----------------------ACTAAAGCAAGTGTTGGATTCAAAGCC
GGTGTTAAAGATTATAAATTGACTTATTACACTCCTGAATATGAAACCAA
AGATACTGATATCTTGGCAGCGTTTCGAGTAACTCCTCAACCTGGAGTTC
CGCCTGAGGAAGCAGGGGCCGCGGTAGCTGCTGAATCTTCTACTGGTACA
TGGACAACTGTATGGACTGATGGGCTTACCAGTCTTGATCGTTACAAAGG
TCGATGCTACGGCCTTGAGCCCGTTGCTGGAGAAGAAAATCAATTTATTG
CTTATGTAGCTTACCCCTTAGACCTTTTTGAAGAAGGTTCTGTTACTAAC
ATGTTTACTTCCATTGTGGGTAATGTTTTTGGGTTCAAGGCCCTGCGCGC
TCTACGTTTGGAGGATTTGCGAATCCCTCCTGCTTATTCTAAAACTTTCC
AAGGACCGCCTCATGGCATCCAGGTTGAAAGAGATAAATTGAACAAGTAT
GGCCGCCCCCTATTGGGATGTACTATTAAACCTAAATTGGGGTTATCTGC
TAAGAATTACGGTAGAGCAGTTTATGAATGTCTTC---------------

------------CAAAAAGGC-CCATTTGATTCCCTAATT-----TT---
--TATCCTA-TACTCTCATTTCGTTAGCGGTTCAAAATTCGTTATGTTTC
TCATTCATTAA---------------------------------------
--TTTTTTTCTTTTCACAAGCCTTGT----GATATATATGATACACG---
----TAC--------AAATGAACATCA--TTGAACAAGT--AACCCCGAT
TGTAAATTGG-------AATGATC-ATATC--------ATCGCTCGTACT
GTACTGAAA----CTTACAAAGTCTTCTTTTT--TTTTTGAAGATCTAAG
AAATT----CCACCAAGGCCTGGATAAGACTTTGTACT----CCCCTTTT
CGYCTTTTTAATTGACATAGACCCAAGTCATCTATTAA------AATGAG
GATGATGCGYCGKG-----AAKGGYCGGRATASCTCASCKGGTARAGCAR
AGGACTGAAAATCC------------------------------------

---AAG
GATCATTGTCGAAA-CCT-GCCCAGCAGAACGACCGGCGAACCCGTAAAA
ACCAACCGGGGGCCCCGGGGCC--TC----GGCC--CCG-GACCCCTCTC
CGGTCGGGGGGGGATG---CGCCCCGCGCCTCGCCCGTCGACGCGCGGTT
GCCGC-CCTTCCCGGCCGC--ACAAACGAACCCCGGCGCAAACCGCGCC-
-AAGGAACACCGAACGAATTGGCATCTGCCCCCCCGCCCCAGAGATGGTG
TGCGGCCGGGGCGCGCGCC-GTATTCTGTATGTATGTCAAAACGACTCTC
GGCAACGGATATCTCGGCTCTCGCATCGATGAAGAACGTAGCGAAATGCG
ATACTTGGTGTGAATTGCAGAATCCCGTGAACCATCGAGTTTTTGAACGC
AAGTTGCGCCCGAAGCCA--TTAGGCCGAGGGCACGTCTGCCTGGGCGTC
ACACAACGTTGCCCCCCCAACCCCG---------TCC---CGG-------
-----------GAGGGAAGGGGGCGGATGCTGGCCTCCCGCGCGCCACGG
TCCG--CGGCTGGCCGAAAC--GCGAGTCCCCGGCGACGAGCGCCGCAGC
GATCGGTGGTTGTCAAGCCC-TCGTTCTCCCGCTGCGCGCGCGGATCGCC
GTCGCGGCCCCACAGAGA---CCCCGACGCGC---CGCCGGTGCGGCGTC
ACCAACGCGACCCCAGGTCAGGCGGGGCCACCCGCTGAGTTTAA------

>Ziziphus_calophylla

--------ATTACGGCTTTTTCTTCACGAGTATTATAATTGGAATAGTTT
TATTACT---------------------CC------AAAAAAATCTATTT
CTTTTTTTTTTAAAAGTAATTCAAGATTTTTCTTGTTCCTATATAATTCT
CATGTTTATGAATACGAATCCATCTTACT----TTTTCTCCGTAACCGAT
CTTCTCATTTACGATTAACATCTTCTGGGGTATTTTTTGAGCGAATTTAT
TTCTATGGAAAAATAAAACATCCTGTACAAGAAGTCTTTTCTAATGATTT
TCCGGCGGTCTTATGGTTCTTCACGGAGCCTTTCATGCATTATGTTAGAT
ATCAAGGAAAATCTATTTTGGTTTCAAAAGATACGCCTCTTCTAATGAAT
AAATGGAAATATTTTCTTGTCCTTTTATGGCAATGTCATTTTTATGTGTG
GGCTCAACCAGGAAGGATCTATATAAACCAATTAGCCAACCATTCTTTCG
GCTTTTTAGGCTATCTTTTAAGTGTGCGACTAAATCTTTCAGTGGTACGG
AGTCAAATGCTAGAAAATTCATTTATAATGGATAATGCTATAAAGAAGCT
TGATACATTAGTTCCAATTAGCCCAATGATTGGATCATTGGCTAAAATGA
AATTTTGTAACGCATTAGGACATCCTGTTAGTAAGTCGACCTGGGCCGAT
TCGTCAGATTTTGAGATTATCGACCGATTTGCCCGTATATGCAGAAATCT
TTCTCATTATTTCAGCGGATCCTCAAAAAAAAA-----------------

----GACTTTG--------GTATTAGTGTATACTGAGTTATTGAACATAA
AGGAGCAATAATCAATTTCTTGTTCTAGCAAGAAGTTTGGTATTGCTCCT
TTA-GGAGTTTTTTTACTTAAATTTTTT-TCTTTACT-TCATTTTTACTT
CA---------------------ACATACCCATACTTTCGTTATGTAGAA
ATTTTTATTTTCGATATCCTCTTCTCCATTTTTTTTTTTTTATTTAACAT
TCCCATTTTTAAAATTTAA----AGGTTTCAATTCCATT--TTTTCTTCT
TTTAGTTGGAAAAATG-AAAAAAAAATGATAAAGGGT----GTAAATGGA
CCTATTGGTTGGTA----------ATTGGTAAGTTGGAAATTGGTAATAG
G---

--------------------------------------ATAATATTGGAT
TGGAAAAATTTTATTAGTATTCTGAATAATTCTTCTCTGAATCATA----
-------------------TCTTTTTCACCAATTGAATCGTGTTCAAATA
ACACGCAGATGTAATTGAATCTATTTGTGATCCCTAAATGTTAAA-----
-TATTTAA-ATAGAATATCTATAATTCCTTTCAGTAA-------------
---CAATTTTTTAGTCTATCTGTATGGGGGTCCCGTATTATTGATTATTG
TTATTTCGATT-CCTATTTTAGCAATCAGTATGAA-----AAAACAACAA
CCTTACACCAGTCTTTATCCACT-------ATTTCACTAAAAAACGAAAT
TTGATTTTTTAGCTATCTAGTTTTTTAATCATTGATGTTTTAATACAAAT
ATGGATTTATCTTAGGATGCTAAAATGGGGTCCTTACTTTAGAATGTTAT
TCAAATAATGATCTCGAGGCCGGAAATTTTTCATTAAATCACTTAAATCT
TTTTGATGATTTCTTTTGAGTTCGGAGTACTCGAAGAAGTGTGAAATTGG
TAAATTTATCCTATCACTATCAAGTTACTTGAAGATGCAGATTCAAAAAT
AAATTTTTTATTTTATTCGTGGTATTTTTATTCATTTTTATTTTATTTTA
ATATTTATTATTCTATTAAATTCAATTTATTATATTAAAATATTAAATAT
ATAAAATATTAAATATAAAATTTAAATAAACAAAGAACGAAATAAAATAT
ATGTATTGGGGATTAAATTGAATTCGTTCTGAGACTTCGTCAGAACTKAG
CCATTCATATTATTCATATTYCATATAG------------AAAGAAAAAG
TA--

--CC
GGTGTTAAAGATTATAAATTGACTTATTACACTCCTGACTATGAAACCAA
AGATACTGATATCTTGGCAGCGTTTCGAGTAACTCCTCAACCTGGAGTTC
CGCCTGAGGAAGCAGGGGCCGCGGTAGCTGCTGAATCTTCTACTGGTACA
TGGACAACTGTATGGACTGACGGGCTTACCAGTCTTGATCGTTACAAAGG
TCGATGCTACGGCCTTGAGCCCGTTGCTGGAGAAGAAAATCAATATATTG
CTTATGTAGCTTACCCCTTAGACCTTTTTGAAGAAGGTTCGGTTACTAAC
ATGTTTACTTCCATTGTGGGTAATGTTTTTGGGTTCAAGGCCCTGCGCGC
TCTACGTTTGGAGGATTTGCGAATCCCTACTGCTTATTCTAAAACTTTCC
AAGGACCGCCTCATGGCATCCAGGTTGAAAGAGATAAATTGAACAAGTAT
GGTCGCCCCCTATTGGGATGTACTATTAAACCTAAATTGGGGTTATCTGC
TAAGAATTACGGTAGAGCAGTTTATGAATGTCTTCGCGGTGGACTTGATT

TTACCAAAGATGATGAGAACGTGAATTCCCAACCATTTATGCGTTGGAGA
GACCGTTTCTTATTTTGTACCGAAGCCCTTTATAAAGCACAGGCTGAAAC
AGGTGAAATCAAAGGGCATTACTTGAATGCTACTGCGGGTACATGCGAAG
AAATGATGAAAAGGGCTATATTTGCCAGAGAATTGGGAGTTCCTATTGTA
ATGCACGATTACTTAACAGGGGGATTCACTGCAAATACTAGCTTGGCTCA
TTATTGCCGAGATAATGGTCTACTTCTTCACATCCACCGTGCAATGCATG
CAGTTATTGATAGACAGAAAAATCATGGTATGCACTTTCGTGTACTAGCT
AAAGCGTTACGTCTGTCTGGTGGAGATCATATTCACGCTGGTACTGTAGT
AGGTAAACTTGAAGGGGAAAGAGACATCACTTTAGGCTTTGTTGATTTAC
TACGTGATGATTTTATTGAAAAAGATCGAAGCCGCGGTATTTATTTCACT
CAAGATTGGGTCTCTCTACCAGGTGTTATACCTGTGGCTTCAGGGGGTAT
TCATGTTTGGCATATGCCCGCTCTGACCGAGATCTTTGGAGATGATGCCG
TACTACAGTTCGGCGGAGGAACTTTAGGACACCCTTGGGGAAATGCGCCC
GGTGCCGTAGCTAATCGAGTAGCTCTAGAAGCATGTGTACAAGCTCGTAA
TGAGGGACGTGATCTTGCTCGTGAGGGTAATGAAATTATCCGTGAGGCTA
GTAAATGGAGTCCTGAACTAGCTGCTGCTTGTGAAGTATGGAAGGAGATC
AAATTTGA--

------------CAAAAAAGC-CCATTTGATTCCCTAATT-----GT---
--TATCCTA-TACTCTCATTTCGTTAGCGGTTCAAAATTCGTTATGTTTC
TCATTCATTAA---------------------------------------
--TTTTTTTCTTTTCACAAACCTTGT----GGTATATATGATACACG---
----TAC--------AAATGAACATCA--TTGAGCAAGT--AACCCCGAT
TGTAAATTGG-------AATGATC-ATATT--------ATCGCTCGTACT
GTACTGAAA----CTTACAAAGTCTTCTTTTT---TTTTGAAGATCTAAG
AAATT----CCACCAAGGCCTGGATAAGACTTTGTACT----CCCCTTTT
CGTCTTTTTAATTGACATAGACCCAAGTCATCTATTAA------AATGAG
GATGATGCGTCGKG-----AATAGTCGGGATAGCTCAGCTGGTAGAGCAR
AGGACTGAAAATCCTCG---------------------------------

---AAG
GATCATTGTCGAAA-CCT-GCCCAGCAGAACGACCGGCGAACCCGTAACA
AACACACCGGGGGGACCCCGGG--GCCTTCGCGC--CCC-GTGCCCCCCT
TCGGTCGGGGGGGCCG---CATCCCGTGCCTCGCCTGTCGATGCGCGTTT
GCC-G-CTCTCTCGGCCGC--TCAAACGAACCCCGGCGCAAACCGCGCC-
-AAGGAACACCTAACGAATTGGCATTCACCCCCTGCCCC-GGAGACGGTG
CGCGGTCGGGGTGTGCGTCGCATTCTGTATTGTATGTCAAAACGACTCTC
GGCAACGGATATCTCGGCTCTCGCATCGATGAAGAACGTAGCGAAATGCG
ATACTTGGTGTGAATTGCAGAATCCCGTGAACCATCGABTTTTTGAACGC
AAGTTGCGCCCGAAGCCA--TTAGGCCGAGGGCACGTCTGCCTGGGCGTC
ACACACAACYTTGCCCCCCACCCCCGACGTCGACCGA---CGG-------
-----------AGGGGAAGGGGGCGGATGCTGGCCTCCCGTGCGCCACGG
TCCG--CGGCTGGCCGAAAC--GCGGGTCCCCGGCGACGAGCGCCGCAGC
AATCGGTGGTTGTCCAAACCCTCGTCTCCCCGCTGCGCGCGCGGATCGCC
GCCGCGGCCCCACAGAGA---CCCCAACGCGC---CGCACCAGCGGCGTC
TCCAACGCGACCCCAGGTCAGGCGGG------------------------

>Hovenia_dulcis

--TCTTCT
TTGCATTTATTACGGCTTTTTCTTCACGAGTATTATAATTGGAATAGTTT
TATTACT---------------------CC------AAAAACATCTATTT
CTTTTTTTTTGAAAAGTTATTCAAGATTTTTCTTGTTCCTATATAATTCT
CATGTTTGTGAATACGAATCCGTCTTACT----TTTTCTCCGTAACCAAT
CTTCTCATTTACGATTAACATCTTCTGGGGTTTTTTTTGAGCGAATTTTT
TTCTATGGAAAAATAAAACATCCTGTAGAAGAAGTCTTTTCTAATGATTT
TCCGGCGGTCTTATGGTTCTTCACGGAGCCTTTCATGCATTATGTTAGAT
ATCAAGGAAAATCTATTTTGGTTTCAAAAGATACGCCTCTTCTAATGAAT
AAATGGAAATATTATCTTGTCCTTTTATGGCAATGTCATTTTTATGTGTG
GGCTCAACCAGAAAGGATCTATATAAACCAATTAGCCAACCATTCCTTCG
GCTTTTTAGGCTATCTTTCAAGTGTGCGACTAAATCTTTCAGTGGTACGG
AGTCAAATGCTAGAAAATTCATTTATAATGGATAATGCTATAAAGAAGCT
TGATACATTAGTTCCAATTAGCCCAATGATTGGATCATTGGCTAAAATGA
AATTTTGTAACGCATTAGGACATCCTGTTAGTAAGTCGAGCTGGGCTGAT
TCGTCGGATTTTGATATTATCGACCGATTTGCCCGTATATGCAGAAATCT
TTCTCATTATTCCAGCGGATCCTCAAAAAAAAAGAGTTTGTATCGAATAA
AATAT---

------------AATCTTCGCCGTATGTGGGTTTTTCCTAGTGTTTTACT
GTTAAGTATAGTTATGATTTTTTCCGCCCATATATTTATTCAACAAATAA
ATAACAGTTCTATCTATCTATCTGTATGGTCTTGGACCATCAATAATGAT
TTTTCTTTAGAATTTGGCTACTTAATTGATCCACTTACTTCTCTTATGTT
AATATTAATCACTACTGTTGGAATTATGGTTCTTATTTATAGTGATAATT
ATATGTCTCATGATCAGGGATATTTGAGATTTTTTGCTTATATGAGTTTT
TCTAATACTTCAATGTTAGGATTAGTTACTAGTTCAAATTTGATACAAAT
TTATTTTTTTTGGGAATTAGTTGGAATGTGTTCTTATCTATTAATAGGTT
TTTGGTTCACCCGACCTATTGCGTCGAATGCTTGTCAAAAGGCGTTTGTA
ACTAATCGTGTAGGGGATTTTGGGTTATTATTAGGAATTTTAGGTTTTTA
TTGGATAACGGGTAGTTTAGAATTTAGGGATTTGTTTGAAATATTCAATA
ACGTGGTTGATAATAATGAAGTTGATTTTTTATTTGTTACTTTGTGTGCC
TGTCTATTATTTGCCGGTGCAGTTGCTAAATCTGCCCAATTTCCCCTTCA
TGTATGGTTACCCGATGCTATGGAAGGACCTACCCCTATTTCGGCTCTTA
TACACGCTGCTACTATGGTAGCAGCCGGAATTTTTCTTGTAGCTCGGCTT
CTTCCGCTTTTCATAGTTATACCTTTCATAATGAATCTAATAGCTTTTAT
TGGGATAATAACATTACTTTTAGGAGCCACTTTAGCTCTTGCTCAAAAAG
ATATTAAGAGGGGTTTAGCTTATTCTACAATGTCTCAATTGGGTTATATG
ATGTTGGCTCTAGGTATGGGGTCTTATCGAGCGGCTTTATTTCATTTGAT
TACTCATGCTTATTCGAAAGCATTGTTATTTTTAGGATCCGGATCAATTA
TTCATTCCATGGAAACAATTGTTGGATATTCTCCAGACAAAAGCCAGAAC
ATGGTTCTTATGGGCGGTTTAAGAAAACATGTACCAATTACAAAAACTGC
GTTTTTATTAGGTACACTTTCTCTGTGTGGTATTCCACCCCTTGCTTGTT
TTTGGTCTAAAGATGAAATTCTTAGTGATAGTTGGTTATATTCACCAAT-

--GTAATGCT
CATAACTTCCCTCTAGATCTAGCTGCTGTCGAAGTTCCGTCTACAAACGG
ATAAGACTTTG--------GTATTAGTGTATACTGAGTTATTGAACATAA
AGGAGCAATAATCAATTTCTTGTTCTAGCAAGAAGTTTGGTATTGCTCCT
TTT-TTATTTATTTATTTAGTATTTAGG-----AGTT-TTTTT-----TT
TTACTTAATTCAATTGTGTCTTCTTCT------AGTTTCTCTTAGAAGAC
CGAAAA--

---------------------AAAAAGGATAAATGGT----GTAAATTGA
CCTATTTTTTTGTA----------ATTT---------------GGAATAG
GGGCGGA---

-----------CCCAGAGCATACCCAACCCATTATAGCATTATAATATAT
ATAATGCTATAT----ATCAGAAAAAAGATTGCCTTTTAAAACATAAAAC
A------CCTTTCTGTTTAAGAATATAATATTAAGAGTATAATATTGGAT
TGGAAAAAGTTTATTAGTATTCTTACTAATTCTTCTCTGAATCATA----
-------------------TCTTTTTCACAAATTGAATCGTGTTCAAACA
ACACGCAGATATAATTGGATCCATTTGTGATCCCTAAATGTTAAA-----
-TATTTAACATAGAATATCTATAACTCCTTTCAGTAA-------------
---CAATTTTTTAGTCTATCTGTATGGGGGTACCATATTATTGTTATTTC
GATTCCTAT-------ATTTAGCAATCAGGATGAAAAAACAACAACCTTA
CCTTACACCAGTCTTTATCCACT-------AGTTCACTAAAAAAAGAAAT
TGGATTTTTTAGCTATCTAGTTTTTTAATCATTGATGGTTTAATACAAAT
ATAGATTTATCTTAGGATGCTAAAATGTGGTCCTTACTTTAAAATGTTAT
TCAAATAATGATCTCGAGTCCGTAAATTTTTC----AATCACTTAAATCT
TTTTGATGATTTCTTATGAGTTCCTAGTACTCGAAGAAGTGTGAAATTGG
TAAATTTATCCTATCACTATCAAGTTACTTGAAGATGCGGATTCAAAAAG
AAACTTTTTATTTTATTCGTGGTATTTATATTTTATTTATATTTAATGTT
TATTATTAATGTTTATTATATATTAAATNATA----------AATAAAATAT
ATGTTAAAATATAT--------------------ATAAAATAAAATATAT
GTATTGTGTATTGGGGATTAAATTCGTTCTGAGACTTTGTCAGAACCTAG
CCATTCATATTTCATATAGAAAGAAAAA------------GTAAAAAGTA
TAGATTACTATGTACCGACCGAACCAATGACTATTCATGATTCCATAATT
GAATCAATTACATACTGGTTCC----------------------------
-----------------------------------GTGGGATTCAAAGCC
GGTGTTAAAGATTATAAATTGACTTATTACACTCCTGACTATGAAACCAA
AGATACTGATATCTTGGCAGCGTTTCGAGTAACTCCTCAACCTGGAGTTC
CGCCTGAGGAAGCAGGGGCCGCGGTAGCTGCTGAATCTTCTACTGGTACA
TGGACAACTGTATGGACTGACGGGCTTACCAGTCTTGATCGTTACAAAGG
TCGATGCTACCACATTGAGCCCGTTGCTGGAGAAGAAAGTCAATTTATTG
CGTATGTAGCTTACCCCTTAGACCTTTTTGAAGAAGGTTCTGTTACTAAC
ATGTTTACTTCCATTGTGGGTAATGTTTTTGGGTTCAAGGCCCTGCGCGC
TCTACGTCTGGAGGATTTGCGAGTCCCTCCTGCTTATTCTAAAACTTTCC
AAGGACCGCCTCATGGCATCCAGGTTGAAAGAGATAAATTGAACAAGTAC

GGCCGCCCCCTATTGGGATGTACTATTAAACCTAAATTGGGGTTATCTGC
TAAGAATTATGGTAGAGCAGTTTATGAATGTCTTCGCGGTGGACTTGATT
TTACCAAAGATGATGAGAACGTGAATTCCCAACCATTTATGCGTTGGAGA
GACCGTTTCTTATTTTGTGCCGAAGCCCTTTATAAAGCACAGGCTGAAAC
AGGCGAAATCAAAGGGCATTACTTGAATGCTACTGCAGGTACATGCGAAG
AAATGATTAAAAGGGCTGTATTTGCCAGAGAATTGGGAGTTCCTATTGTA
ATGCACGATTACTTAACAGGGGGATTCACTGCAAATACTAGCTTGGCTCA
TTATTGCCGAGATAATGGTCTACTTCTTCACATCCACCGTGCAATGCATG
CAGTTATTGATAGACAGAAAAATCATGGTATGCACTTTCGTGTACTAGCT
AAAGCGTTACGTCTGTCTGGTGGAGATCATATTCACGCTGGTACTGTAGT
AGGTAAACTTGAAGGGGAAAGAGACATCACTTTAGGCTTTGTTGATTTAC
TACGTGATGATTTTGTTGAAAAAGATCGAAGCCGTGGTATTTATTTCACT
CAAGATTGGGTCTCTCTACCAGGTGTTCTGCCTGTGGCTTCAGGGGGTAT
TCATGTTTGGCATATGCCTGCTCTGACCGAGATCTTTGGAGATGATTCCG
TACTACAGTTCGGCGGAGGAACTTTAGGACACCCTTGGGGAAATGCGCCC
GGTGCCGTAGCTAATCGAGTAGCTCTAGAAGCATGTGTACAAGCCCGTAA
TGAGGGACGTGATCTTGCTCGTGAGGGTAATGAAATTATCCGTGAGGCTA
GTAAATGGAGTCCTGAACTAGCTGCTGCTTGTGAAGTATGGAAGGAGATC
AAATTTGAATTCGAAGCAATGGATACTTTGTAA----------------
----------------------------TCAGTCTCTCCTCT-------
-ACAGCTGAG-CTATCCCGACCATTCACGACGCATCATCC----------
--TCAT-TTTAATAGATGGCTTGGGTCTATGTCAATTAAAAAGACGAAAA
GGGGGTTACAAAGTCTTATCCAGGCCTTGGTGGAATTTCTTAGATCTTCA
A----------------------------AAAAAAGAAGA-CTTTG---
TAAGTTTCAGTACAGTACGAGCGATAATATGATCA--------------

-----------------------------------TTCCAATTT-AC----
------AAT---------CGGGGTTACTTGCTCAATGATGTTCATTTGTA
CGTGTATCATATATATCGGCT---TGTGAAAAGAAAAAAATTAATGAATG
AGAAACATAACGAATTTTGAACCTCTAAC---GAAATGAGAGTATAGGAT
AAAAATTAGGGAATCAAAAGG--GCCTTTT-TGGGGATAGAGGGACTTGA
ACCCTCACGATTTCTAAAGTCGACGGATTTTCCTCTTACTATAAATTTCA
TTGTTGCCGATATTGACATGTAGAATGGGACTCTATCT------TTATTC
TCGTCCGATTAATAAATTTTTCAA----AAGATCTAT-CAGATTACGATG
GAGTAAATGATTTGATCAATGAATAT----TCCAGATAGTATTTCAGTAC
GT---ATACGTATATATAGGTTTATTCTTCATCCTTTCTGGAGTTTCGAT
GGAAGGATTC--CT
TTACTAACGCAGCGCAGCCAACTCCA----TTTGTTAGAACAGCTTCCAT
TGAGT--------CTCTGCACCTATCCTTTTTTATTATCACTTTCTGAAT
CCTTGTTTGTTTTCAGA---------------AAACAGGATTTGGCTCAG
GATTGCCCATTTGT------AATTCCAGGGTTTCTCTGAATTTGAAAGTT
A-------------TCACTTGGTAGTTTCC--------------------

---AAG
GATCATTGTCGAAA-CCT-GCACAGCAGAACGACCCGTGAACCCGTAAAA
ACACACC--GGGGGGCCCGGG---GCCACAGGCC--CTG-TGCCCTCTT-
TGGTCGGG-GGCTGCA---CCCGTG---ACCCGCCTGCCGGTGCGCGGGT
GCCG--CTCTCCCGGCCGC--ACAAACGAACCCCGGCGCAAACCG-CGCC
-AAGGAAAACCCAACGAATTGGCAT-TGCCCCGTCGCCCCAGAGATGGCG
TGCGGTCGGGGTGTGCGT--CGTGTTC-TGTTAATGTCAAAACGACTCTC
GGCAACGGATATCTCGGCTCTCGCATCGATGAAGAACGTAGCGAAATGCG
ATACTTGGTGTGAATTGCAGAATCCCGTGAACCATCGAATCTTTGAACGC
AAGTTGCGCCCGAAGCCT--TTAGGCCGAGGGCACGTCTGCCTGGGCGTC
ACACAACGTTGCCCCCCCAACCTCGACCCCGA---------GG-------
-----------G---CGGGTGGGCGGATGCTGGCCTCCCGTGCGCCACGG
CGTG--CGGTCGGCCGAAAT--GCGAGTCCTCGGCGATGAGTGCCGTAAC
AGTCGGTGGTTGTCCAACCCTCG-GTGCCCTGTTGCGCGCACGGATCGCC
GTTGCGGATCGACAGACC---CCAA-T-GCGC---CGCAACCGCGGCGTC
TCCAACGCGACCCCAGGTCAGGCGGGGCCACCCGCTGAGTTTAA------

>Phylica_oleifolia

--TCTTCT
TTGCATTTATTAAGGCTTTTTCTTCACGAGTATTATAATTGGAATAGTTT
TATTACT---------------------CA------AAAAAAATCTATTT
CTTTTTTTTTGAAAAGTCATTCAAGATTTTTCTTGTTCCTATATAATTCT
CATGTTTGTGAATACGAATCCATCTTACT----TTTTATACGTAACCAAT
CTTCTCATTTACGATTAACATCTTCTGGGGTTTTTTTTGAGCGAATTTTT
TTCTATGGAAAAATAAAACATCCTGTAGAAGAAGTCTTTTCTAATGATTT
TCCGGCGATCTTATGTTTCTTCACGGAGCCTTTCATGCATTATGTTAGAT
ATCAAGGAAAATCTATTTTGGTTTCAAAAGATACGCCTCTTCTAATGAAT
AAATGGAAACATTACCTTGTCCTTTTATGGCAATGTCATTTTTATGTGTG
GGCTCAACCAGGAAGGATCTATATAAACCAATTAGCCAACCATTCCTTCG
GCTTTTTGGGCTATCTTTCAAGTGTGCGACTAAATCTTGCAGTGGTACGA
AGTCAAATGCTAGAAAATTCGTTTATAATGGATAATGCTATAAAGAAGCT
TGATACATTAGTTCCAATTAGTCCAATGATTGGATCATTGGCTAAAATGA
AATTTTGTAACGCATTAGGACATCCTGTTAGTAAGTCGACCTGGGCCGAT
TCGTCAGATTTTGATATTATCGACCGATTTGCCCGTATATGCAGAAATCT
TTCTCATTATTCCAGCGGATCCTCAAAAAAA-------------------

----------TGTTTATCTATCTGTATGGTCTTGGACCATCAATAATAAT
TTTTCTTTAGAATTTGGCTACTTAATTGATCCACTTACTTCTATTATGTT
AATATTAATCACTACTGTTGGAATTATGGTTCTTATTTATAGTGATAATT
ATATGTCTCATGATCAGGGATATTTGAGATTTTTTGCTTATATGAGTTTT
TTTAATACTTCAATGTTAGGATTAGTTACTAGTTCAAATTTGATACAAAT
TTATTTTTTTTGGGAATTAGTTGGAATGTGTTCTTATCTATTAATAGGTT
TTTGGTTCATCCGGCCTATTGCGGCGAATGCTTGTCAAAAGGCGTTTGTA
ACTAATCGTGTAGGGGATTTTGGTTTATTATTAGGAATTTTAGGTTTTTA
TTGGATAACGGGTAGTTTAGAATTTAGGGATTTGTTTGAAATATTCAATA
ACGTGGTTGATAATAATGAAGTTGATTTTTTATTTGTTACTTTGTGTGCC
TGTCTATTATTTGCCGGTGCAGTTGCTAAATCTGCCCAATTTCCCCTTCA
TGTATGGTTACCCGATGCTATGGAAGGACCTACCCCTATTTCGGCTCTTA
TACACGCCGCTACTATGGTAGCAGCCGGAATTTTTCTTGTAGCTCGGCTT
CTTCCGCTTTTCATAGTTATACCTTTCATAATGAATCTAATAGCTTTTAT
TGGGATAATAACATTACTTTTAGGGGCC----------------------

----------------------CTGCTGTCGAAGTTCCGTCTACAAACGG
ATAAGACTTCG--------TTATTAGTGTATACTGAGTTATTGAACATAA
AGGAGCAATAATCAATTTCTTGTTCTAGCAAGAAGTTTGGTATTGCTCCT
TTT-TTATTTAG--------GAGTTTTT-TTACTTA--AAAAAGTTTTTT
TTCTTTACTTCATCTTTACTTCAACATACCCATATTTTCGTTATATAGAA
ATTTTTATTTTCAATTAAATGTTTCAATTCCATTTTTT------------

----TTTCTTGAAGAATGAACAAAAAAAAAAAAATGGG----GTAAATTGA
CCTATTTGTTTGTA----------ATTTTT----------AATAG-----
GGGCGGATG---

--------------------------------AGTGTTGGATTCAAAGCC
GGCGTTAAAGATTATAAATTGACTTATTACACTCCTGACTATGAAACCAA
AGATACTGATATCTTGGCAGCGTTTCGAGTAACTCCTCAACCTGGAGTTC
CGCCTGAGGAAGCAGGGGCCGCGGTAGCTGCTGAATCTTCTACTGGTACA
TGGACAACTGTATGGACTGACGGGCTTACCAGTCTTGATCGTTACAAAGG
TCGATGCTACCACCTAGAGCCCGTTGCTGGAGAAGAAAATCAATATATTG
CTTATGTAGCTTACCCCTTAGACCTTTTTGAAGAAGGTTCTGTTACTAAC
ATGTTTACTTCCATTGTGGGTAATGTTTTTGGGTTCAAGGCCCTGCGCGC

TCTACGTCTGGAGGATTTGCGAATCCCTCCTGCTTATTCTAAAACTTTCC
AAGGACCGCCTCACGGCATCCAAGTTGAAAGAGATAAATTGAACAAGTAT
GGCCGCCCCCTATTGGGATGTACTATTAAACCTAAATTGGGGTTATCCGC
TAAGAATTACGGTAGAGCAGTTTATGAATGTCTT----------------

GAC--------------ACGAGGATTTTCAGTCCTCTGCTCTA-------
-CCAGCTGAG-CTATCCCGACCCTTCACGACGCATCATCC----------
--TCAT-TTTAATAGATGACTTGGGTCTATGTCAATTAAAAAGACGAAAA
GGGGATTACAAAGTCTTATCCAGGCCTTGGTGGAATTTCTTAGATCTTCA
A-----------------------------AA---AGAAGA-CTTTG---
TAAGTTTCAATACAGTACGAGCGATAATATGATCA---------------

----------------------------------TTCCAATTT-AC----
------AAT---------CGGGGTTACTTGCTAAATGATGTTCATTTGTA
CGTGTATCATATATATCGGCT---TGTGAAAATCAAAAAATTAATGAATG
AGAAACATAACGAATTTTGAACCGCTAAC---GAAATGAGAGTATAAGAT
AAAAAATTAGGGAATCAAATG--G-GTG---GGG--ATAGAGGGACTTGA
ACC---

--------------------------------------GTCGCGGCGACG
GGCGGTTCGCTGCCTGCGACGTCGCGAGAAGTCCACTGAACCTTATCATT
TAGAGGAAGGAGAAGTCGTAACAAGGTTTCCGTAGGTGAACCTGCGGAAG
GATCATTGTCGAAA-CCT-GCTCAGCAGAACGACCCGCGAACCTGTGAAA
AACATACTGGGA---GCGAGG---GGCCTTAGGT--CTC-GAACTCTCT-
TGGTCGG-GGGAGCTG---CATCCCGTGCCTCGCTTGCCGATGCATTGGT
GTTG--CTTTCCAGGCCGC-ACAAACGAACCCCGGCGCCATATCG--CGC
CAAGGAAAATCTAACGAATTGGCAC-TGCCCCTCCGTCCCAGAGATGGTG
TGCGGTTGGGGTGTGCGT--CGTATTC---TATATGTCAAAACGACTCTC
GGCAACGGATATCTCGGCTCTCGCATCGATGAAGAACGTAGCGAAATGCG
ATACTTGGTGTGAATTGCAGAATCCCGTGAACCATCGAGTTTTTGAACGC
AAGTTGCGCCCGAAGCCA--TTAGGCCGAGGGCACGTCTGCCTGGGCGTC
ACACAACGTTGCCCC-CCAACCTC----GAAG------------------
-------------GTGAGGGGGGCGGATGCTGGCCTCCCGTGTGCCATGG
CATG--CGGCTGGTTTGAAT--ATGAGTCCTCGGCGACGAGTGCCGCAAC
AATCGGTGGTTCTCCAACCCTCG-TGGCCCTGTTGCGTGCATAGATCGCC

GCTGTGGCTCGATAGACC---CCAC-TG-CGT---TGCTAATGCAGCGTA
TCCAACGCGACCCCAGTCAGGCGGCTACCCG-------------------

>Phylica_paniculata

--TCTTCT
TTGCATTTATTAAGGCTTTTTCTTCACGAGTATTATAATTGGAATAGTTT
TATTACT---------------------CA------AAAAAAATCTATTT
CTTTTTTTTTGAAAAGTCATTCAAGATTTTTCTTGTTCCTATATAATTCT
CATGTTTGTGAATACGAATCCATCTTACT----TTTTATACGTAACCAAT
CTTCTCATTTACGATTAACATCTTCTGGGGTTTTTTTTGAGCGAATTTTT
TTCTATGGAAAAATAAAACATCCTGTAGAAGAAGTCTTTTCTAATGATTT
TCCGGCGATCTTATGTTTCTTCACGGAGCCTTTCATGCATTATGTTAGAT
ATCAAGGAAAATCTATTTTGGTTTCAAAAGATACGCCTCTTCTAATGAAT
AAATGGAAACATTACCTTGTCCTTTTATGGCAATGTCATTTTTATGTGTG
GGCTCAACCAGGAAGGATCCATATAAACCAATTAGCCAACCATTCCTTCG
GCTTTTTGGGCTATCTTTCAAGTGTGCGACTAAATCTTTCAGTGGTACGG
AGTCAAATGCTAGAAAATTCGTTTATAATGGATAATGCTATAAAGAAGCT
TGATACATTAGTTCCAATTAGTCCAATGATTGGATCATTGGCTAAAATGA
AATTTTGTAACGCATTAGGACATCCTGTTAGTAAGTCGACCTGGGCCGAT
TCGTCAGATTTTGATATTATCGACCGATTTGCCCGTATATGCAGAAATCT
TTCTCATTATTCCAGCGGATCCTCAAAAAAAAAGAGTTTGTAT-------

---TGTTTTACT
GTTAAGTATAGTCATGATTTTTTCAGCCCATATATTTATTCAACAAATCA
ATAACAGTTCTGTTTATCTATCTGTATGGTCTTGGACCATCAATAATAAT
TTTTCTTTAGAATTTGGCTACTTAATTGATCCACTTACTTCTATTATGTT
AATATTAATCACTACTGTTGGAATTATGGTTCTTATTTATAGTGATAATT
ATATGTCTCATGATCAGGGATATTTGAGATTTTTTGCTTATATGAGTTTT
TTTAATACTTCAATGTTAGGATTAGTTACTAGTTCAAATTTGATACAAAT
TTATTTTTTTTGGGAATTAGTTGGAATGTGTTCTTATCTATTAATAGGTT
TTTGGTTCATCCGGCCTATTGCGGCGAATGCTTGTCAAAAGGCGTTTGTA
ACTAATCGTGTAGGGGGATTTTGGTTTATTATTAGGAATTTTAGGTTTTTA
TTGGATAACGGGTAGTTTAGAATTTAGGGATTTGTTTGAAATATTCAATA
ACGTGGTTGATAATAATGAAGTTGATTTTTTATTTGTTACTTTGTGTGCC
TGTCTATTATTTGCCGGTGCAGTTGCTAAATCTGCCCAATTTCCCCTTCA
TGTATGGTTACCCGATGCTATGGAAGGACCTACCCCTATTTCGGCTCTTA
TACACGCCGCTACTATGGTAGCAGCCGGAATTTTTCTTGTAGCTCGGCTT
CTTCCGCTTTTCATAGTTATACCTTTCATAATGAATCTAATAGCTTTTAT
TGGGATAATAACATTACTTTTAGGGGCCACTTTAGCTCTTGCTCAAAAAG
ATATTAAGAGGGGTTTAGCTTATT--------------------------

-----TCCTTTTTTATTTAGGNGNTTTTTTACTTAAA-TAAGTTTTTTTT
TTCTTTACTTCATCTTTACTTCAACATACCCATATTTTCGTTATATAGAA
ATTTTTATTTTCGATTAAATGTTTCAATTCCATTTTTT------------

----TTTCTTGAAGAATGAACAAAAA----AAATGGT----GTAAATTGA
CCTATTTGTTTGTA----------ATTTTA----------AT--------

-------------------------------AAGTGTTGGATTCAAAGCC
GGCGTTAAAGATTATAAATTGACTTATTACACTCCTGACTATGAAACCAA
AGATACTGATATCTTGGCAGCGTTTCGAGTAACTCCTCAACCTGGAGTTC
CGCCTGAGGAAGCAGGGGCCGCGGTAGCTGCTGAATCTTCTACTGGTACA
TGGACAACTGTATGGACTGACGGGCTTACCAGTCTTGATCGTTACAAAGG
TCGATGCTACCACCTAGAGCCCGTTGCTGGAGAAGAAAATCAATATATTG

CTTATGTAGCTTACCCCTTAGACCTTTTTGAAGAAGGTTCTGTTACTAAC
ATGTTTACTTCCATTGTGGGTAATGTTTTTGGGTTCAAAGCCCTGCGCGC
TCTACGTCTGGAGGATTTGCGAATCCCTCCTGCTTATTCTAAAACTTTCC
AAGGACCGCCTCACGGCATCCAAGTTGAAAGAGATAAATTGAACAAGTAT
GGCCGCCCCCTATTGGGATGTACTATTAAACCTAAATTGGGGTTATCCGC
TAAGAATTACGGTAGAGCAGTTTATGAATGTCTT----------------

-----------------------GGTATGGAAACTACCAAGTGATAGCTT
TCAAATTCAGAGAAACCCTGGAATTACAAATGGGCAATCCTGAGCCAAAT
CCTGTTTTCTGAAAACAAACAAGGATTCA-------GAA---A---GTGA
TAATAAAAAAGGATAGGTGCAGAGACTCAATGGAAGCTGTTCTAACAAAT
GG----AGTTGGCTGCGATGCGTT------AGTAAAGGAAT-CCTTCCAT
CGAAATTCCAGAAAGGAGGAAGGATAAACGTATATATACGTATACGTACG
GAAATACTGTCTCCAAACAAAATGATTAATGACGACCCGAATCTTTTTTT
TTTTT--TTAT------TTATATGTTTATATGAAA--------------A
ATGAAAAAATTGTTGTGAA-----TCGATTCCAAGTAAA--------AAA
AAATGGAATATTCGTTGATCAAATCATTTACTCCTTCGTAATCTGAT---
-AGATCTTTTGAAAAATTGAT------TAATCGGACGAGAATAAAGATAG
AGTCCCATTCTACATGTCAA------------TATCGACAACAATGAAAT
TTATAGTAAGAGGAAAATCCGTCGACTTTA-GAAATCGTGAGGGTTCAAG
TCCCTCTATCCCCA------C-CCATTTGATTCCCTAATTTTTTATC---
--CTATACT-C----TCATTTCGTTAGCGGTTCAAAATTCGTTATGTTTC
TCATTC------ATTAATT-TTTT--------------------------
------GAT--TTTCACAAGCCTTGT----GATATATATGATACACG---
----TAC--------AAATGAACATCA--TTTAGCAAGT--AACCCCGAT
TGTAAATTGG-------AATGATC-ATATT--------ACCGCTCGTACT
GTATTGAAA----CTTACAAAGTCTTCTTT----TTG---AAGATCTAAG
AAATT----CCACCAAGGCCTGGATAAGACTTTGTAAT----CCCCTTTT
CGTCTTTTTAATTGACATAGACCCAAGTCATCTATTAA------AATGAG
GATGATGCGTCGTG-----AATGGTCGGGATAGCTCAGCTGGT-------
--CGGCGACGTG
GGCGGTTCGCTGCCTGCGACGTCGCGAGAAGTCCACTGAACCTTATCATT
TAGAGGAAGGAGAAGTCGTAACAAGGTTTCCGTAGGTGAACCTGCGGAAG
GATCATTGTCGAAA-CCT-GCTCAGCAGARCGACCCGCGAACCTGTCAAA
-ACATACTGGGA---GCAAGA---TGCCTTAGGT--CTC-GAACTCTCT-
TGGTCGG-GGGGGCTG---CATCCCGTGCATCGCTTGCCGATGCATTGGT
GTTG--CTTTCCAGGCCGC-ACAAACGAACCCCGGCGCCAAATCG--CGC
CAAGGAAAATCTAACGAATTGGCAC-TGCCTCTCCGCCCCAGAGATGGTG
TGCGGTTGGGGTGTGCGT--CGTATTC---TATATGTCAAAACGACTCTC
GGCAACGGATATCTCGGCTCTCGCATCGATGAAGAACGTAGCGAAATGCG
ATACTTGGTGTGAATTGCAGAATCCCGTGAACCATCGAGTCTTTGAACGC
AAGTTGCGCCCGAAGCCT--TCTGGCCGAGGGCACGTCTGCCTGGGCGTC
ACACAATGTTGCCCCCCCAACCTC----AAAG------------------
-------------GTGAG-GGGGCGGATGCTGGCCTCCCGTGTGCCACGT

CATG--CGGCTGGTTTGAAT--ATGAGTCCTCGGCGACGAGTGCCGCAAC
AATCGGTGGTTCTCCAACCCTCG-TGGCCCTGTTGCGTGCATAGACTGCC
GTTGTGGCTCGATAGACC---CCAC-TG-CGT---TGCTAATGCAGCGTA
TCCAACGCGACCCCAGGTCAGGCGGGGCTACCCGCTGAGTTTAAG-----

>Emmenosperma_alphitonioides

-------------------------ACGAGTATTATAATTGGAATAGTTT
TATTACT---------------------CC------AAAAAAATCTATTT
CTTTTTTTTTGAAAAGTAATTCAAGATTTTTCTTGTTCCTATATAATTCT
CATGTTTGTGAATACGAATCCATCTTACT----TTTTCTCCGTAACCAAT
CTTCTCATTTACGATTAACCTCTTCTGGGGTATTTTTTGAGCGAATTTTT
TTCTATGGAAAAATAAACCATCCTGTAGAAGAAGTCTTTTCTAATGATTT
TCCGGCGATCTTATGGTTCTTCACGGAGCCTTTCATGCATTATGTTAGAT
ATCAAGGAAAATCTATTTGGGTTTCAAAAGATACGCCTCTTCTAATGAAT
AAATGGAAATATTATCTTGTCCTTTTATGGCAATGTCATTTTTATGTGTG
GGCTCAACCAGGAAGGATCTATATAAACCAATTAGCCAACCATCCCTTCG
GCTTTTTGGGCTATCTTTCAAGTGTGCGACTAAATCTTTCAGTGGTACGG
AGTCAAATGCTAGAAAATTCGTTTATAATGGATAATGCTATAAAGAAGCT
TGATACATTAGTTCCAATTAGTCCAATGATTGGATCATTGGCTAAAATGA
AATTTTGTAACGCATTAGGACATCCTGTTAGTAAGTCGACCTGGGCCGAT
TCGTCAGATTTTGATATTATCGACCGATTTGCCCGTATATGCAGAA----

----------------------------AGCAAGTGTTGGATTCAAAGCC
GGTGTTAAAGATTATAAATTGACTTATTACACTCCTGACTATGAAACCAA
AGATACTGATATCTTGGCAGCGTTTCGAGTAACTCCTCAACCTGGAGTTC
CGCCTGAGGAAGCAGGGGCCGCGGTAGCTGCTGAATCTTCTACTGGTACA

TGGACAACTGTATGGACTGACGGGCTTACCAGTCTTGATCGTTACAAAGG
TCGATGCTACCACCTCGAGCCCGTTGCTGGAGAAGAAAATCAATATATTG
CTTATGTAGCTTACCCCTTAGACCTTTTTGAAGAAGGTTCTGTTACTAAC
ATGTTTACTTCCATTGTGGGTAATGTTTTTGGGTTCAAGGCCCTGCGCGC
TCTACGTCTGGAGGATTTGCGAATCCCTCCTGCTTATTCTAAAACTTTCC
AAGGACCACCTCACGGCATCCAAGTTGAAAGAGATAAATTGAACAAGTAT
GGCCGCCCCCTATTGGGATGTACTATTAAACCTAAATTGGGGTTATCCGC
TAAGAATTACGGTAGAGCAGTTTATGAATGTCTTCGCGGTGGACTTGATT
TTACCAAAGATGATGAGAACGTGAATTCCCAACCATTTATGCGTTGGAGA
GACCGTTTCTTATTTTGTGCCGAAGCCCTTTATAAAGCACAGGCTGAAAC
AGGTGAAATCAAAGGGCATTACTTGAATGCTACTGCAGGTACATGCGAAG
AAATGATTAAAAGGGCTGTATTTGCCAGAGAATTGGGAGTTCCTATTGTA
ATGCATGATTACTTAACAGGGGGATTCACTGCAAATACTAGCTTGGCTCA
TTATTGCCGAGATAATGGTCTACTTCTTCACATCCACCGTGCAATGCATG
CAGTTATTGATAGACAGAAAAATCATGGTATGCACTTTCGTGTACTAGCT
AAAGCGTTACGTCTGTCTGGTGGAGATCATATTCACGCTGGTACTGTAGT
AGGTAAACTTGAAGGGGAAAGAGAAATCACTTTAGGCTTTGTTGATTTAC
TACGTGATGATTTCATTGAAAAAGATCGAAGCCGTGGTATTTATTTCACT
CAAGATTGGGTCTCTCTACCAGGTGTTCTGCCTGTGGCTTCAGGGGGTAT
TCATGTTTGGCATATGCCTGCTCTGACCGAGATCTTTGGAGATGATTCCG
TACTACAGTTTGGCGGAGGAACTTTAGGACACCCTTGGGGAAATGCACCC
GGTGCCGTAGCTAATCGAGTAGCTCTAGAAGCATGTGTACAAGCTCGTAA
TGAGGGACGTGATCTTGCTCGTGAGGGTAATGAAATTATCCGTGAGGCTA
GTAAATGGAGTGCTGAACTAGCTGCTGCTTGTGAAGTATGGAAGGAGATC
AAATTTGAATTCGAAGCAATGGATACTTTGTAA-----------------
---TTAATTGGATTGAGCCTTG-GTATGGAAACCTACCAAGTGATAACTT
TCAAATTCAGAGAAACCCTGGAATTACAAATGGGCAATCCTGAGCCAAAT
CCTGTTTTCTGAAAACAAACAAAGATTCA-------GAA---A---GTGA
TAATAAAAAAGGATAGGTGCAGAGACTCAATGGAAGCTGTTCTAACAAAT
GG----AGTTGGCTGCGATGCGTT------AGTAAAGGAAT-CCTTCCAT
CGAAACTCCAGAAAGGATGAAGAATAAACCTATATATACGTATACGTACT
GAAATACTATCTCCAAACCAAACGATTAATGACGACCCGAATCTTTTTTT
T---A---TAT------TCATATGTTTATATGAAA-------------A
ATGAAAGAATTGTTGTGAA-----TCGATTCCAAGTAAA----------A
AAATGGAATATTCATTGATCAAATCATTTACTCCATCGTAATCTGAT---
-AGATCTTTTGAAAAATTGAT------TAATCGGACGAGAATAAAGATAG
AGTCCCATTCTACATGTCAA------------TATCGACAACAATGAAAT
TTATAGTAAGAGGAAAATCCGTCGACTTTA-GAAATCGTGAGGGTTCAAG
TCCCTCTATCCCCAAAAAGGC-CCATTTGATTCCCTAATTTTTTATC---
--CTATACT-C----TCATTCCGCTAGCGGTTCAAAATTCGTTATGCTTC
CCATTC------ATTAATT-TTTT-------------------------
------TCT--TTTCACAAGCCTTGT----GATATATATGATACACG---
----TAC--------AAATGAACATCA--TTGAGCAAGT--AACCCCAAT
TGTAAATTGG-------AATGATC-ATATT--------ATCGCTCGTACT
GTACTGAAA----CTTACAAAGTCTTATTT----T-TGAA--GATCTAAG
AAATT----CCACCAAGGCCTGGATAAGACTTTGTAAT----CCCCTTTT
CGTCTTTTTAATTGACATAGACCCAAGTCATCTATTAA------AATGAG
GGTGATGCGTCGTG-----AATGGTCGGGATAGCTCAGCA----------

-----------------------------------TAACCTGCGGGGAG
GATCATTGTCGAAA-CCT-GCCTAGCAGAACGACCCGCGAACCCGTAAAA
AACATAC-CGGGGGGCTCGGG---GCCCTCGGGC--CTC-GGACTCTCC-
TGGCCGGGGGCGCGCA---TCTCGTGCCTCGCTCGCCGATGCACCGGGTG
CTGG--CTTACCCGGCCGC--ACAAACGAACCCCGGCGCAAACCG-CGCC
AAGGAACATCTAAACGAATTGGCAC-TGCCCCGCCGCCCCAGAGATGGCG
CGCGGCCGGGGTGTGCGT--CGTATTC-TGTATATGTCAAAACGACTCTC
GGCAACGGATATCTCGGCTCTCGCATCGATGAAGAACGTAGCGAAATGCG
ATACTTGGTGTGAATTGCAGAATCCCGCGAACCATCGAGTTTTTGAACGC
AAGTTGCGCCCGAAGCCA--TTAGGCCGAGGGCACGTCTGCCTGGGCGTC

ACACAACGTTGCCCCCCCAACCCCGACCTCGA---------AG-------
-----------GCGAGG---GGGCGGATGATGGCC-------TCCCGCGT
ACCG--CGGATGGCCGAAAT--GCGAGTCCTCGGCGACGAGTGCCGCAAC
AATCGGTGGTTCTCCAACCCTCG-GTGCCCTGTTGCGTGCACGGATCGCT
GCTGCGGCACGATAGACC---CCGA-TG-CGC---TGCTAGTGCGGCGTC
TCCAGCGCGACCTCAGGTCAGCGGGGCTA--------------------

>Alphitonia_oblata

----------GAAAAGTAATTCAAGACTTTTCTTGTTCCTATATAATTCT
CATGTTTGTGAATATGAATCCATCTTACT----TTTTCTCCGTAACCAAT
CTTCTCATTTACGATTAACATCTTCTGGGGTATTTTTTGAGCGAATTTTT
TTCTATGGAAAAATAAAACATCCTGTAGAAGAAGTCTTTTCTAATGATTT
TCCGGCGATCTTATGGTTCTTCACGGAGCCTTTCATGCATTATGTTAGAT
ATCAAGGAAAATCTATTTGGGTTTCAAAAGATACGCCCCTTCTAATGAAT
AAATGGAAATATTATCTTGTCCTTTTATGGCAATGTCATTTTTATGTGTG
GGCTCAACCAGGAAGGATCTATATAAACCAATTAGCCAACCATTCCTTCG
GCTTTTTGGGCTATCTTTCAAGTGTGCGACTAAATCTTTCAGTGGTACGG
AGTCAAATGCTAGAAAATTCATTTATAATGGATAATGCTATAAAGAAGCT
TGATACATTGGTTCCAATTAGTCCAATGATTGGATCATTGGCTAAAATGA
AATTTTGTAACGCATTAGGACATCCTGTTAGTAAGGCGACCTGGGCCGAT
TCGTCAGA--

------------CTAGATCTAGCTGCTGTCGAAGTTCCGTCTACAAACGG
ATAAGACTTCG--------GTATTAGTGTATACTGAGTTATTGAACATAA
AGGAGCAATAATCAATTTCTTGTTCTAGCAAGAAGTTTGGTATTGCTCCT
TTT-TTATTTATTTAGGA-------------------------GTTTTTT
TTCTTTACTTCATCTTTACTTCAACATACCCATCCTTTCGTTATGTAGAA
ATT-----TTTATTTTACATTTAAGGGTTTCAATTCAATTTTTTT-----
---CTTGTAGTT
TCTCTTAGAAGAATGAAAAAAAAAAATGATAAATGGT----GTAAATTGA
CCTATTTGTTTGTA----------ATAT---------------GTAATAG
GGGCGGATGTAGCCA-----------------------------------

---CAATGCC
GGTGTTAAAGATTATAAATTGACTTATTACACTCCTGACTATGAAACCAA

AGATACTGATATCTTGGCAGCGTTTAGAGTAACTCCTCAACCTGGAGTTC
CGCCTGAGGAAGCAGGGGCCGCGGTAGCTGCTGAATCTTCTACCGGTACA
TGGACAACTGTATGGACTGACGGGCTTACCAGTCTTGATCGTTACAAAGG
TCGATGCTACCACCTCGAGCCCGTTGCTGGAGAAGAAAATCAATATATTG
CTTATGTAGCTTACCCCTTAGACCTTTTTGAAGAAGGTTCTGTTACTAAC
ATGTTTACTTCCATTGTGGGTAATGTTTTTGGGTTCAAGGCCCTGCGCGC
TCTACGTCTGGAGGATTTGCGAATCCCTACTGCTTATACTAAAACCTTCC
AAGGACCGCCTCACGGCATCCAAGTTGAAAGAGATAAATTGAACAAGTAT
GGCCGCCCTCTATTGGGATGTACTATTAAACCTAAATTGGGGTTATCCGC
TAAGAATTACGGTAGAGCGGTTTATGAATGTCTTCGCGGTGGACTTGATT
TTACCAAAGATGATGAGAACGTGAATTCCCAACCATTTATGCGTTGGAGA
GACCGTTTCTTATTTTGTGCCGAAGCCCTTTATAAAGCACAGGCTGAAAC
AGGTGAAGTCAAAGGGCATTACTTGAATGCTACTGCAGGTACATGCGAAG
AAATGATGAAAAGGGCTATATTTGCCAGAGAATTGGGAGTTCCTATTGTA
ATGCATGATTACTTAACAGGGGGGTTCACTGCAAATACTAGCTTGGCTCA
TTATTGCCGAGATAATGGTCTACTTCTTCACATCCACCGTGCAATGCATG
CAGTTATTGATAGACAGAAAAATCATGGTATGCACTTTCGTGTACTAGCT
AAAGCGTTACGTCTGTCTGGTGGAGACCATATTCACGCCGGTACTGTAGT
AGGTAAACTTGAAGGGGAAAGAGACATCACTTTAGGCTTTGTTGATTTAC
TACGTGATGATTTTATTGAAAAAGACCGAAGCCGTGGTATTTATTTCACT
CAAGATTGGGTCTCTCTACCAGGTGTTTTGCCTGTGGCTTCGGGGGGTAT
TCATGTTTGGCATATGCCTGCTCTGACCGAGATCTTTGGAGATGATTCCG
TACTACAGTTTGGCGGAGGAACTTTAGGACACCCTTGGGGAAATGCACCC
GGTGCCGTAGCTAATCGAGTAGCTCTAGAAGCATGTGTACAAGCTCGTAA
TGAAGGGCGTGATCTTGCTCGTGAAGGTAATGAAAATATCCGTGAGGCTA
GTAAATGGAGTCCTGAACTAGCTGCTGCTTGTGAAGTATGGAAGGAGATC
AAATTTGAATTCGAAGCAATGGATACTTTGTAA----------------
--AATTAATTGGATTGAGCCTTGGTATGGAAACTTACCAAGTGATAACTT
TCAAATTCAGAGAAACCCTGGAATTACAAATGGGCAATCCTGAGCCAAAT
CCTGTTTTCTGAAAACAAACAAGGATTCA-------GAA---A---GTGA
TAATAA-AAGGGATAGGTGCAGAGACTCAATGGAAGCTGTTCTAACAAAC
AAATGGAGTTGGCTGCGGTGCGTTAGTAAAAGTAAAGGAAT-CCTTCCAT
CGAAACTCCAGAAAGGATGAAGAATAAGCCTATGTATACGTATACGTGCT
GAAATACTATCTCCAAACCAAATGATTAATGACGACCCGAATCTTTTTTT
T--ATATTTAT------ATGTTTATATGAAAAATG-AAA-----------
------GAATTGTTGTGAA-----TCGA---TTCCAAGTAAAAAA--AAA
AAACGGAATATTCATTGATCAAATCATTTACTCCATCGTAATCTGAT---
-AGATCTTTTGAAAAATTGAT------TAATCGGACGAGAATAAAGATAG
AGTCCCATTCTACATGTCAA------------TATCGACAACAATGAAAT
TTATAGTAAGAGGAAAATCCGTCGACTTTA-GAAATCGTGAGGGTTCAAG
TCCCTCTATCCCCAAAAAGGC-CCATTTGATTACCTAATTTTTTATC---
--CTA-----TACTCTCATTTCGTTAGCGGTTCAAAATTCGTTATGTTTC
GCATTCATTAATCTTTTTCTTTTC-------------------------
------ACAAGCCTTGTG-------------GTATATATGATACACG---
----TAC--------AAATGAACATCG--TTGAGCAAGT--AACCCCGAT
TGTAAATTGG-------AATAATC-ATATT--------ATTGCTCGTACT
GAAACTTA---------CAA-------AGTCTTCTTTTTGAAGATCTAAG
AAATT----CCACCAAGGCCTGGATAAGACTTTGTAAT----CCCCTTTT
CGTCTTTTTAATTGACAGAGCC-CAAGTCATCTGTTAA------AATGAG
GATGATGCGTCGTG-----AATGGTCGGGATAGCTCAGCTGGTAGAGCAG
AGGACTGAAAATC----------------------GATCGAGGCGACGTG
GGCGGTTCGCTGCCCGCGACGTCGCGAGAAGTCCACTGAACCTTATCATT
TAGAGGAAGGAGAAGTCGTAACAAGGTTTCCGTAGGTGAACCTGCGGAAG
GATCATTGTCGAAA-CCT-GCCTAGCAGAGCGACCCGCGAACCCGTA--A
AAACAAAACGGGGGGCTCGGG---G-CCTTAGGC--CCT-AGGCTCTCT-
TGGTCGGGGGGTTGCA---CCTTGTGCCTTG--CTTGTCGACGCACATGT
GCTG--CTTTCCCGGCCGC-AAAAAATGAACCCCGGCGCAAACCG-CGCC
-AAGGAACATCTAACGAATTGGCAC-TGCCCCACCGCCCCAGAGATGGTG
TGCGGTCGGGGTGTGCGT--CGTATTC---TATATGTCAAAACGACTCTC
GGCAACGGATATCTCGGCTCTCGCATCGATGAAGAACGTAGCGAAATGCG

ATACTTGGTGTGAATTGCAGAATCCCGCGAACCATCGAGTTTTTGAACGC
AAGTTGCGCCCGAAGCCA--TTAGGCCGAGGGCACGTCTGCCTGGGCGTC
ACACAACGTTGCCCCCC-CAACCTCGACCTCG---------AA-------
-----------GGCGAGGGGGGGCGGATGATGGCCTCCCGTGTGCCACGG
TTTG--CGGCTGGCCGAAAT--ACGAGTCCTCGGCGACGAGTGCCGCAGC
AATCGGTGGTTCTCCAACCCTCG-GTGCCCTGCTGCGTGCACGGACTGCC
GTTGTGACCCGATAAGAC---CCCA-ATGCGC---CGCAAATGCGGCGTC
TCCAACGCGACCCCAGGTCAGGCGGGGCCACCCGCTGAGTTTAAGCATAT
CAATAAGCGGAGGAAAAGAAACTTACAAGGATTCCCCTA-----------

>Colletia_spinosa

-----------------TCGCCGTCTGTGGGTTTTTCCTAGTGTTTTACT
TTTAAGTATAGTTATGATTTTTTCAGCTCATATATTTATTCAACAAATAA
ATAACAGTTCTATCTATCTATCTGTAGGATCTTGGACTATCAATAATGAT
TTTTCTTTAGAATTTGGGTACTTAATTGATCCACTTACTTCTCTTATGTT
AATTTTAATCACTACTGTTGGAATTATGGTTCTTATTTATAGTGATAATT
ATATGTCTCATGATCAGGGATATTTGAGATTTTTTGCTTATATGAGTTTT
TCTACTACTTCAATGTTAGGATTAGTTACTAGTTCAAATTTGATACAAAT
TTATTTTTTTTGGGAATTAGTTGGAATGTGTTCTTATCTATTAATAGGTT
TTTGGTTCACCCGACCTATTGTGGCGAATGCTTGTCAAAAGGCGTTTGTA
ACTAATCGTGTAGGAGATTTTGGTTTATTATTAGGAATTTTAGGTTTTTA
TTGGATAACGGGTAGTTTAGAATTTAGGGATTTGTTTGAAATATTCCATA
ACGTGGTTGATAATAATGAAGTTGATTTTTTATTTGTTTTTTTGTGTGCC
TGTCTATTATTTGCCGGTGCTGTGGCTAAATCTGCCCAATTTCCCCTTCA
TGTATGGTTACCCGATGCTATGGAAGGACCTACCCCTATTTCGGCTCTTA
TACACGCTGCTACTATGGTAGCAGCCGGGATTTTTCTTGTAGCTCGGCTT

ATGCCACTTTTTATAGTTATACCTTTCATAATGTCTATAATAGCTTTTAT
TGGGATAATAACATTACTTTTAGGAGCCACTTTAGCTCTTGCTCAAAAAG
ATATTAAGAGGGGTTTAGCTTATTCTACAATGTCTCAATTGGGTTATATG
ATGTTGGCTCTAGGTATGGGGTCTTATCGAGCTGCTTTATTTCATTTGAT
TACTCATGCTTATTCGAAAGCATTGTTGTTTTTAGGATCCGGATCAATTA
TTCATTCCATGGAAACAATTGTTGGATATTCTCCAGATAAAAGCCAGAAT
ATGGTT--

-----------------------------------GTTGGATTCAAAGCC
GGTGTTAAAGATTATAAATTGACTTATTACACTCCTGACTATGAAACCAA
AGATACTGATATCTTGGCAGCGTTTCGAGTAACTCCTCAACCTGGCGTTC
CGCCTGAGGAAGCAGGGGCCGCGGTAGCTGCTGAATCTTCTACTGGTACA
TGGACAACTGTATGGACTGACGGGCTGACCAGTCTTGATCGTTACAAAGG
TCGATGCTACCACCTCGAGCCCGTTGCTGGAGAAGAAAATCAATATATTG
CTTATGTAGCTTACCCCTTAGACCTTTTTGAAGAAGGTTCTGTTACTAAC
ATGTTTACTTCCATTGTGGGTAATGTTTTTGGGTTCAAGGCCCTGCGCGC
TCTACGTCTGGAGGATTTGCGAATCCCCCCTGCTTATTCTAAAACTTTCC
AAGGACCGCCTCACGGCATCCAAGTTGAAAGAGATAAATTGAACAAGTAT
GGCCGTCCCCTATTGGGATGTACTATTAAACCTAAATTGGGGTTATCCGC
TAAGAATTACGGTAGAGCAGTTTATGAATGTCTTCGCGGTGGACTTGATT
TTACCAAAGATGATGAGAACGTGAATTCCCAACCATTTATGCGTTGGAGA
GACCGTTTCTTATTTTGTGCCGAAGCCCTTTATAAAGCACAGACTGAAAC
AGGTGAAATCAAAGGGCATTACTTGAATGCTACTGCAGGTACATGCGAAG
AAATGATGAAAAGGGCTATATTTGCCAGAGAATTGGGAGTTCCTATTGTA
ATGCATGATTACTTAACAGGGGGATTCACTGCAAATACTAGCTTGGCTCA
TTATTGCCGAGATAATGGTCTACTTCTTCACATCCACCGTGCAATGCATG
CAGTTATTGATAGACAGAAAAATCATGGTATACACTTTCGTGTACTAGCT
AAAGCGTTACGTATGTCTGGTGGAGATCATATTCACGCTGGTACTGTAGT
AGGTAAACTTGAAGGGGAAAGAGACATCACTTTAGGCTTTGTTGATTTAC
TACGTGATGATTTTATTGAAAAAGATCGAAGCCGTGGTATTTATTTCACT
CAAGATTGGGTCTCTCTACCAGGTGTTCTGCCTGTGGCTTCAGGGGGTAT
TCATGTTTGGCATATGCCTGCTCTGACCGAGATCTTTGGAGATGATTCCG
TACTACAGTTTGGCGGAGGAACGTTAGGACACCCTTGGGGAAATGCACCC
GGTGCCGTAGCTAATCGAGTAGCTCTAGAAGCATGTGTACAAGCTCGTAA
TGAGGGACGTGATCTTGCTCGTGAGGGTAATGAAATTATCCGTGAGGCTA
GCNNNTGGAGTGCTGAACTAGCTGCTGCTTGTGAAGTATGGAAGGAGATC
AAATTTGAATTCGAAGCAATGGATACTTTGTAA---------------GT
GAC--------------ACGAGGATTTTCAGTCCTTTGCTCTG-------
-CCAGCTGAG-CTATCCCGACCATTCACGACGCATCATCC----------
--TCAT-TTTAATAGATGACTTGGGTCTATGTCAATTAAAAAAACGAAAA
GGGGATTACAAAGTCTTATCCAGGCCGTGGTGGAATTTCTTAGATCTTCA
A-----------------------------CA---AGAATA-CTTTG---
TAAGTTTCAGTACAGTACGAGCGATGATCTGATCA---------------

----------------------------------TTCA-ATTT-AC----
------AAT---------CGGGGTTACTTGCTCAATGATGTTCATTTGTA
CGTGTATCATATATACCGGCT---TGTGAAAAGAAAAAAATGAATGAATG
AGAAACCTAACGAATTTTGAACCACTAAT---GAAATGAGAGTATAGGAT
AAAAAAAAAATGAGGGAATCA--AATGG---GCCTTTTTGGGGATAGAGG
GACTTGAACC--

GATCATTGTCGAAA-CCT-GCCCAGCAGAACTACCCGCGAACTTGTAAAA
ACACCCTCGGAGGGCCCTCGG---G------------------CTCTCT-
TGGCCGGGGCGGCTGC---TCCCCCGCGCACTGCGTGTCTCTGCGCGGGC
GCCG--CTTCCCCGGCTGC--ACAAACGAACCCCGGCGCAAACCG-CGCC
-AAGGAACATCTAACGGATTGGCAC-CGCCCCGACGCCCCAGAGATGGCG

TGCGGCAGGGGCGCGCGC--CGTATTCAACAATATGTCAAAACGACTCTC
GGCAACGGATATCTCGGCTCTCGCATCGATGAAGAACGTAGCGAAATGCG
ATACTTGGTGTGAATTGCAGAATCCCGTGAACCATCGAGTTTTTGAACGC
AAGTTGCGCCCGAAGCCA--TTAGGCCGAGGGCACGCCTGCCTGGGCGTC
ACACAATGTTGCCCCCCTCCAACCTC-TCCGT---------CG-------
-----------GAGGCGAGGGGGCGGATGCTGGCCTCCCGTGTGCTGCGC
CACG--CGGCTGGCCAAAAT--GAGAGTCCTCGGCGACGAGTGCCGCAGC
AATCGGTGGTTTTCCAACCCTCG-GTGCCCCGCTGCGCGCGCGGACCGCC
GTCGTGGCCCGATAGACC---CCAA-TGCGGC---GC-GGAAGCGCCGTC
TCCAATGCGAC---------------------------------------

>Phylica_emirnensis

---------CTGTTTATCTATCTGTATGGTCTTGGACCATCAATAATGAT
TTTTCTTTAGAATTTGGCTACTTAATTGATCCACTTACTTCTATTATGTT
AATATTAATCACTACTGTTGGAATTATGGTTCTTATTTATAGTGATAATT
ATATGTCTCATGATCAGGGATATTTGAGATTTTTTGCTTATATGAGTTTT
TCTAATACTTCAATGTTAGGATTAGTTACTAGTTCAAATTTGATACAAAT
TTATTTTTTTTGGGAATTAGTTGGAATGTGTTCTTATCTATTAATAGGTT
TTTGGTTCATCCGGCCTATTGCGGCGAATGCTTGTCAAAAGGCGTTTGTA
ACTAATCGTGTAGGGGATTTTGGTTTATTATTAGGAATTTTAGGTTTTTA
TTGGATAACGGGTAGTTTAGAATTTAGGGATTTGTTTGAAATATTCAATA
ACGTGGTTGATAATAATGAAGTTGATTTTTTATTTGTTACTTTGTGTGCC
TGTCTATTATTTACCGGTGCAGTTGCTAAATCTGCCCAATTTCCCCTTCA

TGTATGGTTACCCGATGCTATGGAAGGACCTACCCCTATTTCGGCTCTTA
TACACGCCGCTACTATGGTAGCAGCCGGAATTTTTCTTGTAGCTCGGCTT
CTTCCGCTTTTCATAGTTATACCTTTCATAATGAATCTAATAGCTTTTAT
TGGGATAATAACATTACTTTTAGGGGCC----------------------

------------------------GCTGTCGAAGTTCCGTCTACAAACGG
ATAAGACTTCG--------TTATTAGTGTATACAGAGTTATTGAACATAA
AGGAGCAATAATCAATTTCTTGTTCTAGCAAGAAGTTTGGTATTGCTCCT
TTT-TTA--------TTTAGGAGTTTTT-TTACTTAA-ATAAGTTTTTTT
TTCTTTACTTCATCTTTACTTCAACATACCCATATTTTCGTTATATAGAA
ATTTTTATTTTCGATTAAATGTTTCAATTCCATTTTT-------------

----TTTCTTGAAGAATGAACAAAAAAGATAAATGGT----GTAAATTGA
CCTATTTGTTTGTA----------ATTTTT----------AATAG-----
GGGCGGATGTAGCCAAGTGGATC---------------------------


```
--------------------------------------------------
--------------------------------------------------
--------------------------------------------------
--------------------------------------------------
--------------------------------------------------
--------------------------------------------------
--------------------------------------------------
--------------------------------------------------
--------------------------------------------------
--------------------------------------------------
--------------------------------------------------
--------------------------------------------------
--------------------------------------------------
--------------------------------------------------
--------------------------------------------------
--------------------------------------------------
--------------------------------------------------
--------------------------------------------------
--------------------------------------------------
--------------------------------------------------
--------------------------------------------------
--------------------------------------------------
--------------------------------------------------
--------------------------------------------------
--------------------------------------------------
--------------------------------------------------
--------------------------------------------------
--------------------------------------------------
--------------------------------------------------
--------------------------------------------------
------------------------------------------------GT
GAC--------------ACGAGGATTTTCAGTCCTCTGCTCTA-------
-CCAGCTGAG-CTATCCCGACCATTCACGACGCATCATCC----------
--TCAT-TTTAATAGATGACTGGGGTCTATGTCAATTAAAAAGACGAAAA
GGGGATTACAAAGTCTTATCCAGGCCTTGGTGGAATTTCTTAGATCTTCA
A-----------------------------AA---AGAAGA-CTTTG---
TAAGTTTCAATACAGTACGAGCGATAATATGATCA---------------
--------------------------------------------------
--------------------------------------------------
----------------------------------TTCCAATTT-AC----
------AAT---------CGGGGTTACTTGCTAAATGATGTTCATTTGTA
CGTGTATCATATATATCGGCT---TGTGAAAATCAAAAAATTAATGAATG
AGAAACATAACGAATTTTGAACCGCTAAC---GAAATGAGAGTATAGGAT
AAAAAATTAGGGAATCAAATG--G--------------------------
--------------------------------------------------
--------------------------------------------------
--------------------------------------------------
--------------------------------------------------
--------------------------------------------------
--------------------------------------------------
--------------------------------------------------
--------------------------------------------------
--------------------------------------------------
--------------------------------------------------
--------------------------------------------------
----------------------------------------------CATT
TAGAGGAAGGAGAAGTCGTAACAAGGTTTCCGTAGGTGAACCTGCGGAAG
GATCATTGTCGAAA-CCT-GCTCAGCAGAACGACCCGCGAACCTGTCAAA
-ACATACTGGGA---GCGAGA---GTCCTTAGGT--CTC-GAACTCTCT-
TGGTCGGGGGGGGCTG---CATCCCGTGCATCGCTTGCCGATGCATTGGT
```

GTTG--CTTTCCAGGCCGC-ACAAACGAACCCCGGTGCCAAATCG--CGC
CAAGGAAAATCTAACGAATTGGCAC-TGCCCCTCCGCCCCAGAGATGGTG
TGCGGTTGGGGTGTGCGT--CGTATTC---TATATGTCAAAACGACTCTC
GGCAACGGATATCTCGGCTCTCGCATCGATGAAGAACGTAGCGAAATGCG
ATACTTGGTGTGAATTGCAGAATCCCGTGAACCATCGAGTCTTTGAACGC
AAGTTGCGCCCGAAGCCA--TTAGGCCGAGGGCACGTCTGCCTGGGCGTC
ACACAACATTGCCCCCCCATCCTC----GAAG------------------
-------------GTGAGGGGGGCGGATGCTGGCCTCCCGTGTGCCATGG
CATG--CGGCTGGTTTGAAT--ATGAGTCCTCGGTGACGAGTGCCGCAAC
AATCGGTGGTTCTCCAACCTTCG-TGGCCCTGTTGCGTGCATAGACCGCC
GTTGTGGCTCGATAGACC---CCAC-TG-CGT---TGCTAATGCAGCGTA
TCCAACGCGACCCC------------------------------------

>Rhamnidium_elaeocarpum

--------------------------------------ATTCATATATAC
AATGAT---AATATATATCAGAAAAAGATTGCCCTTTTATTTTAAAATAA
AAATGAAATA------------AAATGAAACATTCTTAAGAGTATAATAT
TGGAAAAA------------------TTGGATTATTATTCTTAATACTAT
AATTCTTTTCTGAATCATATCAATTTCACAAATTGAATCGTATTCAAATA
ACACACAGAGGGAATTGAATCTATTTGTCATCCCTAAATGTTAAATATTT
AACTTTAATATAGAATATCTAGAAT------TAGTAACAATTGTTTAGTC
TATCTGATAAATAT----GAGG-------GTCCCATATT------ATTGT
TTTTGGGA--TTCTGGTTTTATCAAACAGTATGAAAAAATCACAACCCTC
CCTTACATCAGTCTTTATCCACTATTTCACTAAAAAAAGAAAGAATTTCT
TTTTGATTTAGAATT-TT-------TTTTA----------TA--TTATAT
GTTTTTTATTTCTATAACATTC------AAAA------------------
--------------------------AAAAAA----TTCTAATATTAAC-
--------------------TTAATAAATATAGAAAAAAAATGGAATAGA
AATATGCC--------ATTTTAAGATTAATA-------------------

------------------------------A---------------ATAT

ATGTATATGGTATTAAATTGAATTCTTTCTGAGACTTCGTCAGAAACCAG
CCATTCATATTTCATATAGAAATAAAGG-------------------GTA
TAGATTATTATGTACTGGCCGAACCAATGACTATTCGTGATTCCATAATT
GAATCAATTACATA----------------------------------
-------------------------TAAAGCAAGTGTTGGATTCAAGGCT
GGTGTTAAAGATTATAAATTGACTTATTACACTCCTGAATATGAAACCAA
AGATACCGATATCTTGGCAGCATTTCGAGTAACTCCTCAACCCGGAGTTC
CACCTGAAGAAGCAGGGGCCGCGGTAGCTGCTGAATCTTCTACTGGTACA
TGGACAACTGTATGGACTGACGGGCTTACCAGTCTTGATCGTTACAAAGG
TCGATGCTACCACATCGAGCCCGTTGCTGGAGAAGAAAGTCAATTTATTG
CTTATGTAGCTTACCCCTTAGACCTTTTTGAAGAAGGTTCTGTTACTAAC
ATGTTTACTTCCATTGTGGGTAATGTATTTGGGTTCAAGGCCCTGCGCGC
TCTACGTCTGGAGGATTTGCGAATCCCCACTGCTTATACTAAAACTTTCC
AAGGCCCGCCTCATGGCATCCAAGTTGAGAGAGATAAGTTGAACAAGTAT
GGCCGCCCCCTATTGGGATGTACTATTAAACCGAAATTGGGGTTATCCGC
TAAGAATTACGGTAGAGCCGTTTATGAATGTCTTCGCGGTGGACTTGATT
TTACCAAAGATGATGAGAACGTGAATTCCCAACCGTTTATGCGTTGGAGA
GACCGTTTCTTATTTTGTGCCGAAGCAATTTATAAAGCACAGGCCGAAAC
TGGTGAAATCAAAGGGCATTACTTGAATGCTACGGCAGGTACATGCGAAG
AAATGATGAAAAGGGCTATGTGTGCCAGAGAATTGGGAGTTCCTATCGTA
ATGCACGATTACTTAACGGGGGGATTCACTGCAAATACTACCTTGGCTCA
TTATTGCCGAGATAATGGTCTGCTTCTTCACATCCACCGTGCAATGCATG
CCGTTATTGATAGACAGAAAAATCATGGTATGCACTTTCGTGTACTAGCT
AAAGCGTTACGTATGTCTGGTGGAGATCATATTCACGCTGGTACTGTAGT
GGGTAAACTTGAAGGGGAAAGAGACATCACTTTAGGCTTTGTTGATTTAC
TACGTGATGATTTTATTGATAAAGATCGAAGCCGTGGTATTTATTTCACT
CAAGATTGGGTCTCTCTACCAGGTGTTTTGCCCGTGGCTTCAGGGGGTAT
TCATGTTTGGCATATGCCTGCTCTGACCGAGATCTTTGGGAGACATTCCG
TACTACAATTCGGCGGAGGAACTTTAGGGCACCCTTGGGGAAATGCACCC
GGTGCCGTAGCTAATCGAGTAGCTCTAGAAGCATGTGTACAACCTCGTAA
TGAGGGACGTGATCTTGCTCGTGAAGGTAATGAAATTATCCGTGAGGCTA
GTAAATGGAGTGCTGAACTAACTGCTGCTTGTGAAGTATGGAAGGAGATG
AAATTTGAATTCCCAGCAATGGATACTTTGTAA-----------------
---TTAATTGGATGCAGCCTTG--GTATGGAACCTACCGAGTGATAACTT
TCAAATTCAGAGAAACCCTGGAATTAAAAACGGGCAATCCTGAGCCAAAT
CCTGTTTTCTGAAAACAAACAAAGGTTCAGAAAGCGATAATAAAAAA--G
GATAGA-TAGGGATAGGTGCAGAGACTCAATGGAAGTTGTTCTAACAAAT
GG----AGTTGGCCACGATGTGTT------AGTAAAGGACT-CCTTCCAT
CGAAACTCCAGAAAGTATGAAGAATAAACGTATATATACGTACT------
GAAATACTATCTCCAAACCAAATGATTAATGACGACCCGAATCTTTTTTT
T----TATTTATATAAAA------------------------------A
ATGAAAGACTAGTTGT----------GAATCGATTCCAAGTTG-AAAACA
GAATCGAATATTCATTGATCAAATCATTTACTCCATCGTAATCTGAT---
-AGATCTTTTGAAGAATTGAT-----T-AATCGGACGAGAATAAAGATAG
AGTCCCGTTCTACATGTCAA------------TATCGACAACAATGCAAT
TTATAGTAAAAGGAAAATCCGTCGACTTTA-GAAATCGTGAGGGTTCAAG
TCCCTCTATCCCCAAAAAGTC-CCATTGGATTCCCTAATTATTTATC---
-------CTATGCTCTCATTTCGTTAACGGTTCAAAATTCGTTATGTTTC
TCATTCATTCTACTCTTTTACTTTATAAATGGTCTGAGCGGAAATTTTTT
TCT--------TTTC------------------ACAAGCCTTGTGATAT
ATAGGATACACGTACAAATGAACATCGTTGGGCACGT----AACCCCGAT
TGTAAATTGT-------AATGATT-AACAATACATATTATTACTTGTACT
GTACTGAAACTTACA----AAGTC------TTCTTTTTG-AAGATCCAAG
AAATT----CCACCAAGACTCGGATAAGGTTTTGTAATC----CCCTTTT
CGTCTTTTTCATTGACATAGAACTAAGTCCTCTATTAATATTAAAATGAG
GATGGTGCGTCGTG-----AATGGTCGGGATAGCTCAGCTGGTAGAGCGA
GAGGACTGAATA--------------------------------------

--G
GATCATTGTGG--AAACCTTCACAGCAGAACGACCCGTGAACTTATAAAA

CACAACTTGGGGGCGAGGGGT---CAACCCGAAC--CCC-C----TTGGT
GG-GTGG--GCGGTGG---G-------TC---GGGCATTGATGCCCGATG
TTACTGTCTTCCC-CGCTA-TACAAATGAACCCCGGCGCAGGACGCGCCA
--AGGAACTCAAACGAATCAAG--CATGCCCCGGCGCCCCAGAGATGGTA
TGCGTTCGGGGTGCTGCA--TCGTAT--TCTATATGTCAAAACGACTCTC
GGCAACGGATATCTCGGCTCTCGCATCGATGAAGAACGTAGCGAAATGCG
ATACTTGGTGTGAATTGCAGAATCCCGTGAACCATCGAGTCTTTGAACGC
AAGTTGCGCCCGAAGCCA--CTAGGCTGAGGGCACGTCTGCCTGGGCGTC
ACACAACGTTGCCCCCCAACCCCACCCCGTCTTCT-----GGG-------
-----------GGAGGGAGGGGGCGGATGCTGGCCTCCCGTGCGCAATTG
CTCG--CGGTTGGCCCAAAT--GCAAGCACCCGGCGACGAGCGCCGCGGC
AATCGGTGGCTGTCCAACCCTCG-GTGCAACGCCGCGCGCGCGAGCTGCT
CAACGGTGCTGGTGACCC---CCAA--TGCGCT---G-------------

>Condalia_microphylla

----------------------------------CTTAAGAGTATAATAT
TGGAAAAATTG------------------GATTATTATTCTTAATACTAT
AATTCTTTTCTGAATCATATCAATTTCACAAATTGAATCGTATTCCAATA
ACACACAGAGGGAATTGAATCTATTTGTCATCCCTAAATTTTTAA-----
-TATTTAACATCGAATATCTAGAATTCATTTCAGTAACAATTGTTTAGTC
TATCTGATAAATAT----GAGG-------GTCCCATATTATTGTTTTTGG
AATTCTGGTTTTATC--------AAACAGTATGAAAAAATAACAACCCGC
CCTTACATCAGTCTTTATCCACTATTTCACTAAAAAAGAAATAATTTCTT
TTTCATTTTTTTTTTTTATTAATATTAT--------------------A-
-TGTTTTATATGTTTTTTATTTCTATA-----------------------
--------------------------ACATTA----AAAAAAAATGAAA-
--------------------ATATTAACTTAATAAATAAAATGGAATTGA
AATATTCC--------ATTTTAAGATTAATA-------------------

------------------------------A---------------ATAT
ATGTATATAGTATTAAATTGAATTCTTTCTGAGACTTCGTCAGAAACCA-

-------------------------TAAAGCAAGTGTTGGATTCAATGCT
GGTGTTAAAGATTATAAATTGACTTATTACACTCCTGAATATGAAACAAA
GGATACCGATATCTTGGCAGCATTTCAAGTAACTCCTCAACCCGGAGTTC
CACCTGAAGAAGCAGGGGCCGCAGTAGCTGCTGAATCTTCTACTGGTACA
TGGACAACTGTATGGACTGACGGGCTTACCAGTCTTGATCGTTACAAAGG
TCGATGCTACCACATCGAGCCCGTTGCTGGAGAAAAAACTCAATTTATTG
CTTATGTAGCTTACCCCTTAGACCTTTTTAAACAAGGTTCTGTTACTAAC
ATGTTTACTTCCATTGTGGGTAATGTATTTGGGTTCAAGGCCCTGCGCGC
TCTACGTCTGGAGGATTTGCGAATCCCCCCTGCTTATACTAAAACTTTCC
AAGGCCCGCCTCATGGCATCCAAGTTGAGAGAGATAAATTTAACAAGTAT
GGCCGCCCCCTATTGGGATGTACTATTAAACCGAAATTGGGGTTATCCGC
TAAGAATTACGGTAGAGCCGTTTATGAATGTCTTCGCGGTGGACTTGATT
TTACCAAAGATGATGAGAACTTGAATTCCCAGCCGTTTATGCGTTGGAGA
GACCGGTTCTTATTCTGTGCTGAAGCACTTTATAAAGCACAGGCCGAAAC
TGGTGAAATCAAAGGGCATTACTTGAATGCTACGGCAGGTACATGCGAAG
ACATGATGAAAAGGGCTGTATTTGCCAGAGAACTGGGAGTTCCTATCGTA
ATGCACGATTACTTAACGGGGGGATTCACTGCAAATACTACTTTGGCTCA
TTATTGCCGAGATAATGGTCTGCTTCTTCACATCCACCGTGCAATGCATG
CCGTTATTGATAGACAGAAAAATCATGGTATGCACTTTCGTGTACTAGCT
AAAGCGTTACGTATGTCTGGTGGAGATCATATTCACGCTGGTACTGTAGT
GGGTAAACTTGAAGGGGAAAGAGACATCACTTTAGGCTTTGTTGATTTAC
TACGTGATGATTTTATTGATAAAGATCGAAGCCGTGGTATTTATTTCACT
CAAGATTGGGTCTCTCTACCAGGTGTTTTGCCCGTGGCTTCAGGGGGTAT
TCATGTTTGGCATATGCCTGCTCTGACCGAGATCTTTGGAGACGATTCCG
TACTACAATTCGGCGGAGGAACTTTAGGACACCCTTGGGGAAATGCACCC
GGTGCCGTAGCTAATCGAGTAGCTCTAGAAGCATGTGTACAAGCTCCTAA
TGAGGGACGTGATCTTGCTCGTGAAGGTAATGAAATTATCCGTGAGGCTA
GTAAATGGAGTGCTGAACTAGCTGCTGCTTGTGAAGTATGGAAGGAGATT
AAATTTGAATTCCCAGCAATGGATACTTTGTAA-----------------
----TAATTGGATTGAGCTTTGGTATTGGAAACTTACCGGGTGATTACTT
TCAAATTCAGAGAAACCCTGGAATTAAAAACGGGCAATCCTGAGCCAAAT
CCTGTTTTCTGAAAACAAACAAAGGTTCAGAAAGCGATAATAAAAAA--G
GATAGATTAGGGATAGGTGCAGAGACTCAATGGAAGTTGTTCTAACAAAT
GG----AGTTGGCCACGATGCGTT------AGTAAAGGACT-CCTTCCAT
CGAAACTCCAGAAAGTATGAAGAATAAACGTATATATACGTACT------
GAAATACTATCTCCAAACCAAATGATTAATGACGACCCGAATCTTTTTTT
TTT--TATTTATATAAAA-------------------------------A
ATGAAAGAATTGTTGT----------GAATCGATTCCAATTTG-AAAACA
GAATCGAATATTCATTGATCAAATCATTTACTCCATCGTAATCTGAT---
-AAATCTTTTGAAGAATTGAT-----T-ACTCGGACGAGAATAAAGATAG
AGTCCCGTTCTACATGTCAA------------TATCGACAACAATGCAAT
TTATTGTAAAAGGAGGATCCGTCGACTTTA-GAAATCGTGAGGGTTCAAG
TCCCTCTATCCCCAAAAAGTCCCTATTGGATTCCCTAATTCTTTATC---
-------CTATGCTCTCAGTTCGTCAACGGTTCAAAATTCGTTATGTTTC
TCATTCATTCTACTCTTTTACTTTACAAATGGTCTGAGCGGAAATTTGTT
TCT--------TTTCACAAGCCTTG-----TGATATTGTGATATTGATAT
ATAGGATACACGTACAAATGAACATCGTTGGGCACGT----AACCCCGAT
TGTAAATTGT-------AATGATT-AACAATACATATTATTACTTGTACT
GTACTGAAACTTACA----AAGTC------TTCTTTTTG-AAGATCCAAG
AAATT----CCACCAAGACCCGGATAAGGTTTTGTAATC----CCCTTTT
CGTCTTTTTCATTGACATAGAACCAAGTCCTCTATTAATATTAAAATGAG
GATGGTGCGTCGTG-----AATGGTCGGGATAGCTCAGCTGGTAGAGGAG
AGGACTGAATA---------------------------------------

---G
GATCATTGTTGAAACCCT-GCACAGCAGAACGACCCGTGAACTTGTAAAA
CATAATCGGGGGGTGAGGGGC---CAACCTGAAC----C-C----CCCTT
-GGCTGG--GGGGCGC---CAACTCAGGTGTCGCTGGCCGATGCCTGTTG
TTGTTGCCTTCCCG-GCCA-CACAAACGAACCCCGGCGCAAACCGCGCCA
--AGGAAATCAAACGAATCAAG-CATTGCCCCGGCTT-------------
-----TCGGGGTGCTGCG--TCGTAT--TCTGTATGTCAAAACGACTCTC
GGCAACGGATATCTCGGCTCTCGCATCGATGAAGAACGTAGCGAAATGCG
ATACTTGGTGTGAATTGCAGAATCCCGTGAACCATCGAGTCTTTGAACGC
AAGTTGCGCCCGAAGCCA--TTAGGCCGAGGGCACGTCTGCCTGGGCGTC
ACACAACGTTGCCCCCCCAACCCCAAACCTCGTCTCCGAG--G-------
-----------AGGGCG-GGGGGCGGATGCTGGCTTCCCGTGTGCCTTGT
CTCG--CGGTTGGCCTAAAT--GCGAGCACTCGGCGACGAACGCCGTGGC
AATCGGTGGTTGTCCAACCCTCG-GTGCCATGCTGCGCGCGTGAGTCGCT
GATGCGTGCTCATCGCCC---CCAA-TGCGCCG-----------------

>Dallachya_vitiensis

----------------------CCCAACCCGCTAGAATATTCATATATAC
AATGAT---AATATATATCAGAAAAAGATTGCCCTTTTAAAATTAAATAA
AATTAA----------------ACAT-------TCTTAAGAGTATAATAT
TGGAAAAATTGGATTATTATTATTAATACTAT-------------AATAT
AATTCTTTTCTGAATCATATCAATTTCACAAATTGAATCGTATTCAAATA
ACACATAGAGGGAATTGAATCTATTTGTCATCCCTAAATGTTAAA-----
-TATTTAACATAGAATATCTAGAATTCATTTCAGTAACAATTGTTTAGTC
TATCTGATAAATAT----GAGG-------GTCCCATATTATTGTTTTTGG
GATTCTGGTTTTAT--------CAAACAGTATGAAAAAATAACAACCCTC
CCTTACATCAGTCTTTATCCACTATTTCACTAAAAAAAGAAATAATTTTT
TTTTTTATTATATGTATATGTTTTTTAT--------------------T-
-T---------CTATAACATTAAAAAA-----------------------
----------------------------AAAG----A---AAAATAGTA-
--------------------ACTTAATCTTAATAAATAAAATGGAATTGA

AATATTCC--------ATTTTAAGATTAATA-------------------

------------------------------A---------------ATAT
ATGTGTATGGTATTTAATTGAATTCTTTCTGAGACTTCGTCAGAAACCAG
CCATTTATATTTCATATAGAAATAAAAG-------------------GTA
TAGATTATTATGTACCAGCCGAACCAATGACTATTCGTGATTCCATAATT
GAATCAATTACATACTGGTTCCAA--------------------------

------------CAAAAAGTC-CCATCGGATTCCCTAATTATTTATC---
-------CTATGCTCTCATTTCGTTAACGGTTCAAAATTCGTTATGTTTC
TCATTCATTCTACTCTTTTACTTTACAAATGGTCTGAGCGGAAATTTTTT
TCT--------TTTC-------------------ACAAGCCTTGTGATAT
ATAGGATACACGTACAAATGAACATCGTTGTGCACGT----AACCCCGAT
TGTAAATTGT-------AATGATT-AACAATACATATTATTACTTGTACT
GTACTGAAACTTACA----AAGTC------TTCTTTTTG-AAGATCCAAG
AAATT----CCACCAAGACCTGGATAAGGCTTTGTAATC----CCCTTTT
CGTCTTTTTCATTGACATAGAACCAAGTCCTCTATTA------AAATGAG
GATGGTGCGTCGTG-----AATGGTCGGGATAGCTCAGCTGGTAGAGCAG

AGGACTGAAAATC-------------------------------------

------TTGTTGAACCCT-GCACAGCAGAACGACCCGTGAACTCGTAAAA
TACAATCGGGGGGCGAGGGGC---CA-AC-CCGA--ACC-C----CCCTC
GGCGGGT--GGGCATG---CGACTCGGGCTTCGCTGGCCGATGCCCGCCG
CCGCTGCCTTCCCCGCCGC-ACAAACGAAACCCCGGCGCAAACCGCGCCA
--AGGAACTCGAACGGATGAAG-CATTGCCCCGGCGCCCCAGAGATGGTG
CGCGCCGGGGGCTCTGCG--TCGTATTCTGTATATGTCAAAACGACTCTC
GGCAACGGATATCTCGGCTCTCGCATCGATGAAGAACGTAGCGAAATGCG
ATACTTGGTGTGAATTGCAGAATCCCGTGAACCATCGAGTCTTTGAACGC
AAGTTGCGCCCGAAGCCA--TCAGGCCGAGGGCACGTCTGCCTGGGCGTC
ACACAACGTTGCCCCCCCA-CCCCAAGCCTCGTCCTCGAG--G-------
-----------AGGGCGGGGGGGCGGATGCTGGCCTCCCGTGCGCCCCGG
TTCG--CGGCTGGCCCAAAT--GCGAGCACTCGGCGACGAGCGCCGCGGC
AATCGGTGGTTGTCCAACCCTCG-GTGTCACGCCGCGCGCGCGAGCCGCC
CGTGTGTGCCCGTCGACC---CTCA-ATGCGCC---GCACACGCGGCGTT
TACGATGCGACCCCAGGTCAGGCGGGGCTACCCGCTGAGTTTAA------

>Doerpfeldia_cubensis

---------------------------------------GATTCAAAGCT
GGTGTTAAAGATTATAAATTGACTTATTACACTCCTGACTATGAAACCAA
AGATACTGATATCTTAGCAGCATTTCGAGTAACTCCTCAACCTGGAGTTC
CTCCTGAGGAAGCAGGGGCCGCGGTAGCTGCTGAATCTTCTACTGGTACA
TGGACAAATGTATGGACTGACGGGCTTACCAGTCTTGATCGTTACAAAGG
CCGATGCTACCACATCGAGCCCGTTGCTGGAGAAGAAAATCAATATATGG
CTTATGTAGCTTACCCCTTAGACCTTTGGAAACAAGGTTCTGTTACTAAC
ATGTTTACTTCCATTGTGGGTAATGTATTTGGGTTCAAAGCTTTGCGCGC
TCTACGTCTGGAGGATTTGCGAATCCCCACTTCTTATACTAAAACTTTCC
AAGGCCCGCCTCATGGACTCCAAGTTGAGAGAGATAAATTTAATAAGTAT
GGCCGCCCCCTAAAGGGATATACTATTAAACCTAAATTGGGGTTATCCGC
TAAGAATTACGGTAGAACAGTTTACGAATGTCTTCGCGGCGGACTTGATT
TTGACCCCGATGATGAGAATGTGAATTCCCAACCATTTATACGTTGGAGA
GACCGTTTCTTATTCTGTGCCGAAGCACTTTATAAAGCACAGGCAGAAAC
AGGTGAAATCAAAGGGCATTACTTGAATGCTACTGCAGGTACATGCGAAG
AAATGATCAAAAGGGCTGTATTTGCCAGAGAATTGGGAGTTCCTATCGTA
ATGCATGATTACTTAACGGGGGGATTCACTGCAAATACTACCCTGGCTCA
TTATTGCCGAGATAATGGCCTACTTCTTCACATCCACCGTGCAATGCATG
CAGTTATTGATAGACAGAAGAATCATGGTATGCACTTCCGCGTACTAGCT
AAAGCGTTACGTTTGTCTGGTGGAGATCATATTCACGCTGGTACTGTAGT
AGGTAAACTTGAAGGGGAAAGAGAAATCACTTTAGGCTTTGTTGATTTAC
TACGTGATGATTATATTGAAAAAGATCGAAGCCGTGGTATTTATTTCACT
CAAGATTGGGTTTCTCTACCAGGTGTTATACCCGTGGCTTCAGGGGGTAT
TCACGTTTGGCATATGCCCGCTCTGACCGAGATCTTTGGAGACGATTCCG
TACTACAATTCGGCGGAGGAACTTTAGGGCACCCTTGGGGAAATGCACCC
GGTGCTGTAGCTAATCGAGTAGCTCTAGAAGCATGTGTACAAGCTCGTAA
TGAGGGACGCGATCTTGCTCGTGAGGGTAATGAAATTATCCGTGAGGCTA
GTAAATGGAGTCCTGAACTAGCTGCCGCTTGTGAAGTATGGAAGGAGATC
AAATTTGAATTCCAAGCAATGGATACTTTGTAA-----------------
----TTAATTGGATTGAGCCTTGGTATGGAAACCTACCAAGTGATAACTT
TCAAATTCAGAGAAACCCTAGAATTAAAAATGGGCAATCCTGAGCCAAAT
CCCGTTTTCTGAAAACAAACGAAGGTTCGGAGATCGATA-ATA---AAAA
AGGAAAGATAGGATAGGTGCAGAGACTCAATGGAAGCTGTTCTAACAAAG
GG----AGTTACCTACGATGCGTTAGTA--AAGAAATAAAATCCTTACAT
CGAAACGCCAGAAAGGACGAAGAATAAACCTATATATACGTATACATATT
GCAATACTATCTTCAAACCAAATGATTAATGAAGACCCCAATCTTT-TTT
TATTTATATGTATAT---GAAAAATGA-----------------------
----AAGAATTAT-----------TGTGAATCGATTCCAGGTTGAAAAAA
GAATCGAATATTCATTGATCAAATTATTTACTCCGTCGTAATCTGAT---
-AGATCTTTTGAAAAAATTGAT------TAATCGGACGAGAATAAAGATAG
AGTCCCATTCTACATGTCAA------------TATCGACAACAATGAAAT
TTATAGTAAGAGGAAAATCCGTCGACTTTA-GAAATCGTGAGGGTTCAAG
TCCCTCTATCCCCAAAAAGGC-CCATTGTATTCCCTAATTATTTATC---
--CTA-----TACTCTCATTTTGTTAGCGGTTCAAAATTCGTTATGGTTC
GCATTCATTCTACTCTTTTACAAATGTATCTAATCGT-------------
----AAATTCTTTTCACAAGCCTTTTTTTTGATATATATGATACACGTAC
--------------AAATGAACATCGTTGACCACTC----CATTGGAAT
GATTAACAAT-------ACATATTATTACTACATAATTATTACTCGGGCT
GTGCTGAAT----CTTAAAAAGTCTT-------CTTTTTGAAGATCCAAG
AAATTCCACCAAAGCCTGACTGGATAAGACTTTGTAAT----TCCCTTTT

TGTATTTTTAATTGACGAAGACCCAAGCCATCTATGAAAATTACAATCAG
GATGATGCGCCGTG-----AATGGTCGGGATAGCTCAGCTGGTAGAGCAG
AGAC--

>Ampelozizyphus_amazonicus

----------------------------AGCGAGTGTTGGATTCAAAGCT
GGTGTTAAAGATTATAAATTGACTTATTACACTCCTGACTATGAAACCAA
AGATACTGATATCTTAGCAGCATTTCGAGTAACTCCTCAACCTGGAGTTC
CTCCTGAGGAAGCAGGGGCCGCGGTAGCTGCTGAATCTTCTACTGGTACA
TGGACAACTGTATGGACTGACGGGCTTACCAGTCTTGATCGTTACAAAGG
TCGATGCTACCACATTGAGCCCGTTGCTGGAGAAGAAAATCAATATATTG
TTTATGTAGCTTACCCCTTAGACCTTTTTGAAGAAGGTTCTGTTACTAAC
ATGTTTACTTCCATTGTGGGTAATGTATTTGGGTTCAAAGCCTTGCGCGC
TCTACGCCTGGAGGATTTGCGAATCCCTCCTGCTTATTCTAAAACTTTCC
AAGGCCCACCTCATGGAATCCAAGTTGAGAGAGATAAATTGAACAAGTAC
GGCCGCCCCCTATTGGGATGTACTATTAAACCTAAATTGGGGTTATCCGC
TAAGAATTACGGTAGAGCAGTTTATGAATGTCTTCGCGGCGGACTTGATT
TTACCAAAGATGATGAGAATGTGAATTCCCAACCATTTATGCGTTGGAGA
GACCGTTTCTTATTTTGTGCNGAAGCACTTTATAAAGCACAGGCAGAAAC
AGGTGAAATCAAAGGGCATTACTTGAATGCTACTGCAGGTACATGCGAAG
AAATGATCAAAAGGGCTGTATTTGCCAGAGAATTGGGAGTTCCTATCGTA
ATGCATGATTACTTAACGGGGGGATTCACTGCAAATACTAGCTTGGCTCA
TTATTGCCGAGATAATGGCCTACTTCTTCACATCCACCGTGCAATGCATG
CAGTTATTGATAGACAGAAGAATCATGGTATGCACTTTCGCGTACTAGCT
AAAGCGTTACGTTTGTCTGGTGGAGATCATATTCACGCTGGTACTGTAGT
AGGTAAACTTGAAGGGGAAAGAGAAATCACTTTAGGCTTTGTTGATTTAC
TACGTGATGATTATGTTGAAAAAGATCGAAGCCGTGGTATTTATTTCACT
CAAGATTGGGTTTCTCTACCAGGTGTTATACCTGTGGCTTCAGGGGGTAT
TCACGTTTGGCATATGCCCGCTCTGACCGAGATCTTTGGAGACGATTCCG
TACTACAATTCGGCGGAGGAACTTTAGGGCACCCTTGGGGAAATGCACCC
GGTGCTGTAGCTAATCGAGTAGCTCTAGAAGCATGTGTACAAGCTCGTAA
TGAGGGACGCGATCTTGCTCGTGAGGGTAATGAAATTATCCGTGAGGCTA
GTAAATGGAGTCCTGAACTAGCTGCCGCTTGTGAAGTATGGAAGGAGATC
AAATTTGAATTCGAAGCAATGGATACCTTGTAA-----------------
-----------TTGGATGAGCTTGGTATGGAACCTACCAAGTGATAACTT
TCAAATTCAGAGAAACCCTAGAATTAAAAATGGGCAATCCTGAGCCAAAT
CCCGTTTTCTGAAAACAAACGAAGGTTCGGAAAGCGATA-ATA---AAAA
AGGACAGATAGGATAGGTGCAGAGACTCAATGGAAGCTGTTCTAACAAAG
GG----AGTTACCTACGATGCGTTAGTA--AAGTAAAGAAATCCTTCCAT
CGAAACGCCAGAAAGGACGAAGAATAAACCTATATATACGTATACATAGT
GAAATACTATCTTCAAACCAAATGATTAATGAAGACCCCAATCTTTTTTT
TATTTATATGTATAT---GAAAAATGA-----------------------
----AAGAATTGT-----------TGTGAATCAATTCCAGGCTGAAAAAA
GAATCGAATATTCATTGATCAAATTATTTACTCCGTCGTAATCTGAT---
-AGATCTTTTGAAAAATTGAT------TAATCGGACGAGAATAAAGATAG
AGTCCCATTCTACATGTCAA------------TATCGACAACAATGAAAT
TTATAGTAAGAGGAAAATCCGTCGACTTTA-GAAATCGTGAGGGTTCAAG
TCCCTCTATCCCCAAAAAGGC-CCATTGGATTCCCTAATTATTTATC---
--CTA-----TACTCTCATTTTGTTAGCGGTTCAAAATTCGTTATGGTTC
GCATTCATTCTACTCTTTTACAAATGAATCTAATCGT-------------
----AAATTCTTTTCACAAGCCTTTTGA----TATATATGATACACGTAC
---------------AAATGAACATCGTTGAGCAAAT----AACTCCGAT
TGTAAATTGG-------AATGATTA-ACAATACATATTATTACTCGGACT

GTACTGAAT----CTTACAAAGTCTT-------CTTTTTGAAGATCCAAG
AAATTCCACCAAAGCCTGACTGGATAAGACTTTGTAAT----TCCCTTTT
TGTCTTTTTAATTGACGAAGACCCAAGTCATCTATTAAAATTACAATCAG
GATGATGCGTCGTG-----AATGGTCGGGATAGCTCAGCTGTAGAGCAGA
GACTGA--

>Bathiorhamnus_cryptophorous

----------------------------AGCGAGTGTTGGACTCATAGCT
GGTGTTAAAGATTATAAATTGACTTATTACACTCCTGACTATGAAACCAA
AGTTACTGATATCTTGGCAGCATTTCGAGTAACTCCTCAACCTGGAGTTC
CTCCTGAGGAAGCAGGGGCCGCGGTAGCTGCTGAATCTTCTACTGGTACA
TGGACAACTGTATGGACTGACGGGCTTACCAGCCTTGATCGTTACAAAGG
TCGATGCTACCACATCGAGCCCGTTGCTGGAGAAGAAAGTCAATTTATTG
CTTATGTAGCTTACCCCTTAGACCTTTTTGAAGAAGGTTCTGTTACTAAC
ATGTTTACTTCCATTGTGGGTAATGTATTTGGGTTCAAAGCCTTGCGCGC
TCTACGTCTGGAGGATTTGCGAATCCCTCCTGCTTATACTAAAACTTTCC
AAGGCCCGCCTCATGGCATCCAAGTTGAGAGAGATAAATTGAACAAGTAC
GGCCGCCCCCTGTTGGGATGTACTATTAAACCTAAATTGGGGTTATCCGC
TAAGAATTACGGTAGAGCAGTTTATGAATGTCTTCGCGGCGGACTTGATT
TTACCAAAGATGATGAGAATGTGAATTCCCAACCATTTATGCGTTGGAGA
GACCGTTTCTTATTTTGTGCTGAAGCACTTTATAAAGCACAGGCAGAAAC
AGGTGAAATCAAAGGGCATTACTTGAATGCTACTGCAGGTACATGCGAAG
AAATGATCAAAAGGGCTGTATTTGCCAGAGAATTGGGAGTTCCTATCGTA
ATGCATGATTACTTAACAGGGGGATTCACTGCAAATACTAGCTTGGCTCA
TTATTGCCGAGATAATGGCCTACTTCTTCACATCCACCGTGCAATGCATG
CAGTTATTGATAGACAGAAGAATCATGGTATGCACTTTCGCGTACTAGCT
AAAGCGTTACGTCTGTCTGGTGGAGATCATATTCACGCTGGTACTGTAGT
AGGTAAACTTGAAGGGGAAAGAGAAATCACTTTAGGCTTTGTTGATTTAC
TACGCGATGATTTTGTTGAAAAAGATCGAAGCCGTGGTATTTATTTCACT
CAAGATTGGGTTTCTATACCAGGTGTTCTGCCCGTGGCTTCAGGGGGTAT
TCACGTTTGGCATATGCCCGCTCTGACCGAGATCTTTGGAGACGATTCCG
TACTACAATTCGGCGGAGGAACTTTAGGGCACCCTTGGGGAAATGCACCC
GGTGCTGTAGCTAATCGAGTAGCTCTAGAAGCATGTGTACAAGCTCGTAA
TGAGGGACGTGATCTTGCTCGTGAGGGTAATGAAATTATCCGTGAGGCTA
GTAAATGGAGTCCTGAACTACCTGCCGCTTGTGAATTATGGAAGGAGATC
AAATTTGAATTCACAGCATACTTGTATCAGTAA-----------------
-------TTAATTGGGCAGCTTGGTATGGAAACCTACCGGCTGATAACTT
TCAAATTCAGAGAAACCCTAGAATTAAAAATGGGCAATCCTGAGCCAAAT
CCTGTTTTCTGAAAACAAACGAAGGTTCAGAAAGCGATA-ATA---AAAA
AGGACAGATAGGATAGGTGCAGAGACTCAATGGAAGCTGTTCTAACATAG
GG----AGTTACCTACTATGCGTTAGTA--AAGGAAT-----ACTTACAT
CGAAACGCCAGAAAGGATGAAGAATAAACCTATATAT------ACGTATT
GAAATACTATCTTCAAACCAAATGATTAATGACGACCCAAATCTTTTTTT
TTTTTTTTTATATGTTTATAGAAAATG-----------------------
----AAGAATTGT-----------TGTGAATCGATTCCAAGTTGAAAAAA
GAATCGAATATTCATTGATCAAATTATTTACTCCGTCGTAATCTGAT---
-CGATCTTG-GAAAAATTGAT------TAATCGGACGAGAATAAAGATAG
AGTCCCATTCTACATGTCAA------------TATCGACAACAATGAAAT
TTATAGTAAGAGGAAAATCCGTCGACTTTA-GAAATCGTGAGGGTTCAAG
TCCCTCTATCCCCAAAAAGGC-CCATTTGATTCCCTAATTATTTATC---
--CTA-----TACTCTCATTTCGTTAGCGGTTCAAAATTCGTTATGGTTC
GCATTCATTCTACTCTTTTACAAATGGATCTGAGCGT-------------
----AAATTCTTTTCACAAGCCTTTTGA----TATATACGATACACGTAC

---------------AAATGAACATCGTTGAGCAAGT----AATTCCGAT
TGTAAATTGG-------AATGATTA-ACAATACATATTATTACTCGGACT
GTACTAAAT----CTTACAAAGTCTT-------CTTTTTGAAGATCCAAG
AAATTCCACCAAAGCCCGGCCGGATAAGACTTTATAAT----TCCCTTTT
CATCTTTTTAATTGACGGAGACCCAAGTCATCTATGAAAATTAAAATCAG
GATAATGCGTCGTG-----AATGGTCGGGATAGCTCAGCTGGTAGAGCGA
GAGGACTGAATA--------------------------------------

>Crumenaria_erecta

-------------------------TAAAGCAAGTGTTGGATTCAAAGCC
GGTGTTAAAGATTATAAATTGACTTATTACACTCCTGAATATGAAACCAA
AGATACTGATATCTTGGCAGCGTTTCGAGTAACTCCTCAACCCGGAGTTC
CGCCTGAAGAAGCGGGGGCCGCGGTAGCTGCTGAATCTTCTACTGGTACA
TGGACAACTGTATGGACTGACGGGCTTACCAGTCTTGATCGTTACAAAGG
TCGATGCTACCACCTTGAGCCCGTTGCTGGAGAAGAAAATCAATATATTG
CTTATGTAGCTTACCCTTTAGACCTTTTTGAAGAAGGTTCTGTTACTAAC
ATGTTTACTTCCATTGTGGGTAACGTTTTTGGGTTCAAGGCCCTGCGCGC
TCTACGTCTGGAGGATTTGCGAATTCCTCCTGCGTATACTAAAACTTTCC
AAGGACCACCTCATGGCATCCAAGTTGAAAGAGATAAATTGAACAAGTAC
GGCCGCCCACTATTGGGATGTACTATTAAACCTAAATTGGGGTTATCTGC
TAAGAATTACGGTAGAGCAGTTTATGAATGTCTTCGCGGTGGACTTGATT
TTACCAAAGATGATGAGAACGTGAATTCCCAACCATTTATGCGTTGGAGA
GACCGTTTCTTATTTTGTGCCGAAGCAATTTATAAAGCACAGGCTGAAAC
AGGTGAAATCAAAGGGCATTACTTGAATGCTACTGCMGGTACATGCGAAG
AAATGATTAAAAGGGCTGTATTTGCCAGAGAATTGGGAGCTCCTATTGTA
ATGCACGATTACTTAACCGGGGGATTCACTGCAAATACTAGCTTGGCTCA
TTATTGCCGAGATAATGGTCTACTTCTTCACATCCACCGTGCAATGCATG
CGGTTATTGATAGACAGAAAAATCATGGTATGCACTTTCGTGTACTAGCT
AAAGCATTACGTATGTCTGGTGGAGATCATATTCACGCTGGTACTGTAGT
AGGTAAACTTGAAGGGGAAAGGGACATCACTTTAGGCTTTGTTGATTTAC
TACGTGATGATTTTGTTGAAAAAGATCGAAGCCGCGGTATTTATTTCACT
CAAGATTGGGTCTCTCTGCCAGGTGTTCTGCCTGTGGCTTCAGGGGGTAT
TCATGTTTGGCATATGCCTGCTCTGACTGAGATCTTTGGAGATGATTCCG
TACTACAGTTCGGCGGAGGAACTTTAGGACACCCTTGGGGAAATGCACCA
GGTGCCGTAGCTAATCGAGTAGCTCTAGAAGCATGTGTACAAGCCGGTAA
TGAGGGACGTGATCTCGCTTCTGAGGGTAATGATATTATTCCTGAGGCTA
GTAAATGGAGTCCTGAACTAGCTGCTCCTTGTGAAGTATGGAAGGAGATC
AAATTTGAATTC--------------------------------------
------TTAATTGATGGGGCTTGGTATGGAAACCTACCGAGTGATAACTT
TCAAATTCAGAGAAACCCTGGAATTAGAAATGGGCAATCCTGAGCCAAAT
CCTGTTTTCTGAAAACAAACAAGGATTCAGAAAGTGATAAT------AAA
AAAAGGGGGGGGATAGGTGCAGAGACTCAACGGAAGCTGTTCTAACAAAT
GG----AGTTGGCTGCGGCGCGTT------AGTAAAGGAAT-CCTTCTAT
CGAAACTCCAGAAAGGGTGAAGAATAAACCTATATCTACGTATACGTACT
AAAATAGTATCTCCAAACCAAATGATTAATGACGACCCGAATCTTTTTTT
TTTTTTATTTATATGGTTAGATGAAA-----------------------A
ACGAAAGAATTGTTGTGAATC-----GATTCCAAGTCAAAGAAAATGGAA
TATTCATTGATCAAATCATT--------TACTCCATCGTAATCTGAT---
-AGATCTT-TTGAAAAATT-----GACTAATCGGACGAGAATAAAGATAG
AGTCCCATTCTACATGTCAA------------TATCGGCAACAATGAAAT
TTATAGTAAGAGGAAAATCCGTCGACTTTA-TAAATCGTGAGGGTTCAAG
TCCCTCTATCCCCAACCAAAA-AGGCCCATTTGATTCCCTAATTTT----
--TTATCCTATACTCTCATTTGGTTAGCGGTTCAAACTCTGTTATGTTTC

TCATTCATTAATTTTTCACAAGCC--------------------------
----------------------TTG-----TATATATATGATACACGTAC
---------------AAACGAACATCATTGAGCAAGT----AACTCCGAT
TGGAAA------TTATTGTAAATTGGAATGATCATATTATCACTCGTACT
GTACTGAAACTCACAAAGTCTTCT--------TTTTTTTGAAGATCTAAG
AAATT----CCACCAAGGCCTGGATAAGACTTTGTAAT----CCCCTTTT
CGTCTTTTTAATTGACAAAGACCCACGTCATCTATTAA------AATGGG
GGTGATGCGTCGTC-----AATGGTCGGGATAGCTCAGCTGGTAAAGCAG
AGACTGAA--

--------TCGATGCC-T-GCACAGCAGAACGACCAGCGAACTGGTGAAC
ACACACGCGGGAGAGGACGTGGGTTTGC------CCACG-CG-CTCTCCT
GGCTCAG--GGGGCAG---CACCCGCCGCATCGTCCGTC-------TGGT
GCC-TGCATCCTGGGCCGCTCGAACAAAAAACTCGGCGCAGAACGCGCC-
-AAGGAACTCATAACATGTCCGCGT-CTCCCGGCGGCCCAAGCGATTGGG
TGAG--------ATCGAGAGACGCACTGTGAATGTCTGAAAACGACTCTC
GGCAACGGATATCTCGGCTCTCGCATCGATGAAGAACGTAGCGAAATGCG
ATACTTGGTGTGAATTGCAGAATCCCGTGAACCATCGAGTCTTTGAACGC
AAGTTGCGCCCGAAGCCA--TTAGGCCGAGGGCACGTCTGCCTGGGCGTC
ACACAACGTTGCCCCCCACAACCTCTCGGTT--CAAGGGGCGG-------
--------------------------ACGCTGGCCTCCCGTCTCCCC---
---G--CGGTTGGCCTAAATG---CAGTCTGCGGCGGTCAGTGCCGGAGC
AATCGGTGGTGTTGCGAAACCCAGCGTCCCATGCTCCTGCACGGCCCGCC
GRCCGGACGGTCAKTTCC----------C-------CCAAYGCCGCGCCT
AAAGCGGCCTCCACAG----------------------------------

>Reissekia_smilacina

----------------------------AGCAAGTGTTGGATTCAAAGCC
GGTGTTAAAGATTATAAATTGACTTATTACACTCCTGAATATGAAACCAA
AGATACTGATATCTTGGCAGCGTTTCGAGTAACTCCTCAACCCGGAGTTC
CGCCTGAAGAAGCAGGGGCCGCGGTAGCTGCTGAATCTTCTACTGGTACA
TGGACAACTGTATGGACTGACGGGCTTACCAGTCTTGATCGTTACAAAGG
TCGATGCTACCACCTTGAGCCCGTTGCTGGAGAAGAAAATCAATATATTG
CTTATGTAGCTTACCCTTTAGACCTTTTTGAAGAAGGTTCTGTTACTAAC
ATGTTTACTTCCATTGTGGGTAACGTTTTTGGGTTCAAGGCCCTGCGCGC
TCTACGTCTGGAGGATTTGCGAATCCCCCCTGCGTATACTAAAACTTTCC
AAGGACCGCCTCATGGCATCCAAGTTGAAAGAGATAAATTGAACAAGTAT
GGCCGCCCGCTATTGGGATGTACTATTAAACCTAAATTGGGGTTATCTGC
TAAGAATTACGGTAGCTGTGTTTATGAATGTCTTCGCGGTGGACTTGATT
TTACCACAGATGATGAGAACGTGAATTCCCAACCATTTATGCGTTGGAGA
GACCGTTTCTTATTTTGTGCCGAAGCCCTTTTTAAAGCACAGGCTGAAAC
AGGTGAAATCAAAGGGCATTACTTGAATGCTACTGCAGGTACATGCGAAG
AAATGATGAAAAGGGCTATATTTGCCAGAGAATTGGGAGCTCCTATTGTA
ATGCACGATTACTTAACTGGGGGATTCACTGCAAATACTAGCTTGGCTCA
TTATTGCCGAGATAATGGTCTACTTCTTCACATCCACCGTGCAATGCATG
CGGTTATTGATAGACAGAAAAATCATGGTATGCACTTTCGTGTACTAGCT
AAAGGATTACGTATGTCTGGTGGAGATCATATTCACGCCGGTACTGTAGT
AGGTAAACTTGAAGGGGAAAGGGACATCACTTTAGGCTTTGTTGATTTAC
TACGTGATGATTTTGTTGAAAAAGATCGAAGCCGTGGTATTTATTTCACT
CAAGATTGGGTCTCCCTGCCAGGTGTTCTGCCTGTGGCTTCAGGGGGTAT
TCATGTTTGGCATATGCCCGCTCTGACCGAGATCTTTGGAGATGATTCCG
TACTACAGTTCGGCGGAGGAACTTTAGGACACCCTTGGGGAAATGCACCC
GGTGCCGTAGCTAATCGAGTAGCTCTAGAAGCATGCGTACAAGCTCGTAA
TGAGGGACGTGATCTTGCTCGTGAAGGTAATGAAATTATCCGTGAGGCTA
GTAAATGGAGTTCTGAACTAGCTGCTGCTTGTGAAGTATGGAAGGAGATC
AAATTTGAATTCGAAGCAATGGATACTTTGTAA-----------------
---TTAATTGAGTTCGAGCCTAGGTATGGAAACCTATCGAGTGATAACTT
TCAAATTCAGAGAAACCCTGGAATTACAAATGGGCCATCCTGAGCCAAAT
CCTGTTTTCTGAAAACAAACAAGGATTCAGAAAGTGATAATGGTAATAAA
AAAAAAAAGGGGATGGGTGCAAAAACTCAACGGAAGCTGTTCTAACAAAT
GG----AGTTGGCCGCGGCCCGTT------AGTAAAGGAAT-CCTTCTAT
CGAAACCCCAAAAAGGATGAAAAATAAACCTATTTCAACGTATACGTACT
AAAATACTGTCTCCAAACCCAATGATTAATGACGACCCGAATCTTTTTTT
AATATT----TATAGGTCAAATGAAA-----------------------A
ATGAAAGAATTGTTGTGAATC-----GATTCCAAGTCAAAGAAAATGGAA
TATTCATTGATCAAATCATTACTCCATTTACTCCATCGTAATCTGAT---
-AGATCTTTTGGAAAAATT-----GACTAATCGGACGAGAATAAAGATAG
AGTCCCATTCTACATGTCAA------------TATCGGCAACAATGAAAT
TTATAGTAAGAGGAAAATCCGTCGACTTTA-TAAATCGTGAGGGTTCAAG

TCCCTCTATCCCCAACCAAAA-AGGCCCATTTGATTCCCTAATTTT----
--TTATCCTATACTCTCATTTCGTTAGCGGTTCAAACTCTGTTATGTTTC
TCATTCATTAATTTTTCACAAGCC--------------------------
----------------------TTG-----TATATATATGATACACGTAC
---------------AAATGAACATCATTGAGCAAGT----AACTCCGAT
TGTAAA------TTATTGTAAATTGGAATGATCATATTATCACTCGTACT
GTACTGAAACTCACAAAGTCTTCT--------TTTTTTTGAAGATCTAAG
AAATT----CCACCAAGGCCCGGATAAGACTTTGTAAT----CCCTTTAT
CGTCTTTGTATTTGACATAGACCCACGTCATATATTTA------AATGAG
GCTGATGCGCCGCA-----AATGGTCGGGATAGCTCAGCTGGTAGAGCAG
CGACTGAAA---

-ATCATTGTCGATACC-T-GCACAGCASAC-GACCCGTGAACCGTTGACA
CACATGTCGGGAGAGAACGTTGGTTAGC------CTT-C-GG-CCTCTCT
TGGTCTG--GGGGCTG---CATCCGTCGCATCGTTCGTC-------GGGT
GCC-GGCATCCCCGGACGCACAWAACTAAATTCCGGCGCAKAACGCGCC-
-AAGGAACTCTTAACATGTTCGCAT-CTCCCGGTCGCCCCAGCAATGGAG
CGCG--------CTCGGGGGTTGCACTGTGAATGTCTGA-AACGACTCTC
GGCAACGGATATCTCGGCTCTCGCATCGATGAAGAACGTANCGAAATGCG
ATACTTGGTGTGAATTGCAGAATCCCGTGAACCATCGAGTCTTTGAACGC
AAGTTGCGCCCGAAGCC---------------------------------

>Pleuranthodes_hillebrandii

-------------------------TAAAGCAAGTGTTGGATTCAAAGCT
GGTGTTAAAGATATCATATTGACTTATTACACTCCTGAATATGAAACCAA
AGATACTGATATCTTGGCAGCGTTTCGAGTAACTCCTCAACCCGGAGTTC
CGCCTGAAGAAGCAGGGGCCGCGGTAGCTGCTGAATCTTCTACTGGTACA
TGGACAACTGTATGGACTGACGGGCTTACCAGTCTTGATCGTTACAAAGG
TCGATGCTACCACCTCGAGCCCGTTGCTGGAGAAGAAAATCAATATATTG
CTTATGTAGCTTACCCNTTAGACCTTTTTGAAGAAGGTTCTGTTACTAAC
ATGTTTACTTCCATTGTGGGTAACGTATTTGGGTTCAAGGCCCTGCGCGC
TCTACGTCTGGAAGATTTGCGAATCCCTCCTGCGTATACTAAAACTTTCC
AAGGACCGCCTCATGGCATCCAAGTTGAAAGAGATAAATTGAACAAGTAT
GGCCGTCCCCTATTGGGATGTACTATTAAACCTAAATTGGGGTTATCTGC
TAAGAATTACGGTAGAGCTGTTTATGAATGTCTTCGCGGTGGACTTGATT
TTACCAAAGATGATGAGAACGTGAATTCCCAACCATTTATGCGTTGGAGA
GACCGTTTCTTATTTTGTGCCGAAGCCCTTTATAAAGCACAGGCTGAAAC
AGGTGAAATCAAAGGGCATTACTTGAATGCTACTGCAGGTACATGCGAAG
AAATGATCAAAAGGGCTGTATTTGCCAGAGAATTGGGAGTTCCTATCGTA
ATGCACGATTACTTAACGGGGGGATTCACTGCAAATACTAGCTTGGCTCA
TTATTGCCGAGATAATGGTCTACTTCTTCACATCCACCGTGCAATGCATG
CGGTTATTGATAGACAGAAAAATCATGGTATGCACTTTCGTGTACTAGCA
AAAGCGATACGTATGTCTGGTGGAGATCATATTCACGCTGGTACTGTAGT
AGGTAAACTTGAAGGGGAAAGAGACATCACTTTAGGCTTTGTTGATTTAC
TACGTGATGATTTTGTTGAAAAAGATCGAAGTCGTGGTATTTATTTCACT
CAAGATTGGGTCTCTCTGCCAGGTGTTCTGCCTGTGGCTTCAGGGGGTAT
TCATGTTTGGCATATGCCTGCTCTGACCGAGATCTTTGGAGATGATTCCG
TACTACAGTTCGGCGGAGGAACTTTAGGACACCCTTGGGGTAATGCACCC
GGTGCCGTAGCTAATCGAGTTGCTCTAGAAGCATGTGTACAAGCTCGTAA
TGAAGGACGTGATCTTGCTCGTGAAGGTAATGAAATTATCCGTGAGGCTA
GTAATTGGAGTCCTGAACTAGCTGCTGCTTGTGAAGTATGGAAGGAGATC
GAATTTGAATTCGAACCAGTGGATACTTT----------------CTGGT
GAC--------------ACGAGGATTTTCAGTCCTTTGCTCTA-------
-CCAGCTGAG-CTATCCCGACCATTCACGACGCATCATCC----------
--CCAT-TTTAATAGATGACGTGGGTCTATGTCAATTAAAAAGACGAAAA
AAGGATTACAAAGTCTTATCCAGGCCTCGGTGGAATTTCTTAGATCTTCA
A-----------------------------AAAAAAGAAGA-CTTTG---
TGAGTTTCAGTACAGTACGAGCGATAATATGATCA---------------

----------------------------------TTCCAATTT-ACAATA
ATTTCCAAT---------CGGAGTTACTTGCTCAATGATGTTCATTTGTA
CGTGTATCATATATATGGCTT---GT--------GAAAAATTAATGAAGG

AGAAACATAACAGAGTTTGAACCGCTAAC---CAAATGAGAGTATAGGAT
AAAAAATTAGGGAATCAAACG--GGCCTTT-TTGGTTG--GGGATAGAGG
GACTTGAACCC---------------------------------------

>Siegfriedia_darwinioides

------------------------------CAAGTGTTGGATTCAAAGCC
GGTGTTAAAGATTATAAATTGACTTATTACACTCCTGAATATGAAACCAA
AGATACTGATATCTTGGCAGCGTTTCGAGTAACTCCTCAACCTGGAGTTC
CGCCTGAGGAAGCGGGGGCCGCGGTAGCTGCTGAATCTTCTACTGGTACA
TGGACAACTGTATGGACTGACGGGCTTACCAGTCTTGATCGTTACAAAGG
TCGATGCTACCACCTCGAGCCCGTTGCTGGAGAAGAAAATCAATATATTG
CTTATGTAGCTTACCCCTTAGACCTTTTTGAAGAAGGTTCCGTTACTAAC
ATGTTTACTTCCATTGTGGGTAATGTTTTTGGGTTCAAGGCCCTGCGCGC
TCTACGTCTGGAGGATTTGCGAATCCCTCCTGCTTATTCTAAAACTTTCC
AAGGACCGCCTCACGGCATCCAAGTTGAAAGAGATAAATTGAACAAGTAT
GGCCGCCCCCTATTGGGATGTACTATTAAACCTAAATTGGGGTTATCCGC
TAAGAATTACGGTAGAGCAGTTTATGAATGTCTTCGCGGTGGACTTGATT
TTACCAAAGATGATGAGAACGTGAATTCCCAACCATTTATGCGTTGGAGA
GACCGTTTCTTGTTTTGTGCCGAAGCCCTTTATAAAGCACAGGCTGAAAC
AGGTGAAATCAAAGGGCATTACTTGAATGCTACTGCAGGTACATGCGAAG
AAATGATTAAAAGGGCTGTATTTGCCAGAGAATTGGGAGTTCCTATTGTA
ATGCACGATTACTTAACAGGGGGATTCACTGCCAATACTAGCTTGGCTCA
TTATTGCCGAGATAATGGCCTACTTCTTCACATCCACCGTGCAATGCATG
CAGTTATTGATAGACAGAAAAATCATGGTATACACTTTCGTGTACTAGCT
AAAGCGTTACGTCTGTCTGGTGGAGATCATATTCACTCTGGTACTGTAGT
AGGTAAACTTGAAGGGGAAAGAGAAATCACTTTAGGCTTTGTTGATTTAC
TACGTGATGATTTTGTTGAAAAAGATCGAAGCCGTGGTATTTATTTCACT
CAAGATTGGGTCTCTCTACCAGGTGTTCTACCTGTGGCTTCAGGGGGTAT
TCATGTTTGGCATATGCCTGCTCTGACCGAGATCTTTGGAGATGATTCCG
TACTACAGTTTGGCGGAGGAACTTTAGGACACCCTTGGGGAAATGCACCC
GGTGCCGTAGCTAATCGAGTAGCTCTAGAAGCATGTGTACAAGCTCGTAA
TGAGGGACGTGATCTTGCTCGTGAGGGTAATGAAATTATCCGTGAGGCTA
GTAAATGGAGTCCTGAACTAGCTGCTGCTTGTGAAGTATGGAAGGAGATC
AAATTTGAATTCGAAGCAATGGATACTTTGTAA-----------------
---------TGGAATGAGCCTTGGTATGGAAACCTACCAAGTGATAACTT
TCAAATTCAGAGAAACCCTGGAATTACAAATGGGCAATCCTGAGCCAAAT
CCTGTTTTCTGAAAACAAACAAGTATTCA-------GAA---A---GTGA
TAATCAAAAAGGATAGGTGCAGAGACTCAATGGAAGCTGTTCTAACAAAT
GG----GGTTGGCTGCGATGCGTT------AGTAAAGGAAT-CCTTCCAT
CGAAACTCCAGAAAGGATGAAGAATAAACCTATATATACGTATACGTACT
GAAATACTATCTCCAAACCAAATGATTAANGGCGGCCCGAATATTTTTTT
TTTTT---TTT------TTACTTGGAA-----------------------
-----------------------T--------------CGATTCCAAGTA

AAAAAAAATATTCATTGATCAAATCATTTACTCCATCGTAATCTGAT---
-AGATCTTTTGAAAAATGGAT------TAATCGGACGAGAATAAAGATAG
AGTCCCATTCTACATGTCAA------------TATCGACAACAATGAAAT
TTATAGTAAGAGGAAAATCCGTCGACTTTA-GAAATCGTGAGGGTTCAAG
TCCCTCTATCCCCAAAAAGGC-CCAGTTGATTCCCTAATTTTTTATC---
--GTATACT-C----TCATTTCGTTATGGGTTCAAAATTCATTATCTTTC
TCATTC------ATTAATT-TTTT--------------------------
------TCT--TTTCACAAGCCTTGT----GGTATATATGATACACA---
----TAC--------AAATGAACATCA--TTGAGCAAGT--AACCCCGAT
TGTAAATTGG-------AATGATC-ATATT--------ATCGCCCGTACT
GTACTGAAA----CTTACAAAGTCTTTTTT----TTTAAGAT---CTAAG
AAATT----CCACCAAGGCCTGGATAACACTTTGTAAT-----CCCTTTT
CGTCTTTTTAATTGACATAGACCCAAGTCATCTATTAA------AATGAG
GATGATGCGCCGTG-----AATGGTCGGGATAGCTCAGCTGGTAGAGCAG
AGGACTGAAAATCCTCGTGTCACCAGTT----------------------
GGCGGTTCGCTGCCCGCGACGTCGCG-AGAATCCACTGAACCTTATCATT
TAGAGGAAGGAGAAGTCGTAACAAGGTTTCCGTAGGTGAACCTGCGGAAG
GATCATTGT-CGAACCCT-GCCTAGCAGAACGATCCGCGAACACGTAAAA
AAC--ATATTGGGGGGCTCGG---GGCCTTAGGC--CTT-GGGCTCTCT-
TGGCCGAG-GGCTGCA---TGCTGTG--CGTCGCTCGCCGATGCATAT--
GTGCTGCTTCCTCGGCTGC--AAAAACGAACCCCGGCGCAAACCGCGCCA
AGGAA--CATCAAACGAATTAGCAC-TGCCCCGTCACCCCAGAAATGGTG
TGTGGTCGGGGTGTGCGT--CGTATTC--AACTAAGTCAAAACGACTCTC
GGCAACGGATATCTCGGCTCTCGCATCGATGAAGAACGTAGCGAAATGCG
ATACTTGGTGTGAATTGCAGAATCCCGTGAACCATCAAGTCTTTGAACGC
AAGTTGCGCCCGAAGCCA--TTAGGCCGAGGGCACGTCTGCCTGGGCGTC
ACACAATGTTGCCCCCCAAC-CTCGATCTCGAA-----------------
-----------GG---CGGGGGGCGGATGCT-GGCCTCCGTGCCTCACGG
TTTG--CGGTTGGCCGAAAT--ACGAGTCCCCGGCGACGAGTGCCGCAGC
AATCGGTGGTTCTCCAACCCTCG--TGCCCCGCTGCATGCACAGATCGCC
GCTGTGGACGTACAGACC---CCAA-TGCGT----CGCAAATGCGGCGTC
TACAATGCGACCCCAGTCAGGCGGACCACCCG------------------

>Trymalium_floribundum

----------------------------AGCAAGTGTTGGATTCAAAGCC
GGTGTTAAAGATTATAAATTGACTTATTACACTCCTGACTATGAAACCAA
AGATACTGATATCTTGGCAGCGTTTCGAGTAACTCCTCAACCTGGAGTTC
CGCCTGAGGAAGCGGGGGCCGCGGTAGCTGCTGAATCTTCTACTGGTACA
TGGACAACTGTATGGACTGACGGGCTTACCAGTCTTGATCGTTACAAAGG
TCGATGCTACCACATCGAGCCCGTTGCTGGAGAAGAAACTCAATTTATTG
CTTATGTAGCTTACCCCTTAGACCTTTTTGAAGAAGGTTCTGTTACTAAC
ATGTTTACTTCCATTGTGGGTAATGTTTTTGGGTTCAAGGCCCTGCGCGC
TCTACGTCTGGAGGATTTGCGAATCCCTCCTGCTTATTCTAAAACTTTCC
AAGGACCGCCTCACGGCATCCAAGTTGAAAGAGATAAATTGAACAAGTAT
GGCCGCCCCCTATTGGGATGTACTATTAAACCTAAATTGGGGTTATCCGC
TAAGAATTACGGTAGAGCGGTTTATGAATGTCTTCGCGGTGGACTTGATT
TTACCAAAGATGATGAGAACGTGAATTCCCAACCATTTATGCGTTGGAGA
GACCGTTTCTTGTTTTGTGCCGAAGCCCTTTATAAAGCACAGGCTGAAAC
AGGTGAAATCAAAGGGCATTACTTGAATGCTACTGCAGGTACATGCGAAG
AAATGATTAAAAGGGCTGTATTTGCCAGAGAATTGGGAGTTCCTATTGTA
ATGCACGATTACTTAACAGGGGGATTCACTGCCAATACTAGCTTGGCTCA
TTATTGCCGAGATAATGGCCTACTTCTTCACATCCACCGTGCAATGCATG
CAGTTATTGATAGACAGAAAAATCATGGTATACACTTTCGTGTACTAGCT
AAAGCGTTACGTATGTCTGGTGGAGATCATATTCACTCTGGTACTGTAGT
AGGTAAACTTGAAGGGGAAAGAGAAATCACTTTAGGCTTTGTTGATTTAC
TACGTGATGATTTTGTTGAAAAAGATCGAAGCCGTGGTATTTATTTCACT
CAAGATTGGGTCTCTCTACCAGGTGTTCTACCTGTGGCTTCAGGGGGTAT
TCATGTTTGGCATATGCCTGCTCTGACCGAGATCTTTGGAGATGATTCCG
TACTACAGTTTGGCGGAGGAACTTTAGGACACCCTTGGGGAAATGCACCC
GGTGCCGTAGCTAATCGAGTAGCTCTAGAAGCATGTGTACAAGCTCGTAA
TGAGGGACGTGATCTTGCTCGTGAGGGTAATGAAATTATCCGTGAGGCTA
GTAAATGGAGTCCTGAACTAGCTGCTGCTTGTGAAGTATGGAAGGAGATC
AAATTTGAATTCGAAGCAATGGATACTTTGTAA-----------------
----TTAATTGGATTGAGCCTTGGTATGGAAACCTACCAAGTGATAACTT
TCAAATTCAGAGAAACCCTGGAATTACAAATGGGCAATCCTGAGCCAAAT
CCTGTTTTCTGAAAACAAACAAGTATTCA-------GAA-----------
--------AAGGATAGGTGCAGAGACTCAATGGAAGCTGTTCTAACAAAT
GG----AGTTGGCTGCGATGCGTT------AGTAAAGGAAT-CCTTCCAT
CGAAACTCCAGAAAGGATGAAGAATAAACCTATATA--CGTATACGTACT
GAAATACTATCTCCAAACCAAATGATTAATGACGACCCGAATATTTTTTT

TTTATATGTTT------ATATGAAAAATGAAAGAA--------------T
--------------TGTTG-----TGAATCGATTCCAAGTAAAAAAAAAA
AAATGGAATATTCATTGATCAAATCATTTACTCCATCGTAATCTGAT---
-AGATCTTTTGAAAAATGGAT------TAATCGGACGAGAATAAAGATAG
AGTCCCATTTTACATGTCAA------------TATCGACAACAATGAAAT
TTATAGTAAGAGGAAAATCCGTCGACTTTA-GAAATTGTGAGGGTTCAAG
TCCCTCTATCCCCAAAAAGGC-CCAGTTGATTCCCTAATTTTTTATC---
--CTATACT-C----TCATTTCGTTATCGGTTCAAAATTCATTATCTTTC
TCATTC------ATTAATTTTTTT------------------------
------CTT--TTC------ACAAGC----CTTATATATGATACACA---
----TAC--------AAATGAACATCA--TTGAGCAAGT--AACCCCGAT
TGTAAATTGG-------AATGATC-ATATT--------ATCGCCCGTACT
GTACTGAAA----CTTACAAAGTCTTTTTT------TTTTAAGATCTAAG
AAATT----CCACCAAGGCCTGGATAACACTTTGTAAT----CCCT-TTT
CGTCTTTTTAATTGACATAGACCCAAGTCATCTATTAA------AATGAG
GATGATGCGTCGTG-----AATGGTCGGGATAGCTCAGCTGGTAGAGCAG
AGACTG--

------------------------------------CTAACCTGCGGAAG
GATCATTGTCGA-ACCCT-GCCTAGCAGAACGACCCGCGAACATGTAAAT
AAAC-ATATCGGGGGGCTCGGG--GCCTTAGGGC--CTC-GGCTCTCT-
TGGTCGGG-GACTGCA---TGTTGTG--CGTCGCTTGCCGATGCATGGGT
GCTG--CTTCCCCGACTGC--ACAAACGAACCCCGGCGCAAACCGCGCCA
AGGAA--CATCCAATGAATTAGCAC-TGCCCCATCACCCCAGAGATGGTG
TGTGGTCGGGGTGTGCGT--CTTATTC--AACTATGTCAAAACGACTCTC
GGCAACGGATATCTCGGCTCTCGCATCGATGAAGAACGTAGCGAAATGCG
ATACTTGGTGTGAATTGCAGAATCCCGTGAACCATCAAGTCTTTGAACGC
AAGTTGCGCCCGAAGCCA--TTAGGCCGAGGGCACGTCTGCCTGGGCGTC
ACACAATGTTGCCCCCCCAACCTCGATCTCGAA-----------------
-----------GGT--GGAGGGGCGGATGCTGGCCTCCCGTGCCTCACGG
TTTG--CGGCTGGCCGAAAT--GCAAGTCCTCGGCGACGAGTGCCGCAGC
AATCGGTGGTTCTACAACCCTCG-GTTCCTTGATGCGTGCACCGATATCT
GTCGGGACG-GATAGACC---CCAA-AGCGC----GGCACATGCGGAAAA
GAAATT--

>Schistocarpaea_johnsonii

--------------------GACTTATTACACTCCTGAATATGAAACCAA
AGATACTGATATCTTGGCAGCGTTCCGAGTAACTCCTCAACCCGGAGTTC
CGCCTGAAGAAGCAGGGGCCGCGGTAGCTGCTGAATCTTCTACTGGTACA
TGGACAACTGTATGGACTGACGGGCTTACCAGTCTTGATCGTTACAAAGG
TCGATGCTACCACCTCGAGCCCGTTGCTGGAGAAGAAAATCAATATATTG
CTTATGTAGCTTACCCTTTAGACCTTTTTGAAGAAGGTTCTGTTACTAAC
ATGTTTACTTCCATTGTGGGTAACGTATTTGGGTTCAAGGCCCTGCGCGC
TCTACGTCTGGAGGATTTGCGAATCCCTCCTGCTTATTCTAAAACTTTCC
AAGGACCGCCTCATGGCATCCAAGTTGAAAGAGATAAATTGAACAAGTAT
GGTCGTCCCCTATTGGGATGTACTATTAAACCTAAATTGGGGTTATCTGC
TAAGAATTACGGTAGAGCCGTTTATGAATGTCTTCGCGGTGGACTTGATT
TTACCCNNGATGATGAGAACGTGAATTCCCAACCATTTATGCGTTGGAGA
GACCGTTTCTTATTTTGTGCCGAAGCACTTTATAAAGCACAGGCTGAAAC
AGGTGAAATCAAAGGGCATTACTTGAATGCTACTGCAGGTACATGCGAAG
AAATGATCAAAAGAGCTGTATTTGCCAGAGAATTGGGAGTTCCTATCGTA
ATGCATGATTACTTAACAGGGGGATTCACTGCAAATACTAGCTTGGCTCA
TTATTGCCGAGATAATGGTCTACTTCTTCACATCCACCGTGCAATGCATG
CAGTTATTGATAGACAGAAAAATCATGGTATACACTTTCGTGTACTAGCT
AAAGCGTTACGTATGTCTGGTGGAGATCATATTCACGCTGGTACTGTAGT
AGGTAAACTTGAAGGGGAAAGAGACATCACTTTAGGCTTTGTTGATTTAC
TGCGTGATGATTTTATTGAAAAAGATCGAAGCCGTGGTATTTATTTCACT
CAAGATTGGGTCTCTCTACCAGGTGTTCTGCCTGTGGCTTCAGGGGGTAT
TCATGTTTGGCATATGCCTGCTCTGACCGAGATCTTTGGAGATGATTCCG
TACTACAGTTTGGCGGAGGAACTTTAGGACACCCTTGGGGAAACGCACCC
GGTGCCGTAGCTAATCGAGTAGCTCTAGAAGCATGTGTAAAAGCTCGTAA
TGAGGGACGTGATCTTGCTCGTGAGGGTAATGAAATTATCCGTGAGGCTA
GTAAATGGAGTCCTGAACTAGCTGCTGCTTGTGAAGTATGGAAGGAGATC
AAATTTGAATTCGAAGCAATGGATACTTTGTAA-----------------
--TTAATTGGATTGGAGCCTTG-GTATGGAAACCTACCAAGTGATAACTT
TCAAATTCAGAGAAACCCTGGAATTACAAATGGGCAATCCTGAGCCAAAT
CCTGTTTTCTGAAAACAAACAAGGATTCA-------GAA---A---GCGA
TAATAAAAAAGGATAGGTGCAGAGACTCAATGGAAGCTGTTCTAACAAAC
GG----AGTTGGCTGCGATGCGTT------AGTAAAGGAAT-CCTTCCAT

CGAAACTCCAGAAAAGATGAAGAATAAACCTATATATACGTATACGTACT
GAAATACTATCTCCAAACCAAATGATTAATGACGACCCGAATCTTTTTTT
TTTTT---TAT------TTATATGTTTATATGAAA--------------A
ATGAAAGAATTGTTGTGAA-----TCGATTCCAAGTAAAAAAGTAAAAAA
AAATGGAATATTCATTGATCAAATCATTTACTCCATCGTAATCTGAT---
-AGATCTTTTGAAAAATGGAT------TAATCGGACGAGAATAAAGATAG
AGTCCCATTCTACATGTCAA------------TATCGACAACAATGAAAT
TTATAGTAAGAGGAAAATCCGTCGACTTTA-GAAATCGTGAGGGTTCAAG
TCCCTCTATCCCCAAAAAGGC-CCTTTTGATTCCCTAATTTTTTATC---
--CTATACT-C----TCATTTCGTTAGCGGTTCAAAATTCGTTATGTTTC
TCATTC------ATTAATT-TTTT--------------------------
------TCT--TTTCACAAGCCTTGT----GATATATATGATACACG---
----TAC--------AAATGAACATCA--TTGAGCAAGT--AACCCCGAT
TGTAAACTGG-------AATGATC-ATATT--------ATCGCTCGTACT
GTACTGAAA----CTTACAAAGTCTTCTTT----T-TGAA--GATCTAAG
AAATT----CCACCAAGGCCTGGATAAGACTTTGTAAT----CCCCCTTT
CGTCTTTTTAATTGACATAGACCCAAGCCATCTATTAA------AATGAG
GATGATGCGTCGTG-----AATGGTCGGGATAGCTCAGCTGGTAGAGCAN
AGGACTGAAAATCCT-----------------------------------

--CCTGCGGGAG
GATCATTGTCGAAA-CCT-GCCTAGCAGAACGACCCGTGAACCAGTT--A
AAACATAC-CGGGGGCTCAGG---G-CCTTAGGC--CTT-GGTCTCTCT-
TGGTCGGGGGGCTGCA---TCTTGTGCATTG--CTTGTCGATGCACGGGT
GCTG--CTTCCCCGGCCGC-A-CAAACGAACCCCGGCGCAAACCG-CGCC
-AAGGAACATCTAACGAATTGGCAC-TGCCCCGCCGCCCCAGAGATGGTG
TGCGGTTGGGGTGTGCGT--CGTATTC---TATATGTCAAAACGACTCTC
GGCAACGGATATCTCGGCTCTCGCATCGATGAAGAACGTAGCGAAATGCG
ATACTTGGTGTGAATTGCAGAATCCCGTGAACCATCGAGTCTTTGAACGC
AAGTTGCGCCCGAAGCCT--TTAGGCCGAGGGCACGTCTGCCTGGGCGTC
ACACAACGTTGCCCCCCCAACCTCGA------------------------
------------AGGCGAGGGGGCGGATGCTGGCCTCCCGTGTGCCACGG
TTTA--CGGCTGGCCGAAAT--GCGAGTCCTCGGCGACGAGTGCCGCAGC
AATCGGTGGTTCTCCAACCCTCG-GTGCCCTGTTGCGTGCATGGATCGCT
GTTGTGGCCCGATAG-AC---CCCA-ATGCGC---TGCTAATGCGGCGTC
TCCAACGCGACCTCAGGTCAGGCGGGGC----------------------

>Lasiodiscus_mildbraedii


```
--------------------------------------------------
--------------------------------------------------
--------------------------------------------------
--------------------------------------------------
--------------------------------------------------
--------------------------------------------------
--------------------------------------------------
--------------------------------------------------
--------------------------------------------------
--------------------------------------------------
--------------------------------------------------
--------------------------------------------------
--------------------------------------------------
--------------------------------------------------
--------------------------------------------------
--------------------------------------------------
--------------------------------------------------
--------------------------------------------------
--------------------------------------------------
--------------------------------------------------
--------------------------------------------------
--------------------------------------------------
--------------------------------------------------
--------------------------------------------------
--------------------------------------------------
--------------------------------------------------
--------------------------------------------------
--------------------------------------------------
-------------------------TAAAGCAAGTGTTGGATTCAAAGCT
GGTGTTAAAGAGTATAGATTGACTTATTACACTCCTGACTATGAAACCAA
AGATACTGATATCTTGGCAGCGTTTCGAGTAACTCCTCAACCCGGAGTTC
CGCCTGAGGAAGCAGGGGCCGCGGTAGCTGCTGAATCTTCTACTGGTACA
TGGACAACTGTATGGACTGACGGGCTTACCAGTCTTGATCGTTACAAAGG
TCGATGCTACCACCTCGAGCCCGTTGCTGGAGAAGAAAATCAATATATTG
CTTATGTAGCTTACCCCTTAGACCTTTTTGAAGAAGGTTCTGTCACTAAC
ATGTTTACTTCCATTGTGGGTAATGTTTTTGGGTTCAAGGCCCTGCGCGC
TCTACGTCTGGAGGATTTGCGAATCCCTCCTGCTTATTCTAAAACTTTCC
AAGGACCGCCTCACGGCATCCAAGTTGAAAGAGATAAATTGAACAAGTAT
GGCCGCCCCCTATTGGGATGTACTATTAAACCTAAATTGGGGTTATCCGC
TAAGAATTACGGTAGAGCAGTTTATGAATGTCTTCGCGGTGGACTTGATT
TTACCAAAGATGATGAGAACGTGAATTCCCAACCATTTATGCGTTGGAGA
GACCGTTTCTTATTTTGTGCCGAAGCCCTTTATAAAGCACAGGCTGAAAC
AGGTGAAGTCAAAGGGCATTACTTTAATGCTACTGCAGGTACATGCGAAG
AAATGATTAAGAGGGCTGTATTTGCCAGAGAATTGGGAGTTCCTATTGTA
ATGCATGACTACTTAACAGGGGGATTCACTGCAAATACTAGCTTGGCTCA
TTATTGCCGAGATAATGGTCTACTTCTTCACATCCACCGTGCAATGCATG
CAGTTATTGATAGACAGAAAAATCATGGTATGCACTTTCGTGTACTAGCT
AAAGCGTTACGTCTGTCTGGTGGAGATCATATTCACGCTGGTACTGTAGT
AGGTAAACTTGAAGGGGAAAGAGAAATCACTTTAGGCTTTGTTGATTTAC
TACGTGATGATTTTGTTGAAAAAGATCGAAGCCGTGGTATCTATTTCACT
CAAGATTGGGTCTCTATACCAGGTGTTATACCCGTGGCTTCAGGGGGTAT
TCATGTTTGGCATATGCCTGCTCTGACCGAGATCTTTGGAGATGATTCCG
TACTACAGTTCGGCGGAGGAACTTTAGGACACCCTTGGGGAAATGCACCC
GGTGCCGTAGCTAATCGAGTAGCTCTAGAAGCATGTGTACAAGCTCGTAA
TGAGGGACGTGATCTTGCTCGTGAGGGTAATGAAATTATCCGTGAGGCTA
GTAAATGGAGTCCTGAACTAGCTGCTGCCTGTGAAGTATGGAAGGAGATC
AGGTTTGAATTCCAAGCAATGGATACTTTGTAA-----------------
----TTAATTGGATGGAGCCTTGGTATGGAAACCTACCAAGTGATAACTT
TCAAATTCAGAGAAACCCTGGAATTACAAACGGGCAATCCTGAGCCAAAT
CCTGTGTTCTGAAAACAAACAAGGATTCA-------GAA---A---GTGA
```

TAATAAAAAAGGATAGGTGCAGAGACTCAATGGAAGCTGTTCTAACAAAT
GG----AGTTGGCTGCGAAGCGTT------AGTAAAGGAAT-CCTTCCAT
CGAAACTCCAGAAAGGATGAAGAATAAACCTATATATACGTATACGTACT
GAAATACTATCTCCAAACCAAATGATTAATGACGACCCGAATCTTTTTTT
TTATA-----T------TTATATGTTTATATGAAA--------------A
ATGAAAGAATTGTTGTGAA-----TCGATTCCAAGTAAAA-------AAA
AAATGGAATATTCATTGATCAAATCATTTACTCCATCGTAATCTGAT---
-AGATCTTTTGAAAAATTGAT------TAATCGGACGAGAATAAAGATAG
AGTCCCATTCTACATGTCAA------------TATCGACAACAATGAAAT
TTATAGTAAGAGGAAAATCCGTCGACTTTA-GAGATCGTGAGGGTTCAAG
TCCCTCTATCCCCAAAAAGGC-CCATTTGATTCCCTAGTATTTTATC---
--CTATACT-C----TCATTTCGTTAGCGGTTCAAAATTCGTTATGTTTC
TCATTC------AATAATT-TGTT--------------------------
------TCT--TTTCACAAGCCTTG-----TGTATATATGATACACG---
----TAC--------AAATGAACATCA--TCGAGCAAGT--AACCCCGAT
TGTAAATTGG-------AATGATC-ATATT--------ATCGCTCGTACT
GTACTGAAA----CTTACAAAGTCTTCTTT----TTGAAGATCTAAGAAG
AAATT----CCGCCAAGGCCTGGATAAGACTTTGTAAT----CCCCTTTT
CGTCTTTTTAATTGACATAGACCCAAGTCATCTATTAA------AATGAG
GATGATGCGTCGTG-----AATGGTCGGGATAGCTCAGCTGAAGGCAGAG
GACTGAATAA--
-GCGGTTCGCTGCCTGCAACGTCGCGAGAAGTCCACTGAACCTTATCATT
TAGAGGAAGGAGAAGTCGTAACAAGGTTTCCGTAGGTGAACCTGCGGAAG
GATCATTGTCGAAA-CCT-GCACAGCAGAACGACCCGTGAACACGTAAGT
ACGTATCAGGGG---GCCCGG---GGCCTCAGGC--CCC-GGGCTCTCT-
TTGTCGGGGCACTGTA---TCTCGTGTCT-TTCCTTGTCAAAGCACGGGA
GCAG--TTCTCTCGGCTTC--ACAAACAAACCCCGGCGCAAACTG--CGC
CAAGGAACATTTAACGTATTGGCAA-CGCCCCGCCACCCCAGAGATGGGG
TGTGGTTGGGGTGTGCTT--CGTATTC---TATATGTCAAAACGACTCTC
GGCAACGGATATCTCGGCTCTCGCATCGATGAAGAACGTAGCGAAATGCG
ATACTTGGTGTGAATTGCAGAATCCCGTGAACCATCGAGTCTTTGAACGC
AAGTTGCGCCCGAAGCCA--TTAGGCCGAGGGCACGTTTGCCTGGGCGTC
ACACAACGTTGCCCCCCTTAACCTTGACCTTGA-------AGG-------
-----------C---CGGGGGGGCGGATGGTGGCCTTCCGTGTGCCAGGC
AA-G--CGG-TTGGCTGAAA--TGCGATTCTTGGCGACGAGTGCCGCAAC
ATTCGGTGGTTCTCCAACCTTTG-TGCCCTGTTGCGTGCACCGACCGCCT
TCTGCGGCCCGATAGACC---CCAC-TGCGC----CCTCGTTGTGGCGCC
ACCAACGGACCCAG---TCAGGCGGGTAC---------------------

>Granitites_intangendus

--TCAAAGCK
GGTGTTAAAGAGTATAAATTGACTTATTACACTCCTGACTATGAAACCAA
GAATACTGATATCTTGGCAGCGTTTCGAGTAACTCCTCAACCTGGAGTTC
CGCCTGAGGAAGCAGGGGCCGCGGTAGCTGCTGAATCTTCTACCGGTACA
TGGACAACTGTATGGACTGACGGGCTTACCAGTCTTGATCGTTACAAAGG
TCGATGCTACCACCTCGAGCCCGTTGCTGGAGAAGAAAATCAATATATTG
CTTATGTAGCTTACCCCTTAGACCTTTTTGAAGAAGGTTCTGTTACTAAC
ATGTTTACTTCCATTGTGGGTAATGTTTTTGGGTTCAAGGCCCTGCGCGC
TCTACGTCTGGAGGATTTGCGAATCCCTCCTGCTTATTCTAAAACTTTCC
AAGGACCGCCTCACGGCATCCAAGTTGAAAGAGATAAATTGAACAAGTAT
GGCCGCCCCCTATTGGGATGTACTATTAAACCTAAATTGGGGTTATCCGC
TAAGAATTACGGTAGAGCGGTTTATGAATGTCTTCGCGGTGGACTTGATT
TTACCAAAGATGATGAGAACGTGAATTCCCAACCATTTATGCGTTGGAGA
GACCGTTTCTTATTTTGTGCCGAAGCCCTTTATAAAGCACAGGCTGAAAC
AGGTGAAGTCAAAGGGCATTACTTGAATGCTACTGCAGGTACATGCGAAG
AAATGATTAAAAGGGCTGTATTTGCCAGAGAATTGGGAGTTCCTATTGTA
ATGCATGATTACTTAACAGGGGGATTCACTGCAAATACTAGCTTGGCTCA
TTATTGCCGAGATAATGGTCTACTTCTTCACATCCACCGTGCAATGCATG
CAGTTATTGATAGACAGAAAAATCATGGTATACACTTTCGTGTACTAGCT
AAAGCGTTACGTATGTCTGGTGGAGACCATATTCACTCTGGTACTGTAGT
AGGTAAACTTGAAGGGGAAAGAGACATCACTTTAGGCTTTGTTGATTTAC
TACGTGATGATTTTATTGAAAAAGATCGAAGCCGTGGTATTTATTTCACT
CAAGACTGGGTCTCTCTACCAGGTGTTTTGCCTGTGGCTTCGGGGGGTAT
TCATGTTTGGCATATGCCTGCTCTGACCGAGATCTTTGGAGATGATTCCG
TACTACAGTTTGGCGGAGGAACCTTAGGACACCCTTGGGGAAATGCACCC
GGTGCCGTAGCTAATCGAGTAGCTCTAGAAGCATGTGTACAAGCTCGTAA
TGAGGGACGTGATCTTGCTCGTGAGGGTAATGAAATTATCCGTGAGGCTA
GTAA--

---------------------TTGGTTGGAAACTTACCAAGTGATAACTT

TCAAATTCAGAGAAACCCCGGAATTACAAATGGGCAATCCTGAGCCAAAT
CCTGTTTTCTGAAAACAAACAAGGATTCA-------GAA---A---GTGG
TAATAAAAAAGGATAGGTGCAGAGACTCAATGGAAGCTGTTCTAACAAAC
AAATGGAGTTGGCTGCGATGCGTTAGT------AAAGGAAT-CCTTCCAT
CGAAACTCCAGAAAGGGTGAAGAATAAACCTATGTATACGTATACGTACT
GAAATACTATCTCCAAACCAAATGATTAATGACGACCCGAATCTTTTTTT
TTTTTATTTAT------ATGTTCATATGAAACATG-AAA-----------
------AAATTGTTGTGAA-----TCGA---TTCCAAGTAAAAAAAAAAA
AAACGGAATACTCGTTGATCAAATCATTTACTCCATCGTAATCTGAT---
-AGATCTTTTGAAAAATGGAT------TAATCGGACGAGAATAAAGATAG
AGTCCCATTCTACATGTCAA------------TCTCGACAACAATGAAAT
TTATAGTAAGAGGAAAATCCGTCGACTTTA-GAAATCGTGAGGGTTCAAG
TCCCTCTATCCCCAAAAAGGC-CCATTTGATTCCCTAATTTTTTATC---
--CTA-----TACTCTCATTTCGTTAGCGGTTCAAAATCCGTTATGTTTC
GCATTCATTAATATTTTCCTTTTC--------------------------
------ACAA----------------------TATATATGATATACG---
----TAC--------AAATGAACATCA--TTGAGCAAGT--AACCCCAAT
TGTAAATTGG-------AATAATC-AGATT--------ATTGCTCGTACT
GAAACTTA---------CAA-------AGTCTTCTTTTTGAAGATCTAAG
AAATT----CCACCAAGGCCTGGATAAGACTTTGTAAT----CCCTTTTT
CGTCTTTTTAATTGACAGAGGCCCAAGTCATCTGTTAA------AATGAG
GATGATGCGTCGTG-----AATGGTCGGGATAGCTCAGCTGGTAGAGCAG
AGG---

------------TA-CCT-GCCTAGCAGAGCGACCCGTGAACCCGTATAT
ACACATAACGGGGGGCTCGGG---CCCTTGGGGC--CTC-GGGCCCTCT-
TGGTCGGGGGGTTGCA---TCTTTGTGCTTCGCTCTGTCGATGCAAGGCT
GCTG--CTCTCCCGGCCGC-AAAAAACGAACCCCGGCGCAAACCG-CGCC
-AAGGAACATCTAACGAATTGGCT--TGCCCCACCGCCCCAGAGATGGTG
TGCGGTCGGGGTGTGCGT--CGTATTT---TATATGTCAAAACGACTCTC
GGCAACGGATATCTCGGCTCTCGCATCGATGAAGAACGTAGCGAAATGCG
ATACTTGGTGTGAATTGCAGAATCCCGTGAACCATCGAGTTTTTGAACGC
AAGTTGCGCCCGAAGCCA--TTAGGCCGAGGGCACGTCTGCCTGGGCGTC
ACACAACGTTGCCCCCCCAACCTCTTCCTTGG---------AG-------
-----------GC--GAAGGGAGCGGATGCTGGCCTCCCGTGTGCCACGG
TTTG--CGGCTGGCCAAAAT--GCGAGTCCTCGGCGACGAGTGCCGCAGC
AATCGGTGGTTCTCCAACCCTCG-GTGCCCTGCTGCGTGCACAGACCGCT
GTTGCGACCGGATAAGAC---CCCA-GTGCGC---TGCTAATGCAGCGTC
TCAACGCA--

>Stenanthemum_complicatum

---------------------------AAGCAAGTGTTGGATTCAAAGCT
GGTGTTAAAGATTATAAATTGACTTATTACACTCCTGACTATGAAACCAA
AGATACTGATATCTTGGCAGCGTTTCGAGTAACTCCTCAACCTGGAGTTC
CGCCCGAGGAAGCGGGGGCCGCGGTAGCTGCTGAATCTTCTACTGGTACA
TGGACAACTGTATGGACTGACGGGCTTACCAGTCTTGATCGTTACAAAGG
TCGATGCTACCACCTCGAGCCCGTTGCTGGAGAAGAAAATCAATTTATTG
CTTATGTAGCTTACCCCTTAGACCTTTTTGAAGAAGGTTCTGTTACTAAC
ATGTTTACTTCCATTGTGGGTAATGTTTTTGGGTTCAAGGCCCTGCGCGC
TCTACGTCTGGAGGATTTGCGAATCCCTCCTGCTTATTCTAAAACTTTCC
AAGGACCGCCTCACGGCATCCAAGTTGAAAGAGATAAATTGAACAAGTAT
GGCCGCCCCCTATTGGGATGTACTATTAAACCTAAATTGGGGTTATCCGC
TAAGAATTACGGTAGAGCAGTTTATGAATGTCTTCGCGGTGGACTTGATT
TTGCCAAAGATGATGAGAACGTGAATTCCCAACCATTTATGCGTTGGAGA
GACCGTTTCTTGTTTTGTGCCGAAGCCCTTTATAAAGCACAGGCTGAAAC
AGGTGAAATCAAAGGGCATTACTTGAATGCTACTGCCGGTACATGCGAAG
AAATGATTAAAAGGGCTGTATTTGCCAGAGAATTGGGAGTTCCTATTGTA
ATGCACGATTACTTAACAGGGGGATTCACTGCCAATACTAGCTTGGCTCA
TTATTGCCGAGATAATGGCCTACTTCTTCACATCCACCGTGCAATGCATG
CCGTTATTGATAGACAGAAAAATCATGGTATACACTTTCGTGTACTAGCT
AAAGCGTTACGTCTGTCTGGTGGAGATCATATTCACTCGGGTACTGTAGT
AGGTAAACTTGAAGGGGAAAGAGAAATCACTTTAGGCTTTGTTGATTTAC
TACGTGATGATTTTGTTGAAAAAGATCGAAGCCGTGGTATTTATTTCACT
CAAGATTGGGTCTCTCTACCAGGTGTTCTACCTGTGGCTTCAGGGGGTAT
TCATGTTTGGCATATGCCTGCTCTGACCGAGATCTTTGGAGATGATTCCG
TACTACAGTTTGGCGGAGGAACTTTAGGACACCCTTGGGGGAATGCACCC
GGTGCCGTAGCTAATCGAGTAGCTCTAGAAACATGTGTACAAGCTCTTAA
TGAGGGACGTGATCTTGCTCGTGAGGGTAATGAAATTATCCGTGAGGCTA
GTAAATGGAATCCTGAACTACCTGCTGCTTGTGAAGTATGGAAGGAGATC

GAATTTGAATTC-------------------------------------
---TTAATTGGATTGAGCCTTGGTATGGGAAACCTACCAAGTGATAACTT
TCAAATTCAGAGAAACCCTGGAATTACAAATGGGCAATCCTGAGCCAAAT
CCTGTTTTCTGAAAACAGACAAGTATTCA-------GAA---A---GTGA
TAATAAAAAAGGATAGGTGCAGAGACTCAATGGAAGCTGTTCTAACAAAT
GG----AGTTGGCTGCGATGCGTT------AGTAAAGGAAT-CCTTCCAT
CGAAACTCCAGGAAGGATGAAGAATAAACCTAT--ATACGTATACGTACT
GAAATACTATCTCCAAACCAAATGATTAATGACGACCCGAATATATATTT
TTTT-----TT------TTATATGTTTATATGAAA--------------A
ATGAAAGAATTGTTGTGAA-----TCGATTCCAAGTAAAAA-----AAAA
AAATGGAATATTCATTGATCAAATCGTTTACTCCATCGTAATCTGAT---
-AGATCTTTTGAAAAATGGAT------TAATCGGACGAGAATAAAGATAG
AGTCCCATTCTACATGTCAA------------TATCGACAACAATGAAAT
TTATAGTAAGAGGAAAATCCGTCGACTTTA-GAAATCGTGAGGGTTCAAG
TCCCTCTATCCCCAAAAAGGC-CCAGTTGATTCCCTAATTTTTTATC---
--CTATACT-C----TCATTTCATTATCGGTTCAAAATTCATTATGTTTC
TCATTC------ATTAATT-TTTT--------------------------
------TCT--TTTCACAAGCCTTGT----GGTATATATGATACACA---
----TAC--------AAATGAACATCA--TTGAGCAAGT--AAACCCGAT
TGTAAATTGG-------AATGATC-ATATT--------ATCGCCCGTACT
GTACTGAAA----CTTACAAAGTATTTTTT----T-TTAA--GATCTAAG
AAATT----CCACCAAGGCCTGGATAACACTTTGTAAT----CCCCTTTT
CGTCTTTTTAATTGACATAGACCCAAGTCATCTATTAA------AATGAG
GATGATGCGTCGTG-----AATGGTCGGGATAGCTCAGCTGGTAGAGCA-

-------------------------------------TAACCTGCGGAAG
GATCATTGTCGAACCTCT-GCCTAGCAGAACGACCTGCGAACCCGTAAAT
AAC--ATATCGGGGGGCTTGG---GGCCTTAGGC--CAC-GGGCTCCCT-
TGGCCGGG----GGCA---CGCTGTG--CGTCGCTCGACGAGGCACTGGC
GCTTTGCTTCCCCGGCTGC--ACAAACGAACCCCGGCGCAAACCGCGCCA
AGGAA--CATCAAACGAATTAGCAT-CGCCCCGTCACCCCAGAGATGGTG
TGTGGTCGGGGTGGGCGT--CGTATTC--AATTAAGTCAAAACGACTCTC
GGCAACGGATATCTCGGCTCTCGCATCGATGAAGAACGTAGCGAAATGCG
ATACTTGGTGTGAATTGCAGAATCCCGTGAACCATCAAGTCTTTGAACGC
AAGTTGCGCCCGAAGCCA--TTAGGCCGAGGGCACGTCTGCCTGGGCGTC
ACACAATGTTGCCCCCCAACCTCGATC-TC-GA----------------
-----------AG---GCGGGGGCGGATGCTGGCCTCCCGTGCCTTACGG
TTTG--CGGCTGGCCGAAAT--ACGAGTCCCCGGCGACGAATGCCGCAGC
AATCGGTGGTTGTCCAACCCTCG-GTGCCCTGCTGCTTGCACGAATCGCT
GCTGTGGAAAGCTAGACC---CCAA-TGCGC----CGCAGATGCGGCGTC
TACAATGCGACCTCAGGTCAGGCGGGGCTA-------------------

>Noltea_africana

--------------------------------------GGATTCAAAGCC
GGTGTTAAAGATTATAAATTGACTTATTACACTCCTGACTATGAAACCAA
AGATACTGATATCTTGGCAGCGTTTCCAGTAACTCCTCAACCTGGAGTTC
CACTTGAGGAAGCAGGGGCCGCGGTAGCTGCTGAATCTTCTACTGGTACA
TGGACAACTGTATGGACTGACGGGCTTACCAGTCTTGATCGTTACAAAGG
TCGATGCTACCACCTCGAGCCCGTTGCTGGAGAAGAAAATCAATATATTG
CTTATGTAGCTTACCCCTTAGACCTTTTTGAAGAAGGTTCTGTTACTAAC
ATGTTTACTTCCATTGTGGGTAATGTTTTTGGGTTCAAGGCCCTGCGCGC
TCTACGTCTGGAGGATTTGCGAATTCCTCCTGCTTATTCTAAAACTTTCC
AAGGACCGCCTCACGGCATCCAAGTTGAAAGAGATAAATTGAACAAGTAT
GGCCGCCCCCTATTGGGATGTACTATTAAACCTAAATTGGGGTTATCCGC
TAAGAATTACGGTAGAGCAGTTTATGAATGTCTTCGCGGTGGACTTGATT
TTACCAAAGATGATGAGAACGTGAATTCCCAACCATTTATGCGTTGGAGA
GACCGTTTCTTATTTTGTGCCGAAGCCCTTTATAAAGCACAAGCTGAAAC
AGGTGAAATCAAAGGGCATTACTTGAATGCTACTGCAGGTACATGCGAAG
AAATGATTAAAAGGGCTGTATTTGCCAGAGAATTGGGAGTTCCTATTGTA
ATGCATGATTACTTAACAGGGGGATTCACTGCAAATACTAGCTTGGCTCA
TTATTGCCGAGATAATGGTCTACTTCTTCACATCCACCGTGCAATGCACG
CAGTTATTGATAGACAGAAAAATCATGGTATACACTTTCGTGTACTAGCT
AAAGCGTTACGTATGTCTGGTGGAGATCATATTCACTCTGGTACTGTAGT
AGGTAAACTTGAAGGGGAAAGAGACATCACTTTAGGCTTTGTTGATTTAT
TGCGTGATGATTTTATTGAAAAAGATCGAAGCCGTGGTATTTATTTCACT
CAAGATTGGGTCTCTCTACCAGGTGTTCTGCCTGTGGCTTCAGGGGGTAT
TCATGTTTGGCATATGCCTGCTCTGACCGAGATCTTTGGAGATGATTCCG
TACTACAGTTCGGCGGAGGAACTTTAGGACACCCTTGGGGAAATGCACCC
GGTGCCGTAGCTAATCGAGTAGCTCTAGAAGCATGTGTACAAGCTCGTAA

TGAGGGACGTGATCTTGCTCGTGAGGGTAATGAAATTATCCGTGAGGCTA
GTAAATGGAGTCCTGAACTAGCCGCTGCTTGTGAAGTATGGAAGGAGATC
AAATTTGAATTCGAAGCAATGGATACTTTGTAA-----------------
---------------------------GGAAACCTACCAAGTCATAACTT
TCAAATTCAGA-GAAACCTGGAATTACAAACGGGCAATCCTGAGCCAAAT
CCTGTTTTCTGAAAACAAACAAGGATTCA-------GAA---A---GTGA
TAATAAAAAAGGATAGGTGCAGAGACTCAACGGAAGCTGTTCTAACAAAT
GG----AGTTGGCTGCGATGCGTT------AGTAAAGGAAT-CCTTCCAT
CGAAATTCCAGAAAGGATGAAGGATAAACCTATATATAGGTATACGTACT
GAAATACTGTCTCCAAACCAAATGATTAATGACGACCCGAATCTTTTTTT
T-TTT--ATAT------TTATAGGTTTATATGAAA--------------A
ATGAAAGAATTGTTGTGAA-----TAGATTCCAAGTAAA--------AAA
AAATGGAATATTAGTTGATCAAATCATTTACTCCATCGTAATCTGAT---
-AGATCTTTTGAAAAATTGAT------TAATCGGACGAGAATAAAGATAG
AGTCCCATTCTACATGTCAA------------TATCGACAACAATGAAAT
TTATAGTAAGAGGAAAATCCGTCGACTTTA-GAAATCGTGAGGGTTCGAG
TCCCTCTATCCCCA------C-CCATTTGATTCCCTAATTTTTTATC---
--CTATACT-C----TCATTTCGTTAGCGGTTCAAAATTCGTTATGTTTC
TCA------------AATT-TTTT--------------------------
------TAT--TTTCACAAGCCTTGT----GATATATATGATACACG---
----NAC--------AAATGAACATCA--TTGAGCACGT--AACCCCGAT
TGTAAATTGG-------AATGATC-ATATT--------ATCGCTCGTACT
GTATTGAAA----CTTACAAAGTATT-----------TTGAAGATCTAAG
AAATT----CCACCAAGGCCTGGATAAGACTTTGTAAT----CCCCTTTT
CGTCTTTTTAATAGACATAGAACCAAGTCATCTATTCA------AATGAG
GATGATGCGTCGAG-----AA-----------------------------

-------------------------------GTAGGTGA-ACTGC-GGAA
GCGACATTTCGAAA-CCT-GCCTAGCAGACACGACCGCGAACCTGTAA-A
AACATGTTGGGG---GCGAGG---GGCCTTAGGT--CTC-GGACTCTCT-
TGGTTGG-GGGGGCTG---CATTTTGTGCCTCGCTTGCCGATGCATTGGT
GTAG--CTTTCCAGGCCAC-ACAAACGAACCCCGGCGTAAAATCG--CGC
CAAGGAAAATCTAATGAATTGGCAT-TGCCCCTTCACCCCAGAAATGGTG
TGTGGTTGGGGTGTGCGT--CGTATTC---TATATATCAAAACGACTCTC
GGCAACGGATATCTCGGCTCTCGCATCGATGAAGAACGTAGCGAAATGCG
ATACTTGGTGTGAATTGCAGAATCCCGTGAACCATCGAGTCTTTGAACGC
AAGTTGCGCCCGAAGCCA--TTCGGCCGAGGGCACGTCTGCCTGGGCGTC
ACACAACGTTGCCCCTTCGACTT----TGAAG------------------
-------------GCGAAGGAGGCGGATGCTGGCCTCCCGTGTGCCACGG
CATG--CGGCTGGTCGAAAT--ACGAGTCCTCGGCGACGAGTGCCGCAAC
AATCGGTGGTTGTCCAACCCTCG-TGGCCCTGTTGCGTGCATAGATCGCT
GTTGTGACCTGATAGACC---CCAT-TG-CGT---TGCTAATGCAGCGTA
TCCAATGCGACCCCAGTCAGGCGGGCTACC--------------------

>Retanilla_trinervia

--------------------------AAAGCAAGTGTTGGATTCAAAGCC
GGTGTTAAAGATTATAAATTGACTTATTACACTCCTGACTATGAAACCAA
AGATACTGATATCTTGGCAGCGTTCCGAGTAACTCCTCAACCTGGCGTTC
CACCTGAGGAAGCAGGGGCCGCGGTAGCTGCTGAATCTTCTACTGGTACA
TGGACAACTGTATGGACTGAGGGGCTGACCAGTCTTGATCGTTACAGAGG
TCGATGCTACCACATCGAGCCCGTTGCTGGAGAAGAAAATCAATATATTG
CTTATGTAGCTTACCCCTTAGACCTTTTTGAAGAAGGTTCTGTTACTAAC
ATGTTTACTTCCATTGTGGGTAATGTATTTGGGTTCAAGGCCCTGCGCGC
TCTACGTCTGGAGGATTTGCGAATCCCCCCTGCTTATTCTAAAACTTTCC
AAGGACCGCCTCACGGCATCCAAGTTGAAAGAGATAAATTGAACAAGTAT
GGCCGTCCCCTATTGGGATGTACTATTAAACCTAAATTGGGGTTATCCGC
TAAGAATTACGGTCGAGCAGTTTATGAATGTCTTCGCGGTGGACTTGATT
TTACCAAAGATGATGAGAACGTGAATTCCCAACCATTTATGCGTTGGAGA
GACCGTTTCTTATTTTGTGCCGAAGCCCTTTATAAAGCACAGGCTGAAAC
AGGTGAAATCAAAGGGCATTACTTGAATGCTACTGCAGGTACATGCGAAG
AAATGATGAAAAGGGCTGTATTTGCCAGAGAATTGGGAGTTCCTATTGTA
ATGCATGATTACTTAACAGGGGGATTCACTGCAAATACTAACTTGGCTCA
TTATTGCCGAGATAATGGTCTACTTCTTCACATCCACCGTGCAATGCATG
CAGTTATTGATAGACAGAAAAATCATGGTATACACTTTCGTGTACTAGCT
AAAGCGTTACGTATGTCTGGTGGAGATCATATTCACGCTGGTACTGTAGT
AGGTAAACTTGAAGGGGAAAGAGACATCACTTTAGGCTTTGTTGATTTAC
TACGTGATGATTTTATTGAAAAAGATCGAAGCCGTGGTATTTATTTCACT
CAAGATTGGGTCTCTCTACCAGGTGTTCTGCCTGTGGCTTCAGGGGGTAT
TCATGTTTGGCATATGCCTGCTCTGACCGAGATCTTTGGAGATGATTCCG

TACTACAGTTTGGCGGAGGAACTTTAGGACACCCTTGGGGAAATGCACCC
GGTGCCGTAGCTAATCGAGTAGCTCTAGAAGCATGTGTACAAGCTCGTAA
TGAGGGACGTGATCTTGCTCGTGAGGGTAATGAAATTATCCGTGAGGCTA
GTAAATGGAGTGCTGAACTAGCTGCTGCTTGTGAAGTATGGAAGGAGATC
AAATTCGAATTCCAAACAATGGATACTTTGTAA-----------------

--CCTCTATCCCCAAAAAGGC-CCATTTGATTCCCTAATTTTTTATC---
--CTATACT-C----TCATTTCGTTAGCGGTTCAAAATTCGTTAGGTTTC
TCATTC------ATTCATT-TTTT--------------------------
------TCT--TTTCACAAGCCTTGT----GATATATATGATACACG---
----TAC--------AAAGGAACATCA--TTGAGCAAGT--AACCCCGAT
TGGAAATT-G-------AATGATC-AGATT--------ATCGCTCGTACT
GTACTGAAA----CTTACAAAGTCTTCTT-------GTTGAAGATCTAAG
AAATT----CCACCACGGCCTAGATAAGACTTTGTAAT----CCCCTTTT
CGTCTTTTTAATTGACATAGACCCAAGTCATCTATTAA-AATAAAATGAG
GATGATGCGTCGTG-----AATGGTCGGGATAGCTCAGCTGGTAGAGCAG
AGGACTGAAAATCCTCGTGTCAC---------------------------

>Trichocephalus_stipularis

----------------------------------TGTTGGATTCAAAGCC
GGCGTTAAAGATTATAAATTGACTTATTACACTCCTGACTATGAAACCAA
AGATACTGATATCTTGGCAGCGTTTCGAGTAACTCCTCAACCTGGAGTTC
CGCCCGAGGAAGCAGGGGCCGCGGTAGCTGCTGAATCTTCTACTGGTACA
TGGACAACTGTATGGACTGACGGGCTTACCAGTCTTGATCGTTACAAAGG
TCGATGCTACCACCTCGAGCCCGTTGCTGGAGAAGAAAATCAATATATTG
CTTATGTAGCTTACCCCTTAGACCTTTTTGAAGAAGGTTCTGTTACTAAC
ATGTTTACTTCCATTGTGGGTAATGTTTTTGGGTTCAAGGCCCTGCGCGC
TCTACGTCTGGAGGATTTGCGAATCCCTCCTGCTTATTCTAAAACTTTCC
AAGGACCGCCTCACGGCATCCAAGTTGAAAGAGATAAATTGAACAAGTAC
GGCCGCCCCCTATTGGGATGTACTATTAAACCTAAATTGGGGTTATCCGC
TAAGAATTACGGTAGAGCAGTTTATGAATGTCTTCGCGGTGGACTTGATT
TTACCAAAGATGATGAGAACGTGAATTCCCAACCATTTATGCGTTGGAGA
GATCGTTTCTTATTTTGTGCCGAAGCCCTTTTTAAAGCACAGGCTGAAAC
GGGTGAAATCAAAGGGCATTACTTGAATGCTACTGCAGGTACATGCGAAG
AAATGATTAAAAGGGCTGTATTTGCCAGAGAATTGGGAGTTCCTATTGTA
ATGCATGATTACTTAACAGGGGGGTTCACTGCAAATACTAGCTTGGCTCA
TTATTGCCGAGATAATGGTCTACTTCTTCACATCCACCGTGCAATGCACG
CAGTTATTGATAGACAGAAAAATCATGGTATACACTTTCGTGTACTAGCT
AAAGCGTTACGTATGTCTGGTGGAGATCATATTCACTCCGGTACTGTAGT
AGGTAAACTTGAAGGGGAAAGAGACATCACTTTAGGCTTTGTTGATTTAC
TACGTGATGATTTTATTGAAAAAGATCGAAGCCGTGGTATTTATTTCACT

CAAGATTGGGTCTCTCTACCAGGTGTTCTGCCTGTGGCTTCAGGGGGTAT
TCATGTTTGGCATATGCCTGCTCTGACCGAGATCTTTGGAGATGATTCCG
TACTACAGTTCGGCGGAGGAACTTTAGGACACCCTTGGGGAAATGCACCC
GGTGCCGTAGCTAATCGAGTAGCTCTAGAAGCATGTGTACAAGCTCGTAA
TGAGGGACGTGATCTTGCTCGTGAGGGTAATGAAATTATCCGTGAGGCTA
GTAAATGGAGTCCCGAACTAGCTGCTGCTTGTGAAGTATGGAAGGAGATC
AAATTTGAATTCGAAGC---------------------------------

---AGCTGAG-CTATCCCGACCATTCACGACGCATCATCC----------
--TCAT-TTTAATAGATGACTTGGGTCTATGTCAATTAAAAAGACGAAAA
GAGGATTACAAAGTCTTATCCAGGCCTTGGTGGAATTTCTTAGATCTTCA
A----------------------------AA---AGAAGA-CTTTG---
TAAGTTTCAATACAGTACGGGCGATAATATGATCA---------------

----------------------------------TTCCAATTT-AC----
------AAT---------CGGGGTTACTTGCTCAATGATGTTCATTTGTA
CGTGTATCATATATATCGGCT---TGTGAAAATTAAAAAATTAATGAATG
AGAAACATAAGGAATTTTGAACCGCTAAC---GAAATGAGAGTATAGGAT
AAAAAATTAGGGAATCAAATG--G-GTG---GGG--ATAGAGGGACTTGA
ACC---

--CATT
TAGAGGAAGGAGAAGTCGTAACAAGGTTTCCGTAGGTGAACCTGCGGAAG
GATCATTGTCGAAA-CCT-GCTTAGCAGAACGACCCGCGAACATGTGAAA
ACATATTGGGGG---GCGAGG---GGCCTTAGGT--CTC-GGACTCTCT-
TGGTCGG--GGGGTTG---CATCCTGTGCCTCGCTTGCCGATGCATTGGT
GCTT--CTCTCCAGACCAC-ACAAACGAACCCCGGCGCCGAAACG--CGC
CAAGGAAAATCTAACGAAGTGGCAC-CGCCCCTCCGCCCCAGAGATGGTG
TGCGGTCGGGGCGTGCGT--CGTATTC---TATATGTCAAAACGACTCTC
GGCAACGGATATCTCGGCTCTCGCATCGATGAAGAACGTAGCGAAATGCG
ATACTTGGTGTGAATTGCAGAATCCCGTGAACCATCGAGTCTTTGAACGC
AAGTTGCGCCCGAAGCCA--TTAGGCCGAGGGCACGTCTGCCTGGGCGTC
ACACAACGTTGCCCCCCCACCCCACTCTGGGC------------------
-------------GTGAGGGGGGCAGATGCTGGCCTCCCGTGTGCCACGG
CATG--CGGCTGGTTGAAAT--ATGAGTCCTCGGCGGCGAGTGCCGCAAC
AATCGGTGGTTCTCCAACCCTCG-TGGCCCTGTTGCGTGCATAGACTGCT
GCTGTGGCTCGACAGACC---CCAT-TG-TGC---TGCTAATGCAGCGTA
TCCAACGGGACCCCAGGTCAGGCGGGGCTACCCGCTGAGTTTAAGG----

>Nesiota_elliptica

-------------------------------AAGTGTTGGATTCAAAGCC
GGCGTTAAAGATTATAAATTGACTTATTACACTCCTGACTATGAAACCAA
AGATACTGATATCTTGGCAGCGTTTCGAGTAACTCCTCAACCTGGAGTTC
CGCCTGAGGAAGCAGGGGCCGCGGTAGCTGCTGAATCTTCTACTGGTACA
TGGACAACTGTATGGACTGACGGGCTTACCAGTCTTGATCGTTACAAAGG
TCGATGCTACCACCTCGAGCCCGTTGCTGGAGAAGAAAATCAATATATTG
CTTATGTAGCTTACCCCTTAGACCTTTTTGAAGAAGGTTCTGTTACTAAC
ATGTTTACTTCCATTGTGGGTAATGTTTTTGGGTTCAAAGCCCTGCGCGC
TCTACGTCTGGAGGATTTGCGAATCCCTCCTGCTTATTCTAAAACTTTCC
AAGGACCGCCTCACGGCATCCAAGTTGAAAGAGATAAATTGAACAAGTAT
GGCCGTCCCCTATTGGGATGTACTATTAAACCTAAATTGGGGTTATCCGC
TAAGAATTACGGTAGAGCAGTTTATGAATGTCTTCGCGGTGGACTTGATT
TTACCAAAGATGATGAGAACGTGAATTCCCAACCATTTATGCGTTGGAGA
GACCGTTTCTTATTTTGTGCCGAAGCCCTTTATAAAGCACAGGCTGAAAC
AGGTGAAATCAAAGGGCATTACTTGAATGCTACTGCAGGTACATGCGAAG
AAATGATTAAAAGGGCTGTATTTGCCAGAGAATTGGGAGTTCCTATTGTA
ATGCATGATTACTTAACAGGGGGATTCACTGCAAATACTAGCTTGGCTCA
TTATTGCCGAGATAATGGTCTACTTCTTCACATCCACCGTGCAATGCACG
CAGTTATTGATAGACAGAAAAATCATGGTATACACTTTCGTGTACTAGCT
AAAGCGTTACGTATGTCTGGTGGAGATCATATTCACTCTGGTACTGTAGT

AGGTAAACTTGAAGGGGAAAGAGACATCACTTTAGGCTTTGTTGATTTAT
TACGTGATGATTTTGTTGAAAAAGATCGAAGTCGTGGTATTTATTTCACT
CAAGATTGGGTCTCTCTACCAGGTGTTTTGCCTGTGGCTTCAGGGGGTAT
TCATGTTTGGCATATGCCTGCTCTGACCGAGATCTTTGGAGACGATTCCG
TACTACAGTTCGGCGGAGGAACTTTAGGACACCCTTGGGGAAATGCACCC
GGTGCCGTAGCTAATCGAGTAGCTCTAGAAGCATGTGTACAAGCTCGTAA
TGAGGGACGTGATCTTGCTCGTGAGGGTAATGAAATTATCCGTGAGGCTA
GTAAATGGAGTCCTGAACTAGCTGCTGCTTGTGAAGTATGGAAGGAGATC
AAATTTGAATTCGAAGCAATGGATACTTTGTAA-----------------
--A-------
-CCAGCTGAG-CTATCCCGACCATTCACGACGCATCATCC----------
--TCAT-TTTAATAGATGACTTGGGTCTATGTCAATTAAAAAGACGAAAA
GGGGATTACAAAGTCTTATCCAGGCCTTGGTGGAATTTCTTAGATCTTCA
A-----------------------------AA---CGAAGA-CTTTG---
TAAGTTTCAATACAGTACGGGCGATAATATGATCA---------------

----------------------------------TTCCAATTT-AC----
------AAT---------CGGGGTTACTTGCTCAATGATGTTCATTTGTA
CGTGTATCATATATATCGGCT---TGTGAAAATAAAAAAATGAATGAATG
AGAAACATAACGAATTTTGAACCGCTAAC---GAAATGAGAGTATAGGAT
AAAAAATTAGGGAATCCAAAT--GGGTG---GGG--ATAGAGGGACTTGA
ACCCTCACGATTTCTAAAGTCGACGGATTTTCCTCTTACTATAAATTTCA
TTGTTGTCGATATTGACATGTAGAATGGGACTCTATCT------TTATTC
TCGTCCGATTAATCCATTTTTCAA----AAGATCTAT-CAGATTACGATG
GAGTAAATGATTTGATCAACGAATAT----TCCAGACAGTATTTCAGTAC
GT---ATACGTATATATAGGTTTATCCTTCATCCTTTCTGGAATTTCGAT
GGAAGGATTC--------------------------------------CT
TTACTAACGCATCGCAGCCAACTCCA----TTTGTTAGAACAGCTTCCAT
TGAGT--------CTCTGCACCTATCCTTTTTTATTATCACTTTCTGAAT
CCTTGTTTGTTTTCAGA---------------AAAAAGGATTTGGCTCAG
GATTGCCCATTTGT------AATTCCAGGT-TTCTCTGAATTTGAAAGTT
A-------------TCACTTGGTAGTTTCC--------------------

-----------------AATCGTACAAGTTCGTAGG-TGAACTGCGGAAG
GATCATTGTCGAAA-C-T-GCTCAG-CAGAACGACCGCGAACCTGTGAAA
ATATACTGGGGG---GCGAGG---GGCCTTAGGT--CTC-GGACTCTCT-
TGGTCGG-GGGGGCTG---CATCCCGTGCCTCGCTTGCCGATGCATTGGT
GTTG--CTTTCCAGGCCAC-ACAAACGAACCCCGGCGCCAAATCG--CGC
CAAGGAAAATCTAACGAAGTGGCAC-CGCCCTTCCGCCCCAGAAATGGGG
TGCGGTAAGGGTGTGCGT--CGTATTC---TATATGTCAAAACGACTCTC
GGCAACGGATATCTCGGCTCTCGCATCGATGAAGAACGTAGCGAAATGCG
ATACTTGGTGTGAATTGCAGAATCCCGTGAACCATCGAGTCTTTGAACGC
AAGTTGCGCCCGAAGCCTAATTAGGCCGAGGGCACGTCTGCCTGGGCGTC
ACACAACGTTGCCCCCTCGACTC----CAACG------------------
-------------GTGAGGGGGGCAGATGTTGGCCTCCCGTGTGCCACGG
CATG--CGGCTGGTTGAAAT--ACGAGTCCTCGGCGATGAGTGCCGCAAC
AATCGGTGGTTATCCAACCCTCG-TGGCCCTGTTGCGTGCATAGACCTCT
GCTGTGGCTCGATAGACC---CCAT-TG-CGT---TGCTAATGCAGCGTA
TCCAACGCGACCCCAGTCAGCGGGTACCCTGAGTT---------------

>Discaria_chacaye

-----ATGTCACCACAAACAGAGACTAAAGCAAGTGTTGGATTCAAAGCC
GGTGTTAAAGATTATAAATTGACTTATTACACTCCTGACTATGAAACCAA
AGATACTGATATCTTGGCAGCGTTTCGAGTAACTCCTCAACCTGGCGTTC
CGCCTGAGGAAGCAGGGGCCGCGGTAGCTGCTGAATCTTCTACTGGTACA
TGGACAACTGTATGGACTGACGGGCTGACCAGTCTTGATCGTTACAAAGG
TCGATGCTACCACCTCGAGCCCGTTGCTGGAGAAGAAAATCAATATATTG
CTTATGTAGCTTACCCCTTAGACCTTTTTGAAGAAGGTTCTGTTACTAAC
ATGTTTACTTCCATTGTGGGTAATGTTTTTGGGTTCAAGGCCCTGCGCGC
TCTACGTCTGGAGGATTTGCGAATCCCCCCTGCTTATTCTAAAACTTTCC
AAGGACCGCCTCACGGCATCCAAGTTGAAAGAGATAAATTGAACAAGTAT
GGCCGTCCCCTATTGGGATGTACTATTAAACCTAAATTGGGGTTATCCGC
TAAGAATTACGGTAGAGCAGTTTATGAATGTCTTCGCGGTGGACTTGATT
TTACCAAAGATGATGAGAACGTGAATTCCCAACCATTTATGCGTTGGAGA
GACCGTTTCTTATTTTGTGCCGAAGCCCTTTATAAAGCACAGGCTGAAAC
AGGTGAAATCAAAGGGCATTACTTGAATGCTACTGCAGGTACATGCGAAG
AAATGATTAAAAGGGCTGTATTTGCCAGAGAATTGGGAGTTCCTATTGTA
ATGCATGATTACTTAACAGGGGGATTCACTGCAAATACTAGCTTGGCTCA
TTATTGCCGAGATAATGGTCTACTTCTTCACATCCACCGTGCAATGCATG

CAGTTATTGATAGACAGAAAAATCATGGTATACACTTTCGTGTACTAGCT
AAAGCGTTACGTATGTCTGGTGGAGATCATATTCACTCTGGTACTGTAGT
AGGTAAACTTGAAGGGGAAAGAGACATCACTTTAGGCTTTGTTGATTTAC
TACGTGATGATTTTATTGAAAAAGATCGAAGCCGTGGTATTTATTTCACT
CAAGATTGGGTCTCTCTACCAGGTGTTCTGCCTGTGGCTTCAGGGGGTAT
TCATGTTTGGCATATGCCTGCTCTGACCGAGATCTTTGGAGATGATTCCG
TACTACAGTTTGGCGGAGGAACTTTAGGACACCCTTGGGGAAATGCACCC
GGTGCCGTAGCTAATCGAGTAGCTCTAGAAGCATGTGTACAAGCTCGTAA
TGAGGGACGTGATCTTGCTCGTGAGGGTAATGAAATTATCCGTGAGGCTA
GTAAATGGAGTGCTGAACTAGCTGCTGCTTGTGAAGTATGGAAGGAGATC
AAATTTGAATTCGAAGCAATGGATACTTTGTAA-----------------
----------------------TGGTATGGAACCTACCAAGTGATAACTT
TCAAATTCAGAGAAACCCTGGAATTACAAATGGGCAATCCTGAGCCAAAT
CCTGTTTTCTGAAAACAAACAAGGATTCA-------GAA---A---GTGA
TAATAAAAAAGGATAGGTGCAGAGACTCAATGGAAGCTGTTCTAACAAAT
GG----AGTTGGCTGCGATGCGTT------AGTAAAGGAAT-CCTTCCAT
CCAAACTCCAGAAAGGATGAAGAATAAACCTATATATAC------GTACT
GAAATACTATCTCCAAACCAAATGATTAAAGACGACCCGAATCTTTTTTT
TATATTTATAT------GTTTATATATATGTTTAT-ATAAAAAATAAAAA
ATGAAAGAATTGTTGTGAA-----TCG------ATTCCAAGTAAAAAAAA
AAATGGAATATTCATTGATCAAATCATTTACTCCACCGTAATCTGAT---
-AGATCTTTTGAAAAATGGAT------TAATCGGACGAGAATAAAGATAG
AGTCCCATTCTACATGTCAA------------TATCGACAACAATGAAAT
TTATAGTAAGAGGAAAATCCGTCGACTTTA-GAAATCGTGAGGGTTCAAG
TCCCTCTATCCCCAAAAAGGC-CCATTTGATTCCCTAATTTTTTATC---
--CTA-----TACTCTCATTTCGTTAGCGGTTCAAAATTCGTTAGGTTTC
TCATTC------ATTCATT-TTTT--------------------------
------TCT--TTTCACAAGCCTTGT----GATATATATGATACACG---
----TAC--------AAATGAACATCA--TTGAGCAAGT--AACCCCGAT
TGTAAATTGA--------ATGATC-AGATT--------ATCGCTCGTACT
GTATTGAAA----CTTACAAAATCTTCT-------TGTTGAAGATCTAAC
AAATT----CCACCACGGCCTGGATAAGACTTTGTAAT----CCCCTTTT
CGTTTTTTTAATTGACATAGACCCAAGTCCTCTATTAA------AATGAG
GATGATGCGTCGTG-----AATGGTCGGGATAGCTCAGCTGGT-------

>Blackallia_biloba

----TTAATNGGNTTGAGCCTTGGTATGGAAACCTACCAAGTGATAACTT
TCAAATTCAGAGAAACCCTGGAATTACAAATGGGCAATCCTGAGCCAAAT
CCTGTTTTATGAAAACAAACAAGTATTTAGTATTTAGAA---A---GTGA
TAATAAAAAAGGATAGGTGCAGAGACTCAATGGAAGCTGTTCTAACAAAT
GG----AGTTGGCTGCGATGCGTT------AGTAAAGGAAT-CCTTCCAT
CGAAACTCCAGAAAGGATGAAGAATAAACCTATATATACGTATACGTACT
GAAATACTATCTCCAAACCAAATGATTAATGATGACCCGAATTTTTTTTT
TTTTTTTACTT------GGAATCGA-------------------------
-----------------------------TTCCAAGTAAAAAAAAAAAAA
AAATTCAAAATTCATTGATCAAATCGTTTACTCCATCGTAATCTGAT---
-AGATCTTTTGAAAAATGGAT------TAATCGGACGAGAATAAAGATAG
AGTCCCATTCTACATGTCAA------------TATCGACAACAATGAAAT
TTATAGTAAGAGGAAAATCCGTCGACTTTA-GAAATCGTGAGGGTTCAAG
TCCCTCTATCCCCAAAAAGGC-CCGGTTGATTCCCTCATTTTTTATC---
--CTATACT-CACTCTCATTTCGTTATCGGTTCAAAATTCATTATGTTTC
TCATTCATTAA---------------------------------------
--TTTTTTTCTTTTCACAAGCCTTGT----GGTATATATGATACACA---
----TAC--------AAATGAACATCA--TTGAGCAAGT--AACCTCGAT
TGTAAATTGG-------AATCATC-ATATT--------ATCGCCCGTACT
GTACTGAAA----CTTACAAAGTCTTT--------TTTTTAAGATCTAAG
AAATT----CCGCCAAGGCCTGGATAACACTTTGTAAT----CCCCTTTT
CGTCTTTTTAATTGACATAGACCCAAGTCATCTATTAA------AATGAG
GGTGATGCGTCGTG-----AATGGTCGGGATAGCTCAGCTGGTAGAGCAG
AGGACTGAAAATCCTCGTGTCACCAGTC----------------------

--AG
AATCATTGTCGAACCTCT-GCCTAGCAGAACGACCCGCGAACCTGTAAAT
AAC--ATATGAGGGAGCTTGG---GGCCCCAGGC--CAC-GGGCTCTCT-
TGGTCGGG-GGCTGCA---CTCTGTG--CGTCGCTTGCCGAGGCACTGGT
GCTTTGCTTCCCCGGCTGC--ACAAACGAACCCCGGCGCAAACCGCGCCA
AGGAA--CATCAAACGAATCAGCAC-CGCCCCGCCACCCCAGAGATGGTG
TGTGGTCGGGGTGGACGT--CGTATTC--AATTAAGTCAAAACGACTCTC
GGCAACGGATATCTCGGCTCTCGCATCGATGAAGAACGTAGCGAAATGCG
ATACTTGGTGTGAATTGCAGAATCCCGTGAACCATCAAGTCTTTGAACGC
AAGTTGCGCCCGAAGCCA--TTAGGCCGAGGGCACGTCTGCCTGGGCGTC
ACACAATGTTGCCCCCCAACCTCGATC-TC-GA-----------------
----------AG---GCGGGGGCGGATGCTGGCCTCCCGTGCCTTACGG
CTTG--CGGCTGGCCGAAAT--GCGAGTCCCCGGCGACGAGCGCCGCAGC
AATCGGTGGTTGTCAAACCCTCG-GTGCCCTGCTGCGTGCACGAATCGCT
GCCGTGGAAAGCTAGACC---CCAA-TGCGC----CGCATATGCGGCGTC
TACAATGCGACCTNAGGTCAGGC---------------------------

>Polianthion_wichurae

----------------------TGGTATGGAACCTACCAAGTGATAACTT
TCAAATTCAGAGAAACCCTGGAATTACAAATGGGCAATCCTGAGCCAAAT
CCTGTTTTCTGAAAACAAACAAGTATTCA-------GAA---A---GTGA
TAATAAAAAAGGATAGGTGCAGAGACTCAATGGAAGCTGTTCTAACAAAT
GG----AGTTGGCTGCGATGCGTT------AGTAAAGGAAT-CCTTCCAT
CGAAACTCCAGAAAGGATGAAGAATAAACCTATATATACGTATACATACT
GAAATACTATCTCCAAACCAAATGATTAATGACGACCCGAATATTTTTAG
TTTTTTTTTTTT------TTATATGTTTATATGAAA-------------A
ATGAAAGAATTGTTGTGAA-----TCGATTCCAAGTAAAAAAA----AAA
AAATGGAATATTCATTGATCAAATCATTTACTCCATTGTAATCTGAT---
-AGATCTTTTGAAAAATGGATTAAGATTAATCGGACGAGAATAAAGATAG
AGNCCCATTCTACATGTCAA------------TATCGACAACAATGAAAT
TTATAGTAAGAGGAAAATCCGTCGACTTTA------------GGTTCAAG
TCCCTCTATCCCCAAAAAGGC-CCTGTTGATTCCCTAATTTTTTATC---
--CTATACT-C----TCATTTCGTTATCGGTTCAAAATTCATTATGTTTC
TCATTC------ATTAATT-TTTT--------------------------
------TCT--TTTCACAAGCCTTGT----GGTATATATGATACACA---
----TAC--------AAATGAACATCA--TTGAGCAAGT--AACCCCGAT
TGTAAATTGG-------AATGACC-ATATT--------ATCGCCCGTACT
GTACTGAAA----CTTACAAAGTCTTTTTT----TTTT--AAGATCTAAG
AAATT----CCACCAAGGCCTGGATAACACTTTGTAAT----CC-CTTTT
CGTCTTTTTAATTGACATAGACCCAAGTCATCTATTAA------AATGAG
GATGATGCGTCGTG-----AATGGTCGGGATAGCTCAGCTGGTAGAGCAG
AGGACTGAAAATCCTCGTGTCA----------------------------

--------GTCGAAACCT-GCCTAGCAGGACGACCCGCGAACCTGTAAAA
AACA--TCTCGGGGGGGCTTGGG--GCCTTAGGCT--CTG-TGCCCTCTT-
TGGTCGGG-GGTTGCA---CGCTGTG--TGTCGCTTGCCGATGCATCGGT
CCCTGCTTCCCCCGGCTGC--ACAAACGAACCCCGGCGCAAACCGCGCCA
AGGAAAGCATAAAACGAATAAGCAC-CACCCCGTCACCCCAGAGATGGTG
TGTGGTCGGGGTGTGCGT--CGTATTC--AACTAAGTCAAAACGACTCTC
GGCAACGGATATCTCGGCTCTCGCATCGATGAAGAACGTAGCGAAATGCG
ATACTTGGTGTGAATTGCAGAATCCCGTGAACCATCAAGTTTTTGAACGC
AAGTTGCGCCCGAAGCCA--TTAGGCCGAGGGCACGTCTGCCTGGGCGTC
ACACAATGTTGCCCCCAACCTCGAGATCTCGAA-----------------
-----------GG---CGGGGGGCGGATGCTGGCTTCCCGTGCCTTACGG
ATTG--CGGATGGCCGAAAT--GCGAGTCCTCGGCGACGAGTGCCGCAGC
AATCGGTGGTTCTCCAACCCTCG-GTGCCCTGCTGCGTGCGCGGATCGCC
GTTGTGGACATATAGACC---CCAA-TGCGC----TGCAGATG-------

